# Effects of human papillomavirus (HPV) vaccination programmes on community rates of HPV‐related disease and harms from vaccination

**DOI:** 10.1002/14651858.CD015363.pub2

**Published:** 2025-11-24

**Authors:** Nicholas Henschke, Hanna Bergman, Brian S Buckley, Emma J Crosbie, Kerry Dwan, Su P Golder, Maria Kyrgiou, Yoon Kong Loke, Heather M McIntosh, Katrin Probyn, Gemma Villanueva, Jo Morrison

**Affiliations:** Cochrane ResponseThe Cochrane CollaborationLondonUK; Department of SurgeryUniversity of the PhilippinesManilaPhilippines; Division of Cancer Sciences, Faculty of Biology, Medicine and HealthUniversity of ManchesterManchesterUK; Centre for Reviews and DisseminationUniversity of YorkYorkUK; Department of Health SciencesUniversity of YorkYorkUK; IRDB, Department of Gut, Metabolism & Reproduction - Surgery & CancerImperial College LondonLondonUK; Norwich Medical SchoolUniversity of East AngliaNorwichUK; Department of Gynaecological OncologyMusgrove Park HospitalTauntonUK; Faculty of Health and Life SciencesUniversity of ExeterExeter, DevonUK

## Abstract

**Background:**

Human papillomavirus (HPV) vaccination has the potential to enhance prevention of cervical cancer, especially in countries where screening programmes are currently unaffordable or impractical. Rare adverse events and longer‐term benefits of HPV vaccination, such as effects on cancer rates, are difficult to examine in randomised controlled trials (RCTs) and require large data from population‐level studies to inform decision‐making.

**Objectives:**

We aimed to assess population‐level effects of HPV vaccination programmes on HPV‐related disease and harms from vaccination.

**Search methods:**

We conducted electronic searches on 11 September 2024 in CENTRAL (*Cochrane Library*), Ovid MEDLINE and Ovid Embase. We also searched vaccine manufacturer websites and checked reference lists from an index of HPV studies and other relevant systematic reviews.

**Selection criteria:**

We included studies that assessed the impact of HPV vaccination on the general population. This included population‐level studies comparing outcomes before and after the introduction of HPV vaccine. We also included individual‐level, non‐randomised comparative studies, such as cohort studies, case‐control studies, cross‐sectional studies and self‐controlled case series.

**Data collection and analysis:**

We used methods recommended by Cochrane. Two review authors carried out data extraction independently using pretested data extraction forms. We assessed the risk of bias of all included effect estimates using different tools according to study design. We carried out quantitative and qualitative data synthesis separately by outcome and study design. We performed meta‐analysis on studies that reported effect estimates adjusted for confounding, with a focus on those receiving HPV vaccination at or before the age of 16 years (the target age group for vaccination). We rated the certainty of the evidence with GRADE.

**Main results:**

We included 225 studies from 347 records in this review, evaluating over 132 million people. We included 86 cohort studies, four case‐control studies, 46 cross‐sectional studies, 69 pre‐post vaccine introduction studies, five RCT extensions and two self‐controlled case series. Thirteen additional studies reported on more than one type of analysis. Of the included studies, 177 reported only on females, 11 only males and 37 a combination of males and females. Risk of bias ranged from overall moderate risk to critical risk.

**Clinical outcomes**

There was moderate‐certainty evidence from 20 studies that HPV vaccination reduces the incidence of cervical cancer. Five cohort studies including 4,390,243 females reported adjusted estimates showing a reduced risk of cervical cancer following HPV vaccination in the long term (risk ratio (RR) 0.37, 95% confidence interval (CI) 0.25 to 0.56; I^2^ = 88%). There was a significant interaction with age at vaccination, with a greater risk reduction in younger people. For those vaccinated at or before 16 years of age, covering 4.54 million person‐years, there was an 80% reduced risk of cervical cancer (RR 0.20, 95% CI 0.09 to 0.44; I^2^ = 69%). One cohort study, one case‐control study, one cross‐sectional study and three RCT extension studies all reported no cases of cervical cancer in the HPV vaccine groups. Eight pre‐post vaccine introduction studies each reported a reduction in cervical cancer incidence following HPV vaccine introduction but did not provide data in a form that allowed for meta‐analysis.

There was moderate‐certainty evidence from 23 studies that HPV vaccination reduces the incidence of cervical intraepithelial neoplasia grade 3 or higher (CIN3+), including 12 cohort studies. For 1.5 million females vaccinated at or before the age of 16 years in two cohort studies, there was a reduction of CIN3+ incidence of 74% in the long term (RR 0.26, 95% CI 0.12 to 0.56; I^2^ = 80%). Three case‐control studies, one RCT extension study and three cross‐sectional studies also reported a decreased risk of CIN3+ in vaccinated participants. One cross‐sectional study reported no difference in the risk of CIN3+. Three pre‐post vaccine introduction studies reported a decrease in CIN3+ incidence following HPV vaccine introduction.

There was moderate‐certainty evidence from 37 studies that HPV vaccination reduces the incidence of CIN2+. In cohort studies with females vaccinated at or before the age of 16 years, a reduction in risk was seen in the medium term (RR 0.59, 95% CI 0.54 to 0.65; 2 cohort studies, 233,468 females; I^2^ = 0%) and long term (RR 0.38, 95% CI 0.31 to 0.45; 5 cohort studies, 6,455,176 females; I^2^ = 64%).

There was moderate‐certainty evidence from 47 studies that HPV vaccination reduces the incidence of anogenital warts. From the cohort studies with adjusted estimates, the pooled impact of HPV vaccination on rates of anogenital warts indicated a reduction of 47% in the medium term (RR 0.53, 95% CI 0.37 to 0.77; 4 studies, 6,430,295 females and 313 males; I^2^ = 98%) and 53% in the long term (RR 0.47, 95% CI 0.36 to 0.61; 13 studies, 4.5 million person‐years plus 5,802,969 females and males; I^2^ = 99%). Twenty‐three pre‐post vaccine introduction studies reported a decrease in anogenital warts incidence following the introduction of HPV vaccine. Six studies reported no difference in anogenital warts incidence.

There was only very low‐certainty evidence on the effect of HPV vaccination on the incidence of adenocarcinoma in situ (three studies) and vulval cancer (five studies). No studies were identified that reported on community rates of serious adverse events following HPV vaccination.

**Specific adverse events**

Across a range of study designs, HPV vaccination was not associated with an increased risk of postural orthostatic tachycardia syndrome, chronic fatigue syndrome/myalgic encephalomyelitis, paralysis, complex regional pain syndrome, premature ovarian failure, infertility or sexual activity (all moderate‐certainty evidence). There was evidence that suggests HPV vaccination was not associated with an increased risk of Guillain‐Barré syndrome (low‐certainty evidence).

**Authors' conclusions:**

There are now long‐term outcome data from different countries and from different study designs that consistently report a reduction in the development of high‐grade CIN and cervical cancer in females vaccinated against HPV in early adolescence. Data show that there is greater benefit to vaccinating younger adolescents prior to becoming sexually active. There is evidence that HPV vaccination does not increase the risk of the most common adverse events reported on social media.

## Plain language summary

**What are the benefits and risks of different human papillomavirus (HPV) vaccines for preventing cervical cancer and other HPV‐related disease?**

**Key messages**

HPV vaccination:

‐ reduces the incidence of cervical cancer by around 80% in people vaccinated at or before the age of 16 years;

‐ reduces the incidence of high‐grade cervical pre‐cancer lesions, as well as anogenital warts;

‐ is not associated with an increased risk of long‐term side effects or infertility;

‐ is more effective when given at or before the age of 16 years, before onset of sexual activity.

**What is HPV?**

Human papillomavirus (HPV) is transmitted between people through sexual contact, including vaginal, anal or oral sex. There are many types of HPV. Some types are harmless, but other types can cause cancer. Cervical cancer is the most common type of cancer that HPV can cause, but it can also cause vaginal, vulval, penile, anal, and head and neck cancer, as well as anogenital warts (a sexually transmitted infection caused by certain types of human papillomavirus). From the time of HPV infection, cervical cancer usually takes more than 10 years to develop, and other cancers take longer.

**How can HPV vaccines be beneficial?**

In girls and boys, HPV vaccines aim to prevent HPV infection, which can sometimes cause cancer and anogenital warts. The HPV vaccines do not work well in people that have already been exposed to HPV. For this reason, most vaccination programmes aim to offer the vaccine to young people before they become sexually active.

**What did we want to find out?**

We wanted more information on questions about long‐term and rare outcomes that cannot be answered by randomised controlled trials (studies where people are assigned randomly to two or more treatment groups):

‐ What are the effects of introducing HPV vaccination on community rates of cervical, vaginal, vulval, anal and penile cancer, and the pre‐cancerous stages of disease during the development of cancer?

‐ What are the effects of introducing HPV vaccination on the number of people who develop anogenital warts and the number of people who undergo treatment for HPV‐related disease?

We also wanted to know if HPV vaccines were associated with any harmful effects, especially those discussed most frequently on social media.

**What did we do?**

We searched for studies that evaluated the impact of HPV vaccination on population levels of cervical and other cancers, high‐grade pre‐cancer lesions (abnormal cell changes that occur after a persistent high‐risk HPV infection and can develop into cancer if untreated), anogenital warts, treatment rates, HPV infections and unwanted or harmful (adverse) events. These included studies following groups of people after receiving HPV vaccination and studies observing the change in these diseases after national‐level introduction of HPV vaccination.

We also searched social media sites (WebMD and X (formerly Twitter)) for commonly mentioned adverse events related to HPV vaccination. We searched for and included studies evaluating the impact of HPV vaccination on these events.

**What did we find?**

We found 225 suitable studies from around the world that reported on the benefits and harms of HPV vaccination, including over 132 million people.

HPV vaccination probably reduces the incidence of cervical cancer by around 80% in people vaccinated at or before the age of 16 years. The reduction is lower for people vaccinated later.

HPV vaccination probably reduces the incidence of high‐grade cervical pre‐cancer lesions (CIN3+, CIN3, CIN2+ and CIN2), as well as anogenital warts. Again, reductions are greater in people who received the HPV vaccine at or before the age of 16 years.

There was lower‐certainty evidence for the effect of HPV vaccination on rare diseases that take much longer to develop, such as adenocarcinoma in situ, other pre‐cancer lesions and other cancers related to HPV (e.g. vaginal, vulval, anal and penile cancer). We identified fewer studies on these outcomes.

For most of the specific adverse events we looked at, including postural orthostatic tachycardia syndrome, chronic fatigue syndrome/myalgic encephalomyelitis, paralysis, complex regional pain syndrome, Guillain‐Barré syndrome and infertility, there was moderate‐certainty evidence that HPV vaccination likely does not increase the risk of developing them. HPV vaccination also did not increase sexual activity.

HPV vaccination also appears to reduce treatment rates associated with HPV disease, increases attendance at cervical screening programmes and reduces HPV infections.

**What are the limitations of the evidence?**

We are moderately confident in our results for cervical cancer, high‐grade cervical disease, anogenital warts and specific harms. However, better and larger studies could show more reliable and precise results about the amount of protection.

**How up‐to‐date is this evidence?**

The evidence is up‐to‐date to September 2024.

## Summary of findings

**Summary of findings 1:** Summary of findings – clinical outcomes

**Population:** general population of any age **Setting:** any setting **Intervention:** full or partial series HPV vaccination **Comparator:** no vaccination				
**Outcome**	**Number of studies (participants)**	**Summary of effect**	**Overall certainty of the evidence**	**Interpretation of findings**
**Invasive cervical cancer**	Six cohort studies (4,419,387 females plus 27,946 cases of cervical cancer) One case‐control study (12,296 females) Three RCT extension studies (47,456 females) One cross‐sectional study (1392 females) Nine pre‐post vaccine introduction studies (> 1,030,882 cases of cervical cancer)	Of the six cohort studies, five reported a reduced risk of cervical cancer following HPV vaccination (RR 0.37, 95% CI 0.25 to 0.56). One cohort study did not report any cases of cervical cancer in the HPV vaccine group. The case‐control study, cross‐sectional study and the three RCT extension studies all reported no cases of cervical cancer in the HPV vaccine groups. All nine pre‐post vaccine introduction studies reported a reduction in cervical cancer incidence between the pre‐ and post‐introduction periods.	MODERATE^a^ ⊕⊕⊕◯ Downgraded due to methodological limitations	HPV vaccination probably reduces the incidence of cervical cancer.
**Adenocarcinoma in situ (AIS)**	One cross‐sectional study (1392 females) Two pre‐post vaccine introduction studies (> 5475 cases of AIS)	The cross‐sectional study reported no cases of AIS in the HPV vaccine group. One pre‐post vaccine introduction study reported an increase in AIS incidence between the pre‐ and post‐introduction period, while the other reported a reduction.	VERY LOW^b,c,d^ ⊕◯◯◯ Downgraded due to serious methodological limitations, inconsistency and imprecision	We are unclear about the effect of HPV vaccination on AIS incidence because the certainty of the evidence is very low.
**Cervical intraepithelial neoplasia grade 3 or higher (CIN3+)**	Twelve cohort studies (3,105,713 females) Three case‐control studies (26,595 females) One RCT extension study (3148 females) Five cross‐sectional studies (219,953 females) Three pre‐post vaccine introduction studies (116,139 females)	Of the 12 cohort studies, one did not report any cases of CIN3+. One study reported a reduction in the medium term (RR 0.43, 95% CI 0.35 to 0.53) and seven studies showed a reduction in the long term (RR 0.39, 95% CI 0.32 to 0.48). Three case‐control studies reported a reduced risk of CIN3+ in vaccinated participants. The RCT extension study reported a decrease in CIN3+ incidence in vaccinated participants. Four cross‐sectional studies reported a decreased risk of CIN3+ in vaccinated participants. One cross‐sectional study reported no difference in risk of CIN3+. Three pre‐post vaccine introduction studies reported a decrease in CIN3+ incidence between the pre‐ and post‐introduction periods.	MODERATE^e^ ⊕⊕⊕◯ Downgraded due to methodological limitations	HPV vaccination probably reduces the incidence of CIN3+.
**Invasive vulval cancer**	One RCT extension study (189,901 person‐years) Four pre‐post vaccine introduction studies (> 36,563 cases of vulval cancer)	The RCT extension study reported no cases of vulval cancer in vaccinated participants. Two pre‐post‐vaccine introduction studies reported a decrease in vulval cancer incidence between the pre‐ and post‐introduction periods, while one reported an increase. The other study reported inconsistent results, with some ethnic groups seeing an increased incidence and others a decrease.	VERY LOW^f,g,h^ ⊕◯◯◯ Downgraded due to methodological limitations and serious imprecision	We do not know about the effect of HPV vaccination on vulval cancer incidence because the certainty of the evidence is very low.
**Cervical intraepithelial neoplasia grade 2 or higher (CIN2+)**	Fourteen cohort studies (7,059,815 females) Three case‐control studies (142,073 females) Two RCT extensions (11,675 females) Eleven cross‐sectional studies (205,994 females) Seven pre‐post vaccine introduction studies (4,914,524 females)	Twelve cohort studies reported a reduced risk of CIN2+ following HPV vaccination (RR 0.51, 95% CI 0.37 to 0.69) and one reported no difference in risk of CIN2+ between vaccinated and unvaccinated participants. One cohort study did not report any cases of CIN2+ in the HPV vaccine group. Three case‐control studies all reported reduced odds of CIN2+ in vaccinated participants. One RCT extension study reported no cases of CIN2+ in the vaccinated participants and the other reported a decrease in CIN2+ with HPV vaccine. Three cross‐sectional studies reported a reduced risk of CIN2+ in vaccinated participants (RR 0.47, 95% CI 0.34 to 0.64). Five cross‐sectional studies reported no difference in risk between vaccinated and unvaccinated participants. Three cross‐sectional studies reported no cases of CIN2+ in the vaccinated participants. Six pre‐post vaccine introduction studies reported a reduction in CIN2+ incidence between the pre‐ and post‐introduction periods and one study reported an increased incidence.	MODERATE^i^ ⊕⊕⊕◯ Downgraded due to methodological limitations	HPV vaccination probably reduces the incidence of CIN2+.
**Anogenital warts**	Fifteen cohort studies (5,226,044 person‐years plus 12,035,299 females and males) Three cross‐sectional studies (19,662 females) Thirty‐one pre‐post vaccine introduction studies (107,112,909 person‐years plus 16,116,268 females and males plus 13,026 cases of anogenital warts)	Thirteen cohort studies reported a reduced risk of anogenital warts in vaccinated compared with unvaccinated participants (RR 0.47, 95% CI 0.36 to 0.61). Two cohort studies reported no difference in risk of anogenital warts between vaccinated and unvaccinated participants. One cross‐sectional study reported a decreased odds of anogenital warts in vaccinated compared with unvaccinated participants. One cross‐sectional study reported no difference in odds, and one did not report any cases of anogenital warts in the exposed group. Twenty‐five pre‐post vaccine introduction studies reported a decrease in anogenital warts incidence following the introduction of HPV vaccine. Six studies reported no difference in anogenital warts incidence.	MODERATE^j^ ⊕⊕⊕◯ Downgraded due to methodological limitations	HPV vaccination probably reduces the incidence of anogenital warts.
**Serious adverse events**	No studies were identified that reported on this outcome.			

AIN: anal intraepithelial neoplasia (precancer of the perianal skin); AIS: adenocarcinoma in situ (precancer of the glandular cells of the cervix, also known as cervical intraepithelial glandular neoplasia (CGIN)); CI: confidence interval; CIN: cervical intraepithelial neoplasia (precancer of the squamous (skin‐like) cells of the cervix); CIN2: cervical intraepithelial neoplasia grade 2; CIN2+: cervical intraepithelial neoplasia grade 2 or higher; CIN3: cervical intraepithelial neoplasia grade 3; CIN3+: cervical intraepithelial neoplasia grade 3 or higher; HPV: human papillomavirus; PeIN: penile intraepithelial neoplasia (precancer of the penile skin); RCT: randomised controlled trial; RR: risk ratio; VaIN: vaginal intraepithelial neoplasia (precancer of the vaginal skin/mucosa); VIN: vulval intraepithelial neoplasia (precancer of the vulval skin) ^a^Three cohort studies were at moderate risk of bias, two were at serious risk and one at critical risk. The main concerns for bias were the potential for residual confounding and selective reporting. The other designs were at moderate, serious or critical risk of bias. Overall, we have downgraded one level for methodological limitations. ^b^All three studies were at critical risk of bias. The main concerns for bias were the potential for residual confounding and classification of the interventions. Overall, we have downgraded two levels for serious methodological limitations. ^c^Downgraded one level for inconsistency – studies show no effect, a possible harm and a possible benefit of HPV vaccination. ^d^Downgraded one level for imprecision – one cross‐sectional study with no cases, one pre‐post vaccine introduction study with an unclear number of cases. ^e^Eight cohort studies were at serious risk of bias and four at critical risk of bias. The other study designs were at moderate, serious or critical risk of bias. The main concerns for bias were the potential for residual confounding and selection bias. Overall, we have downgraded one level for methodological limitations. ^f^One RCT extension study was at serious risk of bias, four pre‐post vaccine introduction studies were at serious risk of bias. The main concerns for bias were the potential for residual confounding and classification of the interventions. Overall, we have downgraded one level for methodological limitations. ^g^Downgraded one level for inconsistency – studies show no effect, a possible harm and a possible benefit of HPV vaccination. ^h^Downgraded one level for imprecision – one study with no cases in the exposed group, two studies with an unclear number of events counted. ^i^One cohort study was at moderate risk of bias, seven cohort studies were at serious risk and six were at critical risk of bias. The other designs were at moderate, serious or critical risk of bias. Overall, we have downgraded one level for methodological limitations. ^j^One cohort study was at moderate risk of bias and 13 at serious risk of bias. The main concern for bias was the potential for residual confounding. The other designs were at serious or critical risk of bias. Overall, we have downgraded one level for methodological limitations.

**Summary of findings 2:** Summary of findings – specific adverse events

**Population:** general population of any age **Setting:** any setting **Intervention:** full or partial series HPV vaccination **Comparator:** no vaccination				
**Specific adverse events outcome**	**Number of studies (participants)**	**Summary of effects**	**Overall certainty of the evidence**	**Interpretation of findings**
**Postural orthostatic tachycardia syndrome (POTS)**	Two cohort studies (1,058,868 person‐years) One self‐controlled case series (1619 person‐years)	The cohort studies reported no association between HPV vaccination and POTS (RR 0.99, 95% CI 0.46 to 2.22). The self‐controlled case series reported no increased risk of POTS following HPV vaccination.	MODERATE^a^ ⊕⊕⊕◯ Downgraded due to methodological limitations	HPV vaccination likely does not increase the risk of POTS.
**Chronic fatigue syndrome/myalgic encephalomyelitis (CFS/ME)**	Four cohort studies (4,336,406 person‐years) Three self‐controlled case series (297 cases) Two pre‐post vaccine introduction studies (509,331 person‐years)	The cohort studies reported no association between HPV vaccination and CFS/ME (RR 0.96, 95% CI 0.67 to 1.39). Some studies found that HPV vaccination was associated with a lower likelihood of CFS/ME. The self‐controlled case series analyses reported no increased risk of CFS/ME following HPV vaccination (RR 0.74, 95% CI 0.40 to 1.39). The pre‐post vaccine introduction studies reported no increase in the incidence of CFS/ME following introduction of HPV vaccine.	MODERATE^b^ ⊕⊕⊕◯ Downgraded due to methodological limitations	HPV vaccination likely does not increase the risk of CFS/ME.
**Paralysis**	Five cohort studies (24,663,514 person‐years) One self‐controlled case series (33 cases)	The cohort studies reported no association between HPV vaccination and increased risk of paralysis (RR 0.62, 95% CI 0.36 to 1.07). Some studies found that HPV vaccination was associated with a lower likelihood of paralysis. The self‐controlled case series reported no increased risk of paralysis following HPV vaccination.	MODERATE ^c^ ⊕⊕⊕◯ Downgraded due to methodological limitations	HPV vaccination likely does not increase the risk of paralysis.
**Complex regional pain syndrome (CRPS)**	Three cohort studies (3,330,138 person‐years) One self‐controlled case series (535 cases)	The cohort studies reported no association between HPV vaccination and CRPS (RR 0.76, 95% CI 0.62 to 0.94). The self‐controlled case series reported no increased risk of CRPS following HPV vaccination.	MODERATE ^d^ ⊕⊕⊕◯ Downgraded due to methodological limitations	HPV vaccination likely does not increase the risk of CRPS.
**Guillain‐Barré syndrome**	Ten cohort studies (42,442,906 person‐years) One case‐control study (0 cases/143 females) Three self‐controlled case series (156 cases) One pre‐post vaccine introduction study (876,492 females and males)	Nine of the cohort studies reported no association between HPV vaccination and increased risk of Guillain‐Barré syndrome (RR 0.89, 95% CI 0.36 to 2.20). One reported an increase in incidence associated with HPV vaccination. Some studies found that HPV vaccination was associated with a lower likelihood of Guillain‐Barré syndrome. The case‐control study reported no cases of Guillain‐Barré syndrome. The self‐controlled case series analyses reported no increased risk of Guillain‐Barré syndrome following HPV vaccination (RR 1.53, 95% CI 0.78 to 2.98). The pre‐post vaccine introduction study reported no increase in the incidence of Guillain‐Barré syndrome following HPV vaccination.	LOW^e,f^ ⊕⊕◯◯ Downgraded due to methodological limitations and inconsistency	The evidence suggests that HPV vaccination does not increase the risk of Guillain‐Barré syndrome.
**Premature ovarian failure**	Three cohort studies (996,428 females plus 2,774,964 person‐years)	The cohort studies reported no association between HPV vaccination and premature ovarian failure.	MODERATE^g^ ⊕⊕⊕◯ Downgraded due to methodological limitations	HPV vaccination likely does not increase the risk of premature ovarian failure.
**Infertility**	One cohort study (3483 females, 1022 males) One cross‐sectional study (1114 females)	The cohort study reported no association between HPV vaccination and fecundability in females or males. The cross‐sectional study reported no association between HPV vaccination and infertility in females.	MODERATE^h^ ⊕⊕⊕◯ Downgraded due to methodological limitations	HPV vaccination likely does not increase the risk of infertility.
**Sexual activity**	Three cohort studies (1968 females) Two cross‐sectional studies (209,586 females) One pre‐post vaccine introduction study (260,493 females)	The cohort studies reported no association between HPV vaccination and sexual activity, measured as incidence of sexually transmitted infections. The cross‐sectional studies reported no association between HPV vaccination and sexual activity, measured as incidence of sexually transmitted infections. The pre‐post vaccine introduction study reported no association between HPV vaccination and sexual activity, measured as incidence of sexually transmitted infections.	MODERATE^i^ ⊕⊕⊕◯ Downgraded due to methodological limitations	HPV vaccination likely does not increase sexual activity and the incidence of sexually transmitted infections.

CFS/ME: chronic fatigue syndrome/myalgic encephalomyelitis; CI: confidence interval; CRPS: complex regional pain syndrome; HPV: human papillomavirus; POTS: postural orthostatic tachycardia syndrome; RR: risk ratio ^a^One cohort study was at moderate risk of bias, one at serious risk. The main concerns for bias were the potential for residual confounding and selective reporting. The self‐controlled case series was at low risk of bias. Overall, we have downgraded one level for methodological limitations. ^b^The cohort studies were at moderate or serious risk of bias. The main concern for bias was the potential for residual confounding. The self‐controlled case series were at low risk of bias. Overall, we have downgraded one level for methodological limitations. ^c^The cohort studies were at moderate or serious risk of bias. The main concern for bias was the potential for residual confounding. Overall, we have downgraded one level for methodological limitations. ^d^All three cohort studies were at serious risk of bias. The main concerns for bias were the potential for residual confounding and measurement of the outcome. The self‐controlled case series was at low risk of bias. Overall, we have downgraded one level for methodological limitations. ^e^The cohort studies were at serious or critical risk of bias. The main concern for bias was the potential for residual confounding. The self‐controlled case series were at low risk of bias. Overall, we have downgraded one level for methodological limitations. ^f^Downgraded one level for inconsistency – studies show no effect, a possible harm and a possible benefit of HPV vaccination. ^g^The cohort studies were at moderate to critical risk of bias. The main concerns for bias were the potential for residual confounding and selection bias. Overall, we have downgraded one level for methodological limitations. ^h^Both studies were at serious risk of bias. The main concerns for bias were the potential for residual confounding, classification of the interventions and missing data. Overall, we have downgraded one level for methodological limitations. ^i^The cohort studies were at serious or critical risk of bias. The main concern for bias was the potential for residual confounding. Overall, we have downgraded one level for methodological limitations.

## Background

### Description of the condition

Cervical cancer is the fourth most common cancer and the fourth leading cause of death from cancer amongst females worldwide, with an estimated 570,000 new cases and 311,000 deaths in 2018 (Bray 2018). Cervical cancer is a common cancer in young women and people with a uterine cervix, particularly in the 25 to 45 age group (Bray 2018). The risk of developing cervical cancer by age 65 years ranges from 0.8% in developed countries to 1.5% in developing countries, and more than 85% of all cervical cancer deaths occur in low‐ and middle‐income countries (LMIC) (Bray 2018). The large geographical variation in cervical cancer rates and survival correlates with the availability of primary and secondary prevention strategies, as well as the prevalence of high‐risk human papillomavirus (hrHPV) infection. However, even in the UK, with a world‐leading screening programme, cervical cancer in females aged 25 to 49 is the fourth highest cause of cancer death (Cancer Research UK 2024b). In England, 4.63 million women were invited for cervical screening in a year (2019 to 2020), in order to identify and treat those at higher risk of cervical cancer (NHS Digital 2020a). Of these, nearly 100,000 required further investigation with colposcopy (direct visualisation of the cervix with a microscope) to determine whether treatment was needed for cervical intra‐epithelial neoplasia (CIN) or, more rarely, cervical glandular intraepithelial neoplasia (CGIN ‐ also known as adenocarcinoma in situ (AIS)) precursor lesions to prevent cervical cancer (NHS Digital 2020b). This can cause anxiety and distress for many people. Furthermore, treatment for CIN, although relatively minor and straightforward in most cases, may put some people at higher risk of premature birth, thereby having long‐term knock‐on effects of preventative treatment (Kyrgiou 2017).

Human papillomavirus (HPV) is the most common viral infection of the reproductive tract (WHO 2017). Infection with hrHPV is necessary, but not sufficient to develop cervical cancer. The majority of people are exposed to hrHPV and, although most HPV infections resolve spontaneously (Insinga 2011), persistent infections can lead to precancerous lesions and cancer of the cervix, vagina, vulva, anus, penis, and head and neck. In 2012, HPV‐related cancers accounted for an estimated 4.5% of all cancers worldwide (De Martel 2017). Of these estimated 636,000 HPV‐related cancers, 530,000 were cervical cancer, 35,000 anal cancer, 8500 vulval cancer, 13,000 penile cancer and 37,000 head and neck cancers (De Martel 2017).

Anogenital warts are caused by non‐oncogenic HPV subtypes, with HPV 6 and 11 responsible for 90% (Hawkins 2013). Anogenital warts are highly transmissible and difficult to eradicate, with high recurrence rates. The cost of treatment of anogenital warts in England in 2008 was estimated to be GBP 16.8 million, contributing to 6.6 days of healthy life lost per episode (Desai 2011; Woodhall 2011), and USD 220 million in the USA in 2004 (Insinga 2005). A systematic review found that annual incidence rates of new and recurrent anogenital warts, from clinical studies, vary from 160 to 289 per 100,000 (Patel 2013). Incidence is higher in those with immunocompromise, including immunosuppression following organ transplantation and HIV infection, and in men who have sex with men (MSM), with 11.6% of MSM reporting anogenital warts in a UK‐based study (Sonnenberg 2019). Many studies included in the systematic review came from high‐income countries. However, in one study from Nigeria, the incidence of anogenital warts was 1% in HIV‐negative women, and 5% in HIV‐positive women, demonstrating a significant health burden, especially in LMICs, which can have a profound effect upon quality of life (Dareng 2019).

With the advent of immunisation and screening programmes in developed countries, the majority of invasive cervical cancers could be prevented (Cancer Research UK 2024a). In 2018, The World Health Organization (WHO) Director‐General made a global call for the elimination of cervical cancer (Adhanom‐Ghebreyesus 2018). However, in the absence of organised screening, many people present with symptoms and locally advanced cervical cancer at diagnosis (WHO 2018). Sadly, even in countries with well‐organised, freely available screening programmes, screening cannot prevent all cervical cancers and is not widely accessible globally. Cervical cancer therefore remains a significant disease. Furthermore, ~20% of HPV‐related cancers do not have effective screening methods.

The introduction of primary testing for hrHPV, compared to cervical cytology, improves the sensitivity of screening, albeit at the cost of increased referrals to colposcopy (Koliopoulos 2017). This leads to an increase in the rate of detection of CIN and is likely to reduce the rate of cervical cancer within a population over time. However, unless background rates of hrHPV and high‐grade CIN also fall, this will increase the treatment rates for CIN.

### Description of the intervention

HPV vaccines were first licenced in 2006, and by 2016, 55% of high (HIC) and upper‐middle‐income (UMIC) countries had introduced vaccination programmes, compared to just 14% of lower‐middle‐income (LMIC) and lower‐income (LIC) countries, where disease burden of cervical cancer is higher, according to World Bank figures (Gallagher 2018; LaMontagne 2017).

The uptake of HPV vaccination varies widely between countries: in 2017, coverage rates ranged from 8% to 98% across 82 countries (Brotherton 2018). WHO estimated only 13% global HPV vaccine coverage in 2020, a reduction from 15% in 2019, despite the vaccine being available since 2006 (WHO 2021a). Reasons for this variation include organisation of immunisation programmes, resistance from healthcare providers, adverse media coverage and concerns about safety (Gallagher 2018).

Four prophylactic HPV vaccines have been pre‐qualified by WHO (see Table). Each vaccine is directed against two or more high‐risk HPV genotypes. All four vaccines contain L1 proteins of HPV genotypes 16 and 18 (Qiao 2020; WHO 2017), because these cause about 70% of cervical cancer globally. In addition to the pre‐qualified vaccines, as of December 2021, there are two vaccines in stage 2 to 3 development, one bivalent vaccine manufactured by Walvax in China, and a quadrivalent vaccine manufactured by the Serum Institute of India (LaMontagne 2017).

**1:** Characteristics of WHO pre‐qualified prophylactic HPV vaccines

	**Cervarix**	**Gardasil**	**Gardasil 9**	**Cecolin**
**Manufacturer**	GlaxoSmithKline (GSK, Rixensart, Belgium)	Merck, Sharp & Dome (Merck & Co, Whitehouse Station, NJ, USA)	Merck, Sharp & Dome (Merck & Co, Whitehouse Station, NJ, USA)	Xiamen Innovax Biotech Co. Ltd. (Xiamen, Fujian province, China)
**Antigens**	Bivalent: L1 VLPs of HPV16 (20 μg) and HPV18 (20 μg)	Quadrivalent: L1 VLPs of HPV6 (20 μg), HPV11 (40 μg), HPV16 (40 μg) and HPV18 (20 mg)	Nonavalent: L1 VLPs of HPV6 (30 μg), HPV11 (40 μg), HPV16 (60 μg), HPV18 (40 μg), HPV31 (20 μg), HPV33 (20 μg), HPV45 (20 μg), HPV52 (20 μg) and HPV58 (20 μg)	Bivalent: L1 VLPs of HPV16 (40 μg) and HPV18 (20 μg)
**Vaccination schedule**	3 doses: at day 1, month 1 and month 6	3 doses: at day 1, month 2 and month 6	3 doses: at day 1, month 2 and month 6	2 doses: at day 1 and month 6
**Adjuvant**	AS04: 500 μg aluminium hydroxide, 50 μg 3‐deacylated monophosphoryl lipid A (MPL)	225 μg amorphous aluminium hydroxyl‐phosphate sulphate	500 μg amorphous aluminium hydroxyl‐phosphate sulphate	208 μg aluminium adjuvant
**Trade name**	Cervarix	Gardasil, Silgard	Gardasil‐9	Cecolin
**Produced by recombinant technology using**	Baculovirus in *Trichoplusia* in insect cells	*Saccharomyces cerevisae* (Baker’s yeast)	*Saccharomyces cerevisae* (Baker’s yeast)	*Escherichia coli*

HPV: human papillomavirus; VLP: virus‐like particles; WHO: World Health Organization

### How the intervention might work

HPV L1 coat proteins self‐assemble into virus‐like particles (VLP), empty virus particles (capsids), containing no virus DNA (Kirnbauer 1992), which cannot cause an active infection. They work as prophylactic vaccines, which means they prevent an initial infection by HPV, in turn preventing the development of intraepithelial lesions caused by HPV genotypes that are present in the vaccine (Stanley 2006). HPV vaccines are therefore less effective in those already exposed to HPV (Arbyn 2018), hence why they are offered to adolescents, aiming for immunity prior to onset of sexual activity.

The virus‐like particles in the vaccines produce very high levels of antibodies in blood samples. The International Agency for Research on Cancer regards persistent HPV infection with HPV types 16 and 18 as an accurate surrogate marker for the development of precancerous lesions of the cervix and anus (IARC 2014). Persistent infection with hrHPV is the main cause of cervical cancer (Bosch 2002; Jaisamrarn 2013; Munoz 1996), with a well‐recognised progression from persistent HPV infection to the development of cervical intraepithelial neoplasia (CIN), although the majority of infections are cleared spontaneously and do not cause persistent infection (Insinga 2011). However, left untreated, almost one in three of those with high‐grade CIN (CIN3) will go on to develop cancer over 8 to 15 years (Campbell 1989; McIndoe 1984). It was therefore assumed that prevention of precancerous lesions would also be shown to prevent cancer when sufficient follow‐up time has accrued in post‐licensure studies. Less is known about the prognostic value of persistent HPV infection in the development of vaginal, vulval and oropharyngeal cancers (IARC 2014).

### Why it is important to do this review

Prevention or early detection of cancer is a major priority within health care, especially within the UK where survival rates lag behind European counterparts, largely due to late detection (De Angelis 2014). In cervical cancer, we are fortunate as the main focus is on prevention, since, unlike many cancers, it can be prevented or detected at a pre‐invasive stage. HPV vaccination, especially in countries where screening programmes are currently unaffordable, has the potential to be transformative.

Although conventional Cochrane reviews of randomised controlled trials (RCTs) have demonstrated the effectiveness of HPV vaccination (Arbyn 2018; Bergman 2025), due to the relatively short time periods of the studies, effective screening and follow‐up of those in the studies, the outcome measures are surrogate endpoints, rather than cervical cancer outcomes. As HPV can cause a variety of cancers in both males and females, short‐term RCTs are unlikely to capture the population‐level benefits of HPV vaccination, especially in un‐ or under‐screened individuals and populations. Additionally, even very large RCTs are unlikely to be able to fully evaluate rare and very rare adverse events, of treatment or non‐treatment, including those later events, such as premature delivery of infants due to treatment of CIN, which could otherwise have been avoided (Kyrgiou 2017), and prevention of long‐term complications from cancer treatment, such as lymphoedema and late effects of radiotherapy. Furthermore, benefits of vaccination in a population may extend out to non‐vaccinated individuals, if vaccination levels are high enough, due to the development of herd immunity, by reducing the prevalence of an infection in a population. Larger, population‐level, non‐randomised studies (NRS) are therefore better able to inform of the absolute harms and benefits of HPV vaccination, beyond that of selected trial participants. Outcome data on long‐term effects of HPV vaccination are now becoming available and recent studies demonstrate improvement in both cervical cancer rates and preterm delivery rates in HPV vaccinated cohorts (Aldhous 2019; Falcaro 2021; Lei 2020). The full impact of HPV vaccination on cancer incidence will not be known for many years, since the natural history of vulval, penile and head and neck cancers, caused by hrHPV, is much longer.

Evaluating the longer‐term harms and benefits of HPV vaccination is extremely important, especially in the face of community concerns about these issues, which can fuel vaccine hesitancy (Karafillakis 2019; Wong 2020). Scares about adverse events can be catastrophic to a vaccination programme. For example, in Denmark and Ireland, community scares saw vaccination rates temporarily drop from over 80% to around 50% (Corcoran 2018; Suppli 2018). In Japan, a scare also resulted in a pause in government recommendation of vaccination (Ujiie 2022).

With the global reach of social media, dissemination of information regarding adverse effects of vaccination can be extremely pervasive. It is therefore extremely important to more fully evaluate these outcomes, to provide reliable data to young people, parents, clinicians, policymakers and others when they are making choices about vaccination.

A comprehensive examination of the rare risks, and a better understanding of the longer‐term benefits of HPV vaccination, such as effects on cancer rates, preterm birth rates and reduced complications due to falling need for treatment of CIN, require large data from population‐level studies. It is hoped that these data will better inform the public debate about the benefits and harms of HPV vaccination and allow better‐informed decision‐making.

This review will look at non‐randomised studies of the effects of introducing HPV vaccination at a population‐level on rates of HPV‐related disease and harms, not just in the individuals vaccinated, thereby more fully informing the harms and benefits of vaccination, which may not be apparent even in large RCT‐level datasets (Reeves 2022). We evaluate RCTs in a parallel Cochrane review (Bergman 2025). It is hoped that these reviews will better inform the public debate about the benefits and harms of HPV vaccination and allow better decision‐making at an individual level.

## Objectives

We aimed to assess population‐level effects of human papillomavirus (HPV) vaccination programmes on HPV‐related disease and harms from vaccination.

## Methods

### Criteria for considering studies for this review

#### Types of studies

We included studies that assessed the impact of HPV vaccination on the general population. This included population‐level studies comparing outcomes before and after introduction of HPV vaccine, such as pre‐ versus post‐vaccine introduction studies, interrupted time series studies and controlled before‐and‐after studies. We also included individual‐level, non‐randomised comparative studies such as cohort studies, case‐control studies and self‐controlled case series. This included follow‐up of cohorts that were originally included in randomised controlled trials (RCTs). We did not include non‐comparative studies, such as single‐arm cohorts, case series or case reports, nor modelling studies, or RCTs. We included studies that were self‐described as the above designs; however, the final decision on the design was made by the review author team. Working definitions for the different study designs are provided in Appendix 1.

RCTs were not included, as these are assessed in a companion review (Bergman 2025).

#### Types of participants

The target population for HPV vaccination is adolescents, although some countries also vaccinate adults. We have included studies on all ages receiving prophylactic HPV vaccination. Studies on the general population were included and, where possible, we stratified analyses by age at vaccination and sex. Studies with only a subset of eligible participants were included if the eligible participants made up > 75% of the total population.

#### Types of interventions

We investigated primary prophylactic administration of HPV vaccines pre‐qualified by WHO (WHO 2021b), including Cervarix (bivalent, GlaxoSmithKline), Gardasil (quadrivalent, Merck), Gardasil‐9 (nonavalent, Merck) or Cecolin (bivalent, Innovax) HPV vaccines (see Table). We included studies evaluating the effect of a full vaccine series (three doses) or partial vaccine series (one or two doses). We excluded studies assessing non‐prophylactic and secondary prevention (i.e. used to prevent recurrence in those treated for HPV‐related disease) uses of vaccines.

We included studies that compare vaccination with any of the HPV vaccines with no vaccination. We investigated partial vaccination schedules compared with no vaccination using subgroup analysis.

#### Types of outcome measures

Whilst we recognise the importance of serious adverse events (those causing death, disability or hospitalisation), we also realise the importance of those adverse events perceived by patients as most prevalent and those adverse events that may prevent uptake. Prior to this review, we therefore conducted surveillance of the social media platforms WebMD and X (formerly Twitter) for important specific adverse events (see Appendix 2). We identified reports of 276 adverse events on WebMD, which we analysed by frequency and added pertinent adverse events to our strategy. We also identified 9781 tweets on HPV and found that injury was the top mentioned adverse event (51%), followed by death (23%), similar adverse events to those in WebMD, and concern about the potential for HPV vaccination to promote sexual promiscuity.

Any measure of the outcomes below was considered eligible for inclusion. While the duration and completeness of follow‐up varies, we extracted all relevant outcomes and time points reported. We stratified all analyses by outcome time point since vaccination as immediate term (< 4 weeks), short term (< 1 year), medium term (1 to 5 years) and long term (> 5 years). The lists of outcomes below are not exhaustive of all relevant outcomes for HPV vaccination. We excluded studies that did not report on any of the outcomes on the list, such as antibody titres, seroconversion or other specific adverse events.

##### Primary outcomes

- Invasive cervical, vaginal, vulval, anal, penile, or head and neck cancer rates.
- In females, histologically confirmed high‐grade cervical (CIN2, CIN3 and adenocarcinoma in situ (AIS)), vaginal (VaIN), vulval (VIN) or anal intraepithelial neoplasia (AIN), irrespective of HPV genotype (precancers of the cervix, vagina, vulval and anal skin/surface layers).
- In males, histologically confirmed penile (PeIN) or anal (AIN) intraepithelial neoplasia of any grade irrespective of HPV genotype (precancers of the penile and anal skin).
- Specific adverse events: incidence of postural tachycardia syndrome (POTS); chronic fatigue syndrome/myalgic encephalomyelitis (CFS/ME); paralysis; complex regional pain syndrome (CRPS); premature ovarian failure (POF); Guillain‐Barré syndrome (GBS); infertility; indicators of sexual activity.

##### Secondary outcomes

- Participation rates in cervical screening.
- Treatment rates for CIN and other HPV‐related pre‐invasive disease.
- Anogenital warts.
- In females, miscarriage and pre‐term birth rates, and neonatal outcomes.
- All‐cause mortality.
- Serious adverse events (that are fatal, life‐threatening, result in hospitalisation, persistent or significant disability/incapacity, congenital anomaly/birth defect, or require intervention to prevent permanent impairment or damage) (FDA 2024).
- Incident infection with vaccine HPV genotypes (HPV 16 and HPV 18, jointly; HPV 6, HPV 11, HPV 16 and HPV 18, jointly; and HPV 31, HPV 33, HPV 45, HPV 52 and HPV 58, jointly).
- Persistent infection (persisting for at least six months or at least 12 months) with vaccine HPV genotypes (HPV 16 and HPV 18, jointly; HPV 6, HPV 11, HPV 16 and HPV 18, jointly; and HPV 31, HPV 33, HPV 45, HPV 52 and HPV 58, jointly).
- Prevalent infection with vaccine HPV genotypes (HPV 16 and HPV 18, jointly; HPV 6, HPV 11, HPV 16 and HPV 18, jointly; HPV 31, HPV 33, HPV 45, HPV 52 and HPV 58, jointly; and HPV 6, HPV 11, HPV 16, HPV 18, HPV 31, HPV 33, HPV 45, HPV 52 and HPV 58, jointly).

It should be noted that POTS, CFS/ME and CRPS are diagnoses of exclusion, and global population background rates are not well‐established. We therefore sought to ascertain rates of these and other specific diagnoses, rather than rely on a constellation of symptoms that might or might not be indicative of these rare syndromes.

### Search methods for identification of studies

We attempted to identify all relevant studies regardless of language or publication status (published, unpublished, in press and in progress).

#### Electronic searches

The Information Specialist at the Cochrane Gynaecological, Neuro‐oncology and Orphan Cancers group designed the search strategies and ran the searches in the core databases:

- the Cochrane Central Register of Controlled Trials (CENTRAL; 2022, Issue 1), in the Cochrane Library;
- MEDLINE Ovid (2000 to 5 January 2022);
- Embase Ovid (2000 to 5 January 2022).

Due to the timeline of HPV vaccine development, searches earlier than 2000 were not required. An update search was performed in the above databases on 11 September 2024.

We have presented the MEDLINE search strategy in Appendix 3, which reflects the key concepts of the review. We adapted the MEDLINE search strategy, as indicated, for the other databases (Appendix 4; Appendix 5).

We did not apply language restrictions to the electronic searches, and arranged for translations as needed. If relevant studies were only reported in abstract form, we contacted the study authors for additional information when necessary.

#### Searching other resources

We searched the following databases for related systematic reviews and ongoing studies, and checked the reference lists of those that were relevant, for additional studies:

- Epistemonikos: https://www.epistemonikos.org;
- HTA Database (Health Technology Assessments Database): www.york.ac.uk/crd/#HTA.

We handsearched abstract books of meetings of the International Gynaecological Cancer Society, the European Society of Gynaecological Oncology, International Papillomavirus Meetings, EUROGIN (EUropean Research Organisation on Genital Infection and Neoplasia) and the Society of Gynecologic Oncologists from 2010 to the latest edition, to identify ongoing and unpublished studies. Where necessary, we contacted the main investigators of relevant ongoing studies for further information.

Abstracts of the Society of Gynecologic Oncology (SGO) Annual Meetings on Women’s Cancer are published in *Gynecologic Oncology* and were accessed by our electronic searches.

We also searched vaccine manufacturer websites for any relevant non‐randomised studies (NRS) and checked the reference list from an index of HPV studies (Jørgensen 2020).

### Data collection and analysis

We uploaded the results of all searches to DistillerSR (DistillerSR 2021) to aid sifting and remote teamwork. We used Review Manager (RevMan) for review production (RevMan 2025), using standard Cochrane methods.

#### Selection of studies

Citations and abstracts were screened independently, in duplicate, by two systematic review team members or by the Cochrane Crowd and one of our systematic review team members. A third review author resolved any disagreements. Cochrane Crowd is Cochrane’s citizen science platform, hosting citation screening tasks. Evaluations of Crowd accuracy have shown very high levels of sensitivity (99%) and specificity (99%) for RCTs (Noel‐Storr 2021). We developed a learning module and agreement algorithm for the Crowd to screen for NRS. We obtained full‐text reports for all potentially eligible studies. Two independent review authors determined the eligibility of studies for inclusion in the review from the full reports according to predefined criteria. A third systematic review author resolved any disagreements.

We checked all studies for potential overlapping populations. We considered populations to be overlapping if two studies included people in the same region during overlapping time periods, and it was likely that their data were reported in both studies. In this case, we grouped these studies together under the same study name in the list of included studies and only included one study in the meta‐analysis if the studies reported on the same outcomes. This was the study with the most comprehensive coverage of the population.

#### Data extraction and management

Two review authors carried out data extraction independently using pretested data extraction forms. Study characteristics and outcome data were independently extracted, and we resolved any differences by discussion between the two review authors and referral to the study reports. Where there were two or more sources of data with conflicting information, we noted the conflict and attempted to contact the study authors for clarification. We had planned to contact study authors for missing data but did not identify any missing information.

#### Outcome data and confounders

We collected outcome definitions, source of outcome data and duration since vaccination for each outcome.

We collected the number of participants experiencing an outcome event and the number of participants analysed in each group. Where only rates were reported, we collected the event rate or the number of events and the person‐years in each intervention group. Where available, we extracted adjusted effect estimates with their respective measure of variance (standard error (SE), standard deviation (SD) or 95% confidence interval (95% CI)). We collected data on any confounding factors considered in the analysis and the methods used to control for confounding.

We preferentially extracted outcomes assessed by the most clinically valid measure and effect estimates adjusted for the most confounders.

We assessed whether there was targeted ascertainment of pre‐specified participant outcomes, or if the information had to be extracted from routine healthcare administrative or insurance databases.

#### Study characteristics

We recorded information on the following study characteristics.

- Methods: study design, study dates, duration of follow‐up, source of data.
- Setting: country and location, country income level (high‐ (HIC), upper‐middle‐ (UMIC), lower‐middle‐ (LMIC), or low‐income country (LIC) using World Bank classifications) (World Bank 2024).
- Population: sample size, sex, sexual orientation, age at vaccination, age at outcome collection, morbidities and socioeconomic status.
- Intervention: vaccine type, vaccination schedule (doses, interval), start date of vaccination programme, participation rates in vaccination HPV programme and co‐interventions (i.e. type (primary HPV versus cytological with or without HPV‐triage) and participation rates of cervical screening programme in the population).
- Notes: source of funding, conflicts of interest of study authors.

#### Assessment of risk of bias in included studies

We assessed the risk of bias of all included outcome effect estimates using different tools according to study design. For NRS of interventions, e.g. cohort, case‐control, cross‐sectional and pre‐post vaccine introduction studies, we used the ROBINS‐I tool for each outcome (Sterne 2016; Sterne 2021). In the ROBINS‐I tool, the following risks of bias are assessed: confounding, selection bias, bias in classification of interventions, bias due to deviations from intended interventions, bias due to missing data, bias in measurement of outcomes and bias in selection of the reported result. We considered the effect of assignment to the intervention as our effect of interest. For other study designs, such as self‐controlled case series, we used different methodological quality checklists based on the key sources of bias (Farrington 2004; Petersen 2016).

Two review authors independently assessed the risk of bias of each result included in the summary of findings tables. Any disagreements were resolved through discussion, and if consensus could not be reached, a third review author made the final assessment. Following assessment of all included studies, reliability and consistency of ratings across the studies was ensured through discussion among the review team. Any further disagreements were resolved through discussion within the review team.

As part of the risk of bias assessment, a preliminary specification of important confounders and co‐interventions was made using directed acyclic graphs (Suttorp 2015). These confounders and co‐interventions were derived from the adjustment and stratification variables used in analyses of known studies, variables mentioned or used in relevant systematic reviews (Drolet 2019; Markowitz 2018), and variables used in an ongoing living systematic review assessing risk of bias in observational studies on COVID vaccines (COVID NMA 2024).

We considered the most important confounding domains to be as follows.

**Time‐fixed confounders**

- Age
- Sex
- Socioeconomic status
- Ethnicity
- Geographic location
- Preventive health‐seeking behaviour

**Time‐varying confounders**

- Calendar time (to reflect changing incidence of virus and time since vaccine introduction)

We considered the most important co‐intervention to be the presence of a cervical cancer screening programme in the country in which the study was conducted.

The results of the risk of bias assessments are summarised and provide an evaluation of the overall methodological quality of the included studies. They also contributed to the GRADE ratings of the certainty of the evidence on an outcome basis.

#### Measures of treatment effect

Where data permitted, we combined adjusted point estimates using risk ratios (RR), odds ratios (OR), hazard ratios (HR) or relative incidence (RI) and their 95% CIs. We used the generic inverse variance method in RevMan Web (DerSimonian and Laird random‐effects).

If several adjusted estimates were reported within a study, we gave preference to the estimate that adjusts for the most important confounders that we pre‐specified for the review.

#### Unit of analysis issues

Unit of analysis issues were not expected. We analysed partial and full vaccination separately.

#### Dealing with missing data

We did not impute missing outcome data. Where missing data were substantial (> 5%), we assessed the risk of bias due to missing outcome data with the ROBINS‐I tool as moderate or serious risk (Sterne 2016).

#### Clinical and methodological heterogeneity

We did not pool data from different study designs. Analyses are stratified by study design, type of vaccine, age at vaccination and sex. If these characteristics were mixed or unknown within a study and could not be disaggregated, we analysed studies in a mixed group. Potential sources of heterogeneity are described, and the certainty of the evidence downgraded according to GRADE criteria, where appropriate.

#### Statistical heterogeneity

When pooling of studies was feasible (at least two studies included), we visually inspected forest plots for potential outlying studies and variability in the estimated effects across studies. We assessed statistical heterogeneity using the I^2^ statistic. This statistic quantifies the percentage of inconsistency in the treatment effects across studies beyond simple chance.

#### Assessment of reporting biases

For all included studies, we searched for published or online study protocols or statistical analysis plans. We recorded the presence or absence of these in the study characteristics tables and addressed this with the risk of bias tools. Where studies did not explicitly report on outcomes, we did not consider them at risk of selective reporting, unless there was evidence that they were planned and omitted from the report.

#### Data synthesis

The inclusion of various study designs in this review that use different estimation methods and statistical models means that we calculated different measures of effect and interpret these separately. We carried out quantitative and qualitative data syntheses separately for effectiveness and safety (harms).

We grouped studies for quantitative analysis according to study design (see Types of studies and Appendix 1) and outcome. Where possible, we stratified analyses by age at vaccination, sex, type of vaccine and outcome time point. We analysed all outcomes according to time from first vaccination, considering immediate term to be less than 4 weeks, short term to be less than 12 months, medium term from 12 months to 5 years, and long term for follow‐up longer than 5 years. If a study reported multiple time points within these categories, we prioritised the longest time point for meta‐analysis.

To account for confounding, if both adjusted and unadjusted estimates were reported within a study, we gave preference to the estimate that adjusted for the most important confounders for the review. Where data permitted, we combined adjusted point estimates using the generic inverse variance method (DerSimonian and Laird random‐effects). We also performed an analysis of adjusted effect estimates from those in the target population for vaccination, i.e. ≤ 16 years of age.

We checked all observational studies for potential overlapping populations, based on the location, study dates and source of the population and outcome data. Where we considered studies to be overlapping, these are grouped together in the list of included studies, and we only included one study in the meta‐analysis. This was the study with the lowest risk of bias, the largest sample size, or that covered the longest time period.

We used RR and its CI as measures of effect for cohort studies and population‐level studies. We used the OR and its CI for case‐control studies. For self‐controlled case series studies, we calculated a RI and its CI.

When meta‐analysis was not possible or appropriate, we used 'Synthesis without meta‐analysis' (SWiM) methodology (Campbell 2020).

#### Subgroup analysis and investigation of heterogeneity

We were unable to perform our planned subgroup analysis by time since vaccination programme introduction, as this was not clearly reported in most studies. We performed separate analyses for participants in the target population for vaccination, i.e. ≤ 16 years of age. We extracted effect estimates for partial schedule (i.e. one or two doses) and reported these along with full schedule effect estimates for each outcome.

#### Sensitivity analysis

To test the robustness of the data, we planned to carry out the following sensitivity analyses for the primary outcomes.

- We planned to exclude studies with overall critical or serious risk of bias from the analysis. We did not identify any studies that reported effect estimates adjusted for confounding that were considered at critical risk of bias. Most studies were at serious risk of bias, so where possible we have reported in the results which studies are at moderate or low risk of bias. Separate analyses for these studies were not necessary.
- We planned to perform meta‐analysis using the Hartung‐Knapp‐Sidik‐Jonkman method when combining unadjusted estimates (IntHout 2014). However, we now only analyse adjusted effect estimates using the generic inverse variance approach.
- If we had included any studies reported only as abstracts, we had planned to remove these from the analysis. However, we did not include any studies that were only reported as abstracts.

#### Summary of findings and assessment of the certainty of the evidence

We prepared summary of findings tables (Schünemann 2021) for HPV vaccination compared with no vaccination, stratified by study design. We assessed the certainty of evidence in the review through discussion between review authors using the GRADE approach with the GRADEpro online software (GRADEpro GDT) for the following outcomes:

- In females, invasive cervical, vaginal, vulval, anal, or head and neck cancer rates; histologically confirmed high‐grade cervical (CIN3 and adenocarcinoma in situ (AIS)), vaginal, vulva or anal intraepithelial neoplasia (AIN), irrespective of HPV genotype.
- In males, invasive anal, penile, or head and neck cancer rates; histologically confirmed penile (PeIN) or anal (AIN) intraepithelial neoplasia of any grade irrespective of HPV genotype.
- For all populations: anogenital warts, serious adverse events.

We created separate summary tables for specific adverse event outcomes, recording the number and type of studies evaluating each adverse event, the number of participants analysed and the estimates of effect comparing vaccination with no vaccination.

NRS started as high‐certainty evidence, and we considered the following factors for downgrading the certainty of the evidence: limitations in the study design (overall risk of bias); inconsistency of results (heterogeneity); indirectness of evidence (applicability); imprecision (few events and wide confidence intervals); and publication bias (Guyatt 2011). In addition, evidence could be upgraded if the pooled estimates revealed a large magnitude of effect or a dose‐response gradient was apparent (Schünemann 2019).

When the certainty of evidence was downgraded, we detailed the reasons in footnotes of the summary of findings tables and summarised these in the quality of the evidence section. Depending on whether evidence was downgraded or not, we rated the certainty of the evidence for each outcome as follows.

- High‐certainty evidence indicates that we are very confident that the true effect lies close to that of the estimate of the effect (evidence will not be downgraded).
- Moderate‐certainty evidence indicates that we are moderately confident in the effect estimate: the true effect is likely to be close to the estimate of the effect, but there is a possibility that it is substantially different (evidence will be downgraded one step for any of the factors described above).
- Low‐certainty evidence indicates that our confidence in the effect estimate is limited: the true effect may be substantially different from the estimate of the effect (evidence will be downgraded two steps for any of the factors described above).
- Very low‐certainty evidence indicates that we have very little confidence in the effect estimate: the true effect is likely to be substantially different from the estimate of effect (evidence will be downgraded three steps for any of the factors described above).

##### Stakeholder engagement

HPV vaccination is a major target for misinformation, especially targeting parents/carers via social media. We aimed to provide robust and unbiased evidence for patients, clinicians and policymakers, to enable fully informed decision‐making. This Cochrane HPV vaccine population‐level effect review is conducted in parallel with a Cochrane network meta‐analysis of randomised controlled trials (Bergman 2025). These reviews are both high priority for Cochrane and will inform the WHO and national government screening and immunisation strategies at national and global levels. We are aware that this will subject the review authors to significant scrutiny from communities with concerns about vaccination in general, and HPV vaccination specifically, but we are committed to promoting evidence‐based health care and improving outcomes for HPV‐related disease globally.

An Independent Advisory Group (IAG), including consumers, advised on review production and content.

## Results

### Description of studies

Overall, 225 non‐randomised studies from 347 records were included in this review (Figure). The characteristics of individual studies and assessment of risk of bias are presented in the Characteristics of included studies section.

**1:**
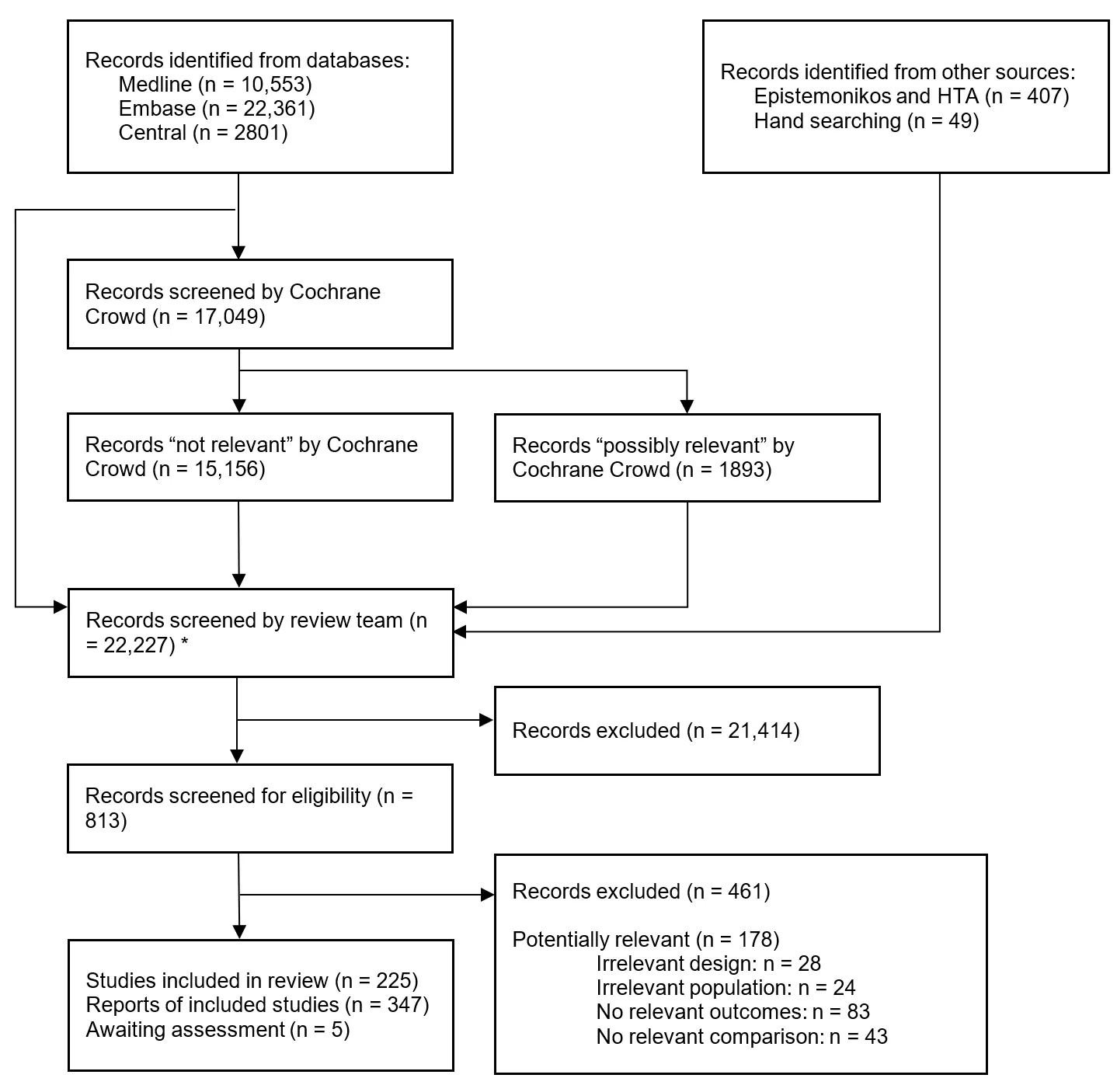
*includes update search n=4722 records (September 2024) not screened by Cochrane Crowd

#### Results of the search

The initial electronic database searches resulted in 17,049 de‐duplicated records. We retrieved 456 records from additional sources: 407 from the Epistemonikos and HTA databases and 49 records from handsearching. An update search was performed in the electronic databases on 11 September 2024, resulting in an additional 4722 records for screening.

The initial 17,049 records were screened by Cochrane Crowd. These records were categorised as “not relevant” (n = 15,156) or “possibly relevant” (n = 1893) by the Crowd. The review team then screened the abstracts of all 17,505 records from the database search and additional sources, plus 4722 records from the search update. We excluded 21,414 records and retrieved the full texts for the remaining 813 records. We excluded 461 full texts and included 347. Five records are included in the Characteristics of studies awaiting classification section.

See Figure for a flow diagram of the search and screening process.

#### Included studies

We included 86 cohort studies, four case‐control studies, 46 cross‐sectional studies, 69 pre‐post vaccine introduction studies, five RCT extensions and two self‐controlled case series. Thirteen additional studies reported on more than one type of analysis.

The included studies reported data from 46 countries. Most studies were carried out in the USA (49), the United Kingdom (21), Denmark (18), Australia (18), Canada (14), Japan (13), the Netherlands (eight), Sweden (seven), Italy (seven), Germany (six), Finland (six), Norway (five), France (five) and Spain (four). Two studies were carried out in Switzerland, Portugal, New Zealand, Mongolia, Thailand, Colombia, South Korea, Belgium and Brazil. There was one study each from Argentina, Armenia, Bhutan, Costa Rica, Czech Republic, Fiji, Greece, India, Israel, Luxembourg, Malaysia, Mexico, Paraguay, Russia, Rwanda, Taiwan and Uganda. The remaining eight studies reported data from more than one country, such as Denmark and Sweden (three), Denmark, Norway and Sweden (two), Denmark and Norway (one), Denmark, Iceland, Norway and Sweden (one), and Bhutan and Rwanda (one).

Of the included studies, 177 reported on only females, 10 only males and 37 a combination of males and females. One study reported on a sample of men who have sex with men and transgender females (Winer 2021‐USA).

Thirty‐two of the included studies reported on the effect of Cervarix vaccine, 131 reported on Gardasil, one reported on Gardasil‐9 and 47 reported on the effect of more than one of these vaccines. In 14 studies it was not clear which vaccine was being evaluated. We did not identify any studies reporting on the effectiveness of the Cecolin vaccine.

Many of the included studies reported on more than one outcome of interest. There were 20 studies reporting on cervical cancer, three studies on vaginal cancer, five studies on vulval cancer, three studies on anal cancer, two on penile cancer, and five studies on head and neck cancer. Three studies reported on adenocarcinoma in situ, 23 studies reported on CIN3+, 13 studies reported on CIN3, 37 studies reported on CIN2+, 11 studies reported on CIN2, two studies each reported on VIN and AIN, and one study reported on VaIN. No studies were identified that reported on population rates of PeIN.

For the specific adverse event outcomes, three studies reported on POTS; eight studies reported on CFS/ME; five studies reported on paralysis; four studies reported on CRPS; three studies reported on POF; 13 studies reported on GBS; two studies reported on infertility; and six studies reported on indicators of sexual activity.

Of the secondary outcomes, 10 studies reported on participation rates in cervical screening; five studies reported on treatment rates; 47 studies reported on anogenital warts; eight studies reported on pregnancy and neonatal outcomes; two studies reported on all‐cause mortality; seven studies reported on incident HPV infection; five on persistent HPV infection; and 80 on prevalent HPV infection. No studies reported on population rates of serious adverse events following HPV vaccination.

#### Excluded studies

We excluded 461 full texts. Of these, 178 were potentially relevant studies, and the reasons for their exclusion are included in the Characteristics of excluded studies table. We excluded 24 studies because they did not assess a relevant population. Most of the excluded studies (n = 83) contained no relevant outcomes or useable data for the review. We excluded 43 studies because they did not have a relevant comparison and 28 because of an irrelevant study design.

### Risk of bias in included studies

We assessed risk of bias for all primary and secondary outcomes using the ROBINS‐I tool (Sterne 2016) or a checklist for self‐controlled case series (SCCS) (Farrington 2004; Petersen 2016). Full details can be found in the additional tables.

#### Cancer and intraepithelial neoplasia outcomes

Of 20 studies reporting on cervical cancer, nine were at critical risk of bias overall because they failed to control for any potential confounding. Seven studies were at serious risk of bias overall and four were at moderate risk of bias overall. The risk of bias due to confounding was the highest risk domain across the 20 studies, with most other domains at low or moderate risk of bias.

The three studies reporting on adenocarcinoma in situ were all at critical risk of bias overall because they failed to control for any potential confounding. These studies were also at serious risk of selection bias or bias due to classification of the intervention.

Of 23 studies that reported on CIN3+, 10 were at critical risk of bias overall, 12 were at serious risk of bias overall and one study at moderate risk of bias. The bias due to confounding was again at highest risk, with other domains at low or moderate risk of bias.

The three studies that reported on vaginal cancer and five studies that reported on vulval cancer were at serious risk of bias overall due to confounding and bias in the classification of the intervention.

Three studies that reported on anal cancer and penile cancer were at serious risk of bias overall due to confounding and bias in the classification of the intervention.

Five studies reported on head and neck cancer, of which two were at critical risk of bias overall and three at serious risk of bias.

Thirteen studies reported on CIN3, of which 10 were at critical risk of bias due to confounding. Two were at serious risk of bias and one at moderate risk of bias.

Thirty‐seven studies reported on CIN2+, 22 of which were at critical risk of bias overall, 12 at serious risk of bias and three at moderate risk of bias.

Of the 11 studies that reported on CIN2, nine were at critical risk of bias overall and two at serious risk.

One study that reported on VaIN, VIN and AIN was at serious risk of bias overall, one study reporting on AIN was also at serious risk of bias and one study on VIN was at critical risk of bias.

#### Anogenital warts

Of 47 studies that reported on anogenital warts, 23 were at critical risk of bias overall due to a lack of control of confounding. Twenty‐three studies were at serious risk of bias overall. The domain bias due to confounding was at the highest risk in these studies, with bias in the classification of interventions also at serious risk in many pre‐post vaccine introduction studies. We considered one study at moderate risk of bias overall.

#### Specific adverse events

Of three studies that reported on POTS, one was at serious risk of bias and one at moderate risk of bias. Both studies controlled for some potential confounders, however the risk of residual confounding remained. One SCCS of POTS was at low risk of bias overall.

Of eight studies that reported on CFS/ME, one was at critical risk of bias, three were at serious risk of bias and two were at moderate risk of bias overall. Three SCCS of CFS/ME were all at low risk of bias overall.

Of five studies that reported on paralysis, four were at serious risk of bias and one at moderate risk of bias. One SCCS of paralysis was at low risk of bias overall.

Three studies reported on CRPS and all were at serious risk of bias overall due to confounding. One SCCS of CRPS was at low risk of bias overall.

Thirteen studies reported on GBS and of these five were at critical risk of bias overall because they did not control for any confounding. Seven studies were at serious risk of bias overall due to the potential for residual confounding. Of three SCCS on GBS, two were at low risk of bias overall and one at moderate risk.

One study on premature ovarian failure was considered at moderate risk of bias overall due to confounding and two studies at critical risk of bias.

Two studies reported on infertility and both were at serious risk of bias overall due to confounding and missing data.

Six studies reported on sexual activity and two were at critical risk of bias due to confounding. Four studies were at serious risk of bias overall due to confounding and bias due to classification of the intervention.

#### Pregnancy and neonatal outcomes

Eight studies reported on pregnancy and neonatal outcomes; one was at critical risk of bias and seven were at serious risk of bias due to confounding.

#### All‐cause mortality

Two studies reported on all‐cause mortality, one at critical risk of bias and the other at serious risk due to confounding.

#### Cervical screening attendance

Of the 10 studies that reported on cervical screening attendance, five were at critical risk of bias overall due to bias from confounding. Four studies were at serious risk of bias overall and one study was at moderate risk of bias.

#### Treatment rates

Of the five studies that reported on treatment rates for cervical disease, four were at critical risk of bias overall due to confounding. One study was considered at serious risk of bias overall.

#### Incident HPV infection

Of the seven studies that reported on incident HPV infection, five were considered at serious risk of bias overall and two were at moderate risk of bias overall.

#### Persistent HPV infection

Of the five studies that reported on persistent HPV infection, three were considered at serious risk of bias overall and two were at moderate risk of bias overall.

#### Prevalent HPV infection

Of 80 studies that reported on prevalent HPV infection, 31 were at critical risk of bias due to confounding, 46 studies were at serious risk of bias overall and three studies were at moderate risk of bias overall.

#### Allocation

Not applicable.

#### Blinding

Not applicable.

#### Incomplete outcome data

Not applicable.

#### Selective reporting

Not applicable.

#### Other potential sources of bias

Not applicable.

### Effects of interventions

See: **Summary of findings 1** Summary of findings – clinical outcomes; **Summary of findings 2** Summary of findings – specific adverse events

#### Primary outcomes

##### Invasive cervical cancer

See Table for effect estimates and Table for the risk of bias summary of included studies on cervical cancer. HPV vaccination probably reduces the incidence of cervical cancer (moderate‐certainty evidence; Table).

**2:** Primary clinical outcomes effect estimates: invasive cervical cancer

**Study**	**Vaccine**	**Population (sex, age at vaccination)**	**Sample size**	**Effect measure (time period)**	**Effect estimate**	**Adjustment factors**	**Notes**
Del Mistro 2021‐ITA	Gardasil (Merck quadrivalent)	Female, 15 to 25 years	Vaccinated: 4718 Unvaccinated: 91,512	Risk ratio (long‐term)	0.92 (0.05 to 15.76)	Unadjusted	Cohort; no events in exposed group
Falcaro 2021‐GBR	Gardasil (Merck quadrivalent)	Female, 12 to 13 years	214,800,000 person‐years; 27,946 cases of cervical cancer	Incidence rate ratio (long‐term)	0.13 (0.06 to 0.28)	Age, cohort, age‐by‐cohort interactions, linear trend (drift), dummy variables for the Jade Goody effect (publicity surrounding the last months and death of the celebrity Jade Goody from cervical cancer), seasonal effects, screening awareness campaign	Cohort
Falcaro 2021‐GBR	Gardasil (Merck quadrivalent)	Female, 14 to 16 years	214,800,000 person‐years; 27,946 cases of cervical cancer	Incidence rate ratio (long‐term)	0.38 (0.29 to 0.48)	Age, cohort, age‐by‐cohort interactions, linear trend (drift), dummy variables for the Jade Goody effect (publicity surrounding the last months and death of the celebrity Jade Goody from cervical cancer), seasonal effects, screening awareness campaign	Cohort
Falcaro 2021‐GBR	Gardasil (Merck quadrivalent)	Female, 16 to 18 years	214,800,000 person‐years; 27,946 cases of cervical cancer	Incidence rate ratio (long‐term)	0.66 (0.59 to 0.75)	Age, cohort, age‐by‐cohort interactions, linear trend (drift), dummy variables for the Jade Goody effect (publicity surrounding the last months and death of the celebrity Jade Goody from cervical cancer), seasonal effects, screening awareness campaign	Cohort
Kjaer 2021‐DNK	Cervarix (GSK bivalent); Gardasil (Merck quadrivalent); Gardasil 9 (Merck nonavalent)	Female, ≤ 16 years	Vaccinated: 502,522 Unvaccinated: 365,167	Incidence rate ratio (long‐term)	0.13 (0.04 to 0.40)	Age	Cohort
Kjaer 2021‐DNK	Cervarix (GSK bivalent); Gardasil (Merck quadrivalent); Gardasil 9 (Merck nonavalent)	Female, 17 to 19 years	Vaccinated: 502,522 Unvaccinated: 365,167	Incidence rate ratio (long‐term)	0.29 (0.08 to 1.01)	Age	Cohort
Kjaer 2021‐DNK	Cervarix (GSK bivalent); Gardasil (Merck quadrivalent); Gardasil 9 (Merck nonavalent)	Female, 20 to 30 years	Vaccinated: 502,522 Unvaccinated: 365,167	Incidence rate ratio (long‐term)	1.15 (0.88 to 1.50)	Age	Cohort
Lei 2020b‐SWE	Gardasil (Merck quadrivalent)	Female, 10 to 16 years	Vaccinated: 527,871 Unvaccinated: 1,145,112	Incidence rate ratio (long‐term)	0.12 (0.00 to 0.34)	Age, county of residence, calendar year, mother’s country of birth, parental education level, annual household income, previous diagnosis in mother of CIN3+ or cancers other than cervical cancer	Cohort
Lei 2020b‐SWE	Gardasil (Merck quadrivalent)	Female, 17 to 30 years	Vaccinated: 527,871 Unvaccinated: 1,145,112	Incidence rate ratio (long‐term)	0.47 (0.27 to 0.75)	Age, county of residence, calendar year, mother’s country of birth, parental education level, annual household income, previous diagnosis in mother of CIN3+ or cancers other than cervical cancer	Cohort
Palmer 2024‐GBR	Cervarix (GSK bivalent)	Female, 12 to 13 years at vaccination	Vaccinated: 29,144 Unvaccinated: 294,221	Vaccine effectiveness (long‐term)	100% (66.9% to 100%)	Scottish Index of Multiple Deprivation	Cohort; no events in exposed group
Palmer 2024‐GBR	Cervarix (GSK bivalent)	Female, ≥ 14 years at vaccination	Vaccinated: 109,838 Unvaccinated: 294,221	Vaccine effectiveness (long‐term)	73.8% (58.9% to 83.4%)	Scottish Index of Multiple Deprivation	Cohort
Ward 2024‐GBR	Cervarix (GSK bivalent); Gardasil (Merck quadrivalent)	Female, 17 to 18 years*	Vaccinated: 562,899 Unvaccinated: 882,613	Vaccine effectiveness (long‐term)	75.4% (11.4% to 94.6%)	Month of birth	Cohort, regression discontinuity analysis; *age at vaccination
Ikeda 2021‐JPN	Cervarix (GSK bivalent; Gardasil (Merck quadrivalent)	Female, 13 to 16 years	Cases: 8 Controls: 12,296	Odds ratio (medium‐term)	0.22 (0.01 to 3.79)	Unadjusted	Case‐control; no events in exposed group
Luostarinen 2018‐FIN	Cervarix (GSK bivalent); Gardasil (Merck quadrivalent)	Female, 14 to 17 years	N = 189,901 person‐years	Incidence rate ratio (long‐term)	0.11 (0.01 to 1.93)	Unadjusted	RCT extension; no events in exposed group
Rana 2013‐FIN	Gardasil (Merck quadrivalent)	Female, 16 to 17 years	Vaccinated: 3464 person‐years Unvaccinated: 62,878 person‐years	Incidence rate ratio (medium‐term)	0.15 (0.01 to 2.47)	Unadjusted	RCT extension; no events in exposed group
Sankaranarayanan 2018‐IND	Gardasil (Merck quadrivalent)	Female, 10 to 18 years	Vaccinated: 4348 Unvaccinated: 1574	Risk ratio (3 doses; long‐term)	0.25 (0.01 to 6.01)	Unadjusted	RCT extension; no events in exposed group
Sankaranarayanan 2018‐IND	Gardasil (Merck quadrivalent)	Female, 10 to 18 years	Vaccinated: 8431 Unvaccinated: 1574	Risk ratio (2 doses; long‐term)	0.23 (0.01 to 5.60)	Unadjusted	RCT extension; no events in exposed group
Sankaranarayanan 2018‐IND	Gardasil (Merck quadrivalent)	Female, 10 to 18 years	Vaccinated: 4950 Unvaccinated: 1574	Risk ratio (1 dose; long‐term)	0.17 (0.01 to 4.25)	Unadjusted	RCT extension; no events in exposed group
Dorton 2015‐USA	Gardasil (Merck quadrivalent)	Female, ≤ 26 years	Vaccinated: 481 Unvaccinated: 911	Risk ratio (long‐term)	1.94 (0.25 to 15.15)	Unadjusted	Cross‐sectional; no events in exposed group
Baldur‐Felskov 2015‐DNK	Gardasil (Merck quadrivalent)	Females, 12 to 99 years*	5567 cases of squamous cell carcinoma	Annual percentage change (2000‐2005)	‐0.1% (‐2.6% to 2.4%)	Age‐standardised	Pre‐ vs post‐vaccine introduction; *age at outcome
				Annual percentage change (2006‐2012)	‐0.6% (‐3.7% to 2.5%)		
				Annual percentage change (2013‐2019)	‐3.9% (‐7.5% to ‐0.2%)		
Baldur‐Felskov 2015‐DNK	Gardasil (Merck quadrivalent)	Females, 12 to 99 years*	1765 cases of adenocarcinoma	Annual percentage change (2000‐2005)	1.2% (‐0.4% to 2.8%)	Age‐standardised	Pre‐ vs post‐vaccine introduction; *age at outcome
				Annual percentage change (2006‐2012)	2.4% (‐2.0% to 7.0%)		
				Annual percentage change (2013‐2019)	0.5% (‐3.4% to 4.6%)		
Goodman 2024‐DEU	Cervarix (GSK bivalent); Gardasil (Merck quadrivalent); Gardasil 9 (Merck nonavalent)	Female, 28 to 33 years*	Pre‐vaccine: 22,533 Post‐vaccine: 38,987	Relative risk (long‐term; 2013‐2021)	0.30 (0.14 to 0.65)	Unadjusted	Pre‐ vs post‐vaccine introduction; *age at outcome
Grieger 2024‐DEU	Cervarix (GSK bivalent); Gardasil (Merck quadrivalent); Gardasil 9 (Merck nonavalent)	Female, 18 to 20 years*	265,365 cases	Annual percent change (2014‐2018)	‐2.6% (‐4.4% to ‐0.7%)	Unadjusted	Pre‐ vs post‐vaccine introduction; *age at outcome
Grieger 2024‐DEU	Cervarix (GSK bivalent); Gardasil (Merck quadrivalent); Gardasil 9 (Merck nonavalent)	Female, 21 to 23 years*	265,365 cases	Annual percent change (2004‐2010)	13.1 % (4.1% to 22.8%)	Unadjusted	Pre‐ vs post‐vaccine introduction; *age at outcome
				Annual percent change (2010‐2018)	‐7.8% (‐12.6% to ‐2.7%)		
Grieger 2024‐DEU	Cervarix (GSK bivalent); Gardasil (Merck quadrivalent); Gardasil 9 (Merck nonavalent)	Female, 24 to 26 years*	265,365 cases	Annual percent change (2004‐2013)	9.2% (6.9% to 11.5%)	Unadjusted	Pre‐ vs post‐vaccine introduction; *age at outcome
				Annual percent change (2013‐2018)	‐15.4% (‐19.6% to ‐11.1%)		
Grieger 2024‐DEU	Cervarix (GSK bivalent); Gardasil (Merck quadrivalent); Gardasil 9 (Merck nonavalent)	Female, 27 to 29 years*	265,365 cases	Annual percent change (2004‐2008)	18.2% (9.4% to 27.7%)	Unadjusted	Pre‐ vs post‐vaccine introduction; *age at outcome
				Annual percent change (2008‐2015)	5.0% (0.7% to 9.4%)		
				Annual percent change (2015‐2018)	‐15.1 (‐25.9% to ‐4.1%)		
Grieger 2024‐DEU	Cervarix (GSK bivalent); Gardasil (Merck quadrivalent); Gardasil 9 (Merck nonavalent)	Female, 30 to 32 years*	265,365 cases	Annual percent change (2013‐2018)	‐2.3% (‐7.7% to 3.4%)	Unadjusted	Pre‐ vs post‐vaccine introduction; *age at outcome
Grieger 2024‐DEU	Cervarix (GSK bivalent); Gardasil (Merck quadrivalent); Gardasil 9 (Merck nonavalent)	Female, 33 to 35 years*	265,365 cases	Annual percent change (2004‐2013)	7.3% (5.4% to 9.3%)	Unadjusted	Pre‐ vs post‐vaccine introduction; *age at outcome
				Annual percent change (2013‐2018)	‐1.0% (‐5.3% to 3.5%)		
Guo 2023‐USA	Gardasil (Merck quadrivalent)	Female, 15 to 24 years at outcome	1133 cases of cervical carcinoma	Incidence rate ratio (long‐term; 2002‐6 vs 2015‐19)	0.71 (0.64 to 0.80)	Age‐standardised	Pre‐ vs post‐vaccine introduction
Guo 2023‐USA	Gardasil (Merck quadrivalent)	Female, 25 to 34 years at outcome	16,979 cases of cervical carcinoma	Incidence rate ratio (long‐term; 2002‐6 vs 2015‐19)	0.91 (0.89 to 0.94)	Age‐standardised	Pre‐ vs post‐vaccine introduction
Jemal 2013‐USA	Gardasil (Merck quadrivalent)	Female, age NR	NR	Annual percent change (long‐term; 2000‐2009)	‐2.5%	Sex, age and delay	Pre‐ vs post‐vaccine introduction
Lopez 2018‐ESP	NR	Female, 11 to 14 years	NR	Incidence rate ratio (long‐term; 2003 vs 2014)	0.83 (0.81 to 0.84)	Unadjusted	Pre‐ vs post‐vaccine introduction
Onuki 2023‐JPN	NR	Female, 20 to 29 years	418,918 cases	Annual percentage change (1975‐2011)	5.9% (5.6% to 6.1%)	Unadjusted	Pre‐ vs post‐vaccine introduction
				Annual percentage change (2011‐2020)	‐13.5% (‐11.9% to ‐14.5%)		
Rebolj 2022‐GBR	Cervarix (GSK bivalent)	Female, 24 to 25 years	32 cases	Vaccine effectiveness (long‐term)	64% (‐91% to 93%)	Deprivation and laboratory	Pre‐ vs post‐vaccine introduction
Restivo 2023‐ITA	NR	Female, age NR	291,368 cases	Rate ratio (2008 vs 2018)	0.68 (0.62 to 0.74)	Unadjusted	Pre‐ vs post‐vaccine introduction

CIN3: cervical intraepithelial neoplasia grade 3; NR: not reported

**3:** Risk of bias summary: invasive cervical cancer

**Study**	**Confounding**	**Selection**	**Classification of interventions**	**Deviations from intended interventions**	**Missing data**	**Measurement of outcomes**	**Selection of reported result**	**Overall risk of bias**
Del Mistro 2021‐ITA	Critical	Low	Low	Low	Low	Low	Low	Critical
Falcaro 2021‐GBR	Moderate	Low	Low	Low	Low	Low	Low	Moderate
Kjaer 2021‐DNK	Serious	Moderate	Low	Low	Low	Low	Moderate	Serious
Lei 2020b‐SWE	Moderate	Low	Low	Low	Low	Low	Low	Moderate
Palmer 2024‐GBR	Serious	Moderate	Low	Low	Moderate	Low	Low	Serious
Ward 2024‐GBR	Moderate	Low	Moderate	Low	No information	Low	Low	Moderate
Ikeda 2021‐JPN	Critical	Moderate	Low	Low	Low	Low	Low	Critical
Luostarinen 2018‐FIN	Serious	Moderate	Moderate	Low	Low	Low	Low	Serious
Rana 2013‐FIN	Serious	Low	Low	Low	Low	Low	Low	Serious
Sankaranarayanan 2018‐IND	Moderate	Low	Low	Low	Moderate	Low	Low	Moderate
Dorton 2015‐USA	Critical	Serious	Moderate	Low	Moderate	Low	Moderate	Critical
Baldur‐Felskov 2015‐DNK	Critical	Low	Serious	Low	Low	Low	Low	Critical
Goodman 2024‐DEU	Critical	Low	Serious	Low	Low	Low	Low	Critical
Grieger 2024‐DEU	Critical	Low	Serious	Low	Low	Low	Low	Critical
Guo 2023‐USA	Serious	Low	Serious	Low	Low	Low	Low	Serious
Jemal 2013‐USA	Serious	Moderate	Serious	Low	Low	Low	Moderate	Serious
Lopez 2018‐ESP	Critical	Low	Serious	Low	Low	Low	Low	Critical
Onuki 2023‐JPN	Critical	Low	Serious	Low	Low	Low	Low	Critical
Restivo 2023‐ITA	Critical	Serious	Serious	Low	Low	Low	Low	Critical

Twenty studies were included that reported on cervical cancer following HPV vaccination (Baldur‐Felskov 2015‐DNK; Del Mistro 2021‐ITA; Dorton 2015‐USA; Falcaro 2021‐GBR; Goodman 2024‐DEU; Grieger 2024‐DEU; Guo 2023‐USA; Ikeda 2021‐JPN; Jemal 2013‐USA; Kjaer 2021‐DNK; Lei 2020b‐SWE; Lopez 2018‐ESP; Luostarinen 2018‐FIN; Onuki 2023‐JPN; Palmer 2024‐GBR; Rana 2013‐FIN; Rebolj 2022‐GBR; Restivo 2023‐ITA; Sankaranarayanan 2018‐IND; Ward 2024‐GBR).

Six were cohort studies (Del Mistro 2021‐ITA; Falcaro 2021‐GBR; Kjaer 2021‐DNK; Lei 2020b‐SWE; Palmer 2024‐GBR; Ward 2024‐GBR), one was a case‐control study (Ikeda 2021‐JPN), three were extensions of RCTs (Luostarinen 2018‐FIN; Rana 2013‐FIN; Sankaranarayanan 2018‐IND), one was a cross‐sectional study (Dorton 2015‐USA), and nine were pre‐post vaccine introduction studies (Baldur‐Felskov 2015‐DNK; Goodman 2024‐DEU; Grieger 2024‐DEU; Guo 2023‐USA; Jemal 2013‐USA; Lopez 2018‐ESP; Onuki 2023‐JPN; Rebolj 2022‐GBR; Restivo 2023‐ITA).

From the six cohort studies, one did not report any cases of cervical cancer in the exposed group (Del Mistro 2021‐ITA). A pooled estimate, from five cohort studies that adjusted for confounding, of the impact of HPV vaccination on rates of cervical cancer indicated a reduction of 63% in the long term (RR 0.37, 95% CI 0.25 to 0.56; 5 cohort studies, 4,390,243 females plus 27,946 cases of cervical cancer; I^2^ = 88%) (Analysis 1.1). The analysis showed high heterogeneity of effect estimates based on age at vaccination. An analysis restricted to those receiving an HPV vaccine at or before the age of 16 years showed a reduction of cervical cancer incidence of 80% (RR 0.20, 95% CI 0.09 to 0.44; 3 cohort studies, 4.54 million person‐years, 15 cases of cervical cancer; I^2^ = 69%) (Analysis 1.2).

There was one case‐control study, which did not identify any cases of cervical cancer in the exposed group (Ikeda 2021‐JPN). The study reported a reduced odds of cervical cancer following HPV vaccination (OR 0.22, 95% CI 0.01 to 3.79).

There were three RCT extension studies identified in which no cases of cervical cancer were reported in the exposed groups (Luostarinen 2018‐FIN; Rana 2013‐FIN; Sankaranarayanan 2018‐IND). All three studies reported a reduced incidence of cervical cancer following HPV vaccination, but with wide confidence intervals that incorporated no effect (Analysis 1.3).

One cross‐sectional study was also identified that did not report any cases of cervical cancer in the exposed group (Dorton 2015‐USA).

Nine pre‐post vaccine introduction studies were identified and all reported a reduction in cervical cancer incidence following HPV vaccine introduction (Baldur‐Felskov 2015‐DNK; Goodman 2024‐DEU; Grieger 2024‐DEU; Guo 2023‐USA; Jemal 2013‐USA; Lopez 2018‐ESP; Onuki 2023‐JPN; Rebolj 2022‐GBR; Restivo 2023‐ITA). These studies reported different effect estimates over different time periods, so data were not in a form that allowed for meta‐analysis.

One RCT extension study reported on the effectiveness of two doses and one dose of HPV vaccine, however in both instances no cases of cervical cancer were reported in the exposed groups (Sankaranarayanan 2018‐IND).

##### Adenocarcinoma in situ

See Table for effect estimates and Table for the risk of bias summary of included studies on adenocarcinoma in situ (AIS). We are unclear about the effect of HPV vaccination on AIS incidence because the certainty of the evidence is very low (very low‐certainty evidence; Table).

**4:** Primary clinical outcomes effect estimates: adenocarcinoma in situ

**Study**	**Vaccine**	**Population (sex, age at vaccination)**	**Sample size**	**Effect measure (time period)**	**Effect estimate**	**Adjustment factors**	**Notes**
Dorton 2015‐USA	Gardasil (Merck quadrivalent)	Female, ≤ 26 years	Vaccinated: 481 Unvaccinated: 911	Risk ratio (long‐term)	0.06 (0.00 to 1.02)	Unadjusted	Cross‐sectional; no events in exposed group
Baldur‐Felskov 2015‐DNK	Gardasil (Merck quadrivalent)	Females, 12 to 99 years*	5475 cases of adenocarcinoma in situ	Incidence rate ratio (long‐term; 2000 vs 2019)	1.09 (0.81 to 1.48)	Age‐standardised	Pre‐ vs post‐vaccine introduction; *age at outcome
Lopez 2018‐ESP	NR	Female, 11 to 14 years	NR	Incidence rate ratio (long‐term; 2003 vs 2014)	0.60 (0.58 to 0.62)	Unadjusted	Pre‐ vs post‐vaccine introduction

NR: not reported

**5:** Risk of bias summary: adenocarcinoma in situ

**Study**	**Confounding**	**Selection**	**Classification of interventions**	**Deviations from intended interventions**	**Missing data**	**Measurement of outcomes**	**Selection of reported result**	**Overall risk of bias**
Dorton 2015‐USA	Critical	Serious	Moderate	Low	Moderate	Low	Moderate	Critical
Baldur‐Felskov 2015‐DNK	Critical	Low	Serious	Low	Low	Low	Low	Critical
Lopez 2018‐ESP	Critical	Low	Serious	Low	Low	Low	Low	Critical

Three studies were included that reported on AIS following HPV vaccination (Baldur‐Felskov 2015‐DNK; Dorton 2015‐USA; Lopez 2018‐ESP).

One was a cross‐sectional study (Dorton 2015‐USA) and two were pre‐post vaccine introduction studies (Baldur‐Felskov 2015‐DNK; Lopez 2018‐ESP).

The cross‐sectional study reported no cases of AIS in the HPV vaccine group (Dorton 2015‐USA).

One pre‐post vaccine introduction study reported an increase in AIS incidence following HPV vaccine introduction (Baldur‐Felskov 2015‐DNK), while the other reported a reduction (Lopez 2018‐ESP).

##### Cervical intraepithelial neoplasia grade 3 and above (CIN3+)

See Table for effect estimates and Table for the risk of bias summary of included studies on CIN3+. HPV vaccination probably reduces the incidence of CIN3+ (moderate‐certainty evidence; Table).

**6:** Primary clinical outcomes effect estimates: CIN3+

**Study**	**Vaccine**	**Population (sex, age at vaccination)**	**Sample size**	**Effect measure (time period)**	**Effect estimate**	**Adjustment factors**	**Notes**
Brotherton 2019‐AUS	Gardasil (Merck quadrivalent)	Female, 12 to 15 years	Vaccinated: 174,995 Unvaccinated: 48,845	Hazard ratio (3 doses; medium‐term)	0.43 (0.35 to 0.53)	Age, area of residence, socioeconomic status	Cohort
Brotherton 2019‐AUS	Gardasil (Merck quadrivalent)	Female, 12 to 15 years	Vaccinated: 18,190 Unvaccinated: 48,845	Hazard ratio (2 doses; medium‐term)	0.42 (0.27 to 0.64)	Age, area of residence, socioeconomic status	Cohort
Brotherton 2019‐AUS	Gardasil (Merck quadrivalent)	Female, 12 to 15 years	Vaccinated: 8618 Unvaccinated: 48,845	Hazard ratio (1 dose; medium‐term)	0.66 (0.41 to 1.06)	Age, area of residence, socioeconomic status	Cohort
Castle 2019‐USA	Gardasil (Merck quadrivalent)	Female, < 18 years	Vaccinated: 15,290 Unvaccinated: 60,359	Risk ratio (medium‐term)	0.28 (0.08 to 0.81)	Unadjusted	Cohort
Castle 2019‐USA	Gardasil (Merck quadrivalent)	Female, 18 to 20 years	Vaccinated: 15,290 Unvaccinated: 60,359	Risk ratio (medium‐term)	0.85 (0.50 to 1.43)	Unadjusted	Cohort
Castle 2019‐USA	Gardasil (Merck quadrivalent)	Female, 21 to 24 years	Vaccinated: 15,290 Unvaccinated: 60,359	Risk ratio (medium‐term)	2.45 (1.73 to 3.48)	Unadjusted	Cohort
Del Mistro 2021‐ITA	Gardasil (Merck quadrivalent)	Female, 15 to 25 years	Vaccinated: 4718 Unvaccinated: 91,512	Risk ratio (long‐term)	1.11 (0.59 to 2.11)	Unadjusted	Cohort
Gargano 2021‐USA	Gardasil (Merck quadrivalent)	Female, 9 to 26 years	Vaccinated: 135,758 Unvaccinated: 559,789	Risk ratio (3 doses; long‐term)	0.34 (0.29 to 0.40)	Birth year, race	Cohort
Gargano 2021‐USA	Gardasil (Merck quadrivalent)	Female, < 20 years	Vaccinated: 171,156 Unvaccinated: 559,789	Risk ratio (long‐term)	0.35 (0.30 to 0.40)	Birth year, race	Cohort
Gargano 2021‐USA	Gardasil (Merck quadrivalent)	Female, ≥ 20 years	Vaccinated: 42,248 Unvaccinated: 559,789	Risk ratio (long‐term)	0.64 (0.55 to 0.75)	Birth year, race	Cohort
Gargano 2021‐USA	Gardasil (Merck quadrivalent)	Female, 9‐26 years	Vaccinated: 34,401 Unvaccinated: 559,789	Risk ratio (2 doses; long‐term)	0.67 (0.54 to 0.82)	Birth year, race	Cohort
Gargano 2021‐USA	Gardasil (Merck quadrivalent)	Female, 9 to 26 years	Vaccinated: 43,245 Unvaccinated: 559,789	Risk ratio (1 dose; long‐term)	0.60 (0.50 to 0.73)	Birth year, race	Cohort
Herweijer 2016‐SWE	Gardasil (Merck quadrivalent)	Female, 11 to 16 years	Vaccinated: 236,372 Unvaccinated: 1,097,319	Incidence rate ratio (long‐term)	0.16 (0.08 to 0.32)	Age, parental highest education	Cohort
Herweijer 2016‐SWE	Gardasil (Merck quadrivalent)	Female, 17 to 19 years	Vaccinated: 236,372 Unvaccinated: 1,097,319	Incidence rate ratio (long‐term)	0.43 (0.33 to 0.57)	Age, parental highest education	Cohort
Herweijer 2016‐SWE	Gardasil (Merck quadrivalent)	Female, 20 to 29 years	Vaccinated: 236,372 Unvaccinated: 1,097,319	Incidence rate ratio (long‐term)	0.75 (0.59 to 0.95)	Age, parental highest education	Cohort
Lehtinen 2017b‐FIN	Cervarix (GSK bivalent)	Female, 16 to 17 years	Vaccinated: 2472 Unvaccinated: 15,665	Incidence rate ratio (long‐term)	0.34 (0.12 to 0.92)	Unadjusted	Cohort
Lei 2020a‐SWE	Cervarix (GSK bivalent); Gardasil (Merck quadrivalent)	Female, 10 to 16 years	Vaccinated: 25,865 Unvaccinated: 100,400	Risk ratio (long‐term)	0.36 (0.31 to 0.42)	Birth cohort	Cohort
Lei 2020a‐SWE	Cervarix (GSK bivalent); Gardasil (Merck quadrivalent)	Female, 17 to 22 years	Vaccinated: 26,892 Unvaccinated: 100,400	Risk ratio (long‐term)	0.56 (0.50 to 0.64)	Birth cohort	Cohort
Orumaa 2024‐NOR	Gardasil (Merck quadrivalent)	Female, 16 to 30 years	Vaccinated: 441 cases Unvaccinated: 14,528 cases	Incidence rate ratio (long‐term)	0.37 (0.33 to 0.41)	Age, calendar year	Cohort
Orumaa 2024‐NOR	Gardasil (Merck quadrivalent)	Female, < 17 years at vaccination	Vaccinated: 135 cases Unvaccinated: 14,528 cases	Incidence rate ratio (long‐term)	0.15 (0.13 to 0.18)	Age, calendar year	Cohort
Palmer 2019‐GBR	Cervarix (GSK bivalent)	Female, 12 to 18+ years	NR	Odds ratio (3 doses; long‐term)	0.14 (0.08 to 0.25)	Deprivation, rurality	Cohort
Palmer 2019‐GBR	Cervarix (GSK bivalent)	Female, 12 to 18+ years	NR	Odds ratio (2 doses; long‐term)	0.77 (0.48 to 1.24)	Deprivation, rurality	Cohort
Palmer 2019‐GBR	Cervarix (GSK bivalent)	Female, 12 to 18+ years	NR	Odds ratio (1 dose; long‐term)	1.19 (0.70 to 2.05)	Deprivation, rurality	Cohort
Schurink‐Van't Klooster 2023‐NLD	Cervarix (GSK bivalent); Gardasil (Merck quadrivalent); Gardasil 9 (Merck nonavalent)	Female, 13 to 22 years	Vaccinated: 2233 Unvaccinated: 17,389	Odds ratio (2 doses; long‐term)	0.60 (0.33 to 1.08)	Age, age of vaccination, birth cohort	Cohort
Schurink‐Van't Klooster 2023‐NLD	Cervarix (GSK bivalent); Gardasil (Merck quadrivalent); Gardasil 9 (Merck nonavalent)	Female, 13 to 22 years	Vaccinated: 22,549 Unvaccinated: 17,389	Odds ratio (3 doses; long‐term)	0.28 (0.19 to 0.41)	Age, age of vaccination, birth cohort	Cohort
Verdoodt 2020‐DNK	Gardasil (Merck quadrivalent)	Female, < 16 years	Vaccinated: 215,309 Unvaccinated: 374,774	Incidence rate ratio (long‐term)	0.37 (0.30 to 0.45)	Attained age, socioeconomic position	Cohort
Yagi 2019‐JPN	Cervarix (GSK bivalent); Gardasil (Merck quadrivalent)	Female, 12 to 16 years	Vaccinated: 7389 Unvaccinated: 7872	Risk ratio (long‐term)	0.07 (0.00 to 1.24)	Unadjusted	Cohort; no events in exposed group
Gargano 2021‐USA	Gardasil (Merck quadrivalent)	Female, 9 to 26 years	Cases: 2746 Controls: 1247	Risk ratio (3 doses; long‐term)	0.28 (0.21 to 0.36)	Birth year, race	Case‐cohort analysis
Gargano 2021‐USA	Gardasil (Merck quadrivalent)	Female, < 20 years	Cases: 2775 Controls: 1295	Risk ratio (long‐term)	0.27 (0.22 to 0.35)	Birth year, race	Case‐cohort analysis
Gargano 2021‐USA	Gardasil (Merck quadrivalent)	Female, ≥ 20 years	Cases: 2756 Controls: 1074	Risk ratio (long‐term)	0.59 (0.44 to 0.79)	Birth year, race	Case‐cohort analysis
Gargano 2021‐USA	Gardasil (Merck quadrivalent)	Female, 9 to 26 years	Cases: 2704 Controls: 1053	Risk ratio (2 doses; long‐term)	0.61 (0.42 to 0.90)	Birth year, race	Case‐cohort analysis
Gargano 2021‐USA	Gardasil (Merck quadrivalent)	Female, 9 to 26 years	Cases: 2712 Controls: 1064	Risk ratio (1 dose; long‐term)	0.52 (0.37 to 0.75)	Birth year, race	Case‐cohort analysis
Ikeda 2021‐JPN	Cervarix (GSK bivalent); Gardasil (Merck quadrivalent)	Female, 13 to 16 years	Cases: 52 Controls: 12,296	Odds ratio (medium‐term)	0.19 (0.03 to 0.15)	Unadjusted	Case‐control
Silverberg 2018‐USA	Gardasil (Merck quadrivalent)	Female, 14 to 17 years	Cases: 1717 Controls: 8537	Incidence rate ratio (long‐term)	0.45 (0.27 to 0.76)	Matched by age, time since first cytology, years of health plan membership.	Case‐control
Silverberg 2018‐USA	Gardasil (Merck quadrivalent)	Female, 18 to 20 years	Cases: 1751 Controls: 8661	Incidence rate ratio (long‐term)	0.84 (0.59 to 1.21)	Matched by age, time since first cytology, years of health plan membership.	Case‐control
Silverberg 2018‐USA	Gardasil (Merck quadrivalent)	Female, ≥ 21 years	Cases: 1771 Controls: 8742	Incidence rate ratio (long‐term)	0.92 (0.59 to 1.17)	Matched by age, time since first cytology, years of health plan membership.	Case‐control
Silverberg 2018‐USA	Gardasil (Merck quadrivalent)	Female, 14 to 21+ years	Cases: 1766 Controls: 8835	Incidence rate ratio (3 doses; long‐term)	0.68 (0.52 to 0.90)	Matched by age, time since first cytology, years of health plan membership.	Case‐control
Silverberg 2018‐USA	Gardasil (Merck quadrivalent)	Female, 14 to 21+ years	Cases: 1742 Controls: 8517	Incidence rate ratio (2 doses; long‐term)	1.02 (0.71 to 1.48)	Matched by age, time since first cytology, years of health plan membership.	Case‐control
Silverberg 2018‐USA	Gardasil (Merck quadrivalent)	Female, 14 to 21+ years	Cases: 1849 Controls: 8588	Incidence rate ratio (1 dose; long‐term)	0.94 (0.68 to 1.30)	Matched by age, time since first cytology, years of health plan membership.	Case‐control
Kreimer 2011‐CRI	Cervarix (GSK bivalent)	Female, 18 to 25 years	Vaccinated: 1365 Unvaccinated: 1783	Incidence rate ratio (long‐term)	0.05 (0.01 to 0.26)	Age‐ and location‐matched	RCT extension
Hikari 2022‐JPN	Cervarix (GSK bivalent); Gardasil (Merck quadrivalent)	Female, 20 to 24 years	Vaccinated: 2467 Unvaccinated: 4786	Risk ratio (long‐term)	0.22 (0.01 to 4.00)	Unadjusted	Cross‐sectional; no events in exposed group
Ozawa 2017‐JPN	Cervarix (GSK bivalent); Gardasil (Merck quadrivalent)	Female, 12 to 16 years	Vaccinated: 1002 Unvaccinated: 4922	Risk ratio (long‐term)	0.14 (0.02 to 1.05)	Unadjusted	Cross‐sectional; no events in exposed group
Shiko 2020‐JPN	Cervarix (GSK bivalent)	Female, 12 to 16 years	Vaccinated: 3770 Unvaccinated: 30,511	Risk ratio (medium‐term)	0.09 (0.00 to 0.42)	Age, place of screening	Cross‐sectional; no events in exposed group
Tozawa‐Ono 2021‐JPN	Cervarix (GSK bivalent); Gardasil (Merck quadrivalent)	Female, 12 to 16 years	Vaccinated: 3102 Unvaccinated: 8611	Risk ratio (medium‐term)	0.59 (0.17 to 2.07)	Unadjusted	Cross‐sectional
Wright 2019‐USA	Gardasil (Merck quadrivalent)	Female, 11 to 26 years	Vaccinated: 2977 Unvaccinated: 11,176	Odds ratio (medium‐term)	1.00 (0.60 to 1.70)	Age	Cross‐sectional
Gargano 2023‐USA	Gardasil (Merck quadrivalent)	Female, 20 to 24 years	6021 cases total	Average annual percent change (2008 to 2016)	‐10.4% (‐13.1% to ‐7.5%)	Unadjusted	Pre‐ vs post‐vaccine introduction
Gargano 2023‐USA	Gardasil (Merck quadrivalent)	Female, 25 to 29 years	6021 cases total	Average annual percent change (2008 to 2016)	0.7% (‐2.1% to 3.7%)	Unadjusted	Pre‐ vs post‐vaccine introduction
Gargano 2023‐USA	Gardasil (Merck quadrivalent)	Female, 30 to 34 years	6021 cases total	Average annual percent change (2008 to 2016)	7.1% (3.8% to 10.6%)	Unadjusted	Pre‐ vs post‐vaccine introduction
Gargano 2023‐USA	Gardasil (Merck quadrivalent)	Female, 35 to 39 years	6021 cases total	Average annual percent change (2008 to 2016)	3.5% (‐2.1% to 9.3%)	Unadjusted	Pre‐ vs post‐vaccine introduction
Gargano 2023‐USA	Gardasil (Merck quadrivalent)	Female, 20 to 24 years	6021 cases total	Incidence rate (2008‐2009 vs 2015‐2016)	0.45 (0.32 to 0.60)	Unadjusted	Pre‐ vs post‐vaccine introduction
Gargano 2023‐USA	Gardasil (Merck quadrivalent)	Female, 25 to 29 years	6021 cases total	Incidence rate (2008‐2009 vs 2015‐2016)	1.01 (0.85 to 1.18)	Unadjusted	Pre‐ vs post‐vaccine introduction
Gargano 2023‐USA	Gardasil (Merck quadrivalent)	Female, 30 to 34 years	6021 cases total	Incidence rate (2008‐2009 vs 2015‐2016)	1.58 (1.32 to 1.88)	Unadjusted	Pre‐ vs post‐vaccine introduction
Gargano 2023‐USA	Gardasil (Merck quadrivalent)	Female, 35 to 39 years	6021 cases total	Incidence rate (2008‐2009 vs 2015‐2016)	1.48 (1.15 to 1.88)	Unadjusted	Pre‐ vs post‐vaccine introduction
Rebolj 2022‐GBR	Cervarix (GSK bivalent)	Female, 24 to 25 years	N = 64,274	Vaccine effectiveness (long‐term)	79% (73% to 83%)	Deprivation and laboratory	Pre‐ vs post‐vaccine introduction
Thamsborg 2020‐DNK	Gardasil (Merck quadrivalent)	Female, 15 years	Pre‐vaccine: 19,629 Post‐vaccine: 26,215	Incidence rate ratio (long‐term; 1999‐2008 vs 2009‐2018)	0.68 (0.58 to 0.79)	Unadjusted	Pre‐ vs post‐vaccine introduction

CIN3+: cervical intraepithelial neoplasia grade 3 or higher; NR: not reported; RCT: randomised controlled trial

**7:** Risk of bias summary: CIN3+

**Study**	**Confounding**	**Selection**	**Classification of interventions**	**Deviations from intended interventions**	**Missing data**	**Measurement of outcomes**	**Selection of reported result**	**Overall risk of bias**
Brotherton 2019‐AUS	Serious	Serious	Low	Low	Moderate	Low	Moderate	Serious
Castle 2019‐USA	Critical	Moderate	Low	Low	Low	Low	Low	Critical
Del Mistro 2021‐ITA	Critical	Low	Moderate	Low	Low	Low	Low	Critical
Gargano 2021‐USA	Serious	Low	Low	Low	Low	Low	Low	Serious
Herweijer 2016‐SWE	Serious	Low	Moderate	Low	Low	Low	Low	Serious
Lehtinen 2017b‐FIN	Critical	Moderate	Moderate	Low	Low	Low	Low	Critical
Lei 2020a‐SWE	Serious	Moderate	Low	Low	Low	Low	Low	Serious
Orumaa 2024‐NOR	Serious	Low	Low	Low	Low	Low	Low	Serious
Palmer 2019‐GBR	Serious	Moderate	Low	Low	Moderate	Low	Low	Serious
Schurink‐Van't Klooster 2023‐NLD	Serious	Low	Low	Low	Moderate	Low	Low	Serious
Verdoodt 2020‐DNK	Serious	Low	Low	Low	Low	Low	Low	Serious
Yagi 2019‐JPN	Critical	Low	Low	Low	Low	Low	Low	Critical
Ikeda 2021‐JPN	Critical	Moderate	Low	Low	Low	Low	Low	Critical
Silverberg 2018‐USA	Serious	Serious	Low	Low	Low	Low	Low	Serious
Kreimer 2011‐CRI	Moderate	Moderate	Moderate	Low	Moderate	Low	Low	Moderate
Hikari 2022‐JPN	Critical	Moderate	Moderate	Low	Moderate	Low	Low	Critical
Ozawa 2017‐JPN	Critical	Moderate	Moderate	Low	Low	Low	Low	Critical
Shiko 2020‐JPN	Serious	Moderate	Moderate	Low	Moderate	Low	Low	Serious
Tozawa‐Ono 2021‐JPN	Critical	Moderate	Moderate	Low	Moderate	Low	Low	Critical
Wright 2019‐USA	Serious	Moderate	Moderate	Low	Low	Low	Low	Serious
Gargano 2023‐USA	Critical	Moderate	Moderate	Low	Low	Low	Low	Critical
Rebolj 2022‐GBR	Serious	Moderate	Moderate	Low	Low	Low	Low	Serious
Thamsborg 2020‐DNK	Critical	Low	Serious	Low	Low	Low	Low	Critical

CIN3+: cervical intraepithelial neoplasia grade 3 or higher

Twenty‐three studies were included that reported on CIN3+ following HPV vaccination (Brotherton 2019‐AUS; Castle 2019‐USA; Del Mistro 2021‐ITA; Gargano 2021‐USA; Gargano 2023‐USA; Herweijer 2016‐SWE; Hikari 2022‐JPN; Ikeda 2021‐JPN; Kreimer 2011‐CRI; Lehtinen 2017b‐FIN; Lei 2020a‐SWE; Orumaa 2024‐NOR; Ozawa 2017‐JPN; Palmer 2019‐GBR; Rebolj 2022‐GBR; Schurink‐Van't Klooster 2023‐NLD; Shiko 2020‐JPN; Silverberg 2018‐USA; Thamsborg 2020‐DNK; Tozawa‐Ono 2021‐JPN; Verdoodt 2020‐DNK; Wright 2019‐USA; Yagi 2019‐JPN).

Eleven were cohort studies (Brotherton 2019‐AUS; Castle 2019‐USA; Del Mistro 2021‐ITA; Herweijer 2016‐SWE; Lehtinen 2017b‐FIN; Lei 2020a‐SWE; Orumaa 2024‐NOR; Palmer 2019‐GBR; Schurink‐Van't Klooster 2023‐NLD; Verdoodt 2020‐DNK; Yagi 2019‐JPN), two were case‐control studies (Ikeda 2021‐JPN; Silverberg 2018‐USA), one was an RCT extension study (Kreimer 2011‐CRI), five were cross‐sectional studies (Hikari 2022‐JPN; Ozawa 2017‐JPN; Shiko 2020‐JPN; Tozawa‐Ono 2021‐JPN; Wright 2019‐USA), and three were pre‐post vaccine introduction studies (Gargano 2023‐USA; Rebolj 2022‐GBR; Thamsborg 2020‐DNK). One study reported both a cohort analysis as well as a case‐cohort analysis (Gargano 2021‐USA).

From the cohort studies, four did not adjust for confounding, with one not reporting any cases of CIN3+ in the exposed group (Yagi 2019‐JPN). A pooled estimate from cohort studies, adjusted for confounding, of the impact of HPV vaccination on rates of CIN3+ indicated a reduction of 57% in the medium term (RR 0.43, 95% CI 0.35 to 0.53; 1 cohort study, 223,840 females) and 61% in the long term (RR 0.39, 95% CI 0.32 to 0.48; 7 cohort studies, > 3.4 million females; I^2^ = 91%) (Analysis 1.4). An analysis restricted to those receiving an HPV vaccine at or before the age of 16 years showed a reduction of CIN3+ incidence of 74% in the long term (RR 0.26, 95% CI 0.12 to 0.56; 2 cohort studies, 1.5 million females; I^2^ = 80%) (Analysis 1.5).

The two case‐control studies (Ikeda 2021‐JPN; Silverberg 2018‐USA) and the case‐cohort analysis (Gargano 2021‐USA) each reported a reduced odds of CIN3+ following HPV vaccination (Table).

The RCT extension study reported a reduced incidence of CIN3+ following HPV vaccination (incidence rate ratio (IRR) 0.05, 95% CI 0.01 to 0.26; 3148 females) (Kreimer 2011‐CRI).

Of the five cross‐sectional studies, four reported a reduction in CIN3+ following HPV vaccination (Hikari 2022‐JPN; Ozawa 2017‐JPN; Shiko 2020‐JPN; Tozawa‐Ono 2021‐JPN) and one reported no difference (Wright 2019‐USA) (Table).

The three pre‐post vaccine introduction studies reported a decreased incidence of CIN3+ when comparing time periods before and after HPV vaccine was introduced (Gargano 2023‐USA; Rebolj 2022‐GBR; Thamsborg 2020‐DNK) (Table).

Four studies reported on the effectiveness of two doses or one dose of HPV vaccine (Brotherton 2019‐AUS; Gargano 2021‐USA; Palmer 2019‐GBR; Silverberg 2018‐USA). Two of the three cohort studies reported a reduction of CIN3+ following two doses of HPV vaccine (Brotherton 2019‐AUS; Gargano 2021‐USA) and one cohort study reported a reduction of CIN3+ following one dose (Gargano 2021‐USA). One case‐control study reported no reduction of CIN3+ from one or two doses of HPV vaccine (Silverberg 2018‐USA).

##### Vaginal cancer

See Table for effect estimates and Table for the risk of bias summary of included studies on vaginal cancer. HPV vaccination may reduce vaginal cancer incidence (low‐certainty evidence; Table).

**8:** Primary clinical outcomes effect estimates: vaginal cancer

**Study**	**Vaccine**	**Population (sex, age at vaccination)**	**Sample size**	**Effect measure (time period)**	**Effect estimate**	**Adjustment factors**	**Notes**
Bertoli 2020‐DNK	Gardasil (Merck quadrivalent)	Female, 12 to 27 years	721 cases of vaginal squamous cell carcinoma	Incidence rate ratio (long‐term; 1978‐82 vs 2013‐17)	0.60 (0.09 to 3.08)	Age‐standardised	Pre‐ vs post‐vaccine introduction
Jemal 2013‐USA	Gardasil (Merck quadrivalent)	Female, age NR	NR	Annual percent change (long‐term; 2000 vs 2009)	White: ‐1.4% Black: ‐4.1% Asian/Pacific Islander: ‐2.1% American Indian/Alaska native: NR Hispanic: ‐0.6%	Age	Pre‐ vs post‐vaccine introduction; data only reported by ethnic groups
Guo 2023‐USA	Gardasil (Merck quadrivalent)	Female, 25 to 34 years at outcome	160 cases of vaginal squamous cell carcinoma	Rate ratio (long‐term; 2002‐6 vs 2015‐19)	0.65 (0.47 to 0.90)	Age‐standardised	Pre‐ vs post‐vaccine introduction

NR: not reported

**9:** Risk of bias summary: vaginal cancer

**Study**	**Confounding**	**Selection**	**Classification of interventions**	**Deviations from intended interventions**	**Missing data**	**Measurement of outcomes**	**Selection of reported result**	**Overall risk of bias**
Bertoli 2020‐DNK	Serious	Moderate	Serious	Low	Low	Low	Low	Serious
Jemal 2013‐USA	Serious	Moderate	Serious	Low	Low	Low	Moderate	Serious
Guo 2023‐USA	Serious	Moderate	Serious	Low	Low	Low	Moderate	Serious

**10:** Summary of findings – additional clinical outcomes

**Population:** general population of any age **Setting:** any setting **Intervention:** full or partial series HPV vaccination **Comparator:** no vaccination				
**Outcome**	**Number of studies (participants)**	**Summary of effect**	**Overall certainty of the evidence**	**Interpretation of findings**
**Invasive vaginal cancer**	Three pre‐post vaccine introduction studies (> 881 cases of vaginal cancer)	Three pre‐post vaccine introduction studies reported a reduction in vaginal cancer incidence between the pre‐ and post‐introduction periods.	LOW^a,b^ ⊕⊕◯◯ Downgraded due to methodological limitations and imprecision.	HPV vaccination may reduce vaginal cancer incidence.
**Invasive anal cancer**	Three pre‐post vaccine introduction studies (> 42,127 cases)	In females and males, two pre‐post vaccine introduction studies reported a decrease in anal cancer incidence between the pre‐ and post‐introduction periods and one study reported an increase.	VERY LOW^a,b,c^ ⊕◯◯◯ Downgraded due to methodological limitations, inconsistency and imprecision.	We do not know about the effect of HPV vaccine on anal cancer incidence because the certainty of the evidence is very low.
**Invasive penile cancer**	Two pre‐post vaccine introduction studies (> 15,804 cases)	Two pre‐post vaccine introduction studies reported a decrease in penile cancer incidence between the pre‐ and post‐introduction periods.	LOW^b,d^ ⊕⊕◯◯ Downgraded due to methodological limitations and imprecision.	HPV vaccination may reduce penile cancer incidence.
**Invasive head and neck cancer**	One cohort study (1,305,954 males and females) One RCT extension study (189,901 person‐years) Three pre‐post vaccine introduction studies (284,372 males and females plus 234,931 cases of oropharyngeal cancer)	In females and males, one cohort study reported a decreased risk of head and neck cancer following HPV vaccination. The RCT extension study did not identify any cases of head and neck cancer in vaccinated participants. Two pre‐post vaccine introduction studies reported a reduction in head and neck cancer incidence between the pre‐ and post‐introduction periods. One pre‐post vaccine introduction study reported inconsistent results, with some ethnic groups seeing an increased incidence and others a decrease.	LOW^b,e^ ⊕⊕◯◯ Downgraded due to methodological limitations and imprecision.	HPV vaccination may reduce head and neck cancer incidence.
**Cervical intraepithelial neoplasia grade 3 (CIN3)**	Three cohort studies (> 214,800,000 person‐years; 27,946 cases of cervical cancer) One case‐control study (12,340 females) One RCT extension (66,340 females) Three cross‐sectional studies (12,923 females) Five pre‐post vaccine introduction studies (234,775 females plus 73,576 cases of CIN3)	One cohort study reported a reduced risk of CIN3 following HPV vaccination (RR 0.17, 95% CI 0.06 to 0.45). Two other cohort studies reported no cases of CIN3 in the vaccinated participants. The case‐control study reported a reduced odds of CIN3 in vaccinated participants. The RCT extension study reported no cases of CIN3 in the vaccinated participants. Two cross‐sectional studies reported no difference in the risk of CIN3 in vaccinated and unvaccinated participants. One cross‐sectional study reported no cases of CIN3 in the vaccinated participants. Four pre‐post vaccine introduction studies reported a reduction in CIN3 incidence between the pre‐ and post‐introduction periods and one study reported an increased risk.	MODERATE^g^ ⊕⊕⊕◯ Downgraded due to methodological limitations	HPV vaccination probably reduces the incidence of CIN3.
**Cervical intraepithelial neoplasia grade 2 (CIN2)**	Four cohort studies (> 50,064 females) One case‐control study (12,461 females) Two cross‐sectional studies (12,074 females) Four pre‐post vaccine introduction studies (109,070 females plus 4296 cases of CIN2)	Three cohort studies reported a reduced risk of CIN2 following HPV vaccination. One other cohort study reported no difference in the risk of CIN2 between vaccinated and unvaccinated participants. One case‐control study reported reduced odds of CIN2 in vaccinated participants. Two cross‐sectional studies reported no difference in risk of CIN2 between vaccinated and unvaccinated participants. Three pre‐post vaccine introduction studies reported a reduction in CIN2 incidence between the pre‐ and post‐introduction periods and one study reported no difference.	MODERATE^h^ ⊕⊕⊕◯ Downgraded due to methodological limitations.	HPV vaccination probably reduces the incidence of CIN2.
**High‐grade vaginal intraepithelial neoplasia (VaIN)**	One pre‐post vaccine introduction study (945 cases of VaIN)	One pre‐post vaccine introduction study reported a reduction in VaIN incidence between the pre‐ and post‐introduction periods.	LOW^i,j^ ⊕⊕◯◯ Downgraded due to methodological limitations and imprecision.	HPV vaccination may reduce the incidence of VaIN.
**High‐grade vulval intraepithelial neoplasia (VIN)**	Two pre‐post vaccine introduction studies (6128 cases of VIN)	One pre‐post vaccine introduction study reported a reduction in VIN incidence between the pre‐ and post‐introduction periods and the other reported an increase in VIN incidence.	VERY LOW^c,j,k^ ⊕◯◯◯ Downgraded due to methodological limitations, inconsistency and imprecision.	We do not know about the effect of HPV vaccine on VIN incidence because the certainty of the evidence is very low.
**High‐grade anal intraepithelial neoplasia (AIN)**	One cohort study (30 cases of AIN) One pre‐post vaccine introduction study (2616 cases of AIN)	One cohort study reported a reduced risk of AIN following HPV vaccination. One pre‐post vaccine introduction study reported an increase in AIN incidence in males and females between the pre‐ and post‐introduction periods.	LOW^c,l^ ⊕⊕◯◯ Downgraded due to methodological limitations and inconsistency.	HPV vaccination may reduce the incidence of AIN.
**High‐grade penile intraepithelial neoplasia (PeIN)**	No studies were identified that reported on this outcome.			

AGW: anogenital warts; AIN: anal intraepithelial neoplasia (precancer of the perianal skin); AIS: adenocarcinoma in situ (precancer of the glandular cells of the cervix, also known as cervical intraepithelial glandular neoplasia (CGIN)); CI: confidence interval; CIN: cervical intraepithelial neoplasia (precancer of the squamous (skin‐like) cells of cervix); CIN3+: cervical intraepithelial neoplasia grade 3 or higher; CIN2: cervical intraepithelial neoplasia grade 2; CIN2+: cervical intraepithelial neoplasia grade 2 or higher; CIN3: cervical intraepithelial neoplasia grade 3; HPV: human papillomavirus; PeIN: penile intraepithelial neoplasia (precancer of the penile skin); RCT: randomised controlled trial; RR: risk ratio; VaIN: vaginal intraepithelial neoplasia (precancer of the vaginal skin/mucosa); VIN: vulval intraepithelial neoplasia (precancer of the vulval skin) ^a^All three pre‐post vaccine introduction studies were at serious risk of bias. The main concerns for bias were the potential for residual confounding and classification of the intervention. Overall, we have downgraded one level for methodological limitations. ^b^Downgraded one level for imprecision – one study with a confidence interval around the effect estimate that incorporates benefit, no effect and harm. One other study did not report the number of cases or an overall effect estimate. ^c^Downgraded one level for inconsistency – studies show no effect, a possible harm and a possible benefit of HPV vaccination. ^d^One pre‐post vaccine introduction study at serious risk of bias and one at critical risk of bias. The main concerns for bias were the potential for residual confounding and classification of the intervention. Overall, we have downgraded one level for methodological limitations. ^e^One cohort study at critical risk of bias, one RCT extension study at serious risk of bias, and three pre‐post vaccine introduction studies at serious or critical risk of bias. Overall, we have downgraded one level for methodological limitations. ^f^One cohort study at moderate risk of bias, two cohorts at critical risk. The other designs were at serious or critical risk of bias. Overall, we have downgraded one level for methodological limitations. ^h^Two cohort studies at serious risk of bias and two critical at risk. The other designs were at critical risk of bias. Overall, we have downgraded one level for methodological limitations. ^i^One pre‐post vaccine introduction study at serious risk of bias. Overall, we have downgraded one level for methodological limitations. ^j^Downgraded one level for imprecision – one study with confidence intervals around the effect estimates that incorporate benefit, no effect and harm. ^k^Two pre‐post vaccine introduction studies, one at serious risk of bias and one at critical risk. Overall, we have downgraded one level for methodological limitations. ^l^One cohort study at serious risk of bias and one pre‐post vaccine introduction study at serious risk of bias. Overall, we have downgraded one level for methodological limitations.

Three studies were included that reported on vaginal cancer following HPV vaccination (Bertoli 2020‐DNK; Guo 2023‐USA; Jemal 2013‐USA). All three were pre‐post vaccine introduction studies.

One study reported a decrease in vaginal cancer incidence from 1978‐1982 to 2013‐2017 but with confidence intervals that included no difference (Bertoli 2020‐DNK). The second study reported a decrease in vaginal cancer incidence from 2002‐2006 to 2015‐2019 (Guo 2023‐USA). The third study reported decreased incidence of vaginal cancer across all ethnic groups evaluated (Jemal 2013‐USA) (Table).

##### Vulval cancer

See Table for effect estimates and Table for the risk of bias summary of included studies on vulval cancer. We do not know about the effect of HPV vaccine on vulval cancer incidence because the certainty of the evidence is very low (very low‐certainty evidence; Table).

**11:** Primary clinical outcomes effect estimates: vulval cancer

**Study**	**Vaccine**	**Population (sex, age at vaccination)**	**Sample size**	**Effect measure (time period)**	**Effect estimate**	**Adjustment factors**	**Notes**
Luostarinen 2018‐FIN	Cervarix (GSK bivalent); Gardasil (Merck quadrivalent)	Female, 14 to 17 years	N = 189,901 person‐years	Incidence rate ratio (long‐term)	0.00 (0.00 to 73.81)	Unadjusted	RCT extension; no events in exposed group
Guo 2023‐USA	Gardasil (Merck quadrivalent)	Female, 15 to 24 years at outcome	374 cases of vulvar squamous cell carcinoma	Rate ratio (long‐term; 2002‐6 vs 2015‐19)	0.18 (0.13 to 0.24)	Age‐standardised	Pre‐ vs post‐vaccine introduction
Guo 2023‐USA	Gardasil (Merck quadrivalent)	Female, 25 to 34 years at outcome	1679 cases of vulvar squamous cell carcinoma	Rate ratio (long‐term; 2002‐6 vs 2015‐19)	0.54 (0.48 to 0.59)	Age‐standardised	Pre‐ vs post‐vaccine introduction
Jemal 2013‐USA	Gardasil (Merck quadrivalent)	Female, age NR	NR	Annual percent change (long‐term, 2000 vs 2009)	White: 1.4% Black: 0.9% Asian/Pacific Islander: ‐1.3% American Indian/Alaska native: NR Hispanic: ‐0.6%	Age	Pre‐ vs post‐vaccine introduction; data only reported by ethnic groups
Rasmussen 2020‐DNK	NR	Female, 12 to 26 years	NR	Annual percentage change (long‐term; 1997‐1998 vs 2017‐2018)	2.94% (2.25% to 3.63%)	Unadjusted	Pre‐ vs post‐vaccine introduction
Restivo 2023‐ITA	NR	Female, age NR	N = 34,510 cases	Rate ratio (2008 vs 2018)	0.87 (0.64 to 1.19)	Unadjusted	Pre‐ vs post‐vaccine introduction

NR: not reported

**12:** Risk of bias summary: vulval cancer

**Study**	**Confounding**	**Selection**	**Classification of interventions**	**Deviations from intended interventions**	**Missing data**	**Measurement of outcomes**	**Selection of reported result**	**Overall risk of bias**
Luostarinen 2018‐FIN	Serious	Moderate	Moderate	Low	Low	Low	Low	Serious
Guo 2023‐USA	Serious	Moderate	Serious	Low	Low	Low	Moderate	Serious
Jemal 2013‐USA	Serious	Moderate	Serious	Low	Low	Low	Moderate	Serious
Rasmussen 2020‐DNK	Serious	Moderate	Serious	Low	Low	Low	Low	Serious
Restivo 2023‐ITA	Serious	Serious	Serious	Low	Low	Low	Low	Serious

Five studies were included that reported on vulval cancer following HPV vaccination (Guo 2023‐USA; Jemal 2013‐USA; Luostarinen 2018‐FIN; Rasmussen 2020‐DNK; Restivo 2023‐ITA).

One study was an RCT extension study with no vulval cancer events reported in the HPV vaccine‐exposed group (Luostarinen 2018‐FIN). The other four were pre‐post vaccine introduction studies (Guo 2023‐USA; Jemal 2013‐USA; Rasmussen 2020‐DNK; Restivo 2023‐ITA). One study reported an increase in vulval cancer incidence (Rasmussen 2020‐DNK) and two studies reported a decrease when comparing time periods before and after HPV vaccine introduction (Guo 2023‐USA; Restivo 2023‐ITA). The other study reported inconsistent results, with some ethnic groups seeing an increased incidence and others a decrease (Jemal 2013‐USA) (Table).

##### Anal cancer

See Table for effect estimates and Table for the risk of bias summary of included studies on anal cancer. We do not know about the effect of HPV vaccine on anal cancer incidence because the certainty of the evidence is very low (very low‐certainty evidence; Table).

**13:** Primary clinical outcomes effect estimates: anal cancer

**Study**	**Vaccine**	**Population (sex, age at vaccination)**	**Sample size**	**Effect measure (time period)**	**Effect estimate**	**Adjustment factors**	**Notes**
Guo 2023‐USA	NR	Male and female, 20 to 44 years	N = 8062	Rate ratio (2001‐2008 vs 2009‐2018)	0.76 (0.7 to 0.83)	Age adjusted to US population	Pre‐ vs post‐vaccine introduction
Jemal 2013‐USA	Gardasil (Merck quadrivalent)	Female, age NR	NR	Annual percent change (long term; 2000 vs 2009)	White: 3.7% Black: 2.5% Asian/Pacific Islander: 1.6% American Indian/Alaska native: NR Hispanic: 0.7%	Age	Pre‐ vs post‐vaccine introduction; data only reported by ethnic groups
Jemal 2013‐USA	Gardasil (Merck quadrivalent)	Male, age NR	NR	Annual percent change (long term; 2000 vs 2009)	White: 2.6% Black: 5.6% Asian/Pacific Islander: 2.1% American Indian/Alaska native: NR Hispanic: 0.9%	Age	Pre‐ vs post‐vaccine introduction; data only reported by ethnic groups
Restivo 2023‐ITA	NR	Male, age NR	N = 42,127 cases	Rate ratio (2008 vs 2018)	0.83 (0.58 to 1.19)	Unadjusted	Pre‐ vs post‐vaccine introduction

NR: not reported

**14:** Risk of bias summary: anal cancer

**Study**	**Confounding**	**Selection**	**Classification of interventions**	**Deviations from intended interventions**	**Missing data**	**Measurement of outcomes**	**Selection of reported result**	**Overall risk of bias**
Guo 2023‐USA	Serious	Moderate	Serious	Low	Low	Low	Moderate	Serious
Jemal 2013‐USA	Serious	Moderate	Serious	Low	Low	Low	Moderate	Serious
Restivo 2023‐ITA	Serious	Serious	Serious	Low	Low	Low	Low	Serious

Three studies were included that reported on anal cancer following HPV vaccination (Guo 2023‐USA; Jemal 2013‐USA; Restivo 2023‐ITA). All three were pre‐post vaccine introduction studies.

One study reported an increased incidence of anal cancer in both males and females between 2000 and 2009 (Jemal 2013‐USA), while the other two studies reported a decrease (Guo 2023‐USA; Restivo 2023‐ITA) (Table).

##### Penile cancer

See Table for effect estimates and Table for the risk of bias summary of included studies on penile cancer. HPV vaccination may reduce penile cancer incidence (low‐certainty evidence; Table).

**15:** Primary clinical outcomes effect estimates: penile cancer

**Study**	**Vaccine**	**Population (sex, age at vaccination)**	**Sample size**	**Effect measure (time period)**	**Effect estimate**	**Adjustment factors**	**Notes**
Jemal 2013‐USA	Gardasil (Merck quadrivalent)	Male, age NR	NR	Annual percent change (long‐term; 2000 vs 2009)	White: ‐0.7% Black: ‐1.1% Asian/Pacific Islander: 0.5% American Indian/Alaska native: NR Hispanic: ‐0.4%	Age	Pre‐ vs post‐vaccine introduction; data only reported by ethnic groups
Restivo 2023‐ITA	NR	Male, age NR	N = 15,804 cases	Rate ratio (2008 vs 2018)	0.96 (0.54 to 1.71)	Unadjusted	Pre‐ vs post‐vaccine introduction

NR: not reported

**16:** Risk of bias summary: penile cancer

**Study**	**Confounding**	**Selection**	**Classification of interventions**	**Deviations from intended interventions**	**Missing data**	**Measurement of outcomes**	**Selection of reported result**	**Overall risk of bias**
Jemal 2013‐USA	Serious	Moderate	Serious	Low	Low	Low	Moderate	Serious
Restivo 2023‐ITA	Critical	Serious	Serious	Low	Low	Low	Low	Critical

Two studies were included that reported on penile cancer following HPV vaccination (Jemal 2013‐USA; Restivo 2023‐ITA). Both were pre‐post vaccine introduction studies and reported decreased incidence of penile cancer in males (Table).

##### Head and neck cancer

See Table for effect estimates and Table for the risk of bias summary of included studies on head and neck cancer. HPV vaccination may reduce head and neck cancer incidence (low‐certainty evidence; Table).

**17:** Primary clinical outcomes effect estimates: head and neck cancer

**Study**	**Vaccine**	**Population (sex, age at vaccination)**	**Sample size**	**Effect measure (time period)**	**Effect estimate**	**Adjustment factors**	**Notes**
Katz 2021‐USA	Cervarix (GSK bivalent); Gardasil (Merck quadrivalent); Gardasil 9 (Merck nonavalent)	Female, 45 to 64 years*	Vaccinated: 14,078 Unvaccinated: 687,567	Risk ratio (long‐term)	0.11 (0.03 to 0.33)	Unadjusted	Cohort; *age at outcome
Katz 2021‐USA	Cervarix (GSK bivalent); Gardasil (Merck quadrivalent); Gardasil 9 (Merck nonavalent)	Male, 45 to 64 years*	Vaccinated: 4720 Unvaccinated: 599,589	Risk ratio (long‐term)	0.04 (0.01 to 0.30)	Unadjusted	Cohort; *age at outcome
Luostarinen 2018‐FIN	Cervarix (GSK bivalent); Gardasil (Merck quadrivalent)	Female, 14 to 17 years	N = 189,901 person‐years	Incidence rate ratio (long‐term)	0.00 (0.00 to 73.81)	Unadjusted	RCT extension; no events in exposed group
Guo 2023‐USA	NR	Female 25 to 34 years	279 cases of oropharyngeal squamous cell carcinoma	Rate ratio (2002‐2006 vs 2015‐2019)	0.87 (0.68 to 1.11)	Age‐adjusted to US population	Pre‐ vs post‐vaccine introduction
Jemal 2013‐USA	Gardasil (Merck quadrivalent)	Female, age NR	NR	Annual percent change (long‐term; 2000 vs 2009)	White: 1.7% Black: ‐0.3% Asian/Pacific Islander: ‐2.5% American Indian/Alaska native: NR Hispanic: 0.2%	Age	Pre‐ vs post‐vaccine introduction; data only reported by ethnic groups
Jemal 2013‐USA	Gardasil (Merck quadrivalent)	Male, age NR	NR	Annual percent change (long‐term; 2000 vs 2009)	White: 3.9% Black: ‐1.6% Asian/Pacific Islander: 1.0% American Indian/Alaska native: 4.9% Hispanic: 0.8%	Age	Pre‐ vs post‐vaccine introduction; data only reported by ethnic groups
Jemal 2013‐USA	Gardasil (Merck quadrivalent)	Female, age NR	N = 55,108	Rate ratio (2014‐2018 vs 2002‐2006)	0.89 (0.84 to 0.93)	Age‐adjusted to the 2000 US standard population	Pre‐ vs post‐vaccine introduction
Jemal 2013‐USA	Gardasil (Merck quadrivalent)	Male, age NR	N = 229,264	Rate ratio (2014‐2018 vs 2002‐2006)	0.86 (0.78 to 0.95)	Age‐adjusted to the 2000 US standard population	Pre‐ vs post‐vaccine introduction
Restivo 2023‐ITA	NR	Female and male, age NR	N = 234,652 cases (oropharyngeal cancer)	Rate ratio (2008 vs 2018)	0.69 (0.52 to 0.92)	Unadjusted	Pre‐ vs post‐vaccine introduction

NR: not reported; RCT: randomised controlled trial

**18:** Risk of bias summary: head and neck cancer

**Study**	**Confounding**	**Selection**	**Classification of interventions**	**Deviations from intended interventions**	**Missing data**	**Measurement of outcomes**	**Selection of reported result**	**Overall risk of bias**
Katz 2021‐USA	Critical	Serious	Low	Low	Serious	Low	Serious	Critical
Luostarinen 2018‐FIN	Serious	Moderate	Moderate	Low	Low	Low	Low	Serious
Guo 2023‐USA	Serious	Moderate	Serious	Low	Low	Low	Moderate	Serious
Jemal 2013‐USA	Serious	Moderate	Serious	Low	Low	Low	Moderate	Serious
Restivo 2023‐ITA	Critical	Serious	Serious	Low	Low	Low	Low	Critical

Five studies were included that reported on head and neck cancer following HPV vaccination (Guo 2023‐USA; Jemal 2013‐USA; Katz 2021‐USA; Luostarinen 2018‐FIN; Restivo 2023‐ITA).

One was a cohort study and reported a reduced risk of head and neck cancer in both females (RR 0.11, 95% CI 0.03 to 0.33) and males (RR 0.04, 95% CI 0.01 to 0.30) (Katz 2021‐USA).

One study was an RCT extension study with no head and neck cancer events reported in the HPV vaccine‐exposed group (Luostarinen 2018‐FIN).

Three studies were pre‐post vaccine introduction studies (Guo 2023‐USA; Jemal 2013‐USA; Restivo 2023‐ITA), two of which reported decreased incidence of head and neck cancer in males and females (Guo 2023‐USA; Restivo 2023‐ITA) (Table). One study reported inconsistent results, with some ethnic groups seeing an increased incidence and others a decrease (Jemal 2013‐USA).

##### Cervical intraepithelial neoplasia grade 3 (CIN3)

See Table for effect estimates and Table for the risk of bias summary of included studies on CIN3. HPV vaccination probably reduces the incidence of CIN3 (moderate‐certainty evidence; Table).

**19:** Primary clinical outcomes effect estimates: CIN3

**Study**	**Vaccine**	**Population (sex, age at vaccination)**	**Sample size**	**Effect measure (time period)**	**Effect estimate**	**Adjustment factors**	**Notes**
Falcaro 2021‐GBR	Gardasil (Merck quadrivalent)	Female 12 to 13 years	214,800,000 person‐years; 27,946 cases of cervical cancer	Incidence rate ratio (long‐term)	0.03 (0.02 to 0.04)	Age, cohort, age‐by‐cohort interactions, linear trend (drift), dummy variables for the Jade Goody effect (publicity surrounding the last months and death of the celebrity Jade Goody from cervical cancer), seasonal effects, screening awareness campaign	Cohort
Falcaro 2021‐GBR	Gardasil (Merck quadrivalent)	Female 14 to 16 years	214,800,000 person‐years; 27,946 cases of cervical cancer	Incidence rate ratio (long‐term)	0.25 (0.23 to 0.28)	Age, cohort, age‐by‐cohort interactions, linear trend (drift), dummy variables for the Jade Goody effect (publicity surrounding the last months and death of the celebrity Jade Goody from cervical cancer), seasonal effects, screening awareness campaign	Cohort
Falcaro 2021‐GBR	Gardasil (Merck quadrivalent)	Female 16 to 18 years	214,800,000 person‐years; 27,946 cases of cervical cancer	Incidence rate ratio (long‐term)	0.61 (0.59 to 0.64)	Age, cohort, age‐by‐cohort interactions, linear trend (drift), dummy variables for the Jade Goody effect (publicity surrounding the last months and death of the celebrity Jade Goody from cervical cancer), seasonal effects, screening awareness campaign	Cohort
Paraskevaidis 2020‐GRC	NR	Female, NR	Vaccinated: 849 Unvaccinated: 849	Risk ratio (long‐term)	0.01 (0.00 to 0.23)	Unadjusted	Cohort; no events in exposed group
Yagi 2019‐JPN	Cervarix (GSK bivalent); Gardasil (Merck quadrivalent)	Female, 12 to 16 years	Vaccinated: 7389 Unvaccinated: 7872	Risk ratio (long‐term)	0.07 (0.00 to 1.24)	Unadjusted	Cohort; no events in exposed group
Ikeda 2021‐JPN	Cervarix (GSK bivalent; Gardasil (Merck quadrivalent)	Female, 13 to 16 years	Cases: 44 Controls: 12,296	Odds ratio (medium‐term)	0.27 (0.08 to 0.89)	Unadjusted	Case‐control
Rana 2013‐FIN	Gardasil (Merck quadrivalent)	Female, 16 to 17 years	Vaccinated: 3464 Unvaccinated: 62,876	Risk ratio (long‐term)	0.15 (0.01 to 2.47)	Unadjusted	RCT extension: no events in exposed group
Hiramatsu 2021‐JPN	Cervarix (GSK bivalent; Gardasil (Merck quadrivalent)	Female, 12 to 18 years	Vaccinated: 170 Unvaccinated: 877	Risk ratio (medium‐term)	5.13 (0.10 to 257.90)	Unadjusted	Cross‐sectional; no events in exposed or unexposed groups
Munro 2017‐GBR	Cervarix (GSK bivalent; Gardasil (Merck quadrivalent)	Female, 20 to 25 years*	Vaccinated: 67 Unvaccinated: 96	Risk ratio (long‐term)	0.37 (0.12 to 1.18)	Unadjusted	Cross‐sectional; *age at outcome
Tozawa‐Ono 2021‐JPN	Cervarix (GSK bivalent); Gardasil (Merck quadrivalent)	Female, 12 to 16 years	Vaccinated: 3102 Unvaccinated: 8611	Risk ratio (medium‐term)	0.59 (0.17 to 2.07)	Unadjusted	Cross‐sectional
Baldur‐Felskov 2015‐DNK	Gardasil (Merck quadrivalent)	Females, 12 to 99 years*	70,753 cases	Incidence rate ratio (long‐term; 2000 vs 2019)	1.10 (1.02 to 1.19)	Age‐standardised	Pre‐ vs post‐vaccine introduction; *age at outcome
Benard 2017‐USA	Gardasil (Merck quadrivalent)	Female, 15 to 19 years*	135 cases	Annual percent change (long‐term; 2007‐2020)	‐34.0% (‐55.0 to ‐2.9)	Changes in cervical screening	Pre‐ vs post‐vaccine introduction; *age at outcome
Benard 2017‐USA	Gardasil (Merck quadrivalent)	Female, 20 to 24 years*	1187 cases	Annual percent change (long‐term; 2007‐2020)	‐5.8% (‐9.1 to ‐2.4)	Changes in cervical screening	Pre‐ vs post‐vaccine introduction; *age at outcome
Benard 2017‐USA	Gardasil (Merck quadrivalent)	Female, 25 to 29 years*	1501 cases	Annual percent change (long‐term; 2007‐2020)	5.2% (2.8 to 7.7)	Changes in cervical screening	Pre‐ vs post‐vaccine introduction; *age at outcome
Cuschieri 2023‐GBR	Cervarix (GSK bivalent)	Female, 20 to 25 years*	Pre‐vaccine: 397 Post‐vaccine: 1309	Odds ratio (long‐term; 2011 vs 2017)	0.34 (0.23 to 0.52)	Diagnosis year, year of birth, deprivation quintile	Pre‐ vs post‐vaccine introduction; *age at outcome
Donken 2021‐CAN	Gardasil (Merck quadrivalent)	Female, 9 to 14 years	Pre‐vaccine: 125,342 Post‐vaccine: 46,207	Incidence rate ratio (long‐term; 2004‐8 vs 2009‐17)	0.26 (0.16 to 0.42)	Birth year and age at first screening	Pre‐ vs post‐vaccine introduction
Goodman 2024‐DEU	Cervarix (GSK bivalent); Gardasil (Merck quadrivalent); Gardasil 9 (Merck nonavalent)	Female, 28 to 33 years	Pre‐vaccine: 22,533 Post‐vaccine: 38,987	Relative risk (long‐term; 2013 vs 2021)	0.44 (0.26 to 0.75)	Unadjusted	Pre‐ vs post‐vaccine introduction

CIN3: cervical intraepithelial neoplasia grade 3; NR: not reported; RCT: randomised controlled trial

**20:** Risk of bias summary: CIN3

**Study**	**Confounding**	**Selection**	**Classification of interventions**	**Deviations from intended interventions**	**Missing data**	**Measurement of outcomes**	**Selection of reported result**	**Overall risk of bias**
Falcaro 2021‐GBR	Moderate	Low	Low	Low	Low	Low	Low	Moderate
Paraskevaidis 2020‐GRC	Critical	Serious	Serious	Low	Serious	Low	Moderate	Critical
Yagi 2019‐JPN	Critical	Low	Low	Low	Low	Low	Low	Critical
Ikeda 2021‐JPN	Critical	Moderate	Low	Low	Low	Low	Low	Critical
Rana 2013‐FIN	Critical	Low	Low	Low	Low	Low	Low	Critical
Hiramatsu 2021‐JPN	Critical	Serious	Low	Low	Moderate	Low	Low	Critical
Munro 2017‐GBR	Critical	Low	Low	Low	Serious	Low	Low	Critical
Tozawa‐Ono 2021‐JPN	Critical	Moderate	Moderate	Low	Moderate	Low	Low	Critical
Baldur‐Felskov 2015‐DNK	Critical	Low	Serious	Low	Low	Low	Low	Critical
Benard 2017‐USA	Serious	Moderate	Serious	Low	Low	Low	Low	Serious
Cuschieri 2023‐GBR	Critical	Low	Serious	Low	Low	Low	Low	Critical
Donken 2021‐CAN	Serious	Low	Serious	Low	Moderate	Low	Low	Serious
Goodman 2024‐DEU	Critical	Low	Serious	Low	Low	Low	Low	Critical

CIN3: cervical intraepithelial neoplasia grade 3

Thirteen studies were included that reported on CIN3 following HPV vaccination (Baldur‐Felskov 2015‐DNK; Benard 2017‐USA; Cuschieri 2023‐GBR; Donken 2021‐CAN; Falcaro 2021‐GBR; Goodman 2024‐DEU; Hiramatsu 2021‐JPN; Ikeda 2021‐JPN; Munro 2017‐GBR; Paraskevaidis 2020‐GRC; Rana 2013‐FIN; Tozawa‐Ono 2021‐JPN; Yagi 2019‐JPN).

Three studies were cohort studies (Falcaro 2021‐GBR; Paraskevaidis 2020‐GRC; Yagi 2019‐JPN), two of which reported no cases of CIN3 in the HPV vaccine‐exposed groups (Paraskevaidis 2020‐GRC; Yagi 2019‐JPN). The other cohort study reported a large decrease in CIN3 incidence following HPV vaccine (RR 0.17, 95% CI 0.06 to 0.45; 1 cohort study, 214.8 million person‐years; I^2^ = 100%) (Analysis 1.6) (Falcaro 2021‐GBR). This decrease was greater when limited to those receiving the HPV vaccine at or before age 16 years (RR 0.09, 95% CI 0.01 to 0.70; 1 cohort study, 214.8 million person‐years; I^2^ = 99%) (Analysis 1.7).

One case‐control study reported a reduced odds of CIN3 following HPV vaccination (OR 0.27, 95% CI 0.08 to 0.89) (Ikeda 2021‐JPN).

One RCT extension study reported no CIN3 events in the HPV vaccine‐exposed group (Rana 2013‐FIN).

Three studies used a cross‐sectional design (Hiramatsu 2021‐JPN; Munro 2017‐GBR; Tozawa‐Ono 2021‐JPN), one of which reported no cases of CIN3 in the HPV vaccine‐exposed group (Hiramatsu 2021‐JPN). The other two studies reported a reduced risk of CIN3 following HPV vaccination but with confidence intervals that included no difference (Table).

Five studies were pre‐post vaccine introduction studies (Baldur‐Felskov 2015‐DNK; Benard 2017‐USA; Cuschieri 2023‐GBR; Donken 2021‐CAN; Goodman 2024‐DEU). One study reported an increased incidence of CIN3 between 1999 and 2009 (Baldur‐Felskov 2015‐DNK), while another reported a decrease for the youngest female age group (15 to 19 years) and an increase for the oldest group (25 to 29 years) (Benard 2017‐USA). Three other studies reported a decrease in CIN3 incidence comparing time periods before and after HPV vaccine introduction (Cuschieri 2023‐GBR; Donken 2021‐CAN; Goodman 2024‐DEU) (Table).

##### Cervical intraepithelial neoplasia grade 2 and above (CIN2+)

See Table for effect estimates and Table for the risk of bias summary of included studies on CIN2+. HPV vaccination probably reduces the incidence of CIN2+ (moderate‐certainty evidence; Table).

**21:** Primary clinical outcomes effect estimates: CIN2+

**Study**	**Vaccine**	**Population (sex, age at vaccination)**	**Sample size**	**Effect measure (time period)**	**Effect estimate**	**Adjustment factors**	**Notes**
Brotherton 2019‐AUS	Gardasil (Merck quadrivalent)	Female, 12 to 15 years	Vaccinated: 174,995 Unvaccinated: 48,845	Hazard ratio (3 doses, medium‐term)	0.59 (0.54 to 0.65)	Age, area of residence and socioeconomic status	Cohort
Brotherton 2019‐AUS	Gardasil (Merck quadrivalent)	Female, 12 to 15 years	Vaccinated: 18,190 Unvaccinated: 48,845	Hazard ratio (2 doses, medium‐term)	0.61 (0.52 to 0.72)	Age, area of residence and socioeconomic status	Cohort
Brotherton 2019‐AUS	Gardasil (Merck quadrivalent)	Female, 12 to 15 years	Vaccinated: 8618 Unvaccinated: 48,845	Hazard ratio (1 dose, medium‐term)	0.65 (0.52 to 0.81)	Age, area of residence and socioeconomic status	Cohort
Castle 2019‐USA	Gardasil (Merck quadrivalent)	Female, < 18 years	Vaccinated: 3911 Unvaccinated: 59,860	Risk ratio (medium‐term)	0.46 (0.29 to 0.75)	Unadjusted	Cohort
Castle 2019‐USA	Gardasil (Merck quadrivalent)	Female, 18 to 20 years	Vaccinated: 5999 Unvaccinated: 59,860	Risk ratio (medium‐term)	0.86 (0.65 to 1.15)	Unadjusted	Cohort
Castle 2019‐USA	Gardasil (Merck quadrivalent)	Female, 21 to 24 years	Vaccinated: 5238 Unvaccinated: 59,860	Risk ratio (medium‐term)	1.86 (1.50 to 2.31)	Unadjusted	Cohort
Dehlendorff 2018‐DNK/SWE	Gardasil (Merck quadrivalent)	Female, < 16 years	Vaccinated: 2,253,561 Unvaccinated: 2,091,579	Incidence rate ratio (long‐term)	0.23 (0.11 to 0.49)	Attained age, mother’s education, country	Cohort
Dehlendorff 2018‐DNK/SWE	Gardasil (Merck quadrivalent)	Female, 17 to 19 years	Vaccinated: 2,253,561 Unvaccinated: 2,091,579	Incidence rate ratio (long‐term)	0.65 (0.41 to 1.03)	Attained age, mother’s education, country	Cohort
Dehlendorff 2018‐DNK/SWE	Gardasil (Merck quadrivalent)	Female, 20 to 29 years	Vaccinated: 2,253,561 Unvaccinated: 2,091,579	Incidence rate ratio (long‐term)	1.31 (0.97 to 1.76)	Attained age, mother’s education, country	Cohort
Dehlendorff 2018‐DNK/SWE	Gardasil (Merck quadrivalent)	Female, < 16 years	Vaccinated: 2,253,561 Unvaccinated: 2,091,579	Incidence rate ratio (2 doses, long‐term)	0.44 (0.10 to 2.03)	Attained age, mother’s education, country	Cohort
Dehlendorff 2018‐DNK/SWE	Gardasil (Merck quadrivalent)	Female, 17 to 19 years	Vaccinated: 2,253,561 Unvaccinated: 2,091,579	Incidence rate ratio (2 doses, long‐term)	0.65 (0.25 to 1.74)	Attained age, mother’s education, country	Cohort
Dehlendorff 2018‐DNK/SWE	Gardasil (Merck quadrivalent)	Female, 20 to 29 years	Vaccinated: 2,253,561 Unvaccinated: 2,091,579	Incidence rate ratio (2 doses, long‐term)	1.56 (1.15 to 2.11)	Attained age, mother’s education, country	Cohort
Dehlendorff 2018‐DNK/SWE	Gardasil (Merck quadrivalent)	Female, < 16 years	Vaccinated: 2,253,561 Unvaccinated: 2,091,579	Incidence rate ratio (1 dose, long‐term)	0.23 (0.01 to 5.24)	Attained age, mother’s education, country	Cohort
Dehlendorff 2018‐DNK/SWE	Gardasil (Merck quadrivalent)	Female, 17 to 19 years	Vaccinated: 2,253,561 Unvaccinated: 2,091,579	Incidence rate ratio (1 dose, long‐term)	0.58 (0.15 to 2.19)	Attained age, mother’s education, country	Cohort
Dehlendorff 2018‐DNK/SWE	Gardasil (Merck quadrivalent)	Female, 20 to 29 years	Vaccinated: 2,253,561 Unvaccinated: 2,091,579	Incidence rate ratio (1 dose, long‐term)	1.56 (1.13 to 2.15)	Attained age, mother’s education, country	Cohort
Del Mistro 2021‐ITA	Gardasil (Merck quadrivalent)	Female, 15 to 25 years	Vaccinated: 4718 Unvaccinated: 91,512	Risk ratio (long‐term)	0.66 (0.41 to 1.06)	Unadjusted	Cohort
Donken 2021‐CAN	Gardasil (Merck quadrivalent)	Female, 9 to 14 years	Vaccinated: 18,975 Unvaccinated: 14,130	Incidence rate ratio (long‐term)	0.42 (0.31 to 0.57)	Birth year, age at first screening	Cohort
Herweijer 2016‐SWE	Gardasil (Merck quadrivalent)	Female, 11 to 16 years	Vaccinated: 236,372 Unvaccinated: 1,097,319	Incidence rate ratio (long‐term)	0.25 (0.18 to 0.35)	Age, parental highest education	Cohort
Herweijer 2016‐SWE	Gardasil (Merck quadrivalent)	Female, 17 to 19 years	Vaccinated: 236,372 Unvaccinated: 1,097,319	Incidence rate ratio (long‐term)	0.54 (0.46 to 0.64)	Age, parental highest education	Cohort
Herweijer 2016‐SWE	Gardasil (Merck quadrivalent)	Female, 20 to 29 years	Vaccinated: 236,372 Unvaccinated: 1,097,319	Incidence rate ratio (long‐term)	0.78 (0.65 to 0.93)	Age, parental highest education	Cohort
Innes 2020‐NZL	Gardasil (Merck quadrivalent)	Female, 14 to 20 years	Vaccinated: 134,563 Unvaccinated: 175,748	Incidence rate ratio (long‐term)	0.69 (0.64 to 0.75)	Unadjusted	Cohort
Kjaer 2020‐EU	Gardasil (Merck quadrivalent)	Female, 16 to 23 years	Vaccinated: 2121 Unvaccinated: NR	Vaccine effectiveness (3 doses; long‐term)	100 (94.7 to 100)	Unadjusted	Cohort
Kjaer 2021‐EU	Gardasil9 (Merck quadrivalent)	Female, 16 to 26 years	Vaccinated: 1783 Unvaccinated: NR	Incidence rate ratio (long‐term)	0.12 (0.00 to 0.72)	Unadjusted	Cohort; no events in exposed group
Lei 2020a‐SWE	Cervarix (GSK bivalent); Gardasil (Merck quadrivalent)	Female, 10 to 16 years	Vaccinated: 25,865 Unvaccinated: 100,400	Risk ratio (long‐term)	0.42 (0.37 to 0.46)	Birth cohort	Cohort
Lei 2020a‐SWE	Cervarix (GSK bivalent); Gardasil (Merck quadrivalent)	Female, 17 to 22 years	Vaccinated: 26,892 Unvaccinated: 100,400	Risk ratio (long‐term)	0.61 (0.56 to 0.67)	Birth cohort	Cohort
Martellucci 2022‐ITA	Cervarix (GSK bivalent); Gardasil (Merck quadrivalent)	Female, 25 to 30 years	Vaccinated: 1118 Unvaccinated: 3547	Odds ratio (3 doses; long‐term)	0.33 (0.11 to 0.96)	Age at screening test, country of birth, residential area, number of screening tests, and municipality average income	Cohort
Martellucci 2022‐ITA	Cervarix (GSK bivalent); Gardasil (Merck quadrivalent)	Female, 25 to 30 years	Vaccinated: 1118 Unvaccinated: 3547	Odds ratio (at least 1 dose; long‐term)	0.31 (0.11 to 0.91)	Age at screening test, country of birth, residential area, number of screening tests, and municipality average income	Cohort
Orumaa 2024‐NOR	Gardasil (Merck quadrivalent)	Female, 16 to 30 years	Vaccinated: 626 Unvaccinated: 18,098	Incidence rate ratio (medium‐term)	0.39 (0.36 to 0.43)	Age, calendar year	Cohort
Orumaa 2024‐NOR	Gardasil (Merck quadrivalent)	Female, < 17 years at vaccination	Vaccinated: 225 Unvaccinated: 18,098	Incidence rate ratio (medium‐term)	0.18 (0.16 to 0.21)	Age, calendar year	Cohort
Rodriguez 2020‐USA	Gardasil (Merck quadrivalent)	Female, 9 to 14 years	Vaccinated: 3784 Unvaccinated: 5844	Hazard ratio (3 doses; medium‐term)	0.71 (0.37 to 1.38)	Age, census region, STD history, pregnancy history	Cohort
Rodriguez 2020‐USA	Gardasil (Merck quadrivalent)	Female, 15 to 19 years	Vaccinated: 24,018 Unvaccinated: 39,264	Hazard ratio (3 doses; medium‐term)	0.66 (0.55 to 0.80)	Age, census region, STD history, pregnancy history	Cohort
Rodriguez 2020‐USA	Gardasil (Merck quadrivalent)	Female, > 20 years	Vaccinated: 11,021 Unvaccinated: 21,433	Hazard ratio (3 doses; medium‐term)	0.96 (0.77 to 1.20)	Age, census region, STD history, pregnancy history	Cohort
Rodriguez 2020‐USA	Gardasil (Merck quadrivalent)	Female, 9 to 14 years	Vaccinated: 1230 Unvaccinated: 5844	Hazard ratio (2 doses; medium‐term)	0.46 (0.13 to 1.62)	Age, census region, STD history, pregnancy history	Cohort
Rodriguez 2020‐USA	Gardasil (Merck quadrivalent)	Female, 15 to 19 years	Vaccinated: 8147 Unvaccinated: 39,264	Hazard ratio (2 doses; medium‐term)	0.72 (0.54 to 0.95)	Age, census region, STD history, pregnancy history	Cohort
Rodriguez 2020‐USA	Gardasil (Merck quadrivalent)	Female, > 20 years	Vaccinated: 4711 Unvaccinated: 21,433	Hazard ratio (2 doses; medium‐term)	1.02 (0.75 to 1.38)	Age, census region, STD history, pregnancy history	Cohort
Rodriguez 2020‐USA	Gardasil (Merck quadrivalent)	Female, 9 to 14 years	Vaccinated: 830 Unvaccinated: 5844	Hazard ratio (1 dose; medium‐term)	0.87 (0.28 to 2.68)	Age, census region, STD history, pregnancy history	Cohort
Rodriguez 2020‐USA	Gardasil (Merck quadrivalent)	Female, 15 to 19 years	Vaccinated: 7099 Unvaccinated: 39,264	Hazard ratio (1 dose; medium‐term)	0.64 (0.47 to 0.88)	Age, census region, STD history, pregnancy history	Cohort
Rodriguez 2020‐USA	Gardasil (Merck quadrivalent)	Female, > 20 years	Vaccinated: 5701 Unvaccinated: 21,433	Hazard ratio (1 dose; medium‐term)	1.16 (0.89 to 1.52)	Age, census region, STD history, pregnancy history	Cohort
Verdoodt 2020‐DNK	Gardasil (Merck quadrivalent)	Female, < 16 years	Vaccinated: 215,309 Unvaccinated: 374,774	Incidence rate ratio (long‐term)	0.43 (0.36 to 0.51)	Attained age, socioeconomic position	Cohort
Yagi 2019‐JPN	Cervarix (GSK bivalent); Gardasil (Merck quadrivalent)	Female, 12 to 16 years	Vaccinated: 7389 Unvaccinated: 7872	Risk ratio (long‐term)	0.18 (0.04 to 0.79)	Unadjusted	Cohort
Crowe 2014‐AUS	Gardasil (Merck quadrivalent)	Female, NR	Cases: 1062 Controls: 96,404	Odds ratio (3 doses, medium‐term)	0.54 (0.43 to 0.67)	Socioeconomic status, remoteness, year of birth, follow‐up times	Case‐control
Crowe 2014‐AUS	Gardasil (Merck quadrivalent)	Female, NR	Cases: 1062 Controls: 96,404	Odds ratio (2 doses, medium‐term)	0.79 (0.64 to 0.98)	Socioeconomic status, remoteness, year of birth, follow‐up times	Case‐control
Crowe 2014‐AUS	Gardasil (Merck quadrivalent)	Female, NR	Cases: 1062 Controls: 96,404	Odds ratio (1 dose, medium‐term)	0.95 (0.77 to 1.16)	Socioeconomic status, remoteness, year of birth, follow‐up times	Case‐control
Ikeda 2021‐JPN	Cervarix (GSK bivalent); Gardasil (Merck quadrivalent)	Female, 13 to 16 years	Cases: 217 Controls: 12,296	Odds ratio (medium‐term)	0.25 (0.12 to 0.54)	Unadjusted	Case‐control
Silverberg 2018‐USA	Gardasil (Merck quadrivalent)	Female, 14 to 17 years	Cases: 4005 Controls: 19,881	Incidence rate ratio (long‐term)	0.62 (0.46 to 0.83)	Matched by age, time since first cytology, years of health plan membership	Case‐control
Silverberg 2018‐USA	Gardasil (Merck quadrivalent)	Female, 18 to 20 years	Cases: 4041 Controls: 20,051	Incidence rate ratio (long‐term)	0.76 (0.61 to 0.94)	Matched by age, time since first cytology, years of health plan membership	Case‐control
Silverberg 2018‐USA	Gardasil (Merck quadrivalent)	Female, ≥ 21 years	Cases: 4167 Controls: 20,571	Incidence rate ratio (long‐term)	0.98 (0.84 to 1.13)	Matched by age, time since first cytology, years of health plan membership	Case‐control
Silverberg 2018‐USA	Gardasil (Merck quadrivalent)	Female, 14 to 21 years	Cases: 4025 Controls: 19,882	Incidence rate ratio (2 doses; long‐term)	1.02 (0.82 to 1.28)	Matched by age, time since first cytology, years of health plan membership	Case‐control
Silverberg 2018‐USA	Gardasil (Merck quadrivalent)	Female, 14 to 21 years	Cases: 4046 Controls: 20,003	Incidence rate ratio (1 dose; long‐term)	0.89 (0.73 to 1.09)	Matched by age, time since first cytology, years of health plan membership	Case‐control
Sankaranarayanan 2018‐IND	Gardasil (Merck quadrivalent)	Female, 10 to 18 years	Vaccinated: 2019 Unvaccinated: 1484	Risk ratio (3 doses; long‐term)	0.06 (0.00 to 1.01)	Unadjusted	RCT extension; no events in exposed group
Sankaranarayanan 2018‐IND	Gardasil (Merck quadrivalent)	Female, 10 to 18 years	Vaccinated: 2166 Unvaccinated: 1484	Risk ratio (2 doses; long‐term)	0.05 (0.00 to 0.94)	Unadjusted	RCT extension; no events in exposed group
Sankaranarayanan 2018‐IND	Gardasil (Merck quadrivalent)	Female, 10 to 18 years	Vaccinated: 2858 Unvaccinated: 1484	Risk ratio (1 dose; long‐term)	0.08 (0.01 to 0.72)	Unadjusted	RCT extension
Kreimer 2011‐CRI	Cervarix (GSK bivalent)	Female, 18 to 25 years	Vaccinated: 1365 Unvaccinated: 1783	Incidence rate ratio (long‐term)	0.026 (0.004 to 0.12)	Age‐ and location‐matched	RCT extension
Dorton 2015‐USA	Gardasil (Merck quadrivalent)	Female, ≤ 26 years*	Vaccinated: 481 Unvaccinated: 911	Risk ratio (long‐term)	0.71 (0.58 to 0.89)	Unadjusted	Cross‐sectional; *age at outcome
Hikari 2022‐JPN	Cervarix (GSK bivalent); Gardasil (Merck quadrivalent)	Female, 20 to 24 years	Vaccinated: 2467 Unvaccinated: 4786	Odds ratio (long‐term)	0.46 (0.21 to 1.00)	Smoking	Cross‐sectional
Hiramatsu 2021‐JPN	Cervarix (GSK bivalent); Gardasil (Merck quadrivalent)	Female 12 to 18 years	Vaccinated: 170 Unvaccinated: 877	Risk ratio (medium‐term)	0.57 (0.03 to 10.60)	Unadjusted	Cross‐sectional; no events in exposed or unexposed groups
Munro 2017‐GBR	Cervarix (GSK bivalent; Gardasil (Merck quadrivalent)	Female 20 to 25 years*	Vaccinated: 69 Unvaccinated: 286	Risk ratio (long‐term)	0.60 (0.35 to 1.01)	Unadjusted	Cross‐sectional; *age at outcome
Muresu 2022‐ITA	Gardasil (Merck quadrivalent); Gardasil 9 (Merck nonavalent)	Female, 24 to 64 years	Vaccinated: 311 Unvaccinated: 875	Odds ratio (medium‐term)	1.13 (0.42 to 3.04)	Age, age at first vaccine dose, education, civil status	Cross‐sectional
Ozawa 2017‐JPN	Cervarix (GSK bivalent); Gardasil (Merck quadrivalent)	Female, 12 to 16 years	Vaccinated: 1002 Unvaccinated: 4922	Risk ratio (long‐term)	0.25 (0.02 to 4.44)	Unadjusted	Cross‐sectional; no events in exposed group
Shiko 2020‐JPN	Cervarix (GSK bivalent)	Female, 12 to 16 years	Vaccinated: 3770 Unvaccinated: 30,511	Risk ratio (medium‐term)	0.24 (0.10 to 0.60)	Age, place of screening	Cross‐sectional
Tanaka 2017‐JPN	Cervarix (GSK bivalent)	Female, 12 to 16 years	Vaccinated: 413 Unvaccinated: 2012	Risk ratio (long‐term)	0.26 (0.02 to 4.41)	Unadjusted	Cross‐sectional; no cases in exposed group
Tozawa‐Ono 2021‐JPN	Cervarix (GSK bivalent); Gardasil (Merck quadrivalent)	Female, 12 to 16 years	Vaccinated: 3102 Unvaccinated: 8611	Risk ratio (medium‐term)	0.86 (0.46 to 1.60)	Unadjusted	Cross‐sectional
Wright 2019‐USA	Gardasil (Merck quadrivalent)	Female, 11 to 26 years	Vaccinated: 2977 Unvaccinated: 11,176	Odds ratio (medium‐term)	0.80 (0.60 to 1.10)	Age	Cross‐sectional
Baldur‐Felskov 2014‐DNK	Gardasil (Merck quadrivalent)	Female, 12 to 26 years	Pre‐vaccine: 2,302,441 Post‐vaccine: 2,431,726	Risk ratio (long‐term; 2000 vs 2012)	1.58 (1.52 to 1.64)	Unadjusted	Pre‐ vs post‐vaccine introduction
Cruickshank 2017‐GBR	Cervarix (GSK bivalent; Gardasil (Merck quadrivalent)	Female, 12 to 18 years	Pre‐vaccine: 1344 Post‐vaccine: 5669	Risk ratio (long‐term; 2008‐9 vs 2009‐2014)	0.88 (0.81 to 0.95)	Unadjusted	Pre‐ vs post‐vaccine introduction
Cuschieri 2023‐GBR	Cervarix (GSK bivalent)	Female, 20 to 25 years	Pre‐vaccine: 397 Post‐vaccine: 1309	Odds ratio (long‐term; 2011 vs 2017)	0.3 (0.2 to 0.4)	Diagnosis year, year of birth, deprivation quintile	Pre‐ vs post‐vaccine introduction
Gargano 2023‐USA	Gardasil (Merck quadrivalent)	Female, 20 to 24 years	4191 cases	Average annual percent change (2008 to 2016)	‐8.4% (‐12.1 to ‐4.7)	Unadjusted	Pre‐ vs post‐vaccine introduction
Gargano 2023‐USA	Gardasil (Merck quadrivalent)	Female, 25 to 29 years	6585 cases	Average annual percent change (2008 to 2016)	2.6% (0.4 to 4.8)	Unadjusted	Pre‐ vs post‐vaccine introduction
Gargano 2023‐USA	Gardasil (Merck quadrivalent)	Female, 30 to 34 years	4805 cases	Average annual percent change (2008 to 2016)	6.4% (1.1 to 11.9)	Unadjusted	Pre‐ vs post‐vaccine introduction
Gargano 2023‐USA	Gardasil (Merck quadrivalent)	Female, 35 to 39 years	2753 cases	Average annual percent change (2008 to 2016)	8.9% (4.1 to 13.9)	Unadjusted	Pre‐ vs post‐vaccine introduction
Gargano 2023‐USA	Gardasil (Merck quadrivalent)	Female, 20 to 24 years	4191 cases	Incidence rate ratio (2008‐2009 vs 2015‐2016)	0.49 (0.42 to 0.56)	Unadjusted	Pre‐ vs post‐vaccine introduction
Gargano 2023‐USA	Gardasil (Merck quadrivalent)	Female, 25 to 29 years	6585 cases	Incidence rate ratio (2008‐2009 vs 2015‐2016)	1.20 (1.09 to 1.31)	Unadjusted	Pre‐ vs post‐vaccine introduction
Gargano 2023‐USA	Gardasil (Merck quadrivalent)	Female, 30 to 34 years	4805 cases	Incidence rate ratio (2008‐2009 vs 2015‐2016)	1.60 (1.43 to 1.79)	Unadjusted	Pre‐ vs post‐vaccine introduction
Gargano 2023‐USA	Gardasil (Merck quadrivalent)	Female, 35 to 39 years	2753 cases	Incidence rate ratio (2008‐2009 vs 2015‐2016)	1.97 (1.70 to 2.29)	Unadjusted	Pre‐ vs post‐vaccine introduction
Goodman 2024‐DEU	Cervarix (GSK bivalent); Gardasil (Merck quadrivalent); Gardasil 9 (Merck nonavalent)	Female, 28 to 33 years	Pre‐vaccine: 22,533 Post‐vaccine: 38,987	Relative risk (long‐term)	0.49 (0.39 to 0.62)	Unadjusted	Pre‐ vs post‐vaccine introduction
Rebolj 2022‐GBR	Cervarix (GSK bivalent);	Female, 24 to 25 years	N = 64,274	Vaccine effectiveness (long‐term)	72% (66 to 77)	Deprivation and laboratory	Pre‐ vs post‐vaccine introduction
Thamsborg 2020‐DNK	Gardasil (Merck quadrivalent)	Female, 15 years	Pre‐vaccine: 19,629 Post‐vaccine: 26,215	Incidence rate ratio (long‐term; 1999‐2008 vs 2009‐2018)	0.74 (0.66 to 0.82)	Unadjusted	Pre‐ vs post‐vaccine introduction

CIN2+: cervical intraepithelial neoplasia grade 2 or higher; NR: not reported; RCT: randomised controlled trial; STD: sexually transmitted disease

**22:** Risk of bias summary: CIN2+

**Study**	**Confounding**	**Selection**	**Classification of interventions**	**Deviations from intended interventions**	**Missing data**	**Measurement of outcomes**	**Selection of reported result**	**Overall risk of bias**
Brotherton 2019‐AUS	Serious	Serious	Low	Low	Moderate	Low	Moderate	Serious
Castle 2019‐USA	Critical	Moderate	Low	Low	Low	Low	Low	Critical
Dehlendorff 2018‐DNK/SWE	Serious	Low	Low	Low	Low	Low	Low	Serious
Del Mistro 2021‐ITA	Critical	Low	Low	Low	Low	Low	Low	Critical
Donken 2021‐CAN	Serious	Low	Serious	Low	Moderate	Low	Low	Serious
Herweijer 2016‐SWE	Serious	Low	Low	Low	Low	Low	Low	Serious
Innes 2020‐NZL	Critical	Serious	Low	Low	Moderate	Low	Moderate	Critical
Kjaer 2020‐EU	Critical	Serious	Low	Low	Moderate	Low	Low	Critical
Kjaer 2021‐EU	Critical	Serious	Low	Low	Moderate	Low	Low	Critical
Martellucci 2022‐ITA	Serious	Low	Low	Low	Moderate	Low	Low	Serious
Orumaa 2024‐NOR	Serious	Low	Low	Low	Low	Low	Low	Serious
Rodriguez 2020‐USA	Moderate	Low	Low	Low	Low	Low	Low	Moderate
Verdoodt 2020‐DNK	Serious	Low	Low	Low	Low	Low	Low	Serious
Yagi 2019‐JPN	Critical	Low	Low	Low	Low	Low	Low	Critical
Crowe 2014‐AUS	Serious	Serious	Low	Low	Low	Low	Low	Serious
Ikeda 2021‐JPN	Critical	Moderate	Low	Low	Low	Low	Low	Critical
Silverberg 2018‐USA	Serious	Serious	Low	Low	Low	Low	Low	Serious
Sankaranarayanan 2018‐IND	Moderate	Low	Low	Low	Moderate	Low	Low	Moderate
Kreimer 2011‐CRI	Moderate	Moderate	Low	Low	Moderate	Low	Low	Moderate
Dorton 2015‐USA	Critical	Serious	Moderate	Low	Moderate	Low	Moderate	Critical
Hikari 2022‐JPN	Critical	Moderate	Moderate	Low	Moderate	Low	Low	Critical
Hiramatsu 2021‐JPN	Critical	Serious	Low	Low	Moderate	Low	Low	Critical
Lei 2020a‐SWE	Serious	Moderate	Low	Low	Low	Low	Low	Serious
Munro 2017‐GBR	Critical	Low	Low	Low	Serious	Low	Low	Critical
Muresu 2022‐ITA	Serious	Low	Serious	No information	Moderate	Low	Low	Serious
Ozawa 2017‐JPN	Critical	Moderate	Moderate	Low	Low	Low	Low	Critical
Shiko 2020‐JPN	Critical	Moderate	Moderate	Low	Moderate	Low	Low	Critical
Tanaka 2017‐JPN	Critical	Moderate	Moderate	Low	Serious	Low	Low	Critical
Tozawa‐Ono 2021‐JPN	Critical	Moderate	Moderate	Low	Moderate	Low	Low	Critical
Wright 2019‐USA	Critical	Moderate	Moderate	Low	Low	Low	Low	Critical
Baldur‐Felskov 2014‐DNK	Critical	Low	Serious	Low	Low	Low	Low	Critical
Cruickshank 2017‐GBR	Critical	Moderate	Serious	Low	Low	Low	Low	Critical
Cuschieri 2023‐GBR	Critical	Low	Serious	Low	Low	Low	Low	Critical
Gargano 2023‐USA	Critical	Moderate	Moderate	Low	Low	Low	Low	Critical
Goodman 2024‐DEU	Critical	Low	Serious	Low	Low	Low	Low	Critical
Rebolj 2022‐GBR	Serious	Moderate	Moderate	Low	Low	Low	Low	Serious
Thamsborg 2020‐DNK	Critical	Low	Serious	Low	Low	Low	Low	Critical

CIN2+: cervical intraepithelial neoplasia grade 2 or higher

Thirty‐seven studies were identified that reported on CIN2+ following HPV vaccination (Baldur‐Felskov 2014‐DNK; Brotherton 2019‐AUS; Castle 2019‐USA; Crowe 2014‐AUS; Cruickshank 2017‐GBR; Cuschieri 2023‐GBR; Dehlendorff 2018‐DNK/SWE; Del Mistro 2021‐ITA; Donken 2021‐CAN; Dorton 2015‐USA; Gargano 2023‐USA; Goodman 2024‐DEU; Herweijer 2016‐SWE; Hikari 2022‐JPN; Hiramatsu 2021‐JPN; Ikeda 2021‐JPN; Innes 2020‐NZL; Kjaer 2020‐EU; Kjaer 2021‐EU; Kreimer 2011‐CRI; Lei 2020a‐SWE; Martellucci 2022‐ITA; Munro 2017‐GBR; Muresu 2022‐ITA; Orumaa 2024‐NOR; Ozawa 2017‐JPN; Rebolj 2022‐GBR; Rodriguez 2020‐USA; Sankaranarayanan 2018‐IND; Shiko 2020‐JPN; Silverberg 2018‐USA; Tanaka 2017‐JPN; Thamsborg 2020‐DNK; Tozawa‐Ono 2021‐JPN; Verdoodt 2020‐DNK; Wright 2019‐USA; Yagi 2019‐JPN).

Fifteen were cohort studies (Brotherton 2019‐AUS; Castle 2019‐USA; Dehlendorff 2018‐DNK/SWE; Del Mistro 2021‐ITA; Donken 2021‐CAN; Herweijer 2016‐SWE; Innes 2020‐NZL; Kjaer 2020‐EU; Kjaer 2021‐EU; Lei 2020a‐SWE; Martellucci 2022‐ITA; Orumaa 2024‐NOR; Rodriguez 2020‐USA; Verdoodt 2020‐DNK; Yagi 2019‐JPN), three were case‐control studies (Crowe 2014‐AUS; Ikeda 2021‐JPN; Silverberg 2018‐USA), two were RCT extensions (Kreimer 2011‐CRI; Sankaranarayanan 2018‐IND), 10 were cross‐sectional studies (Dorton 2015‐USA; Hikari 2022‐JPN; Hiramatsu 2021‐JPN; Munro 2017‐GBR; Muresu 2022‐ITA; Ozawa 2017‐JPN; Shiko 2020‐JPN; Tanaka 2017‐JPN; Tozawa‐Ono 2021‐JPN; Wright 2019‐USA), and seven were pre‐post vaccine introduction studies (Baldur‐Felskov 2014‐DNK; Cruickshank 2017‐GBR; Cuschieri 2023‐GBR; Gargano 2023‐USA; Goodman 2024‐DEU; Rebolj 2022‐GBR; Thamsborg 2020‐DNK).

One of the cohort studies did not report any cases of CIN2+ in the HPV vaccine‐exposed group (Kjaer 2021‐EU). When pooled, the cohort studies indicated a reduction of CIN2+ incidence following HPV vaccination of 38% in the medium term (RR 0.62, 95% CI 0.45 to 0.85; 3 cohort studies, 347,928 females; I^2^ = 95%) and 49% in the long term (RR 0.51, 95% CI 0.41 to 0.64; 6 cohort studies, 6,464,506 females; I^2^ = 91%) (Analysis 1.8). This decrease was 62% in the long term when limited to those receiving the HPV vaccine before age 16 years (RR 0.38, 95% CI 0.31 to 0.45; 5 cohort studies, 6,455,176 females; I^2^ = 64%) (Analysis 1.9).

The case‐control studies all reported decreased odds of CIN2+ following HPV vaccination (Crowe 2014‐AUS; Ikeda 2021‐JPN; Silverberg 2018‐USA) (Table).

Of the two RCT extension studies, one did not identify any cases of CIN2+ in the HPV vaccine‐exposed group (Sankaranarayanan 2018‐IND). The other reported a large decrease of CIN2+ incidence (IRR 0.026, 95% CI 0.004 to 0.12) following HPV vaccination (Kreimer 2011‐CRI) (Table).

Of the cross‐sectional studies, three did not report any cases of CIN2+ in the HPV vaccine‐exposed group (Hiramatsu 2021‐JPN; Ozawa 2017‐JPN; Tanaka 2017‐JPN). Four studies reported adjusted estimates (Hikari 2022‐JPN; Muresu 2022‐ITA; Shiko 2020‐JPN; Wright 2019‐USA), which, when pooled, showed a decreased risk of CIN2+ following HPV vaccination of 38% in the medium term (RR 0.62, 95% CI 0.28 to 1.34; 3 cross‐sectional studies, 49,620 females; I^2^ = 72%) and 54% in the long term (RR 0.46, 95% CI 0.21 to 1.00; 1 cross‐sectional study, 7253 females) (Analysis 1.10). Three additional cross‐sectional studies reported only unadjusted estimates (Dorton 2015‐USA; Munro 2017‐GBR; Tozawa‐Ono 2021‐JPN).

One pre‐post vaccine introduction study reported an increase in CIN2+ incidence between 2000 and 2012 (Baldur‐Felskov 2014‐DNK), while the other six reported a reduced incidence (Cruickshank 2017‐GBR; Cuschieri 2023‐GBR; Gargano 2023‐USA; Goodman 2024‐DEU; Rebolj 2022‐GBR; Thamsborg 2020‐DNK) (Table).

Six studies were identified that reported on the effectiveness of two doses or one dose of HPV vaccine against CIN2+ (Brotherton 2019‐AUS; Crowe 2014‐AUS; Dehlendorff 2018‐DNK/SWE; Rodriguez 2020‐USA; Sankaranarayanan 2018‐IND; Silverberg 2018‐USA). Effectiveness was inconsistent across studies, with four studies indicating a reduction of CIN2+ following two doses in some age groups (Brotherton 2019‐AUS; Crowe 2014‐AUS; Rodriguez 2020‐USA; Sankaranarayanan 2018‐IND), while two did not. Three studies indicated a reduction of CIN2+ following one dose of HPV vaccine (Brotherton 2019‐AUS; Rodriguez 2020‐USA; Sankaranarayanan 2018‐IND).

##### Cervical intraepithelial neoplasia grade 2 (CIN2)

See Table for effect estimates and Table for the risk of bias summary of included studies on CIN2. HPV vaccination probably reduces the incidence of CIN2 (moderate‐certainty evidence; Table).

**23:** Primary clinical outcomes effect estimates: CIN2

**Study**	**Vaccine**	**Population (sex, age at vaccination)**	**Sample size**	**Effect measure (time period)**	**Effect estimate**	**Adjustment factors**	**Notes**
Donken 2021‐CAN	Gardasil (Merck quadrivalent)	Female, 9 to 14 years	Vaccinated: 18,975 Unvaccinated: 14,130	Incidence rate ratio (long‐term)	0.59 (0.40 to 0.88)	Birth year, age at first screening	Cohort
Palmer 2019‐GBR	Cervarix (GSK bivalent)	Female, 12 to 13 years	NR	Odds ratio (3 doses; long‐term)	0.11 (0.06 to 0.19)	Deprivation, rurality	Cohort
Palmer 2019‐GBR	Cervarix (GSK bivalent)	Female, 12 to 18+ years	NR	Odds ratio (2 doses; long‐term)	0.70 (0.45 to 1.07)	Deprivation, rurality	Cohort
Palmer 2019‐GBR	Cervarix (GSK bivalent)	Female, 12 to 18+ years	NR	Odds ratio (1 dose; long‐term)	0.95 (0.56 to 1.59)	Deprivation, rurality	Cohort
Paraskevaidis 2020‐GRC	NR	Female, NR	Vaccinated: 849 Unvaccinated: 849	Risk ratio (long‐term)	0.04 (0.01 to 0.30)	Unadjusted	Cohort
Yagi 2019‐JPN	Cervarix (GSK bivalent); Gardasil (Merck quadrivalent)	Female, 12 to 16 years	Vaccinated: 7389 Unvaccinated: 7872	Risk ratio (long‐term)	0.43 (0.08 to 2.20)	Unadjusted	Cohort
Ikeda 2021‐JPN	Cervarix (GSK bivalent); Gardasil (Merck quadrivalent)	Female, 13 to 16 years	Cases: 165 Controls: 12,296	Odds ratio (medium‐term)	0.57 (0.36 to 0.90)	Unadjusted	Case‐control
Munro 2017‐GBR	Cervarix (GSK bivalent); Gardasil (Merck quadrivalent)	Female 20 to 25 years*	Vaccinated: 67 Unvaccinated: 294	Risk ratio (long‐term)	0.72 (0.37 to 1.39)	Unadjusted	Cross‐sectional; *age at outcome
Tozawa‐Ono 2021‐JPN	Cervarix (GSK bivalent); Gardasil (Merck quadrivalent)	Female, 12 to 16 years	Vaccinated: 3102 Unvaccinated: 8611	Risk ratio (medium‐term)	0.99 (0.48 to 2.04)	Unadjusted	Cross‐sectional
Benard 2017‐USA	Gardasil (Merck quadrivalent)	Female, 15 to 19 years*	421 cases	Annual percent change (long‐term; 2007 vs 2014)	‐10.5% (‐18.8% to ‐1.2%)	Changes in cervical screening	Pre‐ vs post‐vaccine introduction; *age at outcome
Benard 2017‐USA	Gardasil (Merck quadrivalent)	Female, 20 to 24 years*	2028 cases	Annual percent change (long‐term; 2007 vs 2014)	‐6.3% (‐10.9% to ‐1.4%)	Changes in cervical screening	Pre‐ vs post‐vaccine introduction; *age at outcome
Benard 2017‐USA	Gardasil (Merck quadrivalent)	Female, 25 to 29 years*	1847 cases	Annual percent change (long‐term; 2007 vs 2014)	1.9% (‐1.6% to 5.5%)	Changes in cervical screening	Pre‐ vs post‐vaccine introduction; *age at outcome
Cuschieri 2023‐GBR	Cervarix (GSK bivalent)	Female, 20 to 25 years*	Pre‐vaccine: 397 Post‐vaccine: 1309	Odds ratio (long‐term; 2011 vs 2017)	0.32 (0.21 to 0.48)	Diagnosis year, year of birth, deprivation quintile	Pre‐ vs post‐vaccine introduction; *age at outcome
Goodman 2024‐DEU	Cervarix (GSK bivalent); Gardasil (Merck quadrivalent); Gardasil 9 (Merck nonavalent)	Female 28 to 33 years	Pre‐vaccine: 22,533 Post‐vaccine: 38,987	Relative risk (long‐term)	0.85 (0.53 to 1.39)	Unadjusted	Pre‐ vs post‐vaccine introduction
Thamsborg 2020‐DNK	Gardasil (Merck quadrivalent)	Female, 15 years	Pre‐vaccine: 19,629 Post‐vaccine: 26,215	Incidence rate ratio (long‐term; 1999‐2008 vs 2009‐2018)	0.82 (0.68 to 0.96)	Unadjusted	Pre‐ vs post‐vaccine introduction

CIN2: cervical intraepithelial neoplasia grade 2; NR: not reported

**24:** Risk of bias summary: CIN2

**Study**	**Confounding**	**Selection**	**Classification of interventions**	**Deviations from intended interventions**	**Missing data**	**Measurement of outcomes**	**Selection of reported result**	**Overall risk of bias**
Donken 2021‐CAN	Serious	Low	Serious	Low	Moderate	Low	Low	Serious
Palmer 2019‐GBR	Serious	Moderate	Low	Low	Moderate	Low	Low	Serious
Paraskevaidis 2020‐GRC	Critical	Serious	Serious	Low	Serious	Low	Moderate	Critical
Yagi 2019‐JPN	Critical	Low	Low	Low	Low	Low	Low	Critical
Ikeda 2021‐JPN	Critical	Moderate	Low	Low	Low	Low	Low	Critical
Munro 2017‐GBR	Critical	Low	Low	Low	Serious	Low	Low	Critical
Tozawa‐Ono 2021‐JPN	Critical	Moderate	Moderate	Low	Moderate	Low	Low	Critical
Benard 2017‐USA	Critical	Moderate	Serious	Low	Low	Low	Low	Critical
Cuschieri 2023‐GBR	Critical	Low	Serious	Low	Low	Low	Low	Critical
Goodman 2024‐DEU	Critical	Low	Serious	Low	Low	Low	Low	Critical
Thamsborg 2020‐DNK	Critical	Low	Serious	Low	Low	Low	Low	Critical

CIN2: cervical intraepithelial neoplasia grade 2

Eleven studies were identified that reported on CIN2 following HPV vaccination (Benard 2017‐USA; Cuschieri 2023‐GBR; Donken 2021‐CAN; Goodman 2024‐DEU; Ikeda 2021‐JPN; Munro 2017‐GBR; Palmer 2019‐GBR; Paraskevaidis 2020‐GRC; Thamsborg 2020‐DNK; Tozawa‐Ono 2021‐JPN; Yagi 2019‐JPN).

Four were cohort studies (Donken 2021‐CAN; Palmer 2019‐GBR; Paraskevaidis 2020‐GRC; Yagi 2019‐JPN), one was a case‐control study (Ikeda 2021‐JPN), two were cross‐sectional (Munro 2017‐GBR; Tozawa‐Ono 2021‐JPN), and four were pre‐post vaccine introduction studies (Benard 2017‐USA; Cuschieri 2023‐GBR; Goodman 2024‐DEU; Thamsborg 2020‐DNK).

Of the cohort studies, one reported a decreased incidence of CIN2 following HPV vaccination (IRR 0.59, 95% CI 0.40 to 0.88; 33,105 females) (Donken 2021‐CAN), while another reported a reduced odds of CIN2 (OR 0.11, 95% CI 0.06 to 0.19) (Palmer 2019‐GBR). The other two cohort studies also reported a decreased risk of CIN2, but the effects were not adjusted for confounding (Paraskevaidis 2020‐GRC; Yagi 2019‐JPN) (Table).

The case‐control study reported a reduced odds of CIN2 following HPV vaccination (Ikeda 2021‐JPN).

Both cross‐sectional studies reported a reduced risk of CIN2 following HPV vaccination, but with confidence intervals that included no difference (Munro 2017‐GBR; Tozawa‐Ono 2021‐JPN).

All four pre‐post vaccine introduction studies reported a reduced risk of CIN2 comparing time periods before and after HPV vaccine introduction (Benard 2017‐USA; Cuschieri 2023‐GBR; Goodman 2024‐DEU; Thamsborg 2020‐DNK) (Table).

One study reported on the effectiveness of two doses or one dose of HPV vaccine on CIN2 (Palmer 2019‐GBR). While the estimates indicated a reduced odds of CIN2, the confidence intervals included no difference.

##### Vaginal intraepithelial neoplasia (VaIN)

See Table for effect estimates and Table for the risk of bias summary of included studies on VaIN. HPV vaccination may reduce VaIN incidence (low‐certainty evidence; Table).

**25:** Primary clinical outcomes effect estimates: VaIN

**Study**	**Vaccine**	**Population (sex, age at vaccination)**	**Sample size**	**Effect measure (time period)**	**Effect estimate**	**Adjustment factors**	**Notes**
Mix 2022‐USA	Gardasil (Merck quadrivalent)	Female, 15 to 29 years*	945 cases of VaIN	Annual percent change (medium‐term; 2000 vs 2017)	‐9.3% (‐11.5% to ‐7.0%)	Weighted to national population	Pre‐ vs post‐vaccine introduction; *age at outcome
Mix 2022‐USA	Gardasil (Merck quadrivalent)	Female, 30 to 39 years *	945 cases of VaIN	Annual percent change (long‐term; 2000 vs 2017)	‐0.6% (‐4.2% to 3.2%)	Weighted to national population	Pre‐ vs post‐vaccine introduction; *age at outcome

VaIN: vaginal intraepithelial neoplasia

**26:** Risk of bias summary: VaIN

**Study**	**Confounding**	**Selection**	**Classification of interventions**	**Deviations from intended interventions**	**Missing data**	**Measurement of outcomes**	**Selection of reported result**	**Overall risk of bias**
Mix 2022‐USA	Serious	Moderate	Serious	Low	Low	Low	Low	Serious

VaIN: vaginal intraepithelial neoplasia

One study was included that reported on VaIN following HPV vaccination (Mix 2022‐USA). This study had a pre‐post vaccine introduction design and reported a decrease in VaIN in 15‐ to 29‐year‐olds between 2000 and 2017. A smaller decrease was also seen in 30‐ to 39‐year‐olds, but confidence intervals included no difference (Table).

##### Vulval intraepithelial neoplasia (VIN)

See Table for effect estimates and Table for the risk of bias summary of included studies on VIN. We do not know about the effect of HPV vaccine on VIN incidence because the certainty of the evidence is very low (very low‐certainty evidence; Table).

**27:** Primary clinical outcomes effect estimates: VIN

**Study**	**Vaccine**	**Population (sex, age at vaccination)**	**Sample size**	**Effect measure (time period)**	**Effect estimate**	**Adjustment factors**	**Notes**
Mix 2022‐USA	Gardasil (Merck quadrivalent)	Female, 15 to 29 years*	6128 cases of VIN	Annual percent change (medium‐term; 2000 vs 2017)	‐11.7% (‐13.4% to ‐10.0%)	Weighted to national population	Pre‐ vs post‐vaccine introduction; *age at outcome
Mix 2022‐USA	Gardasil (Merck quadrivalent)	Female, 30 to 34 years*	6128 cases of VIN	Annual percent change (long‐term; 2000 vs 2017)	‐0.5% (‐1.3% to 0.4%)	Weighted to national population	Pre‐ vs post‐vaccine introduction; *age at outcome
Rasmussen 2020‐DNK	NR	Female, 12 to 26 years	NR	Annual percentage increase (long‐term; 1997‐1998 vs 2017‐2018)	2.4% (1.8% to 3.0%)	Unadjusted	Pre‐ vs post‐vaccine introduction

NR: not reported; VIN: vulval intraepithelial neoplasia

**28:** Risk of bias summary: VIN

**Study**	**Confounding**	**Selection**	**Classification of interventions**	**Deviations from intended interventions**	**Missing data**	**Measurement of outcomes**	**Selection of reported result**	**Overall risk of bias**
Mix 2022‐USA	Serious	Moderate	Serious	Low	Low	Low	Low	Serious
Rasmussen 2020‐DNK	Critical	Moderate	Serious	Low	Low	Low	Low	Critical

VIN: vulval intraepithelial neoplasia

Two studies were included that reported on VIN following HPV vaccination (Mix 2022‐USA; Rasmussen 2020‐DNK).

Both studies had a pre‐post vaccine introduction design. One reported a decrease in VIN in 15‐ to 29‐year‐olds between 2000 and 2017 (Mix 2022‐USA). A smaller decrease was also seen in 30‐ to 34‐year‐olds, but confidence intervals included no difference. The other study reported an increase in VIN incidence between 1997‐1998 and 2017‐2018 (Rasmussen 2020‐DNK) (Table).

##### Anal intraepithelial neoplasia (AIN)

See Table for effect estimates and Table for the risk of bias summary of included studies on AIN. HPV vaccination may reduce the incidence of AIN (low‐certainty evidence; Table).

**29:** Primary clinical outcomes effect estimates: AIN

**Study**	**Vaccine**	**Population (sex, age at vaccination)**	**Sample size**	**Effect measure (time period)**	**Effect estimate**	**Adjustment factors**	**Notes**
Baandrup 2024‐DNK	Cervarix (GSK bivalent); Gardasil (Merck quadrivalent); Gardasil 9 (Merck nonavalent)	Female, 17 to 32 years	30 cases of AIN	Hazard ratio (long‐term)	0.59 (0.35 to 0.99)	Maximum level of own, mother’s and father’s education	Cohort
Baandrup 2024‐DNK	Cervarix (GSK bivalent); Gardasil (Merck quadrivalent); Gardasil 9 (Merck nonavalent)	Female, < 17 years at vaccination	< 5 cases of AIN	Hazard ratio (long‐term)	0.30 (0.1 to 0.87)	Maximum level of own, mother’s and father’s education	Cohort
Baandrup 2024‐DNK	Cervarix (GSK bivalent); Gardasil (Merck quadrivalent); Gardasil 9 (Merck nonavalent)	Female, 17 to 32 years at vaccination	26 cases of AIN	Hazard ratio (long‐term)	1.21 (0.73 to 2.03)	Maximum level of own, mother’s and father’s education	Cohort
Mix 2022‐USA	Gardasil (Merck quadrivalent)	Female, 15 to 29 years*	462 cases of AIN	Annual percent change (medium‐term; 2000 vs 2017)	7.4% (2.9% to 12.1%)	Weighted to national population	Pre‐ vs post‐vaccine introduction; *age at outcome
Mix 2022‐USA	Gardasil (Merck quadrivalent)	Female, 30 to 39 years*	462 cases of AIN	Annual percent change (long‐term; 2000 vs 2017)	6.3% (4.0% to 8.6%)	Weighted to national population	Pre‐ vs post‐vaccine introduction; *age at outcome
Mix 2022‐USA	Gardasil (Merck quadrivalent)	Male, 15 to 29 years*	2154 cases of AIN	Annual percent change (medium‐term; 2000 vs 2017)	16.7% (10.1% to 23.8%)	Weighted to national population	Pre‐ vs post‐vaccine introduction; *age at outcome
Mix 2022‐USA	Gardasil (Merck quadrivalent)	Male, 30 to 39 years*	2154 cases of AIN	Annual percent change (long‐term; 2000 vs 2017)	3.6% (1.7% to 5.6%)	Weighted to national population	Pre‐ vs post‐vaccine introduction; *age at outcome

AIN: anal intraepithelial neoplasia

**30:** Risk of bias summary: AIN

**Study**	**Confounding**	**Selection**	**Classification of interventions**	**Deviations from intended interventions**	**Missing data**	**Measurement of outcomes**	**Selection of reported result**	**Overall risk of bias**
Baandrup 2024‐DNK	Serious	Low	Low	Low	Low	Low	Low	Serious
Mix 2022‐USA	Serious	Moderate	Serious	Low	Low	Low	Low	Serious

AIN: anal intraepithelial neoplasia

Two studies were included that reported on AIN following the introduction of HPV vaccination (Baandrup 2024‐DNK; Mix 2022‐USA). One cohort study reported a reduced risk of AIN with HPV vaccination in females, with a more pronounced effect in females vaccinated before 17 years of age (Baandrup 2024‐DNK). The other study had a pre‐post vaccine introduction design and reported an increase in AIN incidence in males and females between 2000 and 2017 (Mix 2022‐USA) (Table).

#### Specific adverse events

##### Postural orthostatic tachycardia syndrome (POTS)

See Table for effect estimates and Table for the risk of bias summary of included studies on POTS. HPV vaccination likely does not increase the risk of POTS (moderate‐certainty evidence; Table).

**31:** Specific adverse events effect estimates: postural orthostatic tachycardia syndrome (POTS)

**Study**	**Vaccine**	**Population (sex, age at vaccination)**	**Sample size**	**Effect measure (time period)**	**Effect estimate**	**Adjustment factors**	**Notes**
Skufca 2018‐FIN	Cervarix (GSK bivalent)	Female, 11 to 15 years	Vaccinated: 55,774 person‐years Unvaccinated: 244,171 person‐years	Hazard ratio (short‐term)	1.40 (0.50 to 3.87)	Hospital district, country background, number of any hospital visits or admissions	Cohort
Skufca 2018‐FIN	Cervarix (GSK bivalent)	Female, 11 to 15 years	Vaccinated: 186,946 person‐years Unvaccinated: 244,171 person‐years	Hazard ratio (medium‐term)	0.99 (0.46 to 2.11)	Hospital district, country background, number of any hospital visits or admissions	Cohort
Thomsen 2020‐DNK	Gardasil (Merck quadrivalent)	Female, 11 to 17 years	Vaccinated: 313,880 person‐years Unvaccinated: 313,871 person‐years	Incidence rate ratio (short‐term)	0.54 (0.19 to 1.53)	Age, calendar year of cohort entry, histories of hospital‐diagnosed asthma, diabetes, infections, and mental disorders, number of general practitioner contacts within the past 5 years, previous psychometric tests or talk therapy with a general practitioner, a previous psychologist or psychiatrist visit in primary care, parental education, parental employment status, parental annual income, parental marital status, and parental ethnicity	Cohort
Hviid 2020‐DNK	Gardasil (Merck quadrivalent)	Female, 12 to 27 years	Reference period: 179 cases, 1393 person‐years Risk period: 19 cases, 226 person‐years	Rate ratio (medium‐term)	0.86 (0.48 to 1.54)	Age, season	Self‐controlled case series

**32:** Risk of bias summary: postural orthostatic tachycardia syndrome (POTS)

**Study**	**Confounding**	**Selection**	**Classification of interventions**	**Deviations from intended interventions**	**Missing data**	**Measurement of outcomes**	**Selection of reported result**	**Overall risk of bias**
Skufca 2018‐FIN	Serious	Low	Low	Low	Moderate	Moderate	Moderate	Serious
Thomsen 2020‐DNK	Moderate	Low	Low	Low	Low	Low	Moderate	Moderate

**Study**	**Case definition**	**Case ascertainment independent?**	**Exposure**	**Co‐interventions**	**Observation period defined**	**Risk period defined**	**Comparability**	**Overall**
Hviid 2020‐DNK	Yes, ICD‐10 codes	Not reported	Yes, Danish vaccination register	Unclear	Yes, before and after risk period	Yes, 365 days post vaccine	Yes, adjusted for age and season	Low

ICD‐10: International Statistical Classification of Diseases and Related Health Problems (10th Revision)

Three studies were included that reported on postural orthostatic tachycardia syndrome (POTS) following HPV vaccination (Hviid 2020‐DNK; Skufca 2018‐FIN; Thomsen 2020‐DNK). Two were retrospective cohort studies (Skufca 2018‐FIN; Thomsen 2020‐DNK) and one was a self‐controlled case series analysis (Hviid 2020‐DNK).

Two cohort studies reported on short‐term follow‐up from HPV vaccination and there was no association between HPV vaccination and POTS (RR 0.87, 95% CI 0.34 to 2.22; 2 studies, 927,696 person‐years; I^2^ = 39%) (Skufca 2018‐FIN; Thomsen 2020‐DNK) (Analysis 2.1). One study reported that in a medium‐term follow‐up there was also no association between HPV vaccination and POTS (HR 0.99, 95% CI 0.46 to 2.12; 1 study, 431,117 person‐years) (Skufca 2018‐FIN) (Analysis 2.1). No studies were identified that reported on the association between HPV vaccination and POTS in the long term.

In a self‐controlled case series analysis (Hviid 2020‐DNK), there was no increase in the rate of POTS following HPV vaccination (1 study, 198 cases of POTS; incidence rate ratio (IRR) 0.86, 95% CI 0.48 to 1.54) (Table).

##### Chronic fatigue syndrome/myalgic encephalomyelitis (CFS/ME)

See Table for effect estimates and Table for the risk of bias summary of included studies on CFS/ME. HPV vaccination likely does not increase the risk of CFS/ME (moderate‐certainty evidence; Table).

**33:** Specific adverse events effect estimates: chronic fatigue syndrome/myalgic encephalomyelitis (CFS/ME)

**Study**	**Vaccine**	**Population (sex, age at vaccination)**	**Sample size**	**Effect measure (time period)**	**Effect estimate**	**Adjustment factors**	**Notes**
Thomsen 2020‐DNK	Gardasil (Merck quadrivalent)	Female, 11 to 17 years	Vaccinated: 313,879 Unvaccinated: 313,859	Incidence rate ratio (short‐term)	0.12 (0.02 to 0.99)	Age, calendar year of cohort entry, histories of hospital‐diagnosed asthma, diabetes, infections and mental disorders, number of general practitioner contacts within the past 5 years, previous psychometric tests or talk therapy with a general practitioner, a previous psychologist or psychiatrist visit in primary care, parental education, parental employment status, parental annual income, parental marital status and parental ethnicity	Cohort
Skufca 2018‐FIN	Cervarix (GSK bivalent)	Female, 11 to 15 years	Vaccinated: 55,834 person‐years Unvaccinated: 244,438 person‐years	Hazard ratio (short‐term)	0.61 (0.42 to 0.91)	Hospital district, country background, number of any hospital visits or admissions	Cohort
Skufca 2018‐FIN	Cervarix (GSK bivalent)	Female, 11 to 15 years	Vaccinated: 186,946 person‐years Unvaccinated: 244,171 person‐years	Hazard ratio (medium‐term)	0.75 (0.59 to 0.95)	Hospital district, country background, number of any hospital visits or admissions	Cohort
Feiring 2017‐NOR	Gardasil (Merck quadrivalent)	Female, 11 to 12 years	Vaccinated: 56,334 person‐years Unvaccinated: 74,735 person‐years	Incidence rate ratio (short‐term)	0.97 (0.51 to 1.82)	Unadjusted	Cohort
Feiring 2017‐NOR	Gardasil (Merck quadrivalent)	Female, 11 to 17 years	Vaccinated: 346,717 person‐years Unvaccinated: 156,475 person‐years	Hazard ratio (medium‐term)	0.86 (0.69 to 1.08)	Parental education level, country background, region of residence, and number of previous hospital contacts.	Cohort
Tsai 2023‐TWN	Cervarix (GSK bivalent); Gardasil (Merck quadrivalent); Gardasil 9 (Merck nonavalent)	Female, 12 to 15 years	Vaccinated: 494,296 person‐years Unvaccinated: 2,280,063 person‐years	Standardised incidence ratio (short‐term)	0.33 (‐0.5 to 0.61)	Unadjusted	Cohort
Tsai 2023‐TWN	Cervarix (GSK bivalent); Gardasil (Merck quadrivalent); Gardasil 9 (Merck nonavalent)	Female, 12 to 15 years	Vaccinated: 494,296 person‐years Unvaccinated: 2,280,063 person‐years	Standardised incidence ratio (medium‐term)	1.37 (0.75 to 2.00)	Unadjusted	Cohort
Thomsen 2020‐DNK	Gardasil (Merck quadrivalent)	Female, 11 to 17 years	Reference period: 13 cases Risk period: 11 cases	Incidence rate ratio (medium‐term)	0.82 (0.16 to 4.16)	Unadjusted	Self‐controlled case series
Hviid 2020‐DNK	Gardasil (Merck quadrivalent)	Female, 12 to 27 years	Reference period: 132 cases, 1100 person‐years Risk period: 4 cases, 79 person‐years	Rate ratio (medium‐term)	0.38 (0.13 to 1.09)	Age, season	Self‐controlled case series
Donegan 2013‐GBR	Cervarix (GSK bivalent)	Female, 12 to 18 years	Reference period: NR Risk period: 161 cases	Incidence rate ratio (medium‐term)	1.03 (0.51 to 2.07)	Age, calendar time	Self‐controlled case series
Cameron 2016‐GBR	Cervarix (GSK bivalent); Gardasil (Merck quadrivalent)	Female, 12 to 18 years	Pre‐vaccine: 220,810 Post‐vaccine: 206,323	Incidence rate ratio (long‐term; 2004 vs 2012)	2.68 (0.77 to 11.69)	Unadjusted	Pre‐ vs post‐vaccine introduction
Cameron 2016‐GBR	Cervarix (GSK bivalent); Gardasil (Merck quadrivalent)	Male, 12 to 18 years	Pre‐vaccine: 232,479 Post‐vaccine: 216,880	Incidence rate ratio (long‐term; 2004 vs 2012)	2.14 (0.31 to 23.7)	Unadjusted	Pre‐ vs post‐vaccine introduction
Schurink‐Van't Klooster 2018‐NLD	Cervarix (GSK bivalent)	Female, 12 to 16 years	Pre‐vaccine: 2758 person‐years Post‐vaccine: 57,214 person‐years	Incidence rate ratio (long‐term; 2007‐8 vs 2009‐13)	0.24 (0.03 to 2.09)	Age	Pre‐ vs post‐vaccine introduction

NR: not reported

**34:** Risk of bias summary: chronic fatigue syndrome/myalgic encephalitis (CFS/ME)

**Study**	**Confounding**	**Selection**	**Classification of interventions**	**Deviations from intended interventions**	**Missing data**	**Measurement of outcomes**	**Selection of reported result**	**Overall risk of bias**
Thomsen 2020‐DNK	Moderate	Low	Low	Low	Low	Low	Low	Moderate
Skufca 2018‐FIN	Serious	Low	Low	Low	Moderate	Moderate	Low	Serious
Feiring 2017‐NOR	Moderate	Low	Low	Low	Moderate	Moderate	Low	Moderate
Cameron 2016‐GBR	Critical	Low	Serious	Low	Low	Low	Low	Critical
Schurink‐Van't Klooster 2018‐NLD	Serious	Serious	Serious	Low	Low	Low	Low	Serious
Tsai 2023‐TWN	Serious	Serious	Low	Low	Moderate	Low	Low	Serious
								
**Study**	**Case definition**	**Case ascertainment independent?**	**Exposure**	**Co‐interventions**	**Observation period defined**	**Risk period defined**	**Comparability**	**Overall**
Thomsen 2020‐DNK	Yes, hospital records	Not reported	Yes, national database	Unclear	Yes, before and after risk period	Yes, 365 days post vaccine	Yes, adjusted for age and calendar time	Low
Hviid 2020‐DNK	Yes, ICD‐10 codes	Not reported	Yes, Danish vaccination register	Unclear	Yes, before and after risk period	Yes, 365 days post vaccine	Yes, adjusted for age and season	Low
Donegan 2013‐GBR	Yes, with validation	Not reported	Yes, national statistics	Unclear	Yes, before and after risk period	Yes, 365 days post vaccine	Yes, adjusted for age and calendar time	Low

ICD‐10: International Statistical Classification of Diseases and Related Health Problems (10th Revision)

Eight studies were included that reported on chronic fatigue syndrome/myalgic encephalomyelitis (CFS/ME) following HPV vaccination (Cameron 2016‐GBR; Donegan 2013‐GBR; Feiring 2017‐NOR; Hviid 2020‐DNK; Schurink‐Van't Klooster 2018‐NLD; Skufca 2018‐FIN; Thomsen 2020‐DNK; Tsai 2023‐TWN). Four studies were retrospective cohort studies (Feiring 2017‐NOR; Skufca 2018‐FIN; Thomsen 2020‐DNK; Tsai 2023‐TWN) and three studies reported self‐controlled case series analyses (Donegan 2013‐GBR; Hviid 2020‐DNK; Thomsen 2020‐DNK). Two studies reported on rates of CFS/ME before and after HPV vaccine introduction (Cameron 2016‐GBR; Schurink‐Van't Klooster 2018‐NLD).

In the short term, three cohort studies reported a reduced risk of CFS/ME following HPV vaccination in the short term (RR 0.40, 95% CI 0.22 to 0.75; 3 studies, 3,702,369 person‐years; I^2^ = 67%) (Analysis 2.2) (Skufca 2018‐FIN; Thomsen 2020‐DNK; Tsai 2023‐TWN). In the medium term, three cohort studies indicated no difference in risk of CFS/ME following HPV vaccination (RR 0.96, 95% CI 0.67 to 1.39; 3 studies, 3,708,668 person‐years; I^2^ = 88%) (Analysis 2.2) (Feiring 2017‐NOR; Skufca 2018‐FIN; Tsai 2023‐TWN). No studies were identified that reported on the association between HPV vaccination and CFS/ME in the long term.

In three self‐controlled case series analyses, each reported no increase in the rate of CFS/ME in the weeks following HPV vaccination (RR 0.74, 95% CI 0.40 to 1.39; 3 studies, 321 cases of CFS/ME; I^2^ = 15%) (Analysis 2.3) (Donegan 2013‐GBR; Hviid 2020‐DNK; Thomsen 2020‐DNK).

Two pre‐ versus post‐vaccine introduction studies reported no association between the introduction of HPV vaccination and the risk of CFS/ME (Cameron 2016‐GBR; Schurink‐Van't Klooster 2018‐NLD).

##### Paralysis

See Table for effect estimates and Table for the risk of bias summary of included studies on CFS/ME. HPV vaccination likely does not increase the risk of paralysis (moderate‐certainty evidence; Table).

**35:** Specific adverse events effect estimates: paralysis

**Study**	**Vaccine**	**Population (sex, age at vaccination)**	**Sample size**	**Effect measure (time period)**	**Effect estimate**	**Adjustment factors**	**Notes**
Arnheim‐Dahlström 2013‐DNK/SWE	Gardasil (Merck quadrivalent)	Female, 12 to 17 years	Vaccinated: 229,574 person‐years Unvaccinated: 2,367,206 person‐years	Rate ratio (short‐term)	0.56 (0.35 to 0.90)	Country, age in two‐year intervals, calendar year, and parental country of birth, parental education and paternal socioeconomic status	Cohort
Frisch 2018‐DNK	Gardasil (Merck quadrivalent)	Male, 10 to 17 years	Vaccinated: 24,057 person‐years Unvaccinated: 4,315,133 person‐years	Rate ratio (long‐term)	0.70 (0.17 to 2.80)	Age and calendar year	Cohort
Hviid 2017‐DNK/SWE	Gardasil (Merck quadrivalent)	Female, 18 to 44 years	Vaccinated: 319,298 person‐years Unvaccinated: 16,067,162 person‐years	Rate ratio (short‐term)	0.52 (0.32 to 0.83)	Age, calendar period and country of residence	Cohort
Hviid 2017‐DNK/SWE	Gardasil (Merck quadrivalent)	Female, 18 to 44 years	Vaccinated: 319,298 person‐years Unvaccinated: 16,067,162 person‐years	Rate ratio (medium‐term)	0.42 (0.20 to 0.89)	Age, calendar period and country of residence	Cohort
Hviid 2017‐DNK/SWE	Gardasil (Merck quadrivalent)	Female, 18 to 44 years	Vaccinated: 319,298 person‐years Unvaccinated: 16,067,162 person‐years	Rate ratio (long‐term)	0.61 (0.34 to 1.10)	Age, calendar period and country of residence	Cohort
Skufca 2018‐FIN	Cervarix (GSK bivalent)	Female, 11 to 15 years	Vaccinated: 56,619 person‐years Unvaccinated: 247,695 person‐years	Hazard ratio (short‐term)	0.23 (0.03 to 1.81)	Hospital district, country background and number of any hospital visits or admissions two years before the scheduled vaccination	Cohort
Skufca 2018‐FIN	Cervarix (GSK bivalent)	Female, 11 to 15 years	Vaccinated: 186,946 person‐years Unvaccinated: 244,171 person‐years	Hazard ratio (medium‐term)	0.86 (0.39 to 1.89)	Hospital district, country background and number of any hospital visits or admissions two years before the scheduled vaccination	Cohort
Yoon 2021‐KOR	Cervarix (GSK bivalent); Gardasil (Merck quadrivalent)	Female, 11 to 14 years	Vaccinated: 408,345 person‐years Unvaccinated: 60,626 person‐years	Rate ratio (short‐term)	0.68 (0.22 to 2.11)	Age, region of residence, type of health insurance, income level and anaemia	Cohort
Yoon 2021‐KOR	Cervarix (GSK bivalent)	Female, 11 to 14 years	Vaccinated: 93,203 person‐years Unvaccinated: 60,626 person‐years	Risk ratio (short‐term)	0.45 (0.07 to 2.77)	Age, region of residence, type of health insurance, income level and anaemia	Cohort
Yoon 2021‐KOR	Gardasil (Merck quadrivalent)	Female, 11 to 14 years	Vaccinated: 315,079 person‐years Unvaccinated: 60,626 person‐years	Risk ratio (short‐term)	0.75 (0.24 to 2.39)	Age, region of residence, type of health insurance, income level and anaemia	Cohort
Yoon 2021‐KOR	Cervarix (GSK bivalent); Gardasil (Merck quadrivalent)	Female, 11 to 14 years	Vaccinated: 790,021 person‐years Unvaccinated: 119,946 person‐years	Rate ratio (medium‐term)	0.67 (0.30 to 1.50)	Age, region of residence, type of health insurance, income level and anaemia	Cohort
Yoon 2021‐KOR	Cervarix (GSK bivalent); Gardasil (Merck quadrivalent)	Female, 11 to 14 years	Reference period: 14 cases Risk period: 19 cases	Risk ratio (medium‐term)	0.95 (0.05 to 16.57)	Age of each risk and control interval	Self‐controlled case series

**36:** Risk of bias summary: paralysis

**Study**	**Confounding**	**Selection**	**Classification of interventions**	**Deviations from intended interventions**	**Missing data**	**Measurement of outcomes**	**Selection of reported result**	**Overall risk of bias**
Arnheim‐Dahlström 2013‐DNK/SWE	Moderate	Low	Low	Low	Low	Low	Low	Moderate
Frisch 2018‐DNK	Serious	Low	Low	Low	Low	Low	Low	Serious
Hviid 2017‐DNK/SWE	Serious	Low	Low	Low	Low	Low	Low	Serious
Skufca 2018‐FIN	Serious	Low	Low	Low	Moderate	Moderate	Low	Serious
Yoon 2021‐KOR	Serious	Low	Low	Low	Low	Low	Low	Serious
								
**Study**	**Case definition**	**Case ascertainment independent?**	**Exposure**	**Co‐interventions**	**Observation period defined**	**Risk period defined**	**Comparability**	**Overall**
Yoon 2021‐KOR	Yes, national database	Not reported	Yes, national database	Unclear	Yes, 466‐730 days post vaccine	Yes, 365 days post vaccine	Yes, adjusted for age	Low

Five studies were included that reported on paralysis following HPV vaccination (Arnheim‐Dahlström 2013‐DNK/SWE; Frisch 2018‐DNK; Hviid 2017‐DNK/SWE; Skufca 2018‐FIN; Yoon 2021‐KOR). All five studies were retrospective cohort studies. One study also reported a self‐controlled case series analysis (Yoon 2021‐KOR).

In the short term, four cohort studies reported fewer cases of paralysis following HPV vaccination than no vaccine (RR 0.54, 95% CI 0.39 to 0.74; 4 studies, 19.8 million person‐years; I^2^ = 0%) (Analysis 2.4) (Arnheim‐Dahlström 2013‐DNK/SWE; Hviid 2017‐DNK/SWE; Skufca 2018‐FIN; Yoon 2021‐KOR). In the medium term, three studies also reported fewer cases of paralysis following HPV vaccination than no vaccine (RR 0.61, 95% CI 0.39 to 0.96; 3 studies, 17.7 million person‐years; I^2^ = 0%) (Hviid 2017‐DNK/SWE; Skufca 2018‐FIN; Yoon 2021‐KOR). In the long term, two studies reported no association between HPV vaccination and paralysis (RR 0.62, 95% CI 0.36 to 1.07; 2 studies, 20.7 million person‐years; I^2^ = 0%) (Analysis 2.4) (Frisch 2018‐DNK; Hviid 2017‐DNK/SWE).

In a self‐controlled case series analysis (Yoon 2021‐KOR), there was no increased risk of paralysis following HPV vaccination (1 study, 33 cases of paralysis; RR 0.95, 95% CI 0.05 to 16.57) (Table).

##### Complex regional pain syndrome (CRPS)

See Table for effect estimates and Table for the risk of bias summary of included studies on CRPS. HPV vaccination likely does not increase the risk of CRPS (moderate‐certainty evidence; Table).

**37:** Specific adverse events effect estimates: complex regional pain syndrome (CRPS)

**Study**	**Vaccine**	**Population (sex, age at vaccination)**	**Sample size**	**Effect measure (time period)**	**Effect estimate**	**Adjustment factors**	**Notes**
Skufca 2018‐FIN	Cervarix (GSK bivalent)	Female, 11 to 15 years	Vaccinated: 55,770 person‐years Unvaccinated: 244,158 person‐years	Hazard ratio (short‐term)	0.00 (0.00 to 0.00)	Hospital district, country background, number of hospital visits or admissions two years before vaccination	Cohort; no cases
Skufca 2018‐FIN	Cervarix (GSK bivalent)	Female, 11 to 15 years	Vaccinated: 186,946 person‐years Unvaccinated: 244,171 person‐years	Hazard ratio (medium‐term)	0.34 (0.11 to 1.05)	Hospital district, country background, number of hospital visits or admissions two years before vaccination	Cohort
Tsai 2023‐TWN	Cervarix (GSK bivalent); Gardasil (Merck quadrivalent); Gardasil 9 (Merck nonavalent)	Female, 12 to 15 years	Vaccinated: 494,660 person‐years Unvaccinated: 2,280,373 person‐years	Standardised incidence ratio (short‐term)	0.40 (‐0.73 to 1.54)	Unadjusted	Cohort
Tsai 2023‐TWN	Cervarix (GSK bivalent); Gardasil (Merck quadrivalent); Gardasil 9 (Merck nonavalent)	Female, 12 to 15 years	Vaccinated: 494,660 person‐years Unvaccinated: 2,280,373 person‐years	Standardised incidence ratio (medium‐term)	0.60 (‐0.82 to 2.02)	Unadjusted	Cohort
Vielot 2020‐USA	Cervarix (GSK bivalent); Gardasil (Merck quadrivalent)	Female, 11 to 12 years	Vaccinated: 76,423 Unvaccinated: 47,558	Hazard ratio (immediate‐term)	0.90 (0.46 to 1.73)	Physical trauma, infection, mental illness and use of primary care	Cohort
Vielot 2020‐USA	Cervarix (GSK bivalent); Gardasil (Merck quadrivalent)	Female, 11 to 12 years	Vaccinated: 76,423 Unvaccinated: 47,558	Hazard ratio (short‐term)	1.11 (0.83 to 1.47)	Physical trauma, infection, mental illness and use of primary care	Cohort
Vielot 2020‐USA	Cervarix (GSK bivalent); Gardasil (Merck quadrivalent)	Female, 11 to 12 years	Vaccinated: 76,423 Unvaccinated: 47,558	Hazard ratio (long‐term)	0.76 (0.62 to 0.94)	Physical trauma, infection, mental illness and use of primary care	Cohort
Hviid 2020‐DNK	Gardasil (Merck quadrivalent)	Female, 12 to 27 years	Reference period: 486 cases Risk period: 49 cases	Rate ratio (short‐term)	1.31 (0.91 to 1.90)	Age, season	Self‐controlled case series

**38:** Risk of bias summary: complex regional pain syndrome (CRPS)

**Study**	**Confounding**	**Selection**	**Classification of interventions**	**Deviations from intended interventions**	**Missing data**	**Measurement of outcomes**	**Selection of reported result**	**Overall risk of bias**
Skufca 2018‐FIN	Serious	Low	Low	Low	Moderate	Moderate	Low	Serious
Tsai 2023‐TWN	Serious	Serious	Low	Low	Moderate	Low	Low	Serious
Vielot 2020‐USA	Serious	Low	Moderate	Low	Low	Moderate	Low	Serious
								
**Study**	**Case definition**	**Case ascertainment independent?**	**Exposure**	**Co‐interventions**	**Observation period defined**	**Risk period defined**	**Comparability**	**Overall**
Hviid 2020‐DNK	Yes, ICD‐10 codes	Not reported	Yes, Danish vaccination register	Unclear	Yes, before and after risk period	Yes, 365 days post vaccine	Yes, adjusted for age and season	Low

ICD‐10: International Statistical Classification of Diseases and Related Health Problems (10th Revision)

Four studies were included that reported on CRPS following HPV vaccination (Hviid 2020‐DNK; Skufca 2018‐FIN; Tsai 2023‐TWN; Vielot 2020‐USA). Three studies were retrospective cohort studies (Skufca 2018‐FIN; Tsai 2023‐TWN; Vielot 2020‐USA) and the third was a self‐controlled case series (Hviid 2020‐DNK).

In the immediate term (RR 0.90, 95% CI 0.46 to 1.75; 1 study, 123,981 females) to short term, there was no association between HPV vaccination and CRPS (RR 0.95, 95% CI 0.46 to 1.96; 2 studies, 123,981 females plus 2,775,033 person‐years) (Analysis 2.5). In the medium term, two studies reported no association between HPV vaccination and CRPS (RR 0.43, 95% CI 0.18 to 1.03; 2 studies, 3,206,150 person‐years) (Skufca 2018‐FIN; Tsai 2023‐TWN). In the long term, one study suggested that there was a reduced hazard of CRPS following HPV vaccination (HR 0.76, 95% CI 0.62 to 0.94; 1 study, 123,981 females) (Analysis 2.5) (Vielot 2020‐USA).

In a self‐controlled case series analysis, there was no increase in the rate of CRPS following HPV vaccination (1 study, 535 cases of CRPS; IRR 1.31, 95% CI 0.91 to 1.90) (Hviid 2020‐DNK).

##### Guillain‐Barré syndrome

See Table for effect estimates and Table for the risk of bias summary of included studies on Guillain‐Barré syndrome. The evidence suggests that HPV vaccination does not increase the risk of Guillain‐Barré syndrome (low‐certainty evidence; Table).

**39:** Specific adverse events effect estimates: Guillain‐Barré syndrome (GBS)

**Study**	**Vaccine**	**Population (sex, age at vaccination)**	**Sample size**	**Effect measure (time period)**	**Effect estimate**	**Adjustment factors**	**Notes**
Arnheim‐Dahlström 2013‐DNK/SWE	Gardasil (Merck quadrivalent)	Female, 12 to 17 years	Vaccinated: 296,826 Unvaccinated: 700,759	Not estimable	‐	‐	Cohort; no cases in exposed group
Deceuninck 2018‐CAN	Gardasil (Merck quadrivalent)	Female and male, 9 to 17 years	Vaccinated: 558,995 Unvaccinated: 13,736,169	Risk ratio (long‐term)	0.81 (0.29 to 2.26)	Sex, age, year of GBS diagnosis and H1N1 pandemic period	Cohort
Gronlund 2016‐SWE	Gardasil (Merck quadrivalent)	Female, 10 to 30 years*	Vaccinated: 7848 person‐years Unvaccinated: 245,807 person‐years	Not estimable	‐	‐	Cohort; no cases in vaccinated group; *age at outcome
Hviid 2017‐DNK/SWE	Gardasil (Merck quadrivalent)	Female, 18 to 44 years*	Vaccinated: 319,298 person‐years Unvaccinated: 16,067,162 person‐years	Not estimable	‐	‐	Cohort; no cases in vaccinated group; *age at outcome
Martin‐Merino 2021‐ESP	NR	Female, 9 to 28 years*	Vaccinated: 381,377 person‐years Unvaccinated: 1,029,655 person‐years	Hazard ratio (long‐term)	1.24 (0.19 to 8.00)	Region and antibiotic prescription	Cohort; *age at outcome
Miranda 2017‐FRA	Cervarix (GSK bivalent); Gardasil (Merck quadrivalent)	Female, 13 to 16 years	Vaccinated: 678,765 person‐years Unvaccinated: 4,746,753 person‐years	Hazard ratio (short‐term)	3.94 (1.58 to 9.78)	Age, year of inclusion, geographical zone, CMUc, history of use of health care and other vaccinations, use of health care and other vaccinations after inclusion	Cohort
Miranda 2017‐FRA	Cervarix (GSK bivalent); Gardasil (Merck quadrivalent)	Female, 13 to 16 years	Vaccinated: 1,393,228 person‐years Unvaccinated: 4,746,753 person‐years	Hazard ratio (medium‐term)	3.78 (1.79 to 7.98)	Age, year of inclusion, geographical zone, CMUc, history of use of health care and other vaccinations, use of health care and other vaccinations after inclusion	Cohort
Miranda 2017‐FRA	Gardasil (Merck quadrivalent)	Female, 13 to 16 years	Vaccinated: 1,323,942 person‐years Unvaccinated: 4,746,753 person‐years	Hazard ratio (medium‐term)	3.78 (1.70 to 8.41)	Age, year of inclusion, geographical zone, CMUc, history of use of health care and other vaccinations, use of health care and other vaccinations after inclusion	Cohort
Miranda 2017‐FRA	Cervarix (GSK bivalent)	Female, 13 to 16 years	Vaccinated: 69,286 person‐years Unvaccinated: 4,746,753 person‐years	Hazard ratio (medium‐term)	8.08 (1.69 to 38.61)	Age, year of inclusion, geographical zone, CMUc, history of use of health care and other vaccinations, use of health care and other vaccinations after inclusion	Cohort
Skufca 2018‐FIN	Cervarix (GSK bivalent)	Female, 11 to 15 years	Vaccinated: 55,770 person‐years Unvaccinated: 244,141 person‐years	Hazard ratio (short‐term)	2.76 (0.24 to 32.04)	Hospital district, country background and number of any hospital visits or admissions two years before the scheduled vaccination	Cohort
Skufca 2018‐FIN	Cervarix (GSK bivalent)	Female, 11 to 15 years	Vaccinated: 186,946 person‐years Unvaccinated: 244,171 person‐years	Hazard ratio (medium‐term)	5.31 (0.62 to 45.39)	Hospital district, country background and number of any hospital visits or admissions two years before the scheduled vaccination	Cohort
Tsai 2023‐TWN	Cervarix (GSK bivalent); Gardasil (Merck quadrivalent); Gardasil 9 (Merck nonavalent)	Female, 12 to 15 years	V: 494,678 person‐years C: 2,280,368 person years	Standardised incidence ratio (short‐term)	0.21 (‐0.61 to 1.03)	Unadjusted	Cohort
Tsai 2023‐TWN	Cervarix (GSK bivalent); Gardasil (Merck quadrivalent); Gardasil 9 (Merck nonavalent)	Female, 12 to 15 years	V: 494,678 person‐years C: 2,280,368 person‐years	Standardised incidence ratio (medium‐term)	2.10 (‐0.97 to 5.17)	Unadjusted	Cohort
Willame 2016‐GBR	Cervarix (GSK bivalent)	Female, 9 to 24 years	Vaccinated: 64,705 person‐years Unvaccinated: 64,841 person‐years	Not estimable	‐	‐	Cohort; no cases in vaccinated group
Yoon 2021‐KOR	Cervarix (GSK bivalent); Gardasil (Merck quadrivalent)	Female, 11 to 14 years	Vaccinated: 408,363 person‐years Unvaccinated: 60,626 person‐years	Rate ratio (short‐term)	0.13 (0.03 to 0.53)	Age, region of residence, type of health insurance, income level and anaemia	Cohort
Yoon 2021‐KOR	Cervarix (GSK bivalent)	Female, 11 to 14 years	Vaccinated: 93,272 person‐years Unvaccinated: 60,626 person‐years	Not estimable	‐	‐	Cohort; no cases in vaccinated group
Yoon 2021‐KOR	Gardasil (Merck quadrivalent)	Female, 11 to 14 years	Vaccinated: 315,090 person‐years Unvaccinated: 60,626 person‐years	Risk ratio (short‐term)	0.17 (0.04 to 0.69)	Age, region of residence, type of health insurance, income level and anaemia	Cohort
Yoon 2021‐KOR	Cervarix (GSK bivalent); Gardasil (Merck quadrivalent)	Female, 11 to 14 years	Vaccinated: 790,069 person‐years Unvaccinated: 119,949 person‐years	Rate ratio (medium‐term)	0.19 (0.07 to 0.55)	Age, region of residence, type of health insurance, income level and anaemia	Cohort
Grimaldi‐Bensouda 2017‐FRA	Cervarix (GSK bivalent); Gardasil (Merck quadrivalent)	Female, 11 to 25 years*	Cases: 13 (0 vaccinated) Controls: 130 (2 vaccinated)	Not estimable	‐	‐	Case‐control; no cases exposed to vaccine; *age at outcome
Andrews 2017‐GBR	Cervarix (GSK bivalent)	Female, 12 to 18 years	Reference period: 86 cases Risk period: 5 cases	Relative incidence (short‐term)	0.84 (0.30 to 2.34)	Age in years, period and season	Self‐controlled case series
Andrews 2017‐GBR	Gardasil (Merck quadrivalent)	Female, 12 to 18 years	Reference period: 15 cases Risk period: 4 cases	Relative incidence (short‐term)	1.61 (0.39 to 6.64)	Age in years, period and season	Self‐controlled case series
Andrews 2017‐GBR	Cervarix (GSK bivalent); Gardasil (Merck quadrivalent)	Female, 12 to 18 years	Reference period: 101 cases Risk period: 9 cases	Relative incidence (immediate‐term)	1.04 (0.47 to 2.28)	Age in years, period and season	Self‐controlled case series
Andrews 2017‐GBR	Cervarix (GSK bivalent); Gardasil (Merck quadrivalent)	Female, 12 to 18 years	Reference period: 101 cases Risk period: 24 cases	Relative incidence (short‐term)	1.10 (0.57 to 2.14)	Age in years, period and season	Self‐controlled case series
Miranda 2017‐FRA	Cervarix (GSK bivalent); Gardasil (Merck quadrivalent)	Female, 13 to 16 years	Reference period: 37 cases Risk period: 6 cases	Incidence rate ratio (immediate‐term)	3.83 (1.67 to 8.75)	Age, A(H1N1) pandemics period and winter season Known for gastroenteritis/influenza‐like epidemics in France	Self‐controlled case series; 42 days
Miranda 2017‐FRA	Cervarix (GSK bivalent); Gardasil (Merck quadrivalent)	Female, 13 to 16 years	Reference period: 32 cases Risk period: 11 cases	Incidence rate ratio (short‐term)	2.39 (1.21 to 4.72)	Age, A(H1N1) pandemics period and winter season known for gastroenteritis/influenza‐like epidemics in France	Self‐controlled case series; 6 months
Yoon 2021‐KOR	Cervarix (GSK bivalent); Gardasil (Merck quadrivalent)	Female, 11 to 14 years	Reference period: 7 cases Risk period: 5 cases	Relative risk (short‐term)	0.47 (0.02 to 9.36)	Age of each risk and control interval	Self‐controlled case series
Cameron 2016‐GBR	Cervarix (GSK bivalent); Gardasil (Merck quadrivalent)	Female, 12 to 18 years	Pre‐vaccine: 220,810 Post‐vaccine: 206,323	Incidence rate ratio (long‐term; 2004 vs 2012)	3.21 (0.13 to 78.8)	Unadjusted	Pre‐ vs post‐vaccine introduction
Cameron 2016‐GBR	Cervarix (GSK bivalent); Gardasil (Merck quadrivalent)	Male, 12 to 18 years	Pre‐vaccine: 232,479 Post‐vaccine: 216,880	Incidence rate ratio (long‐term; 2004 vs 2012)	1.07 (0.15 to 7.61)	Unadjusted	Pre‐ vs post‐vaccine introduction

A(H1N1): influenza A virus subtype H1N1; CMUc: complementary Universal Health Insurance; GBS: Guillain‐Barré syndrome; NR: not reported

**40:** Risk of bias summary: Guillain‐Barre Syndrome (GBS)

**Study**	**Confounding**	**Selection**	**Classification of interventions**	**Deviations from intended interventions**	**Missing data**	**Measurement of outcomes**	**Selection of reported result**	**Overall risk of bias**
Arnheim‐Dahlström 2013‐DNK/SWE	Critical	Low	Low	Low	Low	Low	Low	Critical
Deceuninck 2018‐CAN	Serious	Low	Low	Low	Low	Low	Low	Serious
Gronlund 2016‐SWE	Critical	Low	Low	Low	Low	Low	Low	Critical
Hviid 2017‐DNK/SWE	Serious	Low	Low	Low	Low	Low	Low	Serious
Martin‐Merino 2021‐ESP	Serious	Low	Low	Low	Low	Low	Low	Serious
Miranda 2017‐FRA	Serious	Low	Low	Low	Low	Low	Low	Serious
Skufca 2018‐FIN	Serious	Low	Low	Low	Moderate	Moderate	Low	Serious
Willame 2016‐GBR	Critical	Low	Low	Low	Low	Moderate	Low	Critical
Yoon 2021‐KOR	Serious	Low	Low	Low	Low	Low	Low	Serious
Grimaldi‐Bensouda 2017‐FRA	Critical	Moderate	Low	Low	Low	Moderate	Low	Critical
Cameron 2016‐GBR	Critical	Low	Serious	Low	Low	Low	Low	Critical
Tsai 2023‐TWN	Serious	Serious	Low	Low	Moderate	Low	Low	Serious
								
**Study**	**Case definition**	**Case ascertainment independent?**	**Exposure**	**Co‐interventions**	**Observation period defined**	**Risk period defined**	**Comparability**	**Overall**
Andrews 2017‐GBR	Yes, hospital records	Not reported	Yes, GP records	Unclear	Yes, before and after risk period	Yes, 91 days post vaccine	Yes, adjusted for age and calendar time	Low
Miranda 2017‐FRA	Yes, insurance database	Not reported	Yes, insurance database	Unclear	No, limited methods reported	Yes, 42 days to 6 months post vaccine	Yes, adjusted for season and calendar time	Moderate
Yoon 2021‐KOR	Yes, national database	Not reported	Yes, national database	Unclear	Yes, 466 to 730 days post vaccine	Yes, 365 days post vaccine	Yes, adjusted for age	Low

GP: general practitioner

Thirteen studies were included that reported on Guillain‐Barré syndrome following HPV vaccination (Andrews 2017‐GBR; Arnheim‐Dahlström 2013‐DNK/SWE; Cameron 2016‐GBR; Deceuninck 2018‐CAN; Grimaldi‐Bensouda 2017‐FRA; Gronlund 2016‐SWE; Hviid 2017‐DNK/SWE; Martin‐Merino 2021‐ESP; Miranda 2017‐FRA; Skufca 2018‐FIN; Tsai 2023‐TWN; Willame 2016‐GBR; Yoon 2021‐KOR). One study was a case‐control study (Grimaldi‐Bensouda 2017‐FRA), three were self‐controlled case series (Andrews 2017‐GBR; Miranda 2017‐FRA; Yoon 2021‐KOR), one reported pre‐ and post‐vaccine introduction rates (Cameron 2016‐GBR), and seven were cohort studies.

Four cohort studies each reported no cases of Guillain‐Barré syndrome in those exposed to HPV vaccination (Arnheim‐Dahlström 2013‐DNK/SWE; Gronlund 2016‐SWE; Hviid 2017‐DNK/SWE; Willame 2016‐GBR). In the short term, four cohort studies reported inconsistent results (Miranda 2017‐FRA; Skufca 2018‐FIN; Tsai 2023‐TWN; Yoon 2021‐KOR). One study from France reported a higher incidence of Guillain‐Barré syndrome following exposure to HPV vaccine (Miranda 2017‐FRA), while two studies reported no association (Skufca 2018‐FIN; Tsai 2023‐TWN) and a third study reported a negative association between HPV vaccine and Guillain‐Barré syndrome in the short term (Yoon 2021‐KOR). The pooled estimate indicated no difference between HPV vaccine and no vaccine in risk of Guillain‐Barré syndrome (RR 0.78, 95% CI 0.10 to 6.03; 4 studies, 8.2 million person‐years; I^2^ = 83%) (Analysis 2.6).

In the medium term, four studies again reported inconsistent effects of HPV vaccination on Guillain‐Barré syndrome (RR 1.56, 95% CI 0.40 to 5.99; 4 studies, 9.5 million person‐years; I^2^ = 87%) (Analysis 2.6) (Miranda 2017‐FRA; Skufca 2018‐FIN; Tsai 2023‐TWN; Yoon 2021‐KOR).

In the long term, two studies indicated no difference between HPV vaccine and no vaccine in rates of Guillain‐Barré syndrome (RR 0.89, 95% CI 0.36 to 2.20; 2 studies, 15.7 million person‐years; I^2^ = 0%) (Analysis 2.6) (Deceuninck 2018‐CAN; Martin‐Merino 2021‐ESP).

Using a self‐controlled case series analysis, two studies reported no increased risk of Guillain‐Barré syndrome following HPV vaccination in the immediate term (RR 1.98, 95% CI 0.55 to 7.12; 2 studies, 153 cases; I^2^ = 80%) (Analysis 2.7) (Andrews 2017‐GBR; Miranda 2017‐FRA). In the short term, three studies reported no increased risk of Guillain‐Barré syndrome following HPV vaccination (RR 1.53, 95% CI 0.78 to 2.98; 3 studies, 180 cases; I^2^ = 37%) (Analysis 2.7)(Andrews 2017‐GBR; Miranda 2017‐FRA; Yoon 2021‐KOR).

One pre‐ versus post‐vaccine introduction study evaluated 12‐ to 18‐year‐old boys and girls from Great Britain (Cameron 2016‐GBR). There was no increase in the rates of Guillain‐Barré syndrome following the introduction of the HPV vaccine.

One case‐control study evaluated 11‐ to 25‐year‐old females (Grimaldi‐Bensouda 2017‐FRA). There were no cases of Guillain‐Barré syndrome in those exposed to HPV vaccine in this study.

##### Premature ovarian failure

See Table for effect estimates and Table for the risk of bias summary of included studies on premature ovarian failure. The evidence suggests that HPV vaccination does not increase the risk of premature ovarian failure (low‐certainty evidence; Table).

**41:** Specific adverse events effect estimates: premature ovarian failure

**Study**	**Vaccine**	**Population (sex, age at vaccination)**	**Sample size**	**Effect measure (time period)**	**Effect estimate**	**Adjustment factors**	**Notes**
Hviid 2021‐DNK	Gardasil (Merck quadrivalent)	Female, 11 to 34 years	Vaccinated: 505,829 Unvaccinated: 490,471	Hazard ratio (long‐term)	0.96 (0.55 to 1.68)	Calendar year, propensity score	Cohort
Hviid 2021‐DNK	Gardasil (Merck quadrivalent)	Female, vaccinated < 20 years old	Vaccinated: 333,505 Unvaccinated: 490,471	Hazard ratio (long‐term)	0.77 (0.37 to 1.62)	Calendar year, propensity score	Cohort
Hviid 2021‐DNK	Gardasil (Merck quadrivalent)	Female, vaccinated ≥ 20 years old	Vaccinated: 505,829 Unvaccinated: 172,324	Hazard ratio (long‐term)	1.15 (0.58 to 2.28)	Calendar year, propensity score	Cohort
Ter‐Minasyan 2024‐ARM	Gardasil (Merck quadrivalent)	Female, 15 to 24 years	Vaccinated: 39 Unvaccinated: 30	Odds ratio (short‐term)	0.76 (0.05 to 12.72)	Unadjusted	Cohort
Ter‐Minasyan 2024‐ARM	Gardasil (Merck quadrivalent)	Female, 25 to 34 years	Vaccinated: 36 Unvaccinated: 30	Odds ratio (short‐term)	0.83 (0.05 to 13.84)	Unadjusted	Cohort
Ter‐Minasyan 2024‐ARM	Gardasil (Merck quadrivalent)	Female, 35 to 40 years	Vaccinated: 23 Unvaccinated: 30	Odds ratio (short‐term)	0.42 (0.02 to 10.75)	Unadjusted	Cohort
Tsai 2023‐TWN	Cervarix (GSK bivalent); Gardasil (Merck quadrivalent); Gardasil 9 (Merck nonavalent)	Female, 12 to 15 years	Vaccinated: 494,684 person‐years Unvaccinated: 2,280,280 person‐years	Standardised incidence ratio (short‐term)	0.27 (‐0.21 to 0.74)	Unadjusted	Cohort
Tsai 2023‐TWN	Cervarix (GSK bivalent); Gardasil (Merck quadrivalent); Gardasil 9 (Merck nonavalent)	Female, 12 to 15 years	Vaccinated: 494,684 person‐years Unvaccinated: 2,280,280 person‐years	Standardised incidence ratio (medium‐term)	0.91 (‐0.02 to 1.84)	Unadjusted	Cohort

**42:** Risk of bias summary: premature ovarian failure

**Study**	**Confounding**	**Selection**	**Classification of interventions**	**Deviations from intended interventions**	**Missing data**	**Measurement of outcomes**	**Selection of reported result**	**Overall risk of bias**
Hviid 2021‐DNK	Moderate	Moderate	Low	Low	Low	Low	Low	Moderate
Ter‐Minasyan 2024‐ARM	Critical	Moderate	Serious	Low	Low	Serious	Moderate	Critical
Tsai 2023‐TWN	Critical	Low	Low	Low	Low	Low	Moderate	Critical

Three retrospective cohort studies were included that reported on premature ovarian failure following HPV vaccination (Hviid 2021‐DNK; Ter‐Minasyan 2024‐ARM; Tsai 2023‐TWN).

Across the short term (RR 0.21, 95% CI 0.03 to 1.28; 2 studies, 128 females plus 2,774,964 person‐years; I^2^ = 29%), medium term (RR 0.91, 95% CI 0.55 to 1.51) and long term (RR 0.96, 95% CI 0.55 to 1.68) follow‐ups after HPV vaccination there was no association with premature ovarian failure (Analysis 2.8) (Table).

##### Infertility

See Table for effect estimates and Table for the risk of bias summary of included studies on infertility. HPV vaccination likely does not increase the risk of infertility (moderate‐certainty evidence; Table).

**43:** Specific adverse events effect estimates: infertility

**Study**	**Vaccine**	**Population (sex, age)**	**Sample size**	**Effect measure (time period)**	**Effect estimate**	**Adjustment factors**	**Notes**
McInerney 2017‐USA	Gardasil (Merck quadrivalent)	Female, 25 to 32 years*	Vaccinated: 4932 Unvaccinated: 10332	Fecundability ratio (long‐term)	0.98 (0.90 to 1.08)	Age at baseline, education, income, geographic region of residence, race/ethnicity, history of smoking, abnormal Pap test before age at vaccination and parent’s education	Cohort; *age at outcome
McInerney 2017‐USA	Gardasil (Merck quadrivalent)	Female, < 18 years	Vaccinated: 1094 Unvaccinated: 10332	Fecundability ratio (long‐term)	1.00 (0.85 to 1.17)	Age at baseline, education, income, geographic region of residence, race/ethnicity, history of smoking, abnormal Pap test before age at vaccination and parent’s education	Cohort
McInerney 2017‐USA	Gardasil (Merck quadrivalent)	Female, ≥ 18 years	Vaccinated: 3842 Unvaccinated: 10332	Fecundability ratio (long‐term)	0.98 (0.89 to 1.08)	Age at baseline, education, income, geographic region of residence, race/ethnicity, history of smoking, abnormal Pap test before age at vaccination and parent’s education	Cohort
McInerney 2017‐USA	Gardasil (Merck quadrivalent)	Male, 25 to 32 years*	Vaccinated: 211 Unvaccinated: 4177	Fecundability ratio (long‐term)	1.07 (0.79 to 1.46)	Age at baseline, education, income, geographic region of residence, race/ethnicity, history of smoking	Cohort; *age at outcome
McInerney 2017‐USA	Gardasil (Merck quadrivalent)	Male, < 18 years old	Vaccinated: 48 Unvaccinated: 4177	Fecundability ratio (long‐term)	1.10 (0.56 to 2.19)	Age at baseline, education, income, geographic region of residence, race/ethnicity, history of smoking	Cohort
McInerney 2017‐USA	Gardasil (Merck quadrivalent)	Male, ≥ 18 years	Vaccinated: 163 Unvaccinated: 4177	Fecundability ratio (long‐term)	1.06 (0.75 to 1.50)	Age at baseline, education, income, geographic region of residence, race/ethnicity, history of smoking	Cohort
Schmuhl 2020‐USA	NR	Female, < 18 years old	NR	Odds ratio (long‐term)	1.04 (0.22 to 4.97)	Body mass index, ever using birth control pills, any history of STI, health insurance status, routine access to health care, age, race/ethnicity, marriage, education and income	Cross‐sectional
Schmuhl 2020‐USA	NR	Female, ≥ 18 years	NR	Odds ratio (long‐term)	0.42 (0.11 to 1.54)	Body mass index, ever using birth control pills, any history of STI, health insurance status, routine access to health care, age, race/ethnicity, marriage, education and income	Cross‐sectional

NR: not reported; STI: sexually transmitted infection

**44:** Risk of bias summary: infertility

**Study**	**Confounding**	**Selection**	**Classification of interventions**	**Deviations from intended interventions**	**Missing data**	**Measurement of outcomes**	**Selection of reported result**	**Overall risk of bias**
McInerney 2017‐USA	Moderate	Low	Moderate	Low	Serious	Moderate	Low	Serious
Schmuhl 2020‐USA	Serious	Low	Moderate	Low	Moderate	Moderate	Low	Serious

Two studies were included that reported on infertility (not specified whether primary or secondary infertility) following HPV vaccination (McInerney 2017‐USA; Schmuhl 2020‐USA). One study was a retrospective cohort study (McInerney 2017‐USA) and the other was a cross‐sectional study (Schmuhl 2020‐USA).

The cohort study reported on fecundability (total number of pregnancies/total number of cycles) in 25‐ to 32‐year‐old women and their male partners in the USA (McInerney 2017‐USA). There was no association between HPV vaccine and fecundability in females receiving HPV vaccine before the age of 18 (fecundability ratio (FR) 1.0, 95% CI 0.85 to 1.17) or after the age of 18 (FR 0.98, 95% CI 0.89 to 1.08). For males, there was also no association between fecundability and those receiving HPV vaccine before 18 years of age (FR 1.1, 95% CI 0.56 to 2.19) or after 18 years of age (FR 1.06, 95% CI 0.75 to 1.50) (Table).

One study evaluated self‐reported infertility (not specified whether primary or secondary infertility) in 18‐ to 33‐year‐old women in the USA (Schmuhl 2020‐USA). There was no association between infertility and receiving HPV vaccine before the age of 18 (OR 1.04, 95% CI 0.22 to 4.97) or after the age of 18 (OR 0.42, 95% CI 0.11 to 1.54).

##### Sexual activity (measured by incidence of sexually transmitted infections)

See Table for effect estimates and Table for the risk of bias summary of included studies on sexual activity. HPV vaccination likely does not increase sexual activity (moderate‐certainty evidence; Table).

**45:** Specific adverse events effect estimates: sexual activity (measured by incidence of sexually transmitted infections)

**Study**	**Vaccine**	**Population (sex, age)**	**Sample size**	**Effect measure (time period)**	**Effect estimate**	**Adjustment factors**	**Notes**
Bednarczyk 2012‐USA	Gardasil (Merck quadrivalent)	Female, 11 to 12 years	Vaccinated: 493 Unvaccinated: 905	Incidence rate ratio (medium‐term)	0.68 (0.06 to 7.71)	Health care‐seeking behaviour in the previous year, age at vaccination, race and socioeconomic status	Cohort; chlamydia infection
Bednarczyk 2012‐USA	Gardasil (Merck quadrivalent)	Female, 11 to 12 years	Vaccinated: 493 Unvaccinated: 905	Incidence rate ratio (medium‐term)	0.90 (0.09 to 9.07)	Health care‐seeking behaviour in the previous year, age at vaccination, race and socioeconomic status	Cohort; venereal disease, unspecified
Cummings 2012‐USA	Gardasil (Merck quadrivalent)	Female, 14 to 17 years	Vaccinated: 75 Unvaccinated: 150	Odds ratio (medium‐term)	0.9 (0.04 to 2.2)	Matched with two historical controls by age at enrolment, clinic site and reported sexual activity	Cohort; chlamydia infection
Cummings 2012‐USA	Gardasil (Merck quadrivalent)	Female, 14 to 17 years	Vaccinated: 75 Unvaccinated: 150	Odds ratio (medium‐term)	‐	‐	Cohort; gonorrhoea (not estimable because no cases in vaccinated cohort)
Cummings 2012‐USA	Gardasil (Merck quadrivalent)	Female, 14 to 17 years	Vaccinated: 75 Unvaccinated: 150	Odds ratio (medium‐term)	5.3 (0.7 to 42.3)	Matched with two historical controls by age at enrolment, clinic site and reported sexual activity	Cohort; trichomonas
Sadler 2015‐GBR	Cervarix (GSK bivalent); Gardasil (Merck quadrivalent)	Female, 12 to 18 years	Vaccinated: 231 Unvaccinated: 114	Odds ratio (medium‐term)	1.18 (0.68 to 2.04)	Vaccine cohort	Cohort; received previous treatment for STI
Sadler 2015‐GBR	Cervarix (GSK bivalent); Gardasil (Merck quadrivalent)	Female, 12 to 18 years	Vaccinated: 189 Unvaccinated: 81	Odds ratio (medium‐term)	2.30 (1.06 to 5.00)	Vaccine cohort	Cohort; *C trachomatis* test positive
Jena 2015‐USA	Gardasil (Merck quadrivalent)	Female, 12 to 18 years	Vaccinated: 21,610 Unvaccinated: 186,501	Difference‐in‐difference odds ratio (short‐term)	1.05 (0.80 to 1.38)	Matched to non‐vaccinated females according to age, zip code of residence and health plan	Cross‐sectional; chlamydia, gonorrhoea, herpes, human immunodeficiency virus or AIDS, or syphilis
Sauvageau 2021‐CAN	Gardasil (Merck quadrivalent)	Female, 11 to 18 years	Vaccinated: 1002 Unvaccinated: 473	Risk ratio (long‐term)	0.63 (0.44 to 0.90)	Age, level of knowledge about STI and number of sexual partners during last 12 months	Cross‐sectional; diagnosis of a STI during last 12 months
Smith 2015‐CAN	Gardasil (Merck quadrivalent)	Female, 13 years	Pre‐vaccine: 131,781 Post‐vaccine: 128,712	Risk ratio (medium‐term)	0.81 (0.63 to 1.04)	Neighbourhood income quintile, hepatitis B vaccination and history of sexual health‐related indicator and birth quarter	Pre‐ vs post‐vaccine introduction; non‐HPV STI

Vaccinated: vaccinated; Unvaccinated: control HPV: human papillomavirus; NR: not reported; SCCS: self‐controlled case series; STI: sexually transmitted infection

**46:** Risk of bias summary: sexual activity (measured by incidence of sexually transmitted infections)

**Study**	**Confounding**	**Selection**	**Classification of interventions**	**Deviations from intended interventions**	**Missing data**	**Measurement of outcomes**	**Selection of reported result**	**Overall risk of bias**
Bednarczyk 2012‐USA	Serious	Low	Low	Low	Low	Low	Low	Serious
Cummings 2012‐USA	Critical	Moderate	Serious	Low	Low	Low	Low	Critical
Sadler 2015‐GBR	Critical	Moderate	Moderate	Low	Moderate	Moderate	Low	Critical
Jena 2015‐USA	Serious	Low	Low	Low	No information	Low	Low	Serious
Sauvageau 2021‐CAN	Serious	Low	Serious	Low	Serious	Moderate	Low	Serious
Smith 2015‐CAN	Serious	Moderate	Serious	Low	Low	Low	Low	Serious

Six studies were included that reported on sexual activity following HPV vaccination (Bednarczyk 2012‐USA; Cummings 2012‐USA; Jena 2015‐USA; Sadler 2015‐GBR; Sauvageau 2021‐CAN; Smith 2015‐CAN). This outcome was measured by the incidence of sexually transmitted infections (STI) in people who did and did not receive HPV vaccination.

All six studies reported on the incidence of STI, including chlamydia, venereal disease, gonorrhoea, herpes, HIV or AIDS, syphilis or trichomonas in females (Bednarczyk 2012‐USA; Cummings 2012‐USA; Jena 2015‐USA; Sadler 2015‐GBR; Sauvageau 2021‐CAN; Smith 2015‐CAN). There was no increase in the incidence of any STI following HPV vaccination. Two studies reported a decreased incidence of STIs following HPV vaccination (Sadler 2015‐GBR; Sauvageau 2021‐CAN).

One study reported on those receiving treatment for STIs in 14‐ to 20‐year‐old females (Sadler 2015‐GBR). There was no increase in the number receiving treatment for STIs following HPV vaccination.

#### Secondary clinical outcomes

##### Cervical screening attendance

See Table for effect estimates and Table for the risk of bias summary of included studies on cervical screening attendance.

**47:** Secondary clinical outcomes effect estimates: cervical screening attendance

**Study**	**Vaccine**	**Population (sex, age at vaccination)**	**Sample size**	**Effect measure (time period)**	**Effect estimate**	**Adjustment factors**	**Notes**
Ba 2021‐USA	Cervarix (GSK bivalent); Gardasil (Merck quadrivalent); Gardasil 9 (Merck nonavalent)	Female, 21 to 26 years*	Vaccinated: 41,814 person years Unvaccinated: 46,320 person‐years	Incidence rate ratio (long term; 3 doses)	1.60 (1.58 to 1.63)	HPV vaccination status, age, place of residence, US census regions, type of health plan, flu vaccine, previous Pap, gonorrhoea, chlamydia, syphilis, trichomoniasis, HIV/AIDS, hepatitis B virus, hepatitis C virus, alcohol drinking, smoking, depression, anxiety and drug abuse	Cohort; *age at outcome
Ba 2021‐USA	Cervarix (GSK bivalent); Gardasil (Merck quadrivalent); Gardasil 9 (Merck nonavalent)	Female, 21 to 26 years*	Vaccinated: 41,814 person‐years Unvaccinated: 811,553 person‐years	Incidence rate ratio (long term; 2 doses)	1.39 (1.37 to 1.41)	HPV vaccination status, age, place of residence, US census regions, type of health plan, flu vaccine, previous Pap, gonorrhoea, chlamydia, syphilis, trichomoniasis, HIV/AIDS, hepatitis B virus, hepatitis C virus, alcohol drinking, smoking, depression, anxiety and drug abuse	Cohort; *age at outcome
Ba 2021‐USA	Cervarix (GSK bivalent); Gardasil (Merck quadrivalent); Gardasil 9 (Merck nonavalent)	Female, 21 to 26 years*	Vaccinated: 67,630 person‐years Unvaccinated: 811,553 person‐years	Incidence rate ratio (long term; 1 dose)	1.14 (1.13 to 1.16)	HPV vaccination status, age, place of residence, US census regions, type of health plan, flu vaccine, previous Pap, gonorrhoea, chlamydia, syphilis, trichomoniasis, HIV/AIDS, hepatitis B virus, hepatitis C virus, alcohol drinking, smoking, depression, anxiety and drug abuse	Cohort; *age at outcome
Badre‐Esfahani 2019‐DNK	NR	Female, 12 to 18 years	Vaccinated: 22,634 Unvaccinated: 2194	Odds ratio (medium term)	2.1 (1.9 to 2.3)	Parental civil status, highest parental education and occupation, family disposable income area of residence and country of origin	Cohort
Boone 2016‐USA	Gardasil (Merck quadrivalent)	Female, 14 to 26 years	Vaccinated: 233 Unvaccinated: 1123	Hazard ratio (long term; 3 doses)	0.94 (0.71 to 1.26)	Age at study entry, age at initial screen and race	Cohort
Boone 2016‐USA	Gardasil (Merck quadrivalent)	Female, 14 to 26 years	Vaccinated: 256 Unvaccinated: 1123	Hazard ratio (long term; 2 doses)	1.01 (0.77 to 1.34)	Age at study entry, age at initial screen and race	Cohort
Boone 2016‐USA	Gardasil (Merck quadrivalent)	Female, 14 to 26 years	Vaccinated: 634 Unvaccinated: 1123	Hazard ratio (long term; 1 dose)	2.98 (2.45 to 3.61)	Age at study entry, age at initial screen and race	Cohort
Boone 2016‐USA	Gardasil (Merck quadrivalent)	Female, 14 to 20 years	Vaccinated: 131 Unvaccinated: 398	Hazard ratio (long term; 3 doses)	1.15 (0.67 to 1.97)	Age at study entry, age at initial screen and race	Cohort
Boone 2016‐USA	Gardasil (Merck quadrivalent)	Female, 14 to 20 years	Vaccinated: 90 Unvaccinated: 398	Hazard ratio (long term; 2 doses)	0.48 (0.25 to 0.90)	Age at study entry, age at initial screen and race	Cohort
Boone 2016‐USA	Gardasil (Merck quadrivalent)	Female, 14 to 20 years	Vaccinated: 241 Unvaccinated: 398	Hazard ratio (long term; 1 dose)	1.65 (1.20 to 2.25)	Age at study entry, age at initial screen and race	Cohort
Boone 2016‐USA	Gardasil (Merck quadrivalent)	Female, 21 to 26 years	Vaccinated: 118 Unvaccinated: 706	Hazard ratio (long term; 3 doses)	1.48 (1.09 to 2.01)	Age at study entry, age at initial screen and race	Cohort
Boone 2016‐USA	Gardasil (Merck quadrivalent)	Female, 21 to 26 years	Vaccinated: 150 Unvaccinated: 706	Hazard ratio (long term; 2 doses)	1.53 (1.17 to 2.02)	Age at study entry, age at initial screen and race	Cohort
Boone 2016‐USA	Gardasil (Merck quadrivalent)	Female, 21 to 26 years	Vaccinated: 393 Unvaccinated: 706	Hazard ratio (long term; 1 dose)	2.38 (1.97 to 2.88)	Age at study entry, age at initial screen and race	Cohort
Del Mistro 2021‐ITA	Gardasil (Merck quadrivalent)	Female, 15 to 25 years	Vaccinated: 4718 Unvaccinated: 91,512	Odds ratio (long term)	1.07 (1.04 to 1.10)	Unadjusted	Cohort
Ruiz‐Sternberg 2014‐COL	NR	Female, < 26 years*	Vaccinated: 506 Unvaccinated: 930	Odds ratio (NR)	2.35 (1.69 to 3.28)	Educational level, knowledge and risk perception	Cohort; *age at outcome
Thamsborg 2020‐DNK	Gardasil (Merck quadrivalent)	Female, < 15 years	Vaccinated: 3983 Unvaccinated: 2148	Risk ratio (long term)	1.14 (1.08 to 1.22)	Unadjusted	Cohort
Thamsborg 2020‐DNK	Gardasil (Merck quadrivalent)	Female, 15 years	Vaccinated: 17,901 Unvaccinated: 2148	Risk ratio (long term)	1.26 (1.19 to 1.33)	Unadjusted	Cohort
Thamsborg 2020‐DNK	Gardasil (Merck quadrivalent)	Female, > 15 years	Vaccinated: 823 Unvaccinated: 2148	Risk ratio (long term)	1.17 (1.08 to 1.26)	Unadjusted	Cohort
Yagi 2019‐JPN	Cervarix (GSK bivalent); Gardasil (Merck quadrivalent)	Female, 12 to 16 years	Vaccinated: 7389 Unvaccinated: 7872	Risk ratio (long term)	0.97 (0.88 to 1.06)	Unadjusted	Cohort
Sauvageau 2021‐CAN	Gardasil (Merck quadrivalent)	Female, 11 to 18 years	Vaccinated: 1002 Unvaccinated: 473	Risk ratio (NR)	0.98 (0.90 to 1.07)	Age, ethnicity, use of contraception, having a family physician, level of knowledge about STI and number of sexual partners during life	Cross‐sectional
Taniguchi 2019‐JPN	NR	Female, 13 to 16 years	Vaccinated: 1753 Unvaccinated: 974	Risk ratio (NR)	1.60 (1.12 to 2.29)	Unadjusted	Cross‐sectional
Baldur‐Felskov 2014‐DNK	Gardasil (Merck quadrivalent)	Female, 12 to 26 years	Pre‐vaccine: 2,302,441 Post‐vaccine: 2,431,726	Rate ratio (long term; 2000 vs 2012)	0.95 (0.95 to 0.95)	Unadjusted	Pre‐ vs post‐vaccine introduction

HPV: human papillomavirus; NR: not reported; STI: sexually transmitted infection

**48:** Risk of bias summary: cervical screening attendance

**Study**	**Confounding**	**Selection**	**Classification of interventions**	**Deviations from intended interventions**	**Missing data**	**Measurement of outcomes**	**Selection of reported result**	**Overall risk of bias**
Ba 2021‐USA	Moderate	Low	Low	Low	Low	Low	Low	Moderate
Badre‐Esfahani 2019‐DNK	Serious	Low	Low	Low	Low	Low	Low	Serious
Boone 2016‐USA	Serious	Moderate	Low	Low	Low	Low	Low	Serious
Del Mistro 2021‐ITA	Critical	Low	Moderate	Low	Low	Low	Low	Critical
Ruiz‐Sternberg 2014‐COL	Serious	Moderate	Moderate	Low	Low	Low	Low	Serious
Thamsborg 2020‐DNK	Critical	Low	Serious	Low	Low	Low	Low	Critical
Sauvageau 2021‐CAN	Moderate	Low	Serious	Low	Serious	Serious	Low	Serious
Taniguchi 2019‐JPN	Critical	Low	Low	Low	Low	Low	Low	Critical
Yagi 2019‐JPN	Critical	Low	Serious	Low	Low	Low	Low	Critical
Baldur‐Felskov 2014‐DNK	Critical	Low	Serious	Low	Low	Low	Low	Critical

Ten studies were identified that reported on cervical screening attendance following HPV vaccination (Ba 2021‐USA; Badre‐Esfahani 2019‐DNK; Baldur‐Felskov 2014‐DNK; Boone 2016‐USA; Del Mistro 2021‐ITA; Ruiz‐Sternberg 2014‐COL; Sauvageau 2021‐CAN; Taniguchi 2019‐JPN; Thamsborg 2020‐DNK; Yagi 2019‐JPN).

Six were cohort studies (Ba 2021‐USA; Badre‐Esfahani 2019‐DNK; Boone 2016‐USA; Del Mistro 2021‐ITA; Ruiz‐Sternberg 2014‐COL; Thamsborg 2020‐DNK), three were cross‐sectional (Sauvageau 2021‐CAN; Taniguchi 2019‐JPN; Yagi 2019‐JPN), and one was a pre‐post vaccine introduction study (Baldur‐Felskov 2014‐DNK).

One cohort study reported an increased odds of cervical screening attendance in the medium term in those receiving HPV vaccination (OR 2.1, 95% CI 1.9 to 2.3; 1 cohort study, 24,828 females) (Badre‐Esfahani 2019‐DNK). From two of the cohort studies, the pooled estimate of the impact of HPV vaccination on rates of cervical screening attendance indicated an increase of 60% in the long term (RR 1.60, 95% CI 1.57 to 1.62; 2 cohort studies, 88,134 person‐years plus 1353 females; I^2^ = 0%) (Analysis 3.1). One additional cohort study reported an increased odds of cervical screening attendance in the long term in those receiving HPV vaccination (OR 2.35, 95% CI 1.69 to 3.28; 1 cohort study, 1436 females) (Ruiz‐Sternberg 2014‐COL).

Two cross‐sectional studies reported little to no difference in cervical screening attendance following HPV vaccination (Sauvageau 2021‐CAN; Yagi 2019‐JPN), while one reported an increased attendance (Taniguchi 2019‐JPN).

The pre‐post vaccine introduction study reported a decrease in cervical screening attendance between 2000 and 2012 (Baldur‐Felskov 2014‐DNK).

Two studies also reported on the effectiveness of two doses or one dose (Ba 2021‐USA; Boone 2016‐USA). Both indicated an increased likelihood of attending cervical screening following HPV vaccination with one or two doses.

##### Treatment for HPV‐related disease

See Table for effect estimates and Table for the risk of bias summary of included studies on treatment for HPV‐related disease.

**49:** Secondary clinical outcomes effect estimates: treatment rates

**Study**	**Vaccine**	**Population (sex, age at vaccination)**	**Sample size**	**Effect measure (time period)**	**Effect estimate**	**Adjustment factors**	**Notes**
Paraskevaidis 2020‐GRC	Gardasil (Merck quadrivalent)	Female, NR	Vaccinated: 849 Unvaccinated: 849	Risk ratio (NR)	0.02 (0.00 to 0.11)	Unadjusted	Cohort; treatment needed for suspected high‐grade lesion
Elies 2022‐FRA	Cervarix (GSK bivalent); Gardasil (Merck quadrivalent)	Female, 19 to 30 years	Vaccinated: 4129 Unvaccinated: 38,323	Hazard ratio (long‐term)	0.59 (0.39 to 0.90)	Unadjusted	Cohort; conisation rate
Clark 2021‐CAN	Gardasil (Merck quadrivalent)	Female, 18 to 23 years*	Pre‐vaccine: 121,019 Post‐vaccine: 100,020	Incidence rate ratio (long‐term; 2003‐8 vs 2013‐18)	0.24 (0.19 to 0.30)	Unadjusted	Pre‐ vs post‐vaccine introduction; trichloroacetic acid treatment; *age at outcome
Clark 2021‐CAN	Gardasil (Merck quadrivalent)	Female, 18 to 23 years*	Pre‐vaccine: 121,019 Post‐vaccine: 100,020	Incidence rate ratio (long‐term; 2003‐8 vs 2013‐18)	0.13 (0.10 to 0.17)	Unadjusted	Pre‐ vs post‐vaccine introduction; laser of vulval lesion; *age at outcome
Clark 2021‐CAN	Gardasil (Merck quadrivalent)	Female, 18 to 23 years*	Pre‐vaccine: 121,019 Post‐vaccine: 100,020	Incidence rate ratio (long‐term; 2003‐8 vs 2013‐18)	0.18 (0.13 to 0.24)	Unadjusted	Pre‐ vs post‐vaccine introduction; cervical conisation; *age at outcome
Clark 2021‐CAN	Gardasil (Merck quadrivalent)	Female, 18 to 23 years*	Pre‐vaccine: 121,019 Post‐vaccine: 100,020	Incidence rate ratio (long‐term; 2003‐8 vs 2013‐18)	0.14 (0.11 to 0.17)	Unadjusted	Pre‐ vs post‐vaccine introduction; loop electrosurgical excision procedure; *age at outcome
Clark 2021‐CAN	Gardasil (Merck quadrivalent)	Female, 18 to 23 years*	Pre‐vaccine: 121,019 Post‐vaccine: 100,020	Incidence rate ratio (long‐term; 2003‐8 vs 2013‐18)	0.17 (0.10 to 0.28)	Unadjusted	Pre‐ vs post‐vaccine introduction; cryotherapy; *age at outcome
Clark 2021‐CAN	Gardasil (Merck quadrivalent)	Female, 18 to 23 years*	Pre‐vaccine: 121,019 Post‐vaccine: 100,020	Incidence rate ratio (long‐term; 2003‐8 vs 2013‐18)	0.51 (0.50 to 0.53)	Unadjusted	Pre‐ vs post‐vaccine introduction; colposcopy; *age at outcome
Cruickshank 2017‐GBR	Cervarix (GSK bivalent); Gardasil (Merck quadrivalent)	Female, 12 to 18 years	Pre‐vaccine: 1344 Post‐vaccine: 5669	Incidence rate ratio (long‐term; 2008‐9 vs 2009‐14)	0.51 (0.41 to 0.66)	Unadjusted	Pre‐ vs post‐vaccine introduction; ablation (cold coagulation/cryotherapy)
Cruickshank 2017‐GBR	Cervarix (GSK bivalent); Gardasil (Merck quadrivalent)	Female, 12 to 18 years	Pre‐vaccine: 1344 Post‐vaccine: 5669	Incidence rate ratio (long‐term; 2008‐9 vs 2009‐14)	0.67 (0.58 to 0.79)	Unadjusted	Pre‐ vs post‐vaccine introduction; LLETZ/type‐3 excision
Harrison 2014‐AUS	Gardasil (Merck quadrivalent)	Female, 15 to 27 years*	N = 1,175,879 patient encounters	Risk ratio (medium‐term; 2002‐6 vs 2008‐12)	0.39 (0.32 to 0.46)	Unadjusted	Pre‐ vs post‐vaccine introduction; genital warts management per 1000 patient encounters; *age at outcome
Harrison 2014‐AUS	Gardasil (Merck quadrivalent)	Female, 28 to 49 years*	N = 1,175,879 patient encounters	Risk ratio (long‐term; 2002‐6 vs 2008‐12)	0.64 (0.48 to 0.85)	Unadjusted	Pre‐ vs post‐vaccine introduction; genital warts management per 1000 patient encounters; *age at outcome
Harrison 2014‐AUS	Gardasil (Merck quadrivalent)	Female, ≥ 50 years*	N = 1,175,879 patient encounters	Risk ratio (long‐term; 2002‐6 vs 2008‐12)	1.00 (1.00 to 1.00)	Unadjusted	Pre‐ vs post‐vaccine introduction; genital warts management per 1000 patient encounters; *age at outcome
Harrison 2014‐AUS	Gardasil (Merck quadrivalent)	Male, 15 to 27 years*	N = 1,175,879 patient encounters	Risk ratio (medium‐term; 2002‐6 vs 2008‐12)	0.95 (0.84 to 1.09)	Unadjusted	Pre‐ vs post‐vaccine introduction; genital warts management per 1000 patient encounters; *age at outcome
Harrison 2014‐AUS	Gardasil (Merck quadrivalent)	Male, 28 to 49 years*	N = 1,175,879 patient encounters	Risk ratio (long‐term; 2002‐6 vs 2008‐12)	0.85 (0.70 to 1.03)	Unadjusted	Pre‐ vs post‐vaccine introduction; genital warts management per 1000 patient encounters; *age at outcome
Harrison 2014‐AUS	Gardasil (Merck quadrivalent)	Male, ≥ 50 years*	N = 1,175,879 patient encounters	Risk ratio (long‐term; 2002‐6 vs 2008‐12)	0.78 (0.42 to 1.43)	Unadjusted	Pre‐ vs post‐vaccine introduction; genital warts management per 1000 patient encounters; *age at outcome

LLETZ: large loop excision of the transformation zone; NR: not reported

**50:** Risk of bias summary: treatment rates

**Study**	**Confounding**	**Selection**	**Classification of interventions**	**Deviations from intended interventions**	**Missing data**	**Measurement of outcomes**	**Selection of reported result**	**Overall risk of bias**
Paraskevaidis 2020‐GRC	Serious	Serious	Serious	Low	Serious	Low	Low	Serious
Elies 2022‐FRA	Critical	Moderate	Low	Low	Low	Low	Low	Critical
Clark 2021‐CAN	Critical	Moderate	Low	Low	Low	Low	Low	Critical
Cruickshank 2017‐GBR	Critical	Moderate	Serious	Low	Low	Low	Low	Critical
Harrison 2014‐AUS	Critical	Low	Serious	Low	Low	Low	Low	Critical

Five studies were identified that reported on treatment rates following HPV vaccination (Clark 2021‐CAN; Cruickshank 2017‐GBR; Elies 2022‐FRA; Harrison 2014‐AUS; Paraskevaidis 2020‐GRC). Two were cohort studies (Elies 2022‐FRA; Paraskevaidis 2020‐GRC) and three were pre‐post vaccine introduction studies (Clark 2021‐CAN; Cruickshank 2017‐GBR; Harrison 2014‐AUS).

One cohort study reported a decrease in conisation rates (HR 0.59, 95% 0.39 to 0.90) (Elies 2022‐FRA) and the other reported a decrease in treatment required for suspected high‐grade lesions (RR 0.02, 95% CI 0.00 to 0.11) following HPV vaccination (Paraskevaidis 2020‐GRC). Neither cohort study adjusted for confounding in the analysis.

One of the pre‐post vaccine introduction studies reported a decrease from 2003‐2008 to 2013‐2018 for trichloroacetic acid treatment, laser of vulval lesions, cervical conisation, loop electrosurgical excision procedure, cryotherapy and colposcopy (Clark 2021‐CAN). Another pre‐post vaccine introduction study reported a decrease from 2008‐2009 to 2009‐2014 for ablation (cold coagulation/cryotherapy) and loop electrosurgical excision procedure (Cruickshank 2017‐GBR). The third study reported a decrease in anogenital warts management between 2002‐2006 and 2008‐2012 for females ages 15 to 49 years (Harrison 2014‐AUS). For males, a decrease in treatment rates during this period was also reported, but confidence intervals included no difference.

##### Anogenital warts

See Table and Table for effect estimates and Table for the risk of bias summary of included studies on anogenital warts. HPV vaccination probably reduces the incidence of anogenital warts (moderate‐certainty evidence; Table).

**51:** Secondary clinical outcomes effect estimates: anogenital warts (cohort studies)

**Study**	**Vaccine**	**Population (sex, age)**	**Sample size**	**Effect measure (time period)**	**Effect estimate**	**Adjustment factors**	**Notes**
Baandrup 2021‐DNK	Gardasil (Merck quadrivalent)	Female, 12 to 14 years	Vaccinated: 134,908 person‐years Unvaccinated: 1,904,895 person‐years	Incidence rate ratio (long‐term; 1 dose)	0.29 (0.22 to 0.38)	Maternal highest achieved education, attained age, socioeconomic status, calendar time	Cohort
Baandrup 2021‐DNK	Gardasil (Merck quadrivalent)	Female, 15 to 16 years	Vaccinated: 23,106 person‐years Unvaccinated: 1,904,895 person‐years	Incidence rate ratio (long‐term; 1 dose)	0.38 (0.29 to 0.49)	Maternal highest achieved education, attained age, socioeconomic status, calendar time	Cohort
Baandrup 2021‐DNK	Gardasil (Merck quadrivalent)	Female, 17 to 18 years	Vaccinated: 8473 person‐years Unvaccinated: 1,904,895 person‐years	Incidence rate ratio (long‐term; 1 dose)	0.56 (0.42 to 0.73)	Maternal highest achieved education, attained age, socioeconomic status, calendar time	Cohort
Baandrup 2021‐DNK	Gardasil (Merck quadrivalent)	Female, ≥ 19 years	Vaccinated: 69,166 person‐years Unvaccinated: 1,904,895 person‐years	Incidence rate ratio (long‐term; 1 dose)	1.36 (1.24 to 1.49)	Maternal highest achieved education, attained age, socioeconomic status, calendar time	Cohort
Baandrup 2021‐DNK	Gardasil (Merck quadrivalent)	Female, 12 to 14 years	Vaccinated: 269,786 person‐years Unvaccinated: 1,904,895 person‐years	Incidence rate ratio (long‐term; 2 doses)	0.22 (0.18 to 0.26)	Maternal highest achieved education, attained age, socioeconomic status, calendar time	Cohort
Baandrup 2021‐DNK	Gardasil (Merck quadrivalent)	Female, 15 to 16 years	Vaccinated: 50,448 person‐years Unvaccinated: 1,904,895 person‐years	Incidence rate ratio (long‐term; 2 doses)	0.32 (0.26 to 0.38)	Maternal highest achieved education, attained age, socioeconomic status, calendar time	Cohort
Baandrup 2021‐DNK	Gardasil (Merck quadrivalent)	Female, 17 to 18 years	Vaccinated: 13,290 person‐years Unvaccinated: 1,904,895 person‐years	Incidence rate ratio (long‐term; 2 doses)	0.49 (0.39 to 0.62)	Maternal highest achieved education, attained age, socioeconomic status, calendar time	Cohort
Baandrup 2021‐DNK	Gardasil (Merck quadrivalent)	Female, ≥ 19 years	Vaccinated: 127,453 person‐years Unvaccinated: 1,904,895 person‐years	Incidence rate ratio (long‐term; 2 doses)	1.03 (0.95 to 1.12)	Maternal highest achieved education, attained age, socioeconomic status, calendar time	Cohort
Baandrup 2021‐DNK	Gardasil (Merck quadrivalent)	Female, 12 to 14 years	Vaccinated: 1,204,485 person‐years Unvaccinated: 1,904,895 person‐years	Incidence rate ratio (long‐term; 3 doses)	0.16 (0.15 to 0.18)	Maternal highest achieved education, attained age, socioeconomic status, calendar time	Cohort
Baandrup 2021‐DNK	Gardasil (Merck quadrivalent)	Female, 15 to 16 years	Vaccinated: 239,722 person‐years Unvaccinated: 1,904,895 person‐years	Incidence rate ratio (long‐term; 3 doses)	0.20 (0.18 to 0.22)	Maternal highest achieved education, attained age, socioeconomic status, calendar time	Cohort
Baandrup 2021‐DNK	Gardasil (Merck quadrivalent)	Female, 17 to 18 years	Vaccinated: 72,162 person‐years Unvaccinated: 1,904,895 person‐years	Incidence rate ratio (long‐term; 3 doses)	0.29 (0.25 to 0.33)	Maternal highest achieved education, attained age, socioeconomic status, calendar time	Cohort
Baandrup 2021‐DNK	Gardasil (Merck quadrivalent)	Female, ≥ 19 years	Vaccinated: 418,219 person‐years Unvaccinated: 1,904,895 person‐years	Incidence rate ratio (long‐term; 3 doses)	0.76 (0.71 to 0.81)	Maternal highest achieved education, attained age, socioeconomic status, calendar time	Cohort
Cho 2024‐KOR	Gardasil (Merck quadrivalent); Gardasil 9 (Merck nonavalent)	Female, 12 to 13 years*	Vaccinated: 166,031 Unvaccinated: 166,031	Hazard ratio (medium‐term)	1.29 (0.57 to 2.94)	Birth year, socioeconomic status, regional urbanisation level	Cohort; *age at vaccination
Cho 2024‐KOR	Gardasil (Merck quadrivalent); Gardasil 9 (Merck nonavalent)	Female, 12 to 13 years*	Vaccinated: 166,031 Unvaccinated: 166,031	Hazard ratio (long‐term)	0.39 (0.28 to 0.52)	Birth year, socioeconomic status, regional urbanisation level	Cohort; *age at vaccination
Dominiak‐Felden 2015‐BEL	Gardasil (Merck quadrivalent)	Female, 10 to 23 years	Vaccinated: 116,379 person‐years Unvaccinated: 218,524 person‐years	Risk ratio (long‐term; 3 doses)	0.12 (0.07 to 0.26)	Age	Cohort
Dominiak‐Felden 2015‐BEL	Gardasil (Merck quadrivalent)	Female, 10 to 23 years	Vaccinated: 30,402 person‐years Unvaccinated: 218,524 person‐years	Risk ratio (long‐term; 1 or 2 doses)	0.50 (0.30 to 0.83)	Age	Cohort
Hariri 2018‐USA	Gardasil (Merck quadrivalent)	Female, 11 to 22 years	Vaccinated: 21,631 Unvaccinated: 31,563	Hazard ratio (long‐term; 3 doses)	0.23 (0.17 to 0.31)	Race/ethnicity, health plan, age at enrolment in the health plan, age, age at first sexual activity, age at first dose of HPV vaccine, continuously enrolled, months enrolled in health plan, preventive health visits, Medicaid enrolment, oral contraceptive use, history of tests for pregnancy, chlamydia or gonorrhoea	Cohort
Hariri 2018‐USA	Gardasil (Merck quadrivalent)	Female, 11 to 22 years	Vaccinated: 2729 Unvaccinated: 31,563	Hazard ratio (long‐term; 2 doses)	0.32 (0.17 to 0.59)	Race/ethnicity, health plan, age at enrolment in the health plan, age, age at first sexual activity, age at first dose of HPV vaccine, continuously enrolled, months enrolled in health plan, preventive health visits, Medicaid enrolment, oral contraceptive use, history of tests for pregnancy, chlamydia or gonorrhoea	Cohort
Hariri 2018‐USA	Gardasil (Merck quadrivalent)	Female, 11 to 22 years	Vaccinated: 5864 Unvaccinated: 31,563	Hazard ratio (long‐term; 1 dose)	0.81 (0.60 to 1.08)	Race/ethnicity, health plan, age at enrolment in the health plan, age, age at first sexual activity, age at first dose of HPV vaccine, continuously enrolled, months enrolled in health plan, preventive health visits, Medicaid enrolment, oral contraceptive use, history of tests for pregnancy, chlamydia or gonorrhoea	Cohort
Herweijer 2018‐SWE	Gardasil (Merck quadrivalent)	Female, 10 to 16 years	N = 1,045,165	Incidence rate ratio (medium‐term; 3 doses)	0.18 (0.15 to 0.22)	Age and parental education level	Cohort
Herweijer 2018‐SWE	Gardasil (Merck quadrivalent)	Female, 17 to 19 years	N = 1,045,165	Incidence rate ratio (medium‐term; 3 doses)	0.23 (0.18 to 0.29)	Age and parental education level	Cohort
Herweijer 2018‐SWE	Gardasil (Merck quadrivalent)	Female, 10 to 19 years	N = 1,045,165	Incidence rate ratio (medium‐term; 3 doses)	0.20 (0.17 to 0.23)	Age and parental education level	Cohort
Herweijer 2018‐SWE	Gardasil (Merck quadrivalent)	Female, 10 to 16 years	N = 1,045,165	Incidence rate ratio (medium‐term; 2 doses)	0.29 (0.21 to 0.40)	Age and parental education level	Cohort
Herweijer 2018‐SWE	Gardasil (Merck quadrivalent)	Female, 17 to 19 years	N = 1,045,165	Incidence rate ratio (medium‐term; 2 doses)	0.35 (0.26 to 0.47)	Age and parental education level	Cohort
Herweijer 2018‐SWE	Gardasil (Merck quadrivalent)	Female, 10 to 19 years	N = 1,045,165	Incidence rate ratio (medium‐term; 2 doses)	0.32 (0.26 to 0.40)	Age and parental education level	Cohort
Herweijer 2018‐SWE	Gardasil (Merck quadrivalent)	Female, 10 to 16 years	N = 1,045,165	Incidence rate ratio (medium‐term; 1 dose)	0.31 (0.20 to 0.49)	Age and parental education level	Cohort
Herweijer 2018‐SWE	Gardasil (Merck quadrivalent)	Female, 17 to 19 years	N = 1,045,165	Incidence rate ratio (medium‐term; 1 dose)	0.71 (0.55 to 0.92)	Age and parental education level	Cohort
Herweijer 2018‐SWE	Gardasil (Merck quadrivalent)	Female, 10 to 19 years	N = 1,045,165	Incidence rate ratio (medium‐term; 1 dose)	0.54 (0.43 to 0.68)	Age and parental education level	Cohort
Howell‐Jones 2013‐GBR	Cervarix (GSK bivalent)	Female, 15 years*	N = 1,212,679	Incidence rate ratio (medium‐term)	0.83 (0.73 to 0.95)	Chlamydia diagnosis rate	Cohort; *age at outcome
Howell‐Jones 2013‐GBR	Cervarix (GSK bivalent)	Female, 16 years*	N = 1,247,309	Incidence rate ratio (medium‐term)	0.81 (0.73 to 0.89)	Chlamydia diagnosis rate	Cohort; *age at outcome
Howell‐Jones 2013‐GBR	Cervarix (GSK bivalent)	Female, 17 years*	N = 1,278,085	Incidence rate ratio (medium‐term)	0.69 (0.62 to 0.76)	Chlamydia diagnosis rate	Cohort; *age at outcome
Howell‐Jones 2013‐GBR	Cervarix (GSK bivalent)	Female, 18 years*	N = 1,314,995	Incidence rate ratio (medium‐term)	0.73 (0.65 to 0.83)	Chlamydia diagnosis rate	Cohort; *age at outcome
Howell‐Jones 2013‐GBR	Cervarix (GSK bivalent)	Female, 19 years*	N = 1,344,061	Incidence rate ratio (long‐term)	0.97 (0.86 to 1.09)	Chlamydia diagnosis rate	Cohort; *age at outcome
Howell‐Jones 2013‐GBR	Cervarix (GSK bivalent)	Female, 20 years*	N = 1,358,690	Incidence rate ratio (long‐term)	0.90 (0.74 to 1.10)	Chlamydia diagnosis rate	Cohort; *age at outcome
Munoz‐Quiles 2021‐ESP	Gardasil (Merck quadrivalent)	Female, 14 years	Vaccinated: 53,579 Unvaccinated: 290,708	Risk ratio (long‐term; 3 doses)	0.26 (0.21 to 0.32)	Age, calendar year, health department, immunocompromising conditions	Cohort
Munoz‐Quiles 2021‐ESP	Gardasil (Merck quadrivalent)	Female, 14 years	Vaccinated: 3526 Unvaccinated: 290,708	Risk ratio (long‐term; 2 doses)	0.40 (0.22 to 0.65)	Age, calendar year, health department, immunocompromising conditions	Cohort
Munoz‐Quiles 2021‐ESP	Gardasil (Merck quadrivalent)	Female, 14 years	Vaccinated: 1823 Unvaccinated: 290,708	Risk ratio (long‐term; 1 dose)	0.25 (0.08 to 0.56)	Age, calendar year, health department, immunocompromising conditions	Cohort
Nygard 2023‐NOR	Gardasil (Merck quadrivalent)	Female, ≤ 13 at vaccination	Vaccinated: 174,506 Unvaccinated: 869,289	Hazard ratio (long‐term)	0.2 (0.2 to 0.3)	Age, vaccination status, vaccination age, calendar time	Cohort
Nygard 2023‐NOR	Gardasil (Merck quadrivalent)	Female, 14 to 15 at vaccination	Vaccinated: 11,039* Unvaccinated: 869,289	Hazard ratio (long‐term)	0.2 (0.2 to 0.3)	Age, vaccination status, vaccination age, calendar time	Cohort; *total vaccinated 14‐19 years
Nygard 2023‐NOR	Gardasil (Merck quadrivalent)	Female, 16 to 17 at vaccination	Vaccinated: 11,039* Unvaccinated: 869,289	Hazard ratio (long‐term)	0.3 (0.2 to 0.3)	Age, vaccination status, vaccination age, calendar time	Cohort; *total vaccinated 14‐19 years
Nygard 2023‐NOR	Gardasil (Merck quadrivalent)	Female, 18 to 19 at vaccination	Vaccinated: 11,039* Unvaccinated: 869,289	Hazard ratio (long‐term)	0.5 (0.4 to 0.7)	Age, vaccination status, vaccination age, calendar time	Cohort; *total vaccinated 14‐19 years
Nygard 2023‐NOR	Gardasil (Merck quadrivalent)	Female, 20 to 24 at vaccination	Vaccinated: 3320 Unvaccinated: 869,289	Hazard ratio (long‐term)	1.0 (0.8 to 1.4)	Age, vaccination status, vaccination age, calendar time	Cohort
Nygard 2023‐NOR	Gardasil (Merck quadrivalent)	Female, 25 to 29 at vaccination	Vaccinated: 2725 Unvaccinated: 869,289	Hazard ratio (long‐term)	1.3 (0.8 to 2.2)	Age, vaccination status, vaccination age, calendar time	Cohort
Nygard 2023‐NOR	Gardasil (Merck quadrivalent)	Female, 30+ at vaccination	Vaccinated: 1160 Unvaccinated: 869,289	Hazard ratio (long‐term)	2.7 (1.1 to 6.6)	Age, vaccination status, vaccination age, calendar time	Cohort
Osmani 2022‐DEU	Gardasil (Merck quadrivalent); Cervarix (GSK bivalent); Gardasil 9 (Merck nonavalent)	Female, 19 to 28 years	Vaccinated: 121,337 Unvaccinated: 218,953	Hazard ratio (long‐term)	0.37 (0.34 to 0.40)	Place of residence, type of vaccine, contraception use	Cohort
Perkins 2017‐USA	Gardasil (Merck quadrivalent)	Female, 9 to 25 years	Vaccinated: 185,973 Unvaccinated: 201,933	Incidence rate ratio (long‐term)	0.52 (0.60 to 0.46)	Age, geographic region, income, proportion of minorities in county of residence, calendar year	Cohort
Reyburn 2023‐FJI	Gardasil (Merck quadrivalent)	Female, 15 to 23 years	Vaccinated: 189 Unvaccinated: 376	Prevalence ratio (3 doses; long‐term)	1.28 (0.37 to 4.48)	Age, ethnicity and smoking	Cohort
Reyburn 2023‐FJI	Gardasil (Merck quadrivalent)	Female, 15 to 23 years	Vaccinated: 99 Unvaccinated: 376	Prevalence ratio (2 doses; long‐term)	0.61 (0.08 to 4.95)	Age, ethnicity and smoking	Cohort
Reyburn 2023‐FJI	Gardasil (Merck quadrivalent)	Female, 15 to 23 years	Vaccinated: 158 Unvaccinated: 376	Prevalence ratio (1 dose; long‐term)	0.37 (0.05 to 2.95)	Age, ethnicity and smoking	Cohort
Swedish 2013‐USA	Gardasil (Merck quadrivalent)	Male, 26 to 76 years*	Vaccinated: 116 Unvaccinated: 197	Hazard ratio (medium‐term)	0.45 (0.22 to 0.92)	Age, anogenital condyloma within 5 years prior to study entry, oncogenic HPV infection	Cohort; *age at outcome
Willows 2018‐CAN	Gardasil (Merck quadrivalent)	Female, 9 to 18 years	Vaccinated: 3521 Unvaccinated: 94,327	Hazard ratio (long‐term; 1 dose)	0.6 (0.2 to 1.8)	Birth date, area of residence, previous hospitalisation, previous physician visit	Cohort
Willows 2018‐CAN	Gardasil (Merck quadrivalent)	Female, 9 to 18 years	Vaccinated: 6666 Unvaccinated: 94,327	Hazard ratio (long‐term; 2 doses)	1.4 (0.6 to 3.3)	Birth date, area of residence, previous hospitalisation, previous physician visit	Cohort
Willows 2018‐CAN	Gardasil (Merck quadrivalent)	Female, 9 to 18 years	Vaccinated: 21,277 Unvaccinated: 94,327	Hazard ratio (long‐term; 3 doses)	0.4 (0.3 to 0.7)	Birth date, area of residence, previous hospitalisation, previous physician visit	Cohort
Woestenberg 2020‐NLD	Cervarix (GSK bivalent)	Female, 12 to 16 years	Vaccinated: 154,088 person‐years Unvaccinated: 144,129 person‐years	Incidence rate ratio (long‐term; 3 doses)	0.72 (0.61 to 0.86)	Age as time‐varying, migration background, educational level, fear of STI/HIV consultations, mean number of GP consultations per year	Cohort
Woestenberg 2020‐NLD	Cervarix (GSK bivalent)	Female, 12 to 16 years	Vaccinated: 26,409 person‐years Unvaccinated: 144,129 person‐years	Incidence rate ratio (long‐term; 1 or 2 doses)	0.96 (0.68 to 1.32)	Age as time‐varying, migration background, educational level, fear of STI/HIV consultations, mean number of GP consultations per year	Cohort
Zeybek 2018‐USA	Gardasil (Merck quadrivalent)	Female and male, 9 to 14 years	Vaccinated: 16,844 Unvaccinated: 94,233	Hazard ratio (long‐term; 1 dose)	0.80 (0.34 to 1.90)	Sex, region, history of STI	Cohort
Zeybek 2018‐USA	Gardasil (Merck quadrivalent)	Female and male, 9 to 14 years	Vaccinated: 17,090 Unvaccinated: 94,233	Hazard ratio (long‐term; 2 doses)	1.36 (0.65 to 2.86)	Sex, region, history of STI	Cohort
Zeybek 2018‐USA	Gardasil (Merck quadrivalent)	Female and male, 9 to 14 years	Vaccinated: 60,299 Unvaccinated: 94,233	Hazard ratio (long‐term; 3 doses)	0.78 (0.46 to 1.35)	Sex, region, history of STI	Cohort
Zeybek 2018‐USA	Gardasil (Merck quadrivalent)	Female and male, 15 to 19 years	Vaccinated: 26,543 Unvaccinated: 141,662	Hazard ratio (long‐term; 1 dose)	0.65 (0.49 to 0.85)	Sex, region, history of STI	Cohort
Zeybek 2018‐USA	Gardasil (Merck quadrivalent)	Female and male, 15 to 19 years	Vaccinated: 27,884 Unvaccinated: 141,662	Hazard ratio (long‐term; 2 doses)	0.67 (0.51 to 0.89)	Sex, region, history of STI	Cohort
Zeybek 2018‐USA	Gardasil (Merck quadrivalent)	Female and male, 15 to 19 years	Vaccinated: 87,235 Unvaccinated: 141,662	Hazard ratio (long‐term; 3 doses)	0.58 (0.49 to 0.70)	Sex, region, history of STI	Cohort
Zeybek 2018‐USA	Gardasil (Merck quadrivalent)	Female and male, 20 to 26 years	Vaccinated: 10,893 Unvaccinated: 51,068	Hazard ratio (long‐term; 1 dose)	0.96 (0.72 to 1.28)	Sex, region, history of STI	Cohort
Zeybek 2018‐USA	Gardasil (Merck quadrivalent)	Female and male, 20 to 26 years	Vaccinated: 10,658 Unvaccinated: 51,068	Hazard ratio (long‐term; 2 doses)	1.15 (0.87 to 1.51)	Sex, region, history of STI	Cohort
Zeybek 2018‐USA	Gardasil (Merck quadrivalent)	Female and male, 20 to 26 years	Vaccinated: 29,517 Unvaccinated: 51,068	Hazard ratio (long‐term; 3 doses)	1.11 (0.91 to 1.35)	Sex, region, history of STI	Cohort

GP: general practitioner; HPV: human papillomavirus; STI: sexually transmitted infection

**52:** Secondary clinical outcomes effect estimates: anogenital warts (other study designs)

**Study**	**Vaccine**	**Population (sex, age)**	**Sample size**	**Effect measure (time period)**	**Effect estimate**	**Adjustment factors**	**Notes**
Krasnopolsky 2020‐RUS	NR	Female, 18 to 36 years*	Vaccinated: 320 Unvaccinated: 120	Risk ratio (medium‐term)	0.00 (0.00 to 0.02)	Unadjusted	Cross‐sectional; *age at outcome; no cases in exposed group
Petras 2015‐CZE	Gardasil (Merck quadrivalent)	Female, 16 to 40 years	Vaccinated: 882 Unvaccinated: 17,344	Odds ratio (long‐term; 3 doses)	0.12 (0.05 to 0.25)	Age	Cross‐sectional
Petras 2015‐CZE	Cervarix (GSK bivalent)	Female, 16 to 40 years	Vaccinated: 633 Unvaccinated: 17,344	Odds ratio (long‐term; 3 doses)	1.18 (0.86 to 1.63)	Age	Cross‐sectional
Petras 2015‐CZE	Gardasil (Merck quadrivalent)	Female, 16 to 40 years	Vaccinated: 1086 Unvaccinated: 17,344	Odds ratio (long‐term; at least 1 dose)	0.09 (0.04 to 0.20)	Age	Cross‐sectional
Petras 2015‐CZE	Cervarix (GSK bivalent)	Female, 16 to 40 years	Vaccinated: 769 Unvaccinated: 17,344	Odds ratio (long‐term; at least 1 dose)	1.10 (0.82 to 1.49)	Age	Cross‐sectional
Sadler 2015‐GBR	NR	Female, 12 to 18 years	Vaccinated: 231 Unvaccinated: 132	Odds ratio (medium‐term)	0.66 (0.34 to 1.31)	Vaccine cohort	Cross‐sectional
Ali 2013‐AUS	Gardasil (Merck quadrivalent)	Female, 15 to 24 years*	6950 cases of AGW	Rate ratio (medium‐term; 2000‐7 vs 2007‐11)	0.33 (0.30 to 0.37)	Unadjusted	Pre‐ vs post‐vaccine introduction; *age at outcome; vulval/vaginal warts
Ali 2013‐AUS	Gardasil (Merck quadrivalent)	Female, 25 to 34 years*	6950 cases of AGW	Rate ratio (long‐term; 2000‐7 vs 2007‐11)	0.60 (0.54 to 0.66)	Unadjusted	Pre‐ vs post‐vaccine introduction; *age at outcome; vulval/vaginal warts
Ali 2013‐AUS	Gardasil (Merck quadrivalent)	Male, 15 to 24 years*	6950 cases of AGW	Rate ratio (medium‐term; 2000‐7 vs 2007‐11)	0.76 (0.62 to 0.96)	Unadjusted	Pre‐ vs post‐vaccine introduction; *age at outcome; penile warts
Ali 2013‐AUS	Gardasil (Merck quadrivalent)	Male, 25 to 34 years*	6950 cases of AGW	Rate ratio (long‐term; 2000‐7 vs 2007‐11)	0.81 (0.66 to 0.99)	Unadjusted	Pre‐ vs post‐vaccine introduction; *age at outcome; penile warts
Ali 2013‐AUS	Gardasil (Merck quadrivalent)	Male, 15 to 24 years*	6950 cases of AGW	Rate ratio (medium‐term; 2000‐7 vs 2007‐11)	0.92 (0.77 to 1.10)	Unadjusted	Pre‐ vs post‐vaccine introduction; *age at outcome; anal warts
Ali 2013‐AUS	Gardasil (Merck quadrivalent)	Male, 25 to 34 years*	6950 cases of AGW	Rate ratio (long‐term; 2000‐7 vs 2007‐11)	0.69 (0.59 to 0.79)	Unadjusted	Pre‐ vs post‐vaccine introduction; *age at outcome; anal warts
Bauer 2012‐USA	Gardasil (Merck quadrivalent)	Female, all ages*	Pre‐vaccine: 1,679,684 person‐years Post‐vaccine: 1,813,222 person‐years	Incidence rate ratio (medium‐term; 2007 vs 2010	0.88 (0.86 to 0.90)	Unadjusted	Pre‐ vs post‐vaccine introduction; *age at outcome
Bauer 2012‐USA	Gardasil (Merck quadrivalent)	Male, all ages*	Pre‐vaccine: 232,032 person‐years Post‐vaccine: 290,456 person‐years	Incidence rate ratio (medium‐term; 2007 vs 2010	0.93 (0.90 to 0.96)	Unadjusted	Pre‐ vs post‐vaccine introduction; *age at outcome
Canvin 2017‐GBR	Cervarix (GSK bivalent); Gardasil (Merck quadrivalent)	Female, 15 to 19 years*	NR	Incidence rate ratio (medium‐term; 2009 vs 2010‐2014)	0.69 (0.67 to 0.72)	Unadjusted	Pre‐ vs post‐vaccine introduction; *age at outcome
Canvin 2017‐GBR	Cervarix (GSK bivalent); Gardasil (Merck quadrivalent)	Female, 20 to 24 years*	NR	Incidence rate ratio (medium‐term; 2009 vs 2010‐2014)	0.91 (0.87 to 0.94)	Unadjusted	Pre‐ vs post‐vaccine introduction; *age at outcome
Canvin 2017‐GBR	Cervarix (GSK bivalent); Gardasil (Merck quadrivalent)	Male, 15 to 19 years*	NR	Incidence rate ratio (medium‐term; 2009 vs 2010‐2014)	0.75 (0.70 to 0.79)	Unadjusted	Pre‐ vs post‐vaccine introduction; *age at outcome
Canvin 2017‐GBR	Cervarix (GSK bivalent); Gardasil (Merck quadrivalent)	Male, 20 to 24 years*	NR	Incidence rate ratio (medium‐term; 2009 vs 2010‐2014)	0.88 (0.85 to 0.91)	Unadjusted	Pre‐ vs post‐vaccine introduction; *age at outcome
Chow 2021b‐AUS	Gardasil (Merck quadrivalent)	Female, ≥ 15 years*	Pre‐vaccine: 35,137 Post‐vaccine: 81,204	Prevalence ratio (long‐term; 2004‐7 vs 2013‐18	0.42 (0.40 to 0.44)	Unadjusted	Pre‐ vs post‐vaccine introduction; *age at outcome
Chow 2021b‐AUS	Gardasil (Merck quadrivalent)	Male, ≥ 15 years*	Pre‐vaccine: 32,022 Post‐vaccine: 30,343	Prevalence ratio (long‐term; 2004‐7 vs 2013‐18	0.55 (0.53 to 0.57)	Unadjusted	Pre‐ vs post‐vaccine introduction; *age at outcome
Chow 2019‐AUS	Gardasil (Merck quadrivalent)	Male, ≤ 15 years*	Pre‐vaccine: 152 Post‐vaccine: 146	Prevalence ratio (medium‐term; 2014‐15 vs 2016‐17)	0.15 (0.00 to 1.16)	Unadjusted	Pre‐ vs post‐vaccine introduction; *age at outcome
Cocchio 2017‐ITA	Gardasil (Merck quadrivalent)	Male, ≥ 12 years*	6076 cases of AGW	Annual percent change (medium‐term; 2004‐7 vs 2008‐15)	3.8% (1.2% to 6.4%)	Unadjusted	Pre‐ vs post‐vaccine introduction; *age at outcome
Cocchio 2017‐ITA	Gardasil (Merck quadrivalent)	Female, ≥ 12 years*	6076 cases of AGW	Annual percent change (medium‐term; 2004‐7 vs 2008‐15)	‐6.1% (‐8.4% to ‐3.7%)	Unadjusted	Pre‐ vs post‐vaccine introduction; *age at outcome
Dominiak‐Felden 2015‐BEL	Gardasil (Merck quadrivalent)	Female, 10 to 23 years	Pre‐vaccine: 907,047 Post‐vaccine: 1,284,493	Incidence rate ratio (long‐term; 2006 vs 2009‐13)	0.28 (0.22 to 0.35)	Age and gender	Pre‐ vs post‐vaccine introduction
Fernandes 2021‐PRT	Gardasil (Merck quadrivalent)	Female, ≤ 19 years*	NR	Relative change (medium‐term; 2008 vs 2017)	‐86.8%	Unadjusted	Pre‐ vs post‐vaccine introduction; *age at outcome
Fernandes 2021‐PRT	Gardasil (Merck quadrivalent)	Female, 20 to 24 years*	NR	Relative change (long‐term; 2008 vs 2017)	‐77.4%	Unadjusted	Pre‐ vs post‐vaccine introduction; *age at outcome
Fernandes 2021‐PRT	Gardasil (Merck quadrivalent)	Male, ≤ 19 years*	NR	Relative change (medium‐term; 2008 vs 2017)	‐38.5%	Unadjusted	Pre‐ vs post‐vaccine introduction; *age at outcome
Fernandes 2021‐PRT	Gardasil (Merck quadrivalent)	Male, 20 to 24 years*	NR	Relative change (long‐term; 2008 vs 2017)	‐19.3%	Unadjusted	Pre‐ vs post‐vaccine introduction; *age at outcome
Flagg 2018‐USA	Gardasil (Merck quadrivalent)	Female, 15 to 39 years	88,911,951 person‐years	Annual percent change (medium‐term; 2006 vs 2009)	5.6% (‐3.8% to 16.0%)	Unadjusted	Pre‐ vs post‐vaccine introduction
Flagg 2018‐USA	Gardasil (Merck quadrivalent)	Female, 15 to 39 years	88,911,951 person‐years	Annual percent change (medium‐term; 2009 vs 2014)	‐6.2% (‐9.0% to ‐3.3%)	Unadjusted	Pre‐ vs post‐vaccine introduction
Flagg 2018‐USA	Gardasil (Merck quadrivalent)	Male, 15 to 39 years	88,911,951 person‐years	Annual percent change (medium‐term; 2006 vs 2009)	16.5% (8.7% to 24.8%)	Unadjusted	Pre‐ vs post‐vaccine introduction
Flagg 2018‐USA	Gardasil (Merck quadrivalent)	Male, 15 to 39 years	88,911,951 person‐years	Annual percent change (medium‐term; 2009 vs 2014)	2.4% (0.5% to 4.3%)	Unadjusted	Pre‐ vs post‐vaccine introduction
Goodman 2024‐DEU	Cervarix (GSK bivalent); Gardasil (Merck quadrivalent); Gardasil 9 (Merck nonavalent)	Female, 28 to 33 years*	N = 61,520	Relative risk (long‐term)	0.60 (0.46 to 0.79)	Unadjusted	Pre‐ vs post‐vaccine introduction; *age at outcome
Guerra 2016‐CAN	Gardasil (Merck quadrivalent)	Females, 12 to 13 years	NR	Incidence rate ratio (long‐term; 2004 vs 2013)	1.02 (0.78 to 1.33)	Pap‐test rate	Pre‐ vs post‐vaccine introduction
Herweijer 2018‐SWE	Gardasil (Merck quadrivalent)	Female, 15 to 19 years*	NR	Annual percent change (long‐term; 2006‐7 vs 2010‐12)	2006‐7: 2.8% (‐5.5% to 11.8%) 2010‐12: ‐18.6% (‐22.8% to ‐14.1%)	Calendar year, sex and 5‐year age categories	Pre‐ vs post‐vaccine introduction; *age at outcome
Herweijer 2018‐SWE	Gardasil (Merck quadrivalent)	Female, 20 to 24 years*	NR	Annual percent change (long‐term; 2006‐7 vs 2010‐12)	2006‐7: 0.4% (‐3.5% to 4.4%) 2010‐12: ‐11.3% (‐13.5% to ‐9.1%)	Calendar year, sex and 5‐year age categories	Pre‐ vs post‐vaccine introduction; *age at outcome
Herweijer 2018‐SWE	Gardasil (Merck quadrivalent)	Female, 25 to 29 years*	NR	Annual percent change (long‐term; 2006‐7 vs 2010‐12)	2006‐7: ‐4.2% (‐5.0% to ‐3.4%) 2010‐12: ‐4.2% (‐5.0% to ‐3.4%)	Calendar year, sex and 5‐year age categories	Pre‐ vs post‐vaccine introduction; *age at outcome
Herweijer 2018‐SWE	Gardasil (Merck quadrivalent)	Male, 15 to 19 years*	NR	Annual percent change (long‐term; 2006‐7 vs 2010‐12)	2006‐7: 6.6% (2.4% to 10.9%) 2010‐12: ‐16.6% (‐21.7% to ‐11.1%)	Calendar year, sex and 5‐year age categories	Pre‐ vs post‐vaccine introduction; *age at outcome
Herweijer 2018‐SWE	Gardasil (Merck quadrivalent)	Male, 20 to 24 years*	NR	Annual percent change (long‐term; 2006‐7 vs 2010‐12)	2006‐7: ‐0.7% (‐2.1% to 0.6%) 2010‐12: ‐11.0% (‐14.3% to ‐7.6%)	Calendar year, sex and 5‐year age categories	Pre‐ vs post‐vaccine introduction; *age at outcome
Herweijer 2018‐SWE	Gardasil (Merck quadrivalent)	Male, 25 to 29 years*	NR	Annual percent change (long‐term; 2006‐7 vs 2010‐12)	2006‐7: 0.5% (‐2.1% to 3.2%) 2010‐12: ‐7.0% (‐13.2% to ‐0.4%)	Calendar year, sex and 5‐year age categories	Pre‐ vs post‐vaccine introduction; *age at outcome
Judlin 2016‐FRA	Gardasil (Merck quadrivalent)	Female, 15 to 26 years	Pre‐vaccine: 39,190 Post‐vaccine: 45,628	Incidence rate ratio (medium‐term; 2008‐9 vs 2011‐12)	1.12 (0.91 to 1.37)	Unadjusted	Pre‐ vs post‐vaccine introduction
Kury 2013‐BRA	Gardasil (Merck quadrivalent)	Female, 12 to 20 years	NR	Incidence rate ratio (long‐term; 2007 vs 2012)	0.50 (0.25 to 0.96)	Unadjusted	Pre‐ vs post‐vaccine introduction
Liu 2014‐AUS	Gardasil (Merck quadrivalent)	Female, 18 to 39 years*	Pre‐vaccine: 4862 Post‐vaccine: 2363	Odds ratio (long‐term; 2001 vs 2011)	1.10 (0.78 to 1.54)	Age, place of residence, country of birth, Aboriginal or Torres Strait Islander status, education level, self‐reporting of chlamydia	Pre‐ vs post‐vaccine introduction, *age at outcome
Lukac 2020‐CAN	Gardasil (Merck quadrivalent)	Female and male, 20 to 28 years*	N = 85,158	Relative risk (long‐term; birth cohort 1994‐6 vs 1991‐3)	0.44 (0.34 to 0.59)	Age and period	Pre‐ vs post‐vaccine introduction, *age at outcome
Lurie 2017‐ISR	Gardasil (Merck quadrivalent)	Female, 9 to 45 years	Pre‐vaccine: 293,240 Post‐vaccine: 323,436	Odds ratio (medium‐term; 2006 vs 2015)	0.48 (0.38 to 0.60)	Unadjusted	Pre‐ vs post‐vaccine introduction; ≤ 18 at outcome
Lurie 2017‐ISR	Gardasil (Merck quadrivalent)	Female, 9 to 45 years	Pre‐vaccine: 143,955 Post‐vaccine: 133,917	Odds ratio (long‐term; 2006 vs 2015)	0.75 (0.71 to 0.80)	Unadjusted	Pre‐ vs post‐vaccine introduction; 25 to 34 at outcome
Lurie 2017‐ISR	Gardasil (Merck quadrivalent)	Male, 9 to 45 years	Pre‐vaccine: 310,339 Post‐vaccine: 342,190	Odds ratio (medium‐term; 2006 vs 2015)	0.59 (0.45 to 0.77)	Unadjusted	Pre‐ vs post‐vaccine introduction; ≤ 18 at outcome
Lurie 2017‐ISR	Gardasil (Merck quadrivalent)	Male, 9 to 45 years	Pre‐vaccine: 123,476 Post‐vaccine: 125,751	Odds ratio (long‐term; 2006 vs 2015)	0.96 (0.91 to 1.01)	Unadjusted	Pre‐ vs post‐vaccine introduction; 25 to 34 at outcome
Mann 2019‐USA	Gardasil (Merck quadrivalent)	Male, all ages	Pre‐vaccine: 96,243 Post‐vaccine: 185,844	Annual percent change (long‐term; 2010 vs 2016)	‐8.1% (‐10.4% to ‐6.1%)	Jurisdiction	Pre‐ vs post‐vaccine introduction
Naleway 2020‐USA	Gardasil (Merck quadrivalent)	Female, 11 to 26 years	N = 565,356	Incidence rate ratio (long‐term; 2000‐6 vs 2007‐16)	0.69 (0.65 to 0.75)	Baseline level and trend in AGW incidence	Pre‐ vs post‐vaccine introduction
Naleway 2020‐USA	Gardasil (Merck quadrivalent)	Male, 11 to 21 years	N = 565,356	Incidence rate ratio (long‐term; 2000‐10 vs 2011‐16)	0.90 (0.84 to 0.97)	Baseline level and trend in AGW incidence	Pre‐ vs post‐vaccine introduction
Nsouli‐Maktabi 2013‐USA	Gardasil (Merck quadrivalent)	Female, NR	Pre‐vaccine: 1,544,029 Post‐vaccine: 1,440,362	Incidence rate ratio (long‐term; 2005 vs 2012)	0.83 (0.82 to 0.85)	Unadjusted	Pre‐ vs post‐vaccine introduction
Nsouli‐Maktabi 2013‐USA	Gardasil (Merck quadrivalent)	Male, NR	Pre‐vaccine: 1,544,029 Post‐vaccine: 1,440,362	Incidence rate ratio (long‐term; 2005 vs 2012)	1.37 (1.34 to 1.39)	Unadjusted	Pre‐ vs post‐vaccine introduction
Oliphant 2011‐NZL	Gardasil (Merck quadrivalent)	Female and male, 11 to 20 years	Pre‐vaccine: 21,739 Post‐vaccine: 19,054	Incidence rate ratio (medium‐term; 2007 vs 2010)	0.82 (0.77 to 0.89)	Unadjusted	Pre‐ vs post‐vaccine introduction
Orumaa 2020‐NOR/DNK	Gardasil (Merck quadrivalent)	Female, 12 to 26 years	Pre‐vaccine: 693,534 Post‐vaccine: 789,550	Annual percent change (long‐term; 2009 vs 2015)	‐4.8% (‐5.3% to ‐4.3%)	Age‐standardised	Pre‐ vs post‐vaccine introduction; Norway
Orumaa 2020‐NOR/DNK	Gardasil (Merck quadrivalent)	Male, 12 to 26 years	Pre‐vaccine: 830,930 Post‐vaccine: 724,268	Annual percent change (long‐term; 2009 vs 2015)	‐1.9% (‐2.4% to ‐1.4%)	Age‐standardised	Pre‐ vs post‐vaccine introduction; Norway
Orumaa 2020‐NOR/DNK	Gardasil (Merck quadrivalent)	Female, 12 to 26 years	Pre‐vaccine: 817,222 Post‐vaccine: 801,125	Annual percent change (long‐term; 2009 vs 2015)	‐18.0% (‐18.6% to ‐17.5%)	Age‐standardised	Pre‐ vs post‐vaccine introduction; Denmark
Orumaa 2020‐NOR/DNK	Gardasil (Merck quadrivalent)	Male, 12 to 26 years	Pre‐vaccine: 848,038 Post‐vaccine: 824,729	Annual percent change (long‐term; 2009 vs 2015)	‐10.7% (‐11.2% to ‐10.3%)	Age‐standardised	Pre‐ vs post‐vaccine introduction; Denmark
Perkins 2015‐USA	Gardasil (Merck quadrivalent)	Female, 16 to 26 years	Pre‐vaccine: 32,834 Post‐vaccine: 33,007	Diagnosis rate trend (long‐term; 2011‐2013)	‐22.1%	Unadjusted	Pre‐ vs post‐vaccine introduction
Perkins 2015‐USA	Gardasil (Merck quadrivalent)	Male, 16 to 26 years	Pre‐vaccine: 32,834 Post‐vaccine: 33,007	Diagnosis rate trend (long‐term; 2011‐2013)	‐13.5%	Unadjusted	Pre‐ vs post‐vaccine introduction
Restivo 2023‐ITA	NR	Female and male, age NR	N = 59,449 cases	Rate ratio (2008 vs 2018)	0.67 (0.50 to 0.89)	Unadjusted	Pre‐ vs post‐vaccine introduction
Sando 2014‐DNK	Gardasil (Merck quadrivalent)	Female, 15 to 19 years*	Pre‐vaccine: 164,754 Post‐vaccine: 173,448	Incidence rate ratio (medium‐term; 2008 vs 2011)	0.31 (0.29 to 0.34)	Unadjusted	Pre‐ vs post‐vaccine introduction; *age at outcome
Sando 2014‐DNK	Gardasil (Merck quadrivalent)	Female, 20 to 24 years*	Pre‐vaccine: 150,760 Post‐vaccine: 166,608	Incidence rate ratio (long‐term; 2008 vs 2011)	0.83 (0.79 to 0.87)	Unadjusted	Pre‐ vs post‐vaccine introduction; *age at outcome
Sando 2014‐DNK	Gardasil (Merck quadrivalent)	Female, 25 to 29 years*	Pre‐vaccine: 157,405 Post‐vaccine: 155,686	Incidence rate ratio (long‐term; 2008 vs 2011)	1.03 (0.96 to 1.10)	Unadjusted	Pre‐ vs post‐vaccine introduction; *age at outcome
Sando 2014‐DNK	Gardasil (Merck quadrivalent)	Female, 30 to 34 years*	Pre‐vaccine: 181,587 Post‐vaccine: 167,953	Incidence rate ratio (long‐term; 2008 vs 2011)	0.98 (0.89 to 1.07)	Unadjusted	Pre‐ vs post‐vaccine introduction; *age at outcome
Shing 2019‐USA	Gardasil (Merck quadrivalent)	Female, 15 to 19 years*	Pre‐vaccine: 303,825 person‐years Post‐vaccine: 2,461,739 person‐years	Annual percent change (long‐term; 2006 vs 2014)	‐10.6% (‐12.6% to ‐8.5%)	Unadjusted	Pre‐ vs post‐vaccine introduction; *age at outcome
Shing 2019‐USA	Gardasil (Merck quadrivalent)	Female, 20 to 24 years*	Pre‐vaccine: 303,825 person‐years Post‐vaccine: 2,461,739 person‐years	Annual percent change (long‐term; 2006 vs 2014)	‐3.9% (‐7.1% to ‐0.6%)	Unadjusted	Pre‐ vs post‐vaccine introduction; *age at outcome
Shing 2019‐USA	Gardasil (Merck quadrivalent)	Female, 25 to 29 years*	Pre‐vaccine: 303,825 person‐years Post‐vaccine: 2,461,739 person‐years	Annual percent change (long‐term; 2006 vs 2014)	5.2% (0.3% to 10.3%)	Unadjusted	Pre‐ vs post‐vaccine introduction; *age at outcome
Shing 2019‐USA	Gardasil (Merck quadrivalent)	Female, 30 to 39 years*	Pre‐vaccine: 303,825 person‐years Post‐vaccine: 2,461,739 person‐years	Annual percent change (long‐term; 2006 vs 2014)	6.5% (‐4.7% to 18.9%)	Unadjusted	Pre‐ vs post‐vaccine introduction; *age at outcome
Shing 2019‐USA	Gardasil (Merck quadrivalent)	Male, 15 to 19 years*	Pre‐vaccine: 303,825 person‐years Post‐vaccine: 2,461,739 person‐years	Annual percent change (long‐term; 2006 vs 2014)	4.4% (‐11.4% to 22.9%)	Unadjusted	Pre‐ vs post‐vaccine introduction; *age at outcome
Shing 2019‐USA	Gardasil (Merck quadrivalent)	Male, 20 to 24 years*	Pre‐vaccine: 303,825 person‐years Post‐vaccine: 2,461,739 person‐years	Annual percent change (long‐term; 2006 vs 2014)	5.9% (‐0.4% to 12.6%)	Unadjusted	Pre‐ vs post‐vaccine introduction; *age at outcome
Shing 2019‐USA	Gardasil (Merck quadrivalent)	Male, 25 to 29 years*	Pre‐vaccine: 303,825 person‐years Post‐vaccine: 2,461,739 person‐years	Annual percent change (long‐term; 2006 vs 2014)	10.0% (5.7% to 14.6%)	Unadjusted	Pre‐ vs post‐vaccine introduction; *age at outcome
Shing 2019‐USA	Gardasil (Merck quadrivalent)	Male, 30 to 39 years*	Pre‐vaccine: 303,825 person‐years Post‐vaccine: 2,461,739 person‐years	Annual percent change (long‐term; 2006 vs 2014)	4.1% (‐3.1% to 11.9%)	Unadjusted	Pre‐ vs post‐vaccine introduction; *age at outcome
Smith 2016‐AUS	Gardasil (Merck quadrivalent)	Female, 12 to 69 years	Pre‐vaccine: 18,751 Post‐vaccine: 6060	Incidence rate ratio (long‐term; 1999‐2008 vs 2007‐2011	0.59 (0.48 to 0.73)	Unadjusted	Pre‐ vs post‐vaccine introduction
Smith 2016‐AUS	Gardasil (Merck quadrivalent)	Male, 12 to 69 years	Pre‐vaccine: 18,751 Post‐vaccine: 6060	Incidence rate ratio (long‐term; 1999‐2008 vs 2007‐2011	0.90 (0.69 to 1.17)	Unadjusted	Pre‐ vs post‐vaccine introduction
Sonnenberg 2019‐GBR	Cervarix (GSK bivalent)	Female, 16 to 44 years*	Vaccinated: 5257 Unvaccinated: 5869	Prevalence ratio (long‐term; 1999‐2001 vs 2010‐2012	1.10 (0.71 to 1.71)	Unadjusted	Pre‐ vs post‐vaccine introduction; *age at outcome
Sonnenberg 2019‐GBR	Cervarix (GSK bivalent)	Male, 16 to 44 years*	Vaccinated: 3570 Unvaccinated: 4267	Prevalence ratio (long‐term; 1999‐2001 vs 2010‐2012	1.02 (0.64 to 1.66)	Unadjusted	Pre‐ vs post‐vaccine introduction; *age at outcome
Steben 2018‐CAN	Gardasil (Merck quadrivalent)	Female, 9 to 17 years	Pre‐vaccine: 11,098 Post‐vaccine: 10,313	Incidence rate ratio (long‐term; 2004‐7 vs 2009‐12)	0.82 (0.74 to 0.92)	Age	Pre‐ vs post‐vaccine introduction
Steben 2018‐CAN	Gardasil (Merck quadrivalent)	Male, 9 to 17 years	Pre‐vaccine: 11,098 Post‐vaccine: 10,313	Incidence rate ratio (long‐term; 2004‐7 vs 2009‐12)	0.95 (0.86 to 1.04)	Age	Pre‐ vs post‐vaccine introduction
Thompson 2016‐CAN	Gardasil (Merck quadrivalent)	Female, 11 to 12 years	NR	Odds ratio (long‐term; 1990‐94 vs 2010‐11)	0.77 (0.72 to 0.82)	Age group, geographic residential area category and income quintile	Pre‐ vs post‐vaccine introduction
Thompson 2016‐CAN	Gardasil (Merck quadrivalent)	Male, NR	NR	Odds ratio (long‐term; 1990‐94 vs 2010‐11)	1.24 (1.17 to 2.01)	Age group, geographic residential area category and income quintile	Pre‐ vs post‐vaccine introduction
Thöne 2017‐DEU	Cervarix (GSK bivalent); Gardasil (Merck quadrivalent)	Male, 11 to 79 years*	Pre‐vaccine: 4,370,000 person‐years Post‐vaccine: 2,330,000 person‐years	Incidence rate ratio (medium‐term; 2005 vs 2010)	1.18 (1.07 to 1.24)	Unadjusted	Pre‐ vs post‐vaccine introduction; *age at outcome
Thöne 2017‐DEU	Cervarix (GSK bivalent); Gardasil (Merck quadrivalent)	Female, 11 to 79 years*	Pre‐vaccine: 2,040,000 person‐years Post‐vaccine: 2,680,000 person‐years	Incidence rate ratio (medium‐term; 2005 vs 2010)	0.88 (0.83 to 0.94)	Unadjusted	Pre‐ vs post‐vaccine introduction; *age at outcome

AGW: anogenital warts; NR: not reported

**53:** Risk of bias summary: anogenital warts

**Study**	**Confounding**	**Selection**	**Classification of interventions**	**Deviations from intended interventions**	**Missing data**	**Measurement of outcomes**	**Selection of reported result**	**Overall risk of bias**
Baandrup 2021‐DNK	Serious	Low	Low	Low	Low	Low	Low	Serious
Cho 2024‐KOR	Serious	Low	Low	Low	Moderate	Low	Low	Serious
Dominiak‐Felden 2015‐BEL	Serious	Moderate	Low	Low	Moderate	Moderate	Moderate	Serious
Hariri 2018‐USA	Moderate	Low	Low	Low	Low	Low	Low	Moderate
Herweijer 2018‐SWE	Serious	Low	Low	Low	Moderate	Low	Low	Serious
Howell‐Jones 2013‐GBR	Serious	Moderate	Moderate	Low	Low	Low	Low	Serious
Munoz‐Quiles 2021‐ESP	Serious	Low	Low	Low	Low	Low	Serious	Serious
Nygard 2023‐NOR	Serious	Low	Low	Low	Low	Low	Low	Serious
Osmani 2022‐DEU	Serious	Low	Low	Low	Low	Low	Low	Serious
Perkins 2017‐USA	Serious	Low	Low	Low	Low	Low	Low	Serious
Reyburn 2023‐FJI	Serious	Low	Low	Low	Moderate	Low	Low	Serious
Swedish 2013‐USA	Serious	Low	Low	Low	Low	Low	Low	Serious
Willows 2018‐CAN	Serious	Low	Low	Low	Low	Low	Low	Serious
Woestenberg 2020‐NLD	Serious	Low	Low	Low	Moderate	Low	Low	Serious
Zeybek 2018‐USA	Serious	Low	Low	Low	Low	Low	Low	Serious
Krasnopolsky 2020‐RUS	Critical	Critical	Low	Moderate	Low	Low	Low	Critical
Petras 2015‐CZE	Critical	Moderate	Moderate	Low	Low	Low	Low	Critical
Sadler 2015‐GBR	Critical	Moderate	Moderate	Low	Moderate	Moderate	Low	Critical
Ali 2013‐AUS	Critical	Moderate	Low	Low	Low	Low	Low	Critical
Bauer 2012‐USA	Critical	Serious	Serious	Moderate	Moderate	Low	Low	Critical
Canvin 2017‐GBR	Critical	Moderate	Serious	Low	Low	Low	Low	Critical
Chow 2021b‐AUS	Critical	Moderate	Serious	Low	Moderate	Low	Low	Critical
Chow 2019‐AUS	Critical	Serious	Serious	Moderate	Low	Low	Low	Critical
Cocchio 2017‐ITA	Serious	Serious	Serious	Moderate	Low	Low	Moderate	Serious
Dominiak‐Felden 2015‐BEL	Serious	Moderate	Low	Low	Moderate	Moderate	Moderate	Serious
Fernandes 2021‐PRT	Critical	Moderate	Serious	Moderate	Low	Low	Low	Critical
Flagg 2018‐USA	Critical	Low	Serious	Low	Low	Low	Low	Critical
Goodman 2024‐DEU	Critical	Low	Serious	Low	Low	Low	Low	Critical
Guerra 2016‐CAN	Critical	Low	Serious	Low	Low	Low	Low	Critical
Herweijer 2018‐SWE	Serious	Low	Low	Low	Moderate	Low	Low	Serious
Judlin 2016‐FRA	Critical	Moderate	Serious	Low	Low	Low	Low	Critical
Kury 2013‐BRA	Critical	Moderate	Serious	Low	Low	Low	Low	Critical
Liu 2014‐AUS	Serious	Low	Serious	Low	Low	Low	Low	Serious
Lukac 2020‐CAN	Serious	Low	Low	Low	Low	Low	Moderate	Serious
Lurie 2017‐ISR	Critical	Serious	Serious	Low	Low	Serious	Moderate	Critical
Mann 2019‐USA	Serious	Moderate	Serious	Low	Low	Low	Serious	Serious
Naleway 2020‐USA	Serious	Low	Serious	Low	Low	Low	Moderate	Serious
Nsouli‐Maktabi 2013‐USA	Critical	Moderate	Serious	Low	Low	Low	Low	Critical
Oliphant 2011‐NZL	Critical	Moderate	Serious	Low	Low	Low	Low	Critical
Orumaa 2020‐NOR/DNK	Serious	Low	Serious	Low	Low	Low	Low	Serious
Perkins 2015‐USA	Serious	Moderate	Low	Low	Low	Low	Low	Serious
Restivo 2023‐ITA	Critical	Serious	Serious	Low	Low	Low	Low	Critical
Sando 2014‐DNK	Serious	Low	Serious	Low	Moderate	Low	Low	Serious
Shing 2019‐USA	Critical	Low	Serious	Low	Low	Low	Low	Critical
Smith 2016‐AUS	Critical	Serious	Serious	Low	Low	Low	Low	Critical
Sonnenberg 2019‐GBR	Critical	Low	Serious	Low	Moderate	Moderate	Low	Critical
Steben 2018‐CAN	Critical	Serious	Serious	Low	Low	Low	Low	Critical
Thompson 2016‐CAN	Serious	Low	Serious	Low	Low	Low	Low	Serious
Thöne 2017‐DEU	Critical	Moderate	Serious	Low	Low	Low	Low	Critical

Forty‐seven studies were identified that reported on anogenital warts following HPV vaccination (Ali 2013‐AUS; Baandrup 2021‐DNK; Bauer 2012‐USA; Canvin 2017‐GBR; Cho 2024‐KOR; Chow 2019‐AUS; Chow 2021b‐AUS; Cocchio 2017‐ITA; Dominiak‐Felden 2015‐BEL; Fernandes 2021‐PRT; Flagg 2018‐USA; Goodman 2024‐DEU; Guerra 2016‐CAN; Hariri 2018‐USA; Herweijer 2018‐SWE; Howell‐Jones 2013‐GBR; Judlin 2016‐FRA; Krasnopolsky 2020‐RUS; Kury 2013‐BRA; Liu 2014‐AUS; Lukac 2020‐CAN; Lurie 2017‐ISR; Mann 2019‐USA; Munoz‐Quiles 2021‐ESP; Naleway 2020‐USA; Nsouli‐Maktabi 2013‐USA; Nygard 2023‐NOR; Oliphant 2011‐NZL; Orumaa 2020‐NOR/DNK; Osmani 2022‐DEU; Perkins 2015‐USA; Perkins 2017‐USA; Petras 2015‐CZE; Restivo 2023‐ITA; Reyburn 2023‐FJI; Sadler 2015‐GBR; Sando 2014‐DNK; Shing 2019‐USA; Smith 2016‐AUS; Sonnenberg 2019‐GBR; Steben 2018‐CAN; Swedish 2013‐USA; Thompson 2016‐CAN; Thöne 2017‐DEU; Willows 2018‐CAN; Woestenberg 2020‐NLD; Zeybek 2018‐USA).

Fifteen were cohort studies (Baandrup 2021‐DNK; Cho 2024‐KOR; Dominiak‐Felden 2015‐BEL; Hariri 2018‐USA; Herweijer 2018‐SWE; Howell‐Jones 2013‐GBR; Munoz‐Quiles 2021‐ESP; Nygard 2023‐NOR; Osmani 2022‐DEU; Perkins 2017‐USA; Reyburn 2023‐FJI; Swedish 2013‐USA; Willows 2018‐CAN; Woestenberg 2020‐NLD; Zeybek 2018‐USA), three were cross‐sectional (Krasnopolsky 2020‐RUS; Petras 2015‐CZE; Sadler 2015‐GBR), and 29 were pre‐post vaccine introduction studies (Ali 2013‐AUS; Bauer 2012‐USA; Canvin 2017‐GBR; Chow 2021b‐AUS; Chow 2019‐AUS; Cocchio 2017‐ITA; Fernandes 2021‐PRT; Flagg 2018‐USA; Goodman 2024‐DEU; Guerra 2016‐CAN; Judlin 2016‐FRA; Kury 2013‐BRA; Liu 2014‐AUS; Lukac 2020‐CAN; Lurie 2017‐ISR; Mann 2019‐USA; Naleway 2020‐USA; Nsouli‐Maktabi 2013‐USA; Oliphant 2011‐NZL; Orumaa 2020‐NOR/DNK; Perkins 2015‐USA; Restivo 2023‐ITA; Sando 2014‐DNK; Shing 2019‐USA; Smith 2016‐AUS; Sonnenberg 2019‐GBR; Steben 2018‐CAN; Thompson 2016‐CAN; Thöne 2017‐DEU). Two of the cohort studies also reported incidence over time using the pre‐post vaccine introduction design (Dominiak‐Felden 2015‐BEL; Herweijer 2018‐SWE).

From the cohort studies, the pooled estimate of the impact of HPV vaccination on rates of anogenital warts indicated a reduction of 47% in the medium term (RR 0.53, 95% CI 0.37 to 0.77; 4 studies, 6,430,295 females and 313 males; I^2^ = 98%) (Analysis 3.2) and 53% in the long term (RR 0.47, 95% CI 0.36 to 0.61; 13 studies, 4.5 million person‐years plus 5,802,969 females and males; I^2^ = 99%) (Analysis 3.2). An analysis restricted to those receiving an HPV vaccine at or before the age of 16 years showed a reduction of anogenital warts incidence of 40% in the medium term (RR 0.60, 95% CI 0.30 to 1.21; 3 studies, 3,837,215 females; I^2^ = 99%) and 70% in the long term (RR 0.30, 95% CI 0.20 to 0.43; 6 studies, 3,647,319 person‐years plus 1,874,676 females and males; I^2^ = 97%) (Analysis 3.3).

Of the three cross‐sectional studies (Krasnopolsky 2020‐RUS; Petras 2015‐CZE; Sadler 2015‐GBR), one did not report any cases of anogenital warts in the HPV vaccine‐exposed group (Krasnopolsky 2020‐RUS). The other two studies reported a decreased risk of anogenital warts following HPV vaccination, but with confidence intervals that included no difference.

Of the 31 pre‐post vaccine introduction studies, seven reported only on females (Dominiak‐Felden 2015‐BEL; Goodman 2024‐DEU; Guerra 2016‐CAN; Judlin 2016‐FRA; Kury 2013‐BRA; Liu 2014‐AUS; Sando 2014‐DNK), two reported only on males (Chow 2019‐AUS; Mann 2019‐USA), and 22 reported on both (Ali 2013‐AUS; Bauer 2012‐USA; Canvin 2017‐GBR; Chow 2021b‐AUS; Cocchio 2017‐ITA; Fernandes 2021‐PRT; Flagg 2018‐USA; Herweijer 2018‐SWE; Lukac 2020‐CAN; Lurie 2017‐ISR; Naleway 2020‐USA; Nsouli‐Maktabi 2013‐USA; Oliphant 2011‐NZL; Orumaa 2020‐NOR/DNK; Perkins 2015‐USA; Restivo 2023‐ITA; Shing 2019‐USA; Smith 2016‐AUS; Sonnenberg 2019‐GBR; Steben 2018‐CAN; Thompson 2016‐CAN; Thöne 2017‐DEU).

In females, 23 studies (79%) reported a decrease in anogenital warts incidence over time and 6 (22%) reported either an increase or a decrease, but with confidence intervals that included no difference. In males, 12 studies (52%) reported a decrease in anogenital warts incidence over time and 11 (48%) reported either an increase or a decrease, but with confidence intervals that included no difference.

Eight cohort studies (Baandrup 2021‐DNK; Dominiak‐Felden 2015‐BEL; Hariri 2018‐USA; Herweijer 2018‐SWE; Munoz‐Quiles 2021‐ESP; Willows 2018‐CAN; Woestenberg 2020‐NLD; Zeybek 2018‐USA) and one cross‐sectional study (Petras 2015‐CZE) reported on the effectiveness of two doses or one dose of HPV vaccine. Six of the cohort studies reported a reduction in anogenital warts following two doses of HPV vaccine, though the effectiveness appeared to vary depending on age at vaccination (Baandrup 2021‐DNK; Dominiak‐Felden 2015‐BEL; Hariri 2018‐USA; Herweijer 2018‐SWE; Munoz‐Quiles 2021‐ESP; Zeybek 2018‐USA). Four of the studies also reported a reduction in anogenital warts following one dose of HPV vaccine (Baandrup 2021‐DNK; Herweijer 2018‐SWE; Munoz‐Quiles 2021‐ESP; Zeybek 2018‐USA).

##### Pregnancy and neonatal outcomes

See Table for effect estimates and Table for the risk of bias summary of included studies on pregnancy and neonatal outcomes.

**54:** Secondary clinical outcomes effect estimates: pregnancy and neonatal outcomes

**Study**	**Vaccine**	**Population (sex, age)**	**Sample size**	**Effect measure (time period)**	**Effect estimate**	**Adjustment factors**	**Notes**
Baril 2015‐GBR	Cervarix (GSK bivalent)	Female, 14 to 23 years	Vaccinated: 207 Unvaccinated: 632	Hazard ratio (short‐term)	1.34 (0.81 to 2.24)	Age at first day of gestation, smoking, alcohol consumption, gestation start during the H1N1 pandemic season, general practice region, diabetes and high blood pressure during pregnancy, number of previous pregnancies, vaccination with another vaccine from −90 to +90 days gestation, and use of contraindicated drugs during the first trimester of gestation	Cohort; spontaneous abortion during the first 23 weeks of gestation
Baril 2015‐GBR	Cervarix (GSK bivalent)	Female, 14 to 23 years	Vaccinated: 207 Unvaccinated: 632	Odds ratio (short‐term)	2.29 (0.51 to 10.32)	Unadjusted	Cohort; stillbirth
Baril 2015‐GBR	Cervarix (GSK bivalent)	Female, 14 to 23 years	Vaccinated: 207 Unvaccinated: 632	Odds ratio (short‐term)	0.67 (0.28 to 1.67)	Unadjusted	Cohort; preterm delivery
Baril 2015‐GBR	Cervarix (GSK bivalent)	Female, 14 to 23 years	Vaccinated: 207 Unvaccinated: 632	Odds ratio (short‐term)	0.89 (0,29 to 2.71)	Age at first day of gestation	Cohort; major birth defects
Bukowinski 2020‐USA	Gardasil (Merck quadrivalent)	Female, 17 to 28 years	Vaccinated: 1775 Unvaccinated: 88,825	Hazard ratio (short‐term)	1.05 (0.94 to 1.18)	Maternal age, race/ethnicity, military rank, marital status, receipt of vaccines not routinely recommended in pregnancy and receipt of prenatal care	Cohort; spontaneous abortion
Bukowinski 2020‐USA	Gardasil (Merck quadrivalent)	Female, 17 to 28 years	Vaccinated: 1775 Unvaccinated: 88,825	Hazard ratio (short‐term)	0.92 (0.76 to 1.13)	Maternal age, race/ethnicity, military rank, marital status, receipt of vaccines not routinely recommended in pregnancy and receipt of prenatal care	Cohort; preterm labour/delivery
Bukowinski 2020‐USA	Gardasil (Merck quadrivalent)	Female, 17 to 28 years	Vaccinated: 1775 Unvaccinated: 88,825	Relative risk (short‐term)	0.67 (0.47 to 0.96)	Maternal age, race/ethnicity, military rank, marital status, receipt of vaccines not routinely recommended in pregnancy and receipt of prenatal care	Cohort; any structural birth defect
Faber 2019‐DNK	Gardasil (Merck quadrivalent)	Female, 14 to 39 years	Vaccinated: 5160 Unvaccinated: 309,010	Odds ratio (short‐term)	0.96 (0.57 to 1.61)	Age at conception, education, smoking and BMI	Cohort; stillbirth
Faber 2019‐DNK	Gardasil (Merck quadrivalent)	Female, 14 to 39 years	Vaccinated: 5145 Unvaccinated: 308,062	Hazard ratio (short‐term)	0.94 (0.53 to 1.67)	Age at conception, education, smoking and BMI	Cohort; infant mortality
Faber 2019‐DNK	Gardasil (Merck quadrivalent)	Female, 14 to 39 years	Vaccinated: 6710 Unvaccinated: 466,883	Rate ratio (short‐term)	1.08 (0.87 to 1.34)	Age at conception, birth year of the woman, education, marital status, ethnicity, number of previous births, number of previous spontaneous and induced abortions, history of genital warts, chlamydia and pelvic inflammatory disease	Cohort; spontaneous abortion within the first 7 weeks
Scheller 2017‐DNK	Gardasil (Merck quadrivalent)	Female, 12 to 27 years	Vaccinated: 1665 Unvaccinated: 6660	Prevalence odds ratio (short‐term)	1.19 (0.90 to 1.58)	Matched on age, calendar year of pregnancy onset and propensity score (age at pregnancy onset, place of birth, married or living with partner, level of education, household income, pregnancy history, smoking, body mass index, medical history, health care utilisation	Cohort; major birth defect
Scheller 2017‐DNK	Gardasil (Merck quadrivalent)	Female, 12 to 27 years	Vaccinated: 463 Unvaccinated: 1852	Hazard ratio (short‐term)	0.71 (0.45 to 1.14)	Matched on age, calendar year of pregnancy onset and propensity score (age at pregnancy onset, place of birth, married or living with partner, level of education, household income, pregnancy history, smoking, body mass index, medical history, health care utilisation	Cohort; spontaneous abortion
Scheller 2017‐DNK	Gardasil (Merck quadrivalent)	Female, 12 to 27 years	Vaccinated: 1774 Unvaccinated: 7096	Prevalence odds ratio (short‐term)	1.15 (0.93 to 1.42)	Matched on age, calendar year of pregnancy onset and propensity score (age at pregnancy onset, place of birth, married or living with partner, level of education, household income, pregnancy history, smoking, body mass index, medical history, health care utilisation	Cohort; preterm birth
Scheller 2017‐DNK	Gardasil (Merck quadrivalent)	Female, 12 to 27 years	Vaccinated: 501 Unvaccinated: 2004	Hazard ratio (short‐term)	2.43 (0.45 to 13.21)	Matched on age, calendar year of pregnancy onset and propensity score (age at pregnancy onset, place of birth, married or living with partner, level of education, household income, pregnancy history, smoking, body mass index, medical history, health care utilisation	Cohort; stillbirth
Kalliala 2021‐FIN	Cervarix (GSK bivalent)	Female, 15 to 22 years	Vaccinated: 6226 Unvaccinated: 19,849	Odds ratio (short‐term)	0.51 (0.30 to 0.87)	Unadjusted	RCT extension; preterm birth
Kreimer 2011‐CRI	Cervarix (GSK bivalent)	Female, 18 to 25 years	Vaccinated: 1365 Unvaccinated: 1783	Relative risk	1.15 (0.86 to 1.54)	Age at vaccination	RCT extension; miscarriage
Kreimer 2011‐CRI	Cervarix (GSK bivalent)	Female, 18 to 25 years	Vaccinated: 1365	Relative risk	1.06 (0.79 to 1.42)	Calendar year	RCT extension; miscarriage
Kreimer 2011‐CRI	Cervarix (GSK bivalent)	Female, 18 to 25 years	Unvaccinated: 1783	Relative risk	1.03 (0.78 to 1.35)	Age at conception	RCT extension; miscarriage
Krasnopolsky 2020‐RUS	NR	Female, 18 to 36 years	Vaccinated: 320 Unvaccinated: 120	Odds ratio (short‐term)	0.57 (0.32 to 1.03)	Unadjusted	Cross‐sectional; preterm birth
Krasnopolsky 2020‐RUS	NR	Female, 18 to 36 years	Vaccinated: 320 Unvaccinated: 120	Odds ratio (short‐term)	0.34 (0.15 to 0.80)	Unadjusted	Cross‐sectional; miscarriage
Krasnopolsky 2020‐RUS	NR	Female, 18 to 36 years	Vaccinated: 320 Unvaccinated: 120	Odds ratio (short‐term)	0.05 (0.00 to 1.05)	Unadjusted	Cross‐sectional; congenital malformations
Xu 2021‐GBR	Cervarix (GSK bivalent)	Female, 12 to 13 years	Pre‐vaccine: 5134 Post‐vaccine: 131	Odds ratio (short‐term; 2006‐16 vs 2015‐16)	0.71 (0.28 to 1.77)	Smoking during pregnancy, deprivation, marital status, BMI, parity, maternal age and year of infant delivery	Pre‐ vs post‐vaccine introduction; preterm birth

BMI: body mass index; H1N1: influenza A subtype H1N1; NR: not reported; RCT: randomised controlled trial

**55:** Risk of bias summary: pregnancy and neonatal outcomes

**Study**	**Confounding**	**Selection**	**Classification of interventions**	**Deviations from intended interventions**	**Missing data**	**Measurement of outcomes**	**Selection of reported result**	**Overall risk of bias**
Baril 2015‐GBR	Serious	Moderate	Low	Low	Low	Low	Low	Serious
Bukowinski 2020‐USA	Serious	Moderate	Low	Low	Low	Low	Low	Serious
Faber 2019‐DNK	Serious	Low	Low	Low	Moderate	Low	Low	Serious
Scheller 2017‐DNK	Serious	Low	Low	Low	Low	Low	Low	Serious
Kalliala 2021‐FIN	Serious	Moderate	Low	Low	Low	Low	Low	Serious
Kreimer 2011‐CRI	Serious	Moderate	Low	Low	Low	Low	Low	Serious
Krasnopolsky 2020‐RUS	Critical	Critical	Low	Moderate	Low	Low	Low	Critical
Xu 2021‐GBR	Serious	Low	Low	Low	Low	Low	Low	Serious

Six studies were included that reported on adverse pregnancy and neonatal outcomes following HPV vaccination (Baril 2015‐GBR; Bukowinski 2020‐USA; Faber 2019‐DNK; Krasnopolsky 2020‐RUS; Scheller 2017‐DNK; Xu 2021‐GBR).

*Foetal abnormality*

One study reported on major birth defects following HPV vaccination in 15‐ to 25‐year‐old women in the UK (Baril 2015‐GBR). There was no association between HPV vaccination and major birth defects (OR 0.89, 95% CI 0.29 to 2.71).

One study reported on structural birth defects in infants of women aged 17 to 28 years in the USA (Bukowinski 2020‐USA). A negative association was found between exposure to HPV vaccine during pregnancy and structural birth defects (1 study, 2281 events; HR 0.67, 95% CI 0.47 to 0.96).

One study reported on congenital malformations in infants of vaccinated HPV negative women and unvaccinated HPV positive women in Russia (Krasnopolsky 2020‐RUS). There were 3/120 (2.5%) congenital malformations in the unvaccinated group and 0/320 (0%) in the vaccinated group. There was no association between HPV vaccination during pregnancy and congenital malformations (OR 0.05, 95% CI 0.00 to 1.05).

One study reported on major birth defects in infants born to women who received HPV vaccination during pregnancy in Denmark (Scheller 2017‐DNK). There was no association between HPV vaccination and major birth defects (prevalence odds ratio 1.19, 95% CI 0.90 to 1.58).

*Cervical cerclage and incompetence*

No studies were identified that reported on this outcome.

*Miscarriage*

One study reported on spontaneous abortion following HPV vaccination in 15‐ to 25‐year‐old women in the UK (Baril 2015‐GBR). There was no evidence of increased risk of spontaneous abortion during the first 23 weeks of gestation (HR 1.34, 95% CI 0.81 to 2.24) when receiving HPV vaccination in a risk window 30 days prior to and 45 days following gestation.

One study reported on spontaneous abortion in women aged 17 to 28 years in the USA (Bukowinski 2020‐USA). No association was found between exposure to HPV vaccine during pregnancy and spontaneous abortion (1 study, 13,775 spontaneous abortion events; HR 1.05, 95% CI 0.94 to 1.18).

One study reported on spontaneous abortion following HPV vaccination during pregnancy in Denmark (Faber 2019‐DNK). There was no association between HPV vaccination during pregnancy and spontaneous abortion within the first seven weeks gestation (rate ratio 1.08, 95% CI 0.87 to 1.34).

One study reported on spontaneous miscarriage in vaccinated HPV‐negative women and unvaccinated HPV‐positive women in Russia (Krasnopolsky 2020‐RUS). There were 14/120 (11.7%) spontaneous miscarriages in the unvaccinated group and 15/320 (4.7%) in the vaccinated group. There was no association between HPV vaccination during pregnancy and miscarriage (OR 0.34, 95% CI 0.15 to 0.80).

One study reported on spontaneous abortion in infants born to women who received HPV vaccination during pregnancy in Denmark (Scheller 2017‐DNK). There was no association between HPV vaccination and spontaneous abortion (HR 0.71, 95% CI 0.45 to 1.14).

*Pre‐term birth*

One study reported on premature birth following HPV vaccination in 15‐ to 25‐year‐old women in the UK (Baril 2015‐GBR). There was no association between HPV vaccination and pre‐term delivery (OR 0.67, 95% CI 0.28 to 1.67).

One study reported on spontaneous preterm labour/delivery in women aged 17 to 28 years in the USA (Bukowinski 2020‐USA). No association was found between exposure to HPV vaccine during pregnancy and spontaneous preterm labour/delivery (1 study, 5603 preterm births; HR 0.92, 95% CI 0.76 to 1.13).

One study reported on preterm births in vaccinated HPV‐negative women and unvaccinated HPV‐positive women in Russia (Krasnopolsky 2020‐RUS). There were 10/120 (8.3%) preterm births in the unvaccinated group and 25/320 (7.8%) in the vaccinated group. There was no association between HPV vaccination and preterm birth (OR 0.57, 95% CI 0.32 to 1.03).

One study reported on preterm birth in infants born to women who received HPV vaccination during pregnancy in Denmark (Scheller 2017‐DNK). There was no association between HPV vaccination and preterm birth (prevalence OR 1.15, 95% CI 0.93 to 1.42).

One study reported on preterm birth in babies born in the UK (Xu 2021‐GBR). There was no association between preterm birth and routine HPV vaccination (OR 0.71, 95% CI 0.28 to 1.77).

*Perinatal mortality*

One study reported on infant mortality following HPV vaccination during pregnancy in Denmark (Faber 2019‐DNK). There was no association between HPV vaccination during pregnancy and infant mortality (HR 0.94, 95% CI 0.53 to 1.67).

*Neonatal intensive care unit (NICU) admission*

No studies reported on this outcome.

*Stillbirth*

One study reported on stillbirth following HPV vaccination in 15‐ to 25‐year‐old women in the UK (Baril 2015‐GBR). There were seven stillbirths, three in the exposed and four in the non‐exposed cohort. There was no association between HPV vaccination during pregnancy and stillbirth (OR 2.29, 95% CI 0.51 to 10.32).

One study reported on stillbirth following HPV vaccination during pregnancy in Denmark (Faber 2019‐DNK). There was no association between HPV vaccination during pregnancy and stillbirth (OR 0.96, 95% CI 0.57 to 1.61).

One study reported on stillbirth in infants born to women who received HPV vaccination during pregnancy in Denmark (Scheller 2017‐DNK). There was no association between HPV vaccination and stillbirth (HR 2.43, 95% CI 0.45 to 13.21).

##### All‐cause mortality

See Table for effect estimates and Table for the risk of bias summary of included studies on all‐cause mortality. Neither study reported on causes of death.

**56:** Secondary clinical outcomes effect estimates: all‐cause mortality

**Study**	**Vaccine**	**Population (sex, age)**	**Sample size**	**Effect measure (time period)**	**Effect estimate**	**Adjustment factors**	**Notes**
Thomsen 2020‐DNK	Gardasil (Merck quadrivalent)	Female, 11 to 17 years	Vaccinated: 313,894 person‐years Unvaccinated: 313,885 person‐years	Incidence rate ratio (short‐term)	0.52 (0.27 to 0.97)	Age, calendar year of cohort entry, histories of hospital‐diagnosed asthma, diabetes, infections and mental disorders, number of general practitioner contacts within the past 5 years, previous psychometric tests or talk therapy with a general practitioner, a previous psychologist or psychiatrist visit in primary care, parental education, parental employment status, parental annual income, parental marital status and parental ethnicity	Cohort
Jemal 2013‐USA	Cervarix (GSK bivalent); Gardasil (Merck quadrivalent)	Female, NR	NR	Average annual percent change (short‐term; 2000 vs 2009)	‐1.9 (2000‐2009) and ‐0.9 (2005‐2009)	Unadjusted	Pre‐ vs post‐vaccine introduction

Vaccinated: vaccinated; Unvaccinated: control NR: not reported

**57:** Risk of bias summary: all‐cause mortality

**Study**	**Confounding**	**Selection**	**Classification of interventions**	**Deviations from intended interventions**	**Missing data**	**Measurement of outcomes**	**Selection of reported result**	**Overall risk of bias**
Thomsen 2020‐DNK	Serious	Low	Low	Low	Low	Low	Low	Serious
Jemal 2013‐USA	Critical	Moderate	Serious	Low	Low	Low	Low	Critical

Two studies were included that evaluated all‐cause mortality following HPV vaccination (Jemal 2013‐USA; Thomsen 2020‐DNK). One study was a cohort study (Thomsen 2020‐DNK) and the other (Jemal 2013‐USA) was a pre‐ versus post‐vaccine introduction study.

In the short term, there was a negative association between HPV vaccination and death (IRR 0.52, 95% CI 0.27 to 0.97) in the cohort study (Thomsen 2020‐DNK). The other study reported a decrease in the rate of all‐cause mortality from 2000 to 2009 (Jemal 2013‐USA).

##### Serious adverse events

No studies were identified that reported on population‐level rates of serious adverse events following HPV vaccination.

##### Incident HPV infection

See Table, Table and Table for effect estimates and Table for the risk of bias summary of included studies on incident HPV infection.

**58:** Secondary clinical outcomes effect estimates: incident HPV 16/18 infection

**Study**	**Vaccine**	**Population (sex, age at vaccination)**	**Sample size**	**Effect measure (time period)**	**Effect estimate**	**Adjustment factors**	**Notes**
Donken 2018‐NLD	Cervarix (GSK bivalent)	Female, 14 to 16 years	Vaccinated: 905 Unvaccinated: 763	Vaccine effectiveness (HPV 16/18; long‐term)	78.9% (69.2% to 85.6%)	Age, urbanisation degree, history of smoking, contraception use and sex	Cohort
Hoes 2021‐NLD	Cervarix (GSK bivalent)	Female, 12 to 13 years	Vaccinated: 1098 Unvaccinated: 929	Vaccine effectiveness (HPV 16/18; medium‐term)	84.0% (27.0% to 96.5%)	Age, ethnicity, ever had sexual intercourse and ever used contraception	Cohort
Sankaranarayanan 2018‐IND	Gardasil (Merck quadrivalent)	Female, 10 to 18 years	Vaccinated: 2019 Unvaccinated: 1479	Vaccine effectiveness (HPV 16/18; 3 doses; long‐term)	66.4% (53.6% to 76.3%)	Study site, birth cohort, religion, total number of pregnancies, age at first cervical cell sample collection, time between marriage and first cervical sample collection, delayed cervical sample collection, number of cervical cell sample collections	RCT extension
Sankaranarayanan 2018‐IND	Gardasil (Merck quadrivalent)	Female, 10 to 18 years	Vaccinated: 2166 Unvaccinated: 1479	Vaccine effectiveness (HPV 16/18; 2 doses; long‐term)	67.7% (55.2% to 77.2%)	Study site, birth cohort, religion, total number of pregnancies, age at first cervical cell sample collection, time between marriage and first cervical sample collection, delayed cervical sample collection, number of cervical cell sample collections	RCT extension
Sankaranarayanan 2018‐IND	Gardasil (Merck quadrivalent)	Female, 10 to 18 years	Vaccinated: 2858 Unvaccinated: 1479	Vaccine effectiveness (HPV 16/18; 1 dose; long‐term)	63.5% (51.2% to 73.1%)	Study site, birth cohort, religion, total number of pregnancies, age at first cervical cell sample collection, time between marriage and first cervical sample collection, delayed cervical sample collection, number of cervical cell sample collections	RCT extension
Kreimer 2011‐CRI	Cervarix (GSK bivalent)	Female, 18 to 25 years	Vaccinated: 1365 Unvaccinated: 1783	Vaccine efficacy (HPV 16/18; 1 dose; long‐term)	53.9% (‐57.1% to 92.4%)	Age‐ and location‐matched	RCT extension
Kreimer 2011‐CRI	Cervarix (GSK bivalent)	Female, 18 to 25 years	Vaccinated: 1365 Unvaccinated: 1783	Vaccine efficacy (HPV 16/18; 2 doses; long‐term)	58.4% (‐110.9% to 97.9%)	Age‐ and location‐matched	RCT extension
Kreimer 2011‐CRI	Cervarix (GSK bivalent)	Female, 18 to 25 years	Vaccinated: 1365 Unvaccinated: 1783	Vaccine efficacy (HPV 16/18; 3 doses; long‐term)	84.9% (69.8% to 93.2%)	Age‐ and location‐matched	RCT extension

HPV: human papillomavirus; RCT: randomised controlled trial

**59:** Secondary clinical outcomes effect estimates: incident HPV 6/11/16/18 infection

**Study**	**Vaccine**	**Population (sex, age at vaccination)**	**Sample size**	**Effect measure (time period)**	**Effect estimate**	**Adjustment factors**	**Notes**
Chambers 2022‐CAN	Gardasil (Merck quadrivalent)	Male, 16 to 30 years	Vaccinated: 109 Unvaccinated: 139	Prevalence ratio (HPV 6/11/16/18; medium‐term)	0.56 (0.24 to 1.31)	Age group, city, highest level of education, race/ethnicity, sexual orientation, laboratory‐confirmed HIV status, self‐reported lifetime history of STBBIs, lifetime smoking history, risk of alcohol‐related harm in the past 6 months, lifetime illicit drug use, lifetime poppers use, number of male anal sex partners in the past 6 months, sexual activity	Cohort
Ma 2017‐USA	Gardasil (Merck quadrivalent)	Female, 18 to 24 years*	Vaccinated: 58 Unvaccinated: 104	Odds ratio (HPV 6/11/16/18; short‐term)	0.36 (0.09 to 1.43)	Lifetime number of male sex partners, sexual behaviour in the past 6 months	Cohort; *age at outcome
Sankaranarayanan 2018‐IND	Gardasil (Merck quadrivalent)	Female, 10 to 18 years	Vaccinated: 2019 Unvaccinated: 1479	Vaccine effectiveness (HPV 6/11/16/18; 3 doses; long‐term)	54.7% (40.9% to 65.0%)	Study site, birth cohort, religion, total number of pregnancies, age at first cervical cell sample collection, time between marriage and first cervical sample collection, delayed cervical sample collection, number of cervical cell sample collections	RCT extension
Sankaranarayanan 2018‐IND	Gardasil (Merck quadrivalent)	Female, 10 to 18 years	Vaccinated: 2166 Unvaccinated: 1479	Vaccine effectiveness (HPV 6/11/16/18; 2 doses; long‐term)	59.0% (46.9% to 69.1%)	Study site, birth cohort, religion, total number of pregnancies, age at first cervical cell sample collection, time between marriage and first cervical sample collection, delayed cervical sample collection, number of cervical cell sample collections	RCT extension
Sankaranarayanan 2018‐IND	Gardasil (Merck quadrivalent)	Female, 10 to 18 years	Vaccinated: 2858 Unvaccinated: 1479	Vaccine effectiveness (HPV 6/11/16/18; 1 dose; long‐term)	54.1% (41.8% to 64.1%)	Study site, birth cohort, religion, total number of pregnancies, age at first cervical cell sample collection, time between marriage and first cervical sample collection, delayed cervical sample collection, number of cervical cell sample collections	RCT extension
Wissing 2019‐CAN	Gardasil (Merck quadrivalent)	Female, 18 to 26 years*	Vaccinated: 63 Unvaccinated: 434	Hazard ratio (HPV 6/11/16/18; at least 1 dose; medium‐term)	0.19 (0.07 to 0.55)	Age, race, smoking status, age at first coitus, number of lifetime sex partners, same‐sex partners and/or concurrent sex partners, condom use, average frequency of coitus with HITCH partner per week, duration of the sexual relationship	Cohort; *age at outcome
Wissing 2019‐CAN	Gardasil (Merck quadrivalent)	Female, 18 to 26 years*	Vaccinated: 63 Unvaccinated: 434	Hazard ratio (HPV 6/11/16/18; 1 dose; medium‐term)	0.21 (0.06 to 0.76)	Age, race, smoking status, age at first coitus, number of lifetime sex partners, same‐sex partners and/or concurrent sex partners, condom use, average frequency of coitus with HITCH partner per week, duration of the sexual relationship	Cohort; *age at outcome
Wissing 2019‐CAN	Gardasil (Merck quadrivalent)	Female, 18 to 26 years*	Vaccinated: 63 Unvaccinated: 434	Hazard ratio (HPV 6/11/16/18; at least 2 doses; medium‐term)	0.43 (0.23 to 0.81)	Age, race, smoking status, age at first coitus, number of lifetime sex partners, same‐sex partners and/or concurrent sex partners, condom use, average frequency of coitus with HITCH partner per week, duration of the sexual relationship	Cohort; *age at outcome

HPV: human papillomavirus; RCT: randomised controlled trial; STBBI: sexually transmitted and blood‐borne infections

**60:** Secondary clinical outcomes effect estimates: incident HPV 6/11/16/18/31/33/45/52/58 infection

**Study**	**Vaccine**	**Population (sex, age at vaccination)**	**Sample size**	**Effect measure (time period)**	**Effect estimate**	**Adjustment factors**	**Notes**
Chambers 2022‐CAN	Gardasil (Merck quadrivalent)	Male 16 to 30 years	Vaccinated: 109 Unvaccinated: 139	Prevalence ratio (HPV 6/11/16/18/31/33/45/52/58; medium‐term)	0.80 (0.43 to 1.49)	Age group, city, highest level of education, race/ethnicity, sexual orientation, laboratory‐confirmed HIV status, self‐reported lifetime history of STBBIs, lifetime smoking history, risk of alcohol‐related harm in the past 6 months, lifetime illicit drug use, lifetime poppers use, number of male anal sex partners in the past 6 months, sexual activity	Cohort
Donken 2018‐NLD	Cervarix (GSK bivalent)	Female, 14 to 16 years	Vaccinated: 905 Unvaccinated: 763	Vaccine effectiveness (HPV 6/11/16/18/31/33/45/52/58; long‐term)	32.3% (20.2% to 42.4%)	Age, urbanisation degree, history of smoking, contraception use and sex	Cohort

HPV: human papillomavirus; STBBI: sexually transmitted and blood‐borne infections

**61:** Risk of bias summary: incident HPV infection

**Study**	**Confounding**	**Selection**	**Classification of interventions**	**Deviations from intended interventions**	**Missing data**	**Measurement of outcomes**	**Selection of reported result**	**Overall risk of bias**
**Incident HPV 16/18 infection**								
Donken 2018‐NLD	Serious	Low	Low	Low	Moderate	Low	Low	Serious
Hoes 2021‐NLD	Serious	Low	Low	Low	Moderate	Low	Low	Serious
Sankaranarayanan 2018‐IND	Moderate	Low	Low	Low	Moderate	Low	Low	Moderate
Kreimer 2011‐CRI	Serious	Low	Low	Low	Moderate	Low	Low	Serious

**Incident HPV 6/11/16/18 infection**								
Chambers 2022‐CAN	Moderate	Low	Moderate	Low	Moderate	Low	Low	Moderate
Ma 2017‐USA	Serious	Low	Moderate	Low	Moderate	Low	Low	Serious
Sankaranarayanan 2018‐IND	Moderate	Low	Low	Low	Moderate	Low	Low	Moderate
Wissing 2019‐CAN	Serious	Low	Low	Low	Low	Low	Low	Serious

**Incident HPV 6/11/16/18/31/33/45/52/58 infection**								
Chambers 2022‐CAN	Moderate	Low	Moderate	Low	Moderate	Low	Low	Moderate
Donken 2018‐NLD	Serious	Low	Low	Low	Moderate	Low	Low	Serious

HPV: human papillomavirus

Seven studies were identified that reported on incident HPV infection following HPV vaccination (Chambers 2022‐CAN; Donken 2018‐NLD; Hoes 2021‐NLD; Kreimer 2011‐CRI; Ma 2017‐USA; Sankaranarayanan 2018‐IND; Wissing 2019‐CAN).

*HPV 16/18*

Two cohort studies (Donken 2018‐NLD; Hoes 2021‐NLD) and two RCT extension studies (Kreimer 2011‐CRI; Sankaranarayanan 2018‐IND) reported on incident HPV 16/18 infections following HPV vaccination.

Vaccine effectiveness against incident HPV 16/18 infection ranged from 77.5% to 84% in the cohort studies and 66.4% to 84.9% in the RCT extension studies.

Vaccine effectiveness for partial schedules (i.e. one or two doses) ranged in the RCT extension studies from 58.4% to 67.7% for two doses and 53.9% to 63.5% for one dose.

*HPV 6/11/16/18*

Three cohort studies (Chambers 2022‐CAN; Ma 2017‐USA; Wissing 2019‐CAN) and one RCT extension study (Sankaranarayanan 2018‐IND) reported on incident HPV 6/11/16/18 infections following HPV vaccination.

Two cohort studies reported a reduced odds of incident HPV 6/11/16/18 infection following HPV vaccination but with confidence intervals that included no difference (Chambers 2022‐CAN; Ma 2017‐USA). The other cohort study reported a reduced risk of incident HPV 6/11/16/18 infection following HPV vaccination with at least two doses (HR 0.43, 95% CI 0.23 to 0.81) and at least one dose (HR 0.19, 95% CI 0.07 to 0.55).

Vaccine effectiveness against incident HPV 6/11/16/18 infection ranged between 54.7% following three doses, 59% following two doses and 54.1% following one dose of HPV vaccine in the RCT extension study (Sankaranarayanan 2018‐IND).

*HPV 6/11/16/18/31/33/45/52/58*

Two cohort studies reported on incident HPV 6/11/16/18/31/33/45/52/58 infection following HPV vaccination (Chambers 2022‐CAN; Donken 2018‐NLD). Vaccine effectiveness was 33% (95% CI 19.1% to 44.6%) in one study (Donken 2018‐NLD) and the prevalence ratio was 0.80 (95% CI 0.43 to 1.49) in the other (Chambers 2022‐CAN).

##### Persistent HPV infection

See Table, Table and Table for effect estimates and Table for the risk of bias summary of included studies on persistent HPV infection.

**62:** Secondary clinical outcomes effect estimates: persistent HPV 16/18 infection

**Study**	**Vaccine**	**Population (sex, age at vaccination)**	**Sample size**	**Effect measure (time period)**	**Effect estimate**	**Adjustment factors**	**Notes**
Donken 2018‐NLD	Cervarix (GSK bivalent)	Female, 14 to 16 years	Vaccinated: 883 Unvaccinated: 752	Vaccine effectiveness (HPV 16/18; long‐term)	95.8% (86.6% to 98.7%)	Age, urbanisation degree, any history of smoking, any history of contraception use and any history of sex	Cohort
Ounchanum 2024‐THA/VNM	Cervarix (GSK bivalent)	Female, 12 to 24 years	Vaccinated: 47 Unvaccinated: 145	Prevalence ratio (HPV 16/18); long‐term – not receiving vaccination	1.37 (1.08 to 1.74)	HIV, education, ever been pregnant, age < 20, BMI ≥ 20 kg/m^2^, alcohol, tobacco, substance use, lifetime number of sex partners ≥ 6, number of sex partners, past 6 months, condom use with vaginal sex, past 6 months, history of STIs at baseline, laboratory diagnosis of STIs during the study	Cohort
Sankaranarayanan 2018‐IND	Gardasil (Merck quadrivalent)	Female, 10 to 18 years	Vaccinated: 1460 Unvaccinated: 1260	Vaccine effectiveness (HPV 16/18; 3 doses; long‐term)	93.3% (77.5% to 99.7%)	Study site, birth cohort, religion, total number of pregnancies, age at first cervical cell sample collection, time between dates of marriage and first cervical sample collection, delayed cervical sample collection, number of cervical cell sample collections per participant	RCT extension
Sankaranarayanan 2018‐IND	Gardasil (Merck quadrivalent)	Female, 10 to 18 years	Vaccinated: 1452 Unvaccinated: 1260	Vaccine effectiveness (HPV 16/18; 2 doses; long‐term)	93.1% (77.3% to 99.8%)	Study site, birth cohort, religion, total number of pregnancies, age at first cervical cell sample collection, time between dates of marriage and first cervical sample collection, delayed cervical sample collection, number of cervical cell sample collections per participant	RCT extension
Sankaranarayanan 2018‐IND	Gardasil (Merck quadrivalent)	Female, 10 to 18 years	Vaccinated: 2135 Unvaccinated: 1260	Vaccine effectiveness (HPV 16/18; 1 dose; long‐term)	95.4% (85.0% to 99.9%)	Study site, birth cohort, religion, total number of pregnancies, age at first cervical cell sample collection, time between dates of marriage and first cervical sample collection, delayed cervical sample collection, number of cervical cell sample collections per participant	RCT extension

BMI: body mass index; HPV: human papillomavirus; RCT: randomised controlled trial; STI: sexually transmitted infection

**63:** Secondary clinical outcomes effect estimates: persistent HPV 6/11/16/18 infection

**Study**	**Vaccine**	**Population (sex, age at vaccination)**	**Sample size**	**Effect measure (time period)**	**Effect estimate**	**Adjustment factors**	**Notes**
Chambers 2022‐CAN	Gardasil (Merck quadrivalent)	Male, 16 to 30 years	Vaccinated: 109 Unvaccinated: 139	Prevalence ratio (HPV 6/11/16/18; medium‐term)	0.53 (0.25 to 1.14)	Age group, city, highest level of education, race/ethnicity, sexual orientation, laboratory‐confirmed HIV status, self‐reported lifetime history of STBBIs, lifetime smoking history, risk of alcohol‐related harm in the past 6 months, lifetime illicit drug use, lifetime poppers use, number of male anal sex partners in the past 6 months, sexual activity	Cohort
Wissing 2019‐CAN	Gardasil (Merck quadrivalent)	Female, 18 to 26 years*	Vaccinated: 63 Unvaccinated: 434	Odds ratio (HPV 6/11/16/18; at least 1 dose; medium‐term)	0.13 (0.03 to 0.63)	Age, race, smoking status, age at first coitus, number of lifetime sex partners (coitus), whether the individual had same‐sex partners and/or concurrent sex partners, condom use, average frequency of coitus with HITCH partner per week, and duration of the sexual relationship	Cohort; *age at outcome
Sankaranarayanan 2018‐IND	Gardasil (Merck quadrivalent)	Female, 10 to 18 years	Vaccinated: 1460 Unvaccinated: 1260	Vaccine effectiveness (HPV 6/11/16/18; 3 doses; long‐term)	90.3% (71.9% to 98.5%)	Study site, birth cohort, religion, total number of pregnancies, age at first cervical cell sample collection, time between dates of marriage and first cervical sample collection, delayed cervical sample collection, number of cervical cell sample collections per participant	RCT extension
Sankaranarayanan 2018‐IND	Gardasil (Merck quadrivalent)	Female, 10 to 18 years	Vaccinated: 1452 Unvaccinated: 1260	Vaccine effectiveness (HPV 6/11/16/18; 2 doses; long‐term)	93.7% (79.8% to 99.8%)	Study site, birth cohort, religion, total number of pregnancies, age at first cervical cell sample collection, time between dates of marriage and first cervical sample collection, delayed cervical sample collection, number of cervical cell sample collections per participant	RCT extension
Sankaranarayanan 2018‐IND	Gardasil (Merck quadrivalent)	Female, 10 to 18 years	Vaccinated: 2135 Unvaccinated: 1260	Vaccine effectiveness (HPV 6/11/16/18; 1 dose; long‐term)	93.4% (81.1% to 99.1%)	Study site, birth cohort, religion, total number of pregnancies, age at first cervical cell sample collection, time between dates of marriage and first cervical sample collection, delayed cervical sample collection, number of cervical cell sample collections per participant	RCT extension

HPV: human papillomavirus; RCT: randomised controlled trial; STBBI: sexually transmitted and blood‐borne infections

**64:** Secondary clinical outcomes effect estimates: persistent HPV 6/11/16/18/31/33/45/52/58 infection

**Study**	**Vaccine**	**Population (sex, age at vaccination)**	**Sample size**	**Effect measure (time period)**	**Effect estimate**	**Adjustment factors**	**Notes**
Chambers 2022‐CAN	Gardasil (Merck quadrivalent)	Male, 16 to 30 years	Vaccinated: 109 Unvaccinated: 139	Prevalence ratio (HPV 6/11/16/18/31/33/45/52/58; medium‐term)	0.65 (0.33 to 1.27)	Age group, city, highest level of education, race/ethnicity, sexual orientation, laboratory‐confirmed HIV status, self‐reported lifetime history of STBBIs, lifetime smoking history, risk of alcohol‐related harm in the past 6 months, lifetime illicit drug use, lifetime poppers use, number of male anal sex partners in the past 6 months, sexual activity	Cohort
Donken 2018‐NLD	Cervarix (GSK bivalent)	Female, 14 to 16 years	Vaccinated: 883 Unvaccinated: 752	Vaccine effectiveness (HPV 6/11/16/18/31/33/45/52/58; long‐term)	51.7% (35.9% to 63.7%)	Age, urbanisation degree, any history of smoking, any history of contraception use, and any history of sex	Cohort

HPV: human papillomavirus; STBBI: sexually transmitted and blood‐borne infections

**65:** Risk of bias summary: persistent HPV infection

**Study**	**Confounding**	**Selection**	**Classification of interventions**	**Deviations from intended interventions**	**Missing data**	**Measurement of outcomes**	**Selection of reported result**	**Overall risk of bias**
**Persistent HPV 16/18 infection**								
Donken 2018‐NLD	Serious	Low	Low	Low	Moderate	Low	Low	Serious
Ounchanum 2024‐THA/VNM	Serious	Moderate	Low	Low	Moderate	Low	Low	Serious
Sankaranarayanan 2018‐IND	Moderate	Low	Low	Low	Moderate	Low	Low	Moderate

**Persistent HPV 6/11/16/18 infection**								
Chambers 2022‐CAN	Moderate	Low	Moderate	Low	Moderate	Low	Low	Moderate
Sankaranarayanan 2018‐IND	Moderate	Low	Low	Low	Moderate	Low	Low	Moderate
Wissing 2019‐CAN	Serious	Low	Low	Low	Low	Low	Low	Serious

**Persistent HPV 6/11/16/18/31/33/45/52/58 infection**								
Chambers 2022‐CAN	Moderate	Low	Moderate	Low	Moderate	Low	Low	Moderate
Donken 2018‐NLD	Serious	Low	Low	Low	Moderate	Low	Low	Serious

HPV: human papillomavirus

Five studies were identified that reported on persistent HPV infection following HPV vaccination (Chambers 2022‐CAN; Donken 2018‐NLD; Ounchanum 2024‐THA/VNM; Sankaranarayanan 2018‐IND; Wissing 2019‐CAN).

*HPV 16/18*

Two cohort studies (Donken 2018‐NLD; Ounchanum 2024‐THA/VNM) and one RCT extension study (Sankaranarayanan 2018‐IND) reported on persistent HPV 16/18 infection. In one cohort study, vaccine effectiveness was 97.7% (95% CI 83.5% to 99.7%) (Donken 2018‐NLD), and in the other the prevalence ratio was 1.37 (95% CI 1.08 to 1.74) (Ounchanum 2024‐THA/VNM). Vaccine effectiveness was 93.3% (95% CI 77.5% to 99.7%) in the RCT extension study (Sankaranarayanan 2018‐IND).

The effectiveness of two doses (93.1%, 95% CI 77.3% to 99.8%) and one dose (95.4%, 95% CI 85.0% to 99.9%) were also reported by the RCT extension study (Sankaranarayanan 2018‐IND).

*HPV 6/11/16/18*

Two cohort studies (Chambers 2022‐CAN; Wissing 2019‐CAN) and one RCT extension study (Sankaranarayanan 2018‐IND) reported on persistent HPV 6/11/16/18 infection. One cohort study reported an odds ratio of 0.13 (95% CI 0.03 to 0.63) for persistent infection (Wissing 2019‐CAN) and the other a prevalence ratio of 0.53 (95% CI 0.25 to 1.14) following HPV vaccine (Chambers 2022‐CAN). Vaccine effectiveness was 90.3% (71.9% to 98.5%) in the RCT extension.

The effectiveness of two doses (93.7%, 95% CI 79.8% to 99.8%) and one dose (93.4%, 95% CI 81.1% to 99.1%) were also reported by the RCT extension study (Sankaranarayanan 2018‐IND).

*HPV 6/11/16/18/31/33/45/52/58*

Two cohort studies reported on persistent HPV 6/11/16/18/31/33/45/52/58 infection (Chambers 2022‐CAN; Donken 2018‐NLD). Vaccine effectiveness was reported at 50.4% (95% CI 29.7% to 65.1%) in one study (Donken 2018‐NLD) and a prevalence ratio of 0.65 (95% CI 0.33 to 1.27) in the other (Chambers 2022‐CAN).

##### Prevalent HPV infection

See Table, Table, Table and Table for effect estimates and Table for the risk of bias summary of included studies on incident HPV infection.

**66:** Secondary clinical outcomes effect estimates: prevalent HPV 16/18 infection

**Study**	**Vaccine**	**Population (sex, age at vaccination)**	**Sample size**	**Effect measure (time period)**	**Effect estimate**	**Adjustment factors**	**Notes**
Batmunkh 2020‐MNG	Gardasil (Merck quadrivalent)	Female, 16 to 26 years*	Vaccinated: 87 Unvaccinated: 266	Prevalence ratio (HPV 16/18; 1 dose; long‐term)	0.08 (0.01 to 0.56)	Employment status and income	Cross‐sectional; *age at outcome
Batmunkh 2019‐MNG	Gardasil (Merck quadrivalent)	Female, 16 to 26 years*	Vaccinated: 726 Unvaccinated: 790	Risk ratio (HPV 16/18; 3 doses; long‐term)	0.31 (0.22 to 0.45)	Unadjusted	Cross‐sectional; *age at outcome
Bobadilla 2024‐PAR	Gardasil (Merck quadrivalent);	Female, 18 to 25 years	Vaccinated: 104 Unvaccinated: 150	Prevalence ratio (HPV 16/18)	0.35 (0.10 to 1.20)	Unadjusted	Cross‐sectional
Bogaards 2019‐NLD	Cervarix (GSK bivalent)	Female, 16 to 24 years*	Vaccinated: 1305 Unvaccinated: 799	Odds ratio (HPV 16/18; ≥ 1 dose; long‐term)	0.09 (0.06 to 0.14)	Age, migration background, education level, number of sex partners last 6 months, lifetime number of sex partners, age at sexual debut, history of STI, hormonal contraceptives use, STI‐related symptoms and age vaccination was offered	Cross‐sectional; *age at outcome
Carnalla 2021‐MEX	Cervarix (GSK bivalent); Gardasil (Merck quadrivalent)	Female, 9 to 10 years	Vaccinated: 93 Unvaccinated: 88	Prevalence ratio (HPV 16/18; long‐term)	0.16 (0.02 to 1.28)	Unadjusted	Cross‐sectional
Carozzi 2018‐ITA	Gardasil (Merck quadrivalent)	Female, 18 to 30 years*	Vaccinated: 771 Unvaccinated: 537	Odds ratio (HPV 16/18; long‐term)	0.11 (0.04 to 0.30)	Marital status, smoking status, number of sexual partners in the past 6 months, number of lifetime sexual partners and sexually transmitted diseases	Cross‐sectional; *age at outcome
Combita 2021‐COL	Gardasil (Merck quadrivalent)	Female, 18 to 25 years*	Vaccinated: 1986 Unvaccinated: 1287	Vaccine efficacy (HPV 16/18; long‐term)	61.5 (54.3 to 67.6)	Age, socioeconomic stratum, residence area, marital status, smoking, age of sexual debut, number of sexual partners, occasional sexual partners, contraceptive method and history of sexually transmitted diseases	Cross‐sectional; *age at outcome
Cummings 2012‐USA	Gardasil (Merck quadrivalent)	Female, 14 to 17 years*	Vaccinated: 75 Unvaccinated: 150	Odds ratio (HPV 16/18)	3.6 (1.2 to 10.6)	Matched with two historical controls from a previous cross‐sectional study by age at enrolment, clinic site and reported sexual activity at the time of enrolment	Cross‐sectional; *age at outcome
Delere 2014‐DEU	Cervarix (GSK bivalent); Gardasil (Merck quadrivalent)	Female, 20 to 25 years*	Vaccinated: 223 Unvaccinated: 512	Prevalence ratio (HPV 16/18)	0.62 (0.43 to 0.89)	Unadjusted	Cross‐sectional; *age at outcome
Enerly 2019‐NOR	Gardasil (Merck quadrivalent)	Female, 18 to 20 years*	Vaccinated: 239 Unvaccinated: 73	Prevalence ratio (HPV 16/18; ≥ 1 dose; long‐term)	0.11 (0.01 to 1.30)	Lifetime number of sexual partners, age at sexual debut and time since last sexual intercourse	Cross‐sectional; *age at outcome
Feder 2019‐USA	Gardasil (Merck quadrivalent)	Female, 21 to 29 years*	Vaccinated: 221 Unvaccinated: 143	Risk ratio (HPV 16/18; ≥ 1 dose; long‐term)	0.50 (0.19 to 1.32)	Unadjusted	Cross‐sectional; *age at outcome
Gonzalez 2020‐ARG	Gardasil (Merck quadrivalent)	Female, 15 to 17 years*	Vaccinated: 1224 Unvaccinated: 957	Odds ratio (HPV 16/18; medium‐term)	0.07 (0.04 to 0.12)	Unadjusted	Cross‐sectional; *age at outcome
Heard 2017‐FRA	Gardasil (Merck quadrivalent)	Female, 18 to 25 years*	Vaccinated: 822 Unvaccinated: 1893	Prevalence ratio (HPV 16/18; ≥ 1 dose)	0.01 (0.00 to 0.07)	Unadjusted	Cross‐sectional; *age at outcome
Hiramatsu 2021‐JPN	Gardasil (Merck quadrivalent)	Female, 20 to 21 years*	Vaccinated: 877 Unvaccinated: 170	Odds ratio (HPV 16/18)	0.06 (0.00 to 0.92)	Unadjusted	Cross‐sectional; *age at outcome
Hirth 2017‐USA	Gardasil (Merck quadrivalent)	Female, 18 to 30 years*	Vaccinated: 668 Unvaccinated: 2372	Prevalence ratio (oral HPV 16/18)	0.31 (0.07 to 1.31)	Unadjusted	Cross‐sectional; *age at outcome
Jeannot 2018‐CHE	Gardasil (Merck quadrivalent)	Female, 18 to 23 years*	Vaccinated: 284 Unvaccinated: 125	Prevalence ratio (HPV 16/18)	0.15 (0.04 to 0.53)	Unadjusted	Cross‐sectional; *age at outcome
Kahn 2016‐USA	Gardasil (Merck quadrivalent)	Female, 13 to 26 years*	Vaccinated: 286 Unvaccinated: 485	Odds ratio (HPV 16/18; 2006‐7 vs 2013‐4)	0.19 (0.12 to 0.31)	Propensity score analysis adjusted for sociodemographic characteristics, gynaecologic history, sexual history and enrolment site.	Repeated cross‐sectional; *age at outcome
Kitamura 2023‐JPN	Cervarix (GSK bivalent); Gardasil (Merck quadrivalent); Gardasil 9 (Merck nonavalent)	Female, 16 to 75 years	Vaccinated: 454 Unvaccinated: 1579	Odds ratio (HPV 16/18)	0.05 (0.01 to 0.20)	Age, educational status, smoking status, number of lifetime sexual partners, age at coitarche, marital status, divorce, number of children, commercial sex work experience, current STI, history of STI	Cross‐sectional
Kreimer 2011‐CRI	Cervarix (GSK bivalent)	Female, 18 to 25 years	Vaccinated: 112 Unvaccinated: 1783	Vaccine efficacy (HPV 16/18; 1 dose)	82.1 (40.2 to 97.0)	Age‐ and location‐matched	RCT extension
Kreimer 2011‐CRI	Cervarix (GSK bivalent)	Female, 18 to 25 years	Vaccinated: 62 Unvaccinated: 1783	Vaccine efficacy (HPV 16/18; 2 doses)	83.8 (19.5 to 99.2)	Age‐ and location‐matched	RCT extension
Kreimer 2011‐CRI	Cervarix (GSK bivalent)	Female, 18 to 25 years	Vaccinated: 1365 Unvaccinated: 1783	Vaccine efficacy (HPV 16/18; 3 doses)	80.2 (70.7 to 87.0)	Age‐ and location‐matched	RCT extension
Kudo 2019‐JPN	Gardasil (Merck quadrivalent)	Female, 20 to 22 years*	Vaccinated: 3167 Unvaccinated: 1386	Odds ratio (HPV 16/18)	0.11 (0.05 to 0.27)	Year of birth and lifetime number of sex partners	Cross‐sectional; *age at outcome
Kudo 2019‐JPN	Gardasil (Merck quadrivalent)	Female, 25 to 26 years*	Vaccinated: 150 Unvaccinated: 279	Odds ratio (HPV 16/18; long‐term)	0.06 (0.00 to 1.05)	Unadjusted	Cross‐sectional; *age at outcome
Kumakech 2016‐UGA	Cervarix (GSK bivalent)	Female, 15 to 24 years*	Vaccinated: 252 Unvaccinated: 236	Odds ratio (HPV 16/18)	0.08 (0.01 to 0.64)	Age, age at sexual debut and educational level	Cross‐sectional; *age at outcome
Laake 2020‐NOR	Gardasil (Merck quadrivalent)	Female, 17 years*	Vaccinated: 6360 Unvaccinated: 5468	Relative risk (HPV 16/18)	0.22 (0.17 to 0.29)	Unadjusted	Cross‐sectional; *age at outcome
Latsuzbaia 2019‐LUX	Gardasil (Merck quadrivalent)	Female, 18 to 29 years*	Vaccinated: 216 Unvaccinated: 232	Odds ratio (HPV 16/18)	0.10 (0.01 to 0.82)	Number of lifetime sexual partners, last partnership duration and age	Cross‐sectional; *age at outcome
Latsuzbaia 2019‐LUX	Cervarix (GSK bivalent)	Female, 18 to 29 years*	Vaccinated: 216 Unvaccinated: 131	Odds ratio (HPV 16/18)	0.19 (0.02 to 1.62)	Number of lifetime sexual partners, last partnership duration and age	Cross‐sectional; *age at outcome
Lee 2022‐THA	Cervarix (GSK bivalent); Gardasil (Merck quadrivalent)	Female, 20 to 45 years	Vaccinated: 493 Unvaccinated: 500	Vaccine effectiveness (HPV 16/18)	84.6% (43.5 to 95.8)	Baseline Pap test results and baseline hrHPV test	Cross‐sectional
Lehtinen 2017a‐FIN	Cervarix (GSK bivalent)	Male, 12 to 15 years	Vaccinated: 395 Unvaccinated: 149	Relative risk (HPV 16/18)	0.05 (0.00 to 1.04)	Unadjusted	Cross‐sectional
Loenenbach 2023‐DEU	Cervarix (GSK bivalent); Gardasil (Merck quadrivalent); Gardasil 9 (Merck nonavalent)	Female 20 to 25 years	Vaccinated: 348 Unvaccinated: 377	Prevalence ratio (HPV 16/18)	0.5 (0.3 to 1.0)	Age, nationality, education, smoking, number of sexual partners, immunodeficiency and cancer screening	Cross‐sectional
Lynge 2020‐DNK	Gardasil (Merck quadrivalent)	Female, 14 years	Vaccinated: 5685 Unvaccinated: 518	Relative risk (HPV 16/18)	0.05 (0.03 to 0.09)	Unadjusted	Cross‐sectional
Markowitz 2019‐USA	Gardasil (Merck quadrivalent)	Female, 20 to 24 years	Vaccinated: 2059 Unvaccinated: 2057	Prevalence ratio (HPV 16/18; 2007 vs 2015‐2016)	0.24 (0.18 to 0.32)	Unadjusted	Repeated cross‐sectional
Markowitz 2019‐USA	Gardasil (Merck quadrivalent)	Female, 25 to 29 years	Vaccinated: 2420 Unvaccinated: 2081	Prevalence ratio (HPV 16/18; 2007 vs 2015‐2016)	0.64 (0.50 to 0.81)	Unadjusted	Repeated cross‐sectional
Mehanna 2019‐GBR	Cervarix (GSK bivalent)	Female, 12 to 13 years	Vaccinated: 123 Unvaccinated: 16	Prevalence ratio (oral HPV 16/18)	0.26 (0.03 to 2.71)	Unadjusted	Cross‐sectional
Mehanna 2019‐GBR	Cervarix (GSK bivalent)	Female, 14 to 17 years	Vaccinated: 59 Unvaccinated: 25	Prevalence ratio (oral HPV 16/18)	0.14 (0.01 to 3.43)	Unadjusted	Cross‐sectional
Mesher 2018‐GBR	Cervarix (GSK bivalent)	Female, 12 to 15 years	Vaccinated: 1176 Unvaccinated: 117	Vaccine effectiveness (HPV 16/18)	82.0% (60.6 to 91.8)	Age, testing venue type and chlamydia positivity	Repeated cross‐sectional
Mesher 2018‐GBR	Cervarix (GSK bivalent)	Female, 16 to 18 years	Vaccinated: 614 Unvaccinated: 289	Vaccine effectiveness (HPV 16/18)	48.7% (20.8 to 66.8)	Age, testing venue type and chlamydia positivity	Repeated cross‐sectional
Napolitano 2024‐ITA	Not reported	Female and male, 18 to 30 years	Vaccinated: 490 Unvaccinated: 512	Prevalence ratio (HPV 16/18)	1.04 (0.07 to 16.66)	Unadjusted	Cross‐sectional
Nilyanimit 2024‐THA	Cervarix (GSK bivalent)	Female, 16 to 18 years	Vaccinated: 211 Unvaccinated: 376	Prevalence ratio (HPV 16/18)	0.07 (0.00 to 1.14)	No cases in exposed group; age, sexual experience, sexual debut age in years, condom usage	Cross‐sectional
Palmer 2019‐GBR	Cervarix (GSK bivalent)	Female, 20 to 21 years*	Vaccinated: 3962 Unvaccinated: 4008	Odds ratio (HPV 16/18; 3 doses)	0.40 (0.33 to 0.48)	Birth year, SIMD score and age at vaccination	Cross‐sectional; *age at outcome
Palmer 2019‐GBR	Cervarix (GSK bivalent)	Female, 20 to 21 years*	Vaccinated: 391 Unvaccinated: 4008	Odds ratio (HPV 16/18; 2 doses)	0.75 (0.57 to 0.99)	Birth year, SIMD score and age at vaccination	Cross‐sectional; *age at outcome
Palmer 2019‐GBR	Cervarix (GSK bivalent)	Female, 20 to 21 years*	Vaccinated: 223 Unvaccinated: 4008	Odds ratio (HPV 16/18; 1 dose)	0.89 (0.63 to 1.25)	Birth year, SIMD score and age at vaccination	Cross‐sectional; *age at outcome
Purrinos‐Hermida 2018‐ESP	Cervarix (GSK bivalent)	Female, 18 to 26 years*	Vaccinated: 353 Unvaccinated: 392	Prevalence ratio (HPV 16/18)	0.06 (0.01 to 0.28)	Age group, first intercourse > 16 years old, 3 or more partners along life and 2 or more partners in the last year	Cross‐sectional; *age at outcome
Reyburn 2023‐FJI	Gardasil (Merck quadrivalent)	Female, 15 to 23 years	Vaccinated: 189 Unvaccinated: 376	Prevalence ratio (3 doses; HPV 16/18)	0.11 (0.04 to 0.36)	Age, ethnicity and smoking	Cross‐sectional
Reyburn 2023‐FJI	Gardasil (Merck quadrivalent)	Female, 15 to 23 years	Vaccinated: 158 Unvaccinated: 376	Prevalence ratio (1 dose; HPV 16/18)	0.19 (0.07 to 0.52)	Age, ethnicity and smoking	Cross‐sectional
Saeki 2024‐JPN	Cervarix (GSK bivalent); Gardasil (Merck quadrivalent); Gardasil 9 (Merck nonavalent)	Female, 16 to 39 years	Vaccinated: 299 Unvaccinated: 1230	Odds ratio (HPV 16/18)	0.03 (0.00 to 0.19)	Unadjusted	Cross‐sectional
Saldanha 2020‐PRT	Gardasil (Merck quadrivalent)	Female, < 25 years*	Vaccinated: 951 Unvaccinated: 902	Prevalence ratio (HPV 16/18)	0.27 (0.13 to 0.56)	Unadjusted	Cross‐sectional; *age at outcome
Sankaranarayanan 2018‐IND	Gardasil (Merck quadrivalent)	Female, 10 to 18 years	Vaccinated: 818 Unvaccinated: 179	Odds ratio (oral HPV 16/18)	0.4 (0.2 to 1.0)	Age at oral sample collection	Cross‐sectional
Sarr 2019‐CAN	Gardasil (Merck quadrivalent)	Female, > 18 years*	Vaccinated: 79 Unvaccinated: 956	Vaccine effectiveness (HPV 16/18)	86.1 (15.0 to 99.7)	Age and number of new sexual partners in the last 12 months	Cross‐sectional; *age at outcome
Tanton 2017‐GBR	Cervarix (GSK bivalent)	Female, 18 to 20 years*	Vaccinated: 84 Unvaccinated: 265	Odds ratio (HPV 16/18)	0.46 (0.20 to 1.05)	Age, number of lifetime partners	Repeated cross‐sectional; *age at outcome
Tanton 2017‐GBR	Cervarix (GSK bivalent)	Female, 18 to 20 years*	Vaccinated: 84 Unvaccinated: 265	Prevalence ratio (HPV 16/18; 1999‐2001 vs 2010‐2012)	0.48 (0.24 to 0.93)	Age	Repeated cross‐sectional; *age at outcome
Van Eer 2021‐NLD	Cervarix (GSK bivalent)	Female, 16 to 24 years*	Vaccinated: 352 Unvaccinated: 190	Prevalence ratio (HPV 16/18)	0.40 (0.23 to 0.70)	Unadjusted	Cross‐sectional; *age at outcome
Van Eer 2021‐NLD	Cervarix (GSK bivalent)	Female, 16 to 24 years*	Vaccinated: 352 Unvaccinated: 190	Prevalence ratio (concurrent genital‐anal HPV 16/18)	0.05 (0.01 to 0.34)	Unadjusted	Cross‐sectional; *age at outcome
Wendland 2021‐BRA	Gardasil (Merck quadrivalent)	Female, 16 to 25 years*	Vaccinated: 677 Unvaccinated: 5268	Risk ratio (HPV 16/18)	0.40 (0.28 to 0.56)	Unadjusted	Cross‐sectional; *age at outcome
Woestenberg 2020‐NLD	Cervarix (GSK bivalent)	Female, 16 to 24 years*	Vaccinated: 357 Unvaccinated: 191	Vaccine effectiveness (anal HPV 16/18)	89.9 (63.0 to 97.2)	Age, education level, history of anal sex, number of sex partners in the past 6 months, sexually transmitted infection‐related symptoms, and use of hormonal contraceptives	Cross‐sectional; *age at outcome
Wright 2019‐USA	Gardasil (Merck quadrivalent)	Female, 21 to 34 years*	Vaccinated: 2977 Unvaccinated: 11,176	Odds ratio (HPV 16/18)	0.3 (0.2 to 0.4)	Age	Cross‐sectional; *age at outcome
Huyghe 2023‐BEL	Cervarix (GSK bivalent); Gardasil (Merck quadrivalent)	Female 20 to 23 years	N = 3008	Relative risk (HPV 16/18; 2010 vs 2019)	0.19 (0.14 to 0.27)	Unadjusted	Pre‐ vs post‐vaccine introduction
Khoo 2022‐MYS	Cervarix (GSK bivalent); Gardasil (Merck quadrivalent)	Female, 18 to 24 years	Vaccinated: 75 Unvaccinated: 1135	Prevalence change (HPV 16/18)	‐91% (‐99% to ‐14.5%)	Unadjusted	Pre‐ vs post‐vaccine introduction
Khoo 2022‐MYS	Cervarix (GSK bivalent); Gardasil (Merck quadrivalent)	Female, 35 to 45 years	Vaccinated: 75 Unvaccinated: 1135	Prevalence change (HPV 16/18)	‐38.2% (‐77.8% to 72.3%)	Unadjusted	Pre‐ vs post‐vaccine introduction
Rebolj 2022‐GBR	Cervarix (GSK bivalent)	Female 24 to 25 years	N = 64274	Vaccine effectiveness (HPV 16/18)	90 (89 to 92)	Deprivation and laboratory	Pre‐ vs post‐vaccine introduction
Saeki 2024‐JPN	Cervarix (GSK bivalent); Gardasil (Merck quadrivalent); Gardasil 9 (Merck nonavalent)	Female, 16 to 39 years	Vaccinated: 382 Unvaccinated: 3984	Odds ratio (HPV 16/18; 2011 vs 2021)	0.56 (0.41 to 0.76)	Unadjusted	Pre‐ vs post‐vaccine introduction

HPV: human papillomavirus; hrHPV: high‐risk human papillomavirus; RCT: randomised controlled trial; SIMD: Scottish Index of Multiple Deprivation; STI: sexually transmitted infection

**67:** Secondary clinical outcomes effect estimates: prevalent HPV 6/11/16/18 infection

**Study**	**Vaccine**	**Population (sex, age at vaccination)**	**Sample size**	**Effect measure (time period)**	**Effect estimate**	**Adjustment factors**	**Notes**
Ahrlund‐Richter 2019‐SWE	Gardasil (Merck quadrivalent)	Female, 15 to 23 years*	Vaccinated: 138 Unvaccinated: 30	Risk ratio (HPV 6/11/16/18)	0.26 (0.10 to 0.65)	Unadjusted	Cross‐sectional; *age at outcome
Abel 2021‐USA	Gardasil (Merck quadrivalent)	Female and male, 18 to 36 years*	Vaccinated: 198 Unvaccinated: 4801	Prevalence ratio (HPV 6/11/16/18; 1 dose)	0.41 (0.06 to 2.95)	Unadjusted	Cross‐sectional; *age at outcome
Abel 2021‐USA	Gardasil (Merck quadrivalent)	Female and male, 18 to 36 years*	Vaccinated: 799 Unvaccinated: 4801	Prevalence ratio (HPV 6/11/16/18; 2 or 3 doses)	0.20 (0.05 to 0.83)	Unadjusted	Cross‐sectional; *age at outcome
Balgovind 2024‐AUS	Gardasil (Merck quadrivalent)	Male MSM, 18 to 34 years	Vaccinated: 152 Unvaccinated: 479	Prevalence ratio (HPV 6/11/16/18)	0.8 (0.6 to 1.06)	Unadjusted	Cross‐sectional
Balgovind 2024‐AUS	Gardasil (Merck quadrivalent)	Male, 18 to 34 years	Vaccinated: 103 Unvaccinated: 891	Prevalence ratio (HPV 6/11/16/18)	1.01 (0.56 to 1.83)	Unadjusted	Cross‐sectional
Baussano 2021‐RWA/BTN	Gardasil (Merck quadrivalent)	Female, 17 to 22 years*	Vaccinated: 962 Unvaccinated: 519	Prevalence ratio (HPV 6/11/16/18; Rwanda)	0.05 (0.01 to 0.17)	Age group, place of birth and reported history of sexual intercourse	Cross‐sectional; *age at outcome
Baussano 2021‐RWA/BTN	Gardasil (Merck quadrivalent)	Female, 17 to 22 years*	Vaccinated: 864 Unvaccinated: 77	Prevalence ratio (HPV 6/11/16/18; Bhutan)	0.05 (0.01 to 0.51)	Reported history of sexual intercourse	Cross‐sectional; *age at outcome
Baussano 2020‐BTN	Gardasil (Merck quadrivalent)	Female, 17 to 29 years*	Vaccinated: 1053 Unvaccinated: 1338	Prevalence ratio (HPV 6/11/16/18)	0.12 (0.08 to 0.20)	Age group, type of invitation to participate in the survey, age at first sexual intercourse, lifetime number of sexual partners and partner's “extramarital” sexual behaviour	Cross‐sectional; *age at outcome
Berenson 2021‐USA	Gardasil (Merck quadrivalent)	Female and male, 18 to 59 years*	Vaccinated: 939 Unvaccinated: 8498	Risk ratio (oral HPV 6/11/16/18)	0.44 (0.19 to 0.99)	Unadjusted	Cross‐sectional; *age at outcome
Berenson 2021‐USA	Gardasil (Merck quadrivalent)	Female, 18 to 59 years*	Vaccinated: 723 Unvaccinated: 4164	Risk ratio (oral HPV 6/11/16/18)	0.25 (0.03 to 1.86)	Unadjusted	Cross‐sectional; *age at outcome
Berenson 2021‐USA	Gardasil (Merck quadrivalent)	Male, 18 to 59 years*	Vaccinated: 216 Unvaccinated: 4334	Risk ratio (oral HPV 6/11/16/18)	0.10 (0.04 to 0.25)	Unadjusted	Cross‐sectional; *age at outcome
Bobadilla 2024‐PAR	Gardasil (Merck quadrivalent)	Female, 18 to 25 years	Vaccinated: 104 Unvaccinated: 150	Prevalence ratio (HPV 16/18)	0.27 (0.10 to 0.75)	Unadjusted	Cross‐sectional
Carozzi 2018‐ITA	Gardasil (Merck quadrivalent)	Female, 18 to 30 years*	Vaccinated: 771 Unvaccinated: 537	Odds ratio (HPV 6/11/16/18)	0.10 (0.04 to 0.27)	Marital status, smoking status, number of sexual partners in the past 6 months, number of lifetime sexual partners and sexually transmitted diseases	Cross‐sectional; *age at outcome
Chambers 2022‐CAN	Gardasil (Merck quadrivalent); Gardasil 9 (Merck nonavalent)	Male, ≤ 23 years	Vaccinated: 118 Unvaccinated: 349	Prevalence ratio (anal HPV 6/11/16/18)	0.64 (0.42 to 0.99)	Age group, city, education, lifetime smoking history, lifetime history of STIs (excluding HIV and anogenital warts) and number of condomless receptive anal sex encounters in the past 6 months	Cross‐sectional
Chambers 2022‐CAN	Gardasil (Merck quadrivalent); Gardasil 9 (Merck nonavalent)	Male, > 23 years	Vaccinated: 112 Unvaccinated: 349	Prevalence ratio (anal HPV 6/11/16/18)	0.82 (0.55 to 1.20)	Age group, city, education, lifetime smoking history, lifetime history of STIs (excluding HIV and anogenital warts) and number of condomless receptive anal sex encounters in the past 6 months	Cross‐sectional
Chambers 2022‐CAN	Gardasil (Merck quadrivalent); Gardasil 9 (Merck nonavalent)	Male, 16 to 30 years*	Vaccinated: 136 Unvaccinated: 349	Prevalence ratio (anal HPV 6/11/16/18; 3 doses)	0.75 (0.52 to 1.10)	Age group, city, education, lifetime smoking history, lifetime history of STIs (excluding HIV and anogenital warts) and number of condomless receptive anal sex encounters in the past 6 months	Cross‐sectional; *age at outcome
Chambers 2022‐CAN	Gardasil (Merck quadrivalent); Gardasil 9 (Merck nonavalent)	Male, 16 to 30 years*	Vaccinated: 184 Unvaccinated: 349	Prevalence ratio (HPV 6/11/16/18; at least 2 doses)	0.77 (0.55 to 1.07)	Age group, city, education, lifetime smoking history, lifetime history of STIs (excluding HIV and anogenital warts) and number of condomless receptive anal sex encounters in the past 6 months	Cross‐sectional; *age at outcome
Chambers 2022‐CAN	Gardasil (Merck quadrivalent); Gardasil 9 (Merck nonavalent)	Male 16 to 30 years*	Vaccinated: 241 Unvaccinated: 349	Prevalence ratio (HPV 6/11/16/18; at least 1 dose)	0.73 (0.54 to 1.00)	Age group, city, education, lifetime smoking history, lifetime history of STIs (excluding HIV and anogenital warts) and number of condomless receptive anal sex encounters in the past 6 months	Cross‐sectional; *age at outcome
Chow 2017‐AUS	Gardasil (Merck quadrivalent)	Male, ≤ 25 years*	Vaccinated: 1217 Unvaccinated: 250	Prevalence ratio (HPV 6/11/16/18; 2004‐7 vs 2007‐15)	0.50 (0.37 to 0.70)	Unadjusted	Repeated cross‐sectional; *age at outcome
Chow 2019‐AUS	Gardasil (Merck quadrivalent)	Male, 17 to 19 years*	Vaccinated: 146 Unvaccinated: 152	Prevalence ratio (Penile HPV 6/11/16/18; 2014‐5 vs 2016‐7)	0.28 (0.03 to 2.62)	Age and source of recruitment	Repeated cross‐sectional; *age at outcome
Chow 2021a‐AUS	Gardasil (Merck quadrivalent)	Male, 16 to 20 years*	Vaccinated: 193 Unvaccinated: 193	Prevalence ratio (Anal HPV 6/11/16/18)	0.24 (0.14 to 0.42)	Age, circumcision and sex with women	Repeated cross‐sectional; *age at outcome
Chow 2021a‐AUS	Gardasil (Merck quadrivalent)	Male, 16 to 20 years*	Vaccinated: 179 Unvaccinated: 177	Prevalence ratio (Penile HPV 6/11/16/18)	0.48 (0.24 to 0.97)	Age, circumcision and sex with women	Repeated cross‐sectional; *age at outcome
Chow 2021a‐AUS	Gardasil (Merck quadrivalent)	Male, 16 to 20 years*	Vaccinated: 199 Unvaccinated: 200	Prevalence ratio (Oral HPV 6/11/16/18)	0.10 (0.01 to 0.97)	Age, circumcision and sex with women	Repeated cross‐sectional; *age at outcome
Closson 2020‐USA	Gardasil (Merck quadrivalent)	Female, 18 to 35 years*	Vaccinated: 325 Unvaccinated: 725	Odds ratio (HPV 6/11/16/18)	0.39 (0.19 to 0.83)	US birth, US citizenship, marital status, ethnicity, age, year of survey, education, health insurance, condom use, number of sexual partners, age at first sex, smoking history, binge‐drinking	Cross‐sectional; *age at outcome
Combita 2021‐COL	Gardasil (Merck quadrivalent)	Female, 18 to 25 years*	Vaccinated: 1986 Unvaccinated: 1287	Vaccine efficacy (HPV 6/11/16/18)	62.6 (56.1 to 68.2)	Age, socioeconomic stratum, residence area, marital status, smoking, age of sexual debut, number of sexual partners, occasional sexual partners, contraceptive method and history of sexually transmitted diseases	Cross‐sectional; *age at outcome
Cummings 2012‐USA	Gardasil (Merck quadrivalent)	Female, 14 to 17 years*	Vaccinated: 75 Unvaccinated: 150	Odds ratio (HPV 6/11/16/18)	5.6 (1.9 to 16.5)	Matched with two historical controls from a previous cross‐sectional study by age at enrolment, clinic site and reported sexual activity at the time of enrolment	Cross‐sectional; *age at outcome
DeSisto 2024‐USA	Gardasil (Merck quadrivalent); Gardasil 9 (Merck nonavalent)	MSM, 18 to 45 years	Vaccinated: 1249 Unvaccinated: 1553	Prevalence ratio (anal HPV 6/11/16/18)	0.8 (0.68 to 0.95)	Adjusted for city, race/ethnicity and non‐9vHPV type prevalent infection	Cross‐sectional
De Souza 2023‐AUS	Gardasil (Merck quadrivalent); Gardasil 9 (Merck nonavalent)	Female and male, 18 to 70 years	Vaccinated: 230 Unvaccinated: 671	Relative risk (oral; HPV 6/11/16/18)	0.2 (0.03 to 1.49)	Unadjusted	Cross‐sectional
Dillner 2018‐EU	Gardasil (Merck quadrivalent)	Female, 18 to 50 years*	Vaccinated: 6299 Unvaccinated: 6494	Prevalence ratio (HPV 6/11/16/18; 2006‐8 vs 2012‐3)	0.86 (0.79 to 0.95)	Unadjusted	Repeated cross‐sectional; *age at outcome
Enerly 2019‐NOR	Gardasil (Merck quadrivalent)	Female, 18 to 20 years*	Vaccinated: 239 Unvaccinated: 73	Prevalence ratio (HPV 6/11/16/18; ≥1 dose)	0.04 (0.00 to 0.42)	Lifetime number of sexual partners, age at sexual debut and time since last sexual intercourse	Cross‐sectional; *age at outcome
Garland 2018‐AUS	Gardasil (Merck quadrivalent)	Female, 18 to 25 years*	Vaccinated: 620 Unvaccinated: 117	Prevalence ratio (HPV 6/11/16/18)	0.22 (0.08 to 0.64)	Unadjusted	Cross‐sectional; *age at outcome
Goggin 2018‐CAN	Gardasil (Merck quadrivalent)	Female, 17 to 19 years*	Vaccinated: 577 Unvaccinated: 114	Prevalence ratio (long‐term; HPV 6/11/16/18)	0.05 (0.01 to 0.24)	Unadjusted	Cross‐sectional; *age at outcome
Goggin 2018‐CAN	Gardasil (Merck quadrivalent)	Female, 20 to 22 years*	Vaccinated: 372 Unvaccinated: 194	Prevalence ratio (long‐term; HPV 6/11/16/18)	0.15 (0.06 to 0.39)	Unadjusted	Cross‐sectional; *age at outcome
Goggin 2018‐CAN	Gardasil (Merck quadrivalent)	Female, 23 to 29 years*	Vaccinated: 87 Unvaccinated: 371	Prevalence ratio (long‐term; HPV 6/11/16/18)	0.88 (0.45 to 1.75)	Unadjusted	Cross‐sectional; *age at outcome
Gonzalez 2020‐ARG	Gardasil (Merck quadrivalent)	Female, 15 to 17 years*	Vaccinated: 1224 Unvaccinated: 957	Odds ratio (HPV 6/11/16/18)	0.24 (0.18 to 0.31)	Unadjusted	Cross‐sectional; *age at outcome
Heard 2017‐FRA	Gardasil (Merck quadrivalent)	Female, 18 to 25 years*	Vaccinated: 822 Unvaccinated: 1893	Prevalence ratio (HPV 6/11/16/18; ≥1 dose)	0.04 (0.02 to 0.10)	Unadjusted	Cross‐sectional; *age at outcome
Hirth 2017‐USA	Gardasil (Merck quadrivalent)	Female, 18 to 30 years*	Vaccinated: 668 Unvaccinated: 2372	Prevalence ratio (oral HPV 6/11/16/18)	0.22 (0.05 to 0.92)	Unadjusted	Cross‐sectional; *age at outcome
Jacot‐Guillarmod 2017‐CHE	Gardasil (Merck quadrivalent)	Female, 18 years*	Vaccinated: 245 Unvaccinated: 77	Prevalence ratio (HPV 6/11/16/18)	0.63 (0.16 to 2.45)	Unadjusted	Cross‐sectional; *age at outcome
Kahn 2016‐USA	Gardasil (Merck quadrivalent)	Female, 13 to 26 years*	Vaccinated: 286 Unvaccinated: 485	Odds ratio (HPV 6/11/16/18; 2006‐7 vs 2013‐4)	0.18 (0.12 to 0.27)	Propensity score analysis adjusted for sociodemographic characteristics, gynaecologic history, sexual history and enrolment site	Repeated cross‐sectional; *age at outcome
Laake 2020‐NOR	Gardasil (Merck quadrivalent)	Female, 17 years*	Vaccinated: 6360 Unvaccinated: 5468	Relative risk (HPV 6/11/16/18)	0.19 (0.15 to 0.24)	Unadjusted	Cross‐sectional; *age at outcome
Loenenbach 2023‐DEU	Cervarix (GSK bivalent); Gardasil (Merck quadrivalent); Gardasil 9 (Merck nonavalent)	Female 20 to 25 years	Vaccinated: 348 Unvaccinated: 377	Prevalence ratio (HPV 6/11/16/18)	0.5 (0.3 to 0.9)	Age, nationality, education, smoking, number of sexual partners, immunodeficiency and cancer screening	Cross‐sectional
Machalek 2018‐AUS	Gardasil (Merck quadrivalent)	Female, 18 to 35 years	Vaccinated: 381 Unvaccinated: 275	Prevalence ratio (HPV 6/11/16/18; 2005‐7 vs 2015)	0.08 (0.03 to 0.20)	Age and smoking status	Repeated cross‐sectional; *age at outcome
Markowitz 2020‐USA	Gardasil (Merck quadrivalent)	Female, ≤ 18 years	Vaccinated: 2349 Unvaccinated: 1052	Prevalence ratio (HPV 6/11/16/18; 3 doses)	0.06 (0.04 to 0.12)	Race/ethnicity and age at screening	Cross‐sectional
Markowitz 2020‐USA	Gardasil (Merck quadrivalent)	Female, ≤ 18 years	Vaccinated: 229 Unvaccinated: 1052	Prevalence ratio (HPV 6/11/16/18; 2 doses)	0.05 (0.01 to 0.39)	Race/ethnicity and age at screening	Cross‐sectional
Markowitz 2020‐USA	Gardasil (Merck quadrivalent)	Female, ≤ 18 years	Vaccinated: 207 Unvaccinated: 1052	Prevalence ratio (HPV 6/11/16/18; 1 dose)	0.06 (0.01 to 0.42)	Race/ethnicity and age at screening	Cross‐sectional
Markowitz 2020‐USA	Gardasil (Merck quadrivalent)	Female, > 18 years	Vaccinated: 261 Unvaccinated: 1052	Prevalence ratio (HPV 6/11/16/18; 3 doses)	0.77 (0.44 to 1.36)	Race/ethnicity and age at screening	Cross‐sectional
Markowitz 2020‐USA	Gardasil (Merck quadrivalent)	Female, > 18 years	Vaccinated: 75 Unvaccinated: 1052	Prevalence ratio (HPV 6/11/16/18; 2 doses)	0.36 (0.09 to 1.44)	Race/ethnicity and age at screening	Cross‐sectional
Markowitz 2020‐USA	Gardasil (Merck quadrivalent)	Female, > 18 years	Vaccinated: 96 Unvaccinated: 1052	Prevalence ratio (HPV 6/11/16/18; 1 dose)	0.57 (0.21 to 1.53)	Race/ethnicity and age at screening	Cross‐sectional
Markowitz 2019‐USA	Gardasil (Merck quadrivalent)	Female, < 19 years	Vaccinated: 706 Unvaccinated: 4138	Prevalence ratio (HPV 6/11/16/18)	0.1 (0.1 to 0.3)	Age, race, poverty, any chlamydia, HIV or pregnancy test	Repeated cross‐sectional
Markowitz 2019‐USA	Gardasil (Merck quadrivalent)	Female, ≥ 19 years	Vaccinated: 625 Unvaccinated: 4138	Prevalence ratio (HPV 6/11/16/18)	0.7 (0.5 to 1.2)	Age, race, poverty, any chlamydia, HIV or pregnancy test	Repeated cross‐sectional
Markowitz 2019‐USA	Gardasil (Merck quadrivalent)	Female, 20 to 24 years	Vaccinated: 2059 Unvaccinated: 2057	Prevalence ratio (HPV 6/11/16/18; 2007 vs 2015‐2016)	0.22 (0.17 to 0.29)	Unadjusted	Repeated cross‐sectional
Markowitz 2019‐USA	Gardasil (Merck quadrivalent)	Female, 25 to 29 years	Vaccinated: 2420 Unvaccinated: 2081	Prevalence ratio (HPV 6/11/16/18; 2007 vs 2015‐2016)	0.62 (0.50 to 0.78)	Unadjusted	Repeated cross‐sectional
McDaniel 2020‐USA	Gardasil (Merck quadrivalent)	Female and male, 30 to 33 years*	Vaccinated: 46 Unvaccinated: 776	Prevalence ratio (oral HPV 6/11/16/18)	0.62 (0.09 to 4.50)	Unadjusted	Cross‐sectional; *age at outcome
McGregor 2018‐AUS	Gardasil (Merck quadrivalent)	Female, 18 to 26 years*	Vaccinated: 142 Unvaccinated: 155	Prevalence ratio (HPV 6/11/16/18; 2005‐7 vs 2014‐5)	0.06 (0.01 to 0.24)	Unadjusted	Repeated cross‐sectional; indigenous subgroup of population; *age at outcome
Napolitano 2024‐ITA	Not reported	Female and male, 18 to 30 years	Vaccinated: 490 Unvaccinated: 512	Prevalence ratio (HPV 6/11/16/18)	0.70 (0.12 to 4.16)	Unadjusted	Cross‐sectional
Rosenblum 2021‐USA	Gardasil (Merck quadrivalent)	Female, 14 to 17 years*	Vaccinated: 233 Unvaccinated: 244	Relative risk (cervicogenital HPV 6/11/16/18)	0.2 (0.1 to 0.8)	Unadjusted	Cross‐sectional; *age at outcome
Rosenblum 2021‐USA	Gardasil (Merck quadrivalent)	Female, 18 to 24 years*	Vaccinated: 241 Unvaccinated: 448	Relative risk (cervicogenital HPV 6/11/16/18)	0.2 (0.1 to 0.3)	Unadjusted	Cross‐sectional; *age at outcome
Rosenblum 2021‐USA	Gardasil (Merck quadrivalent)	Male, 18 to 24 years*	Vaccinated: 52 Unvaccinated: 252	Relative risk (HPV 6/11/16/18)	0.7 (0.1 to 5.4)	Unadjusted	Cross‐sectional; *age at outcome
Rosenblum 2021‐USA	Gardasil (Merck quadrivalent)	Female, 18 to 24 years*	Vaccinated: 430 Unvaccinated: 679	Relative risk (oral HPV 6/11/16/18)	0.1 (0.0 to 1.3)	Unadjusted	Cross‐sectional; *age at outcome
Rosenblum 2021‐USA	Gardasil (Merck quadrivalent)	Female, 18 to 26 years*	Vaccinated: 106 Unvaccinated: 1004	Difference in predicted probability (HPV 6/11/16/18; 1 dose)	‐5.0 (‐5.6 to ‐4.5)	age, race/ethnicity, age at sexual debut, and lifetime number of male sexual partners.	Cross‐sectional; *age at outcome
Rosenblum 2021‐USA	Gardasil (Merck quadrivalent)	Female, 18 to 26 years*	Vaccinated: 126 Unvaccinated: 1004	Difference in predicted probability (HPV 6/11/16/18; 2 doses)	‐1.7 (‐2.4 to ‐0.1)	Age, race/ethnicity, age at sexual debut and lifetime number of male sexual partners.	Cross‐sectional; *age at outcome
Rosenblum 2021‐USA	Gardasil (Merck quadrivalent)	Female, 18 to 26 years*	Vaccinated: 384 Unvaccinated: 1004	Difference in predicted probability (HPV 6/11/16/18; 3 doses)	‐4.3 (‐4.6 to ‐4.0)	Age, race/ethnicity, age at sexual debut and lifetime number of male sexual partners	Cross‐sectional; *age at outcome
Rosenblum 2021‐USA	Gardasil (Merck quadrivalent)	Female, 14 to 19 years*	Vaccinated: 666 Unvaccinated: 1363	Prevalence ratio (HPV 6/11/16/18; 2003‐6 vs 2015‐18)	0.12 (0.06 to 0.26)	Race/ethnicity and ever having had sex	Repeated cross‐sectional; *age at outcome
Rosenblum 2021‐USA	Gardasil (Merck quadrivalent)	Female, 20 to 24 years*	Vaccinated: 368 Unvaccinated: 432	Prevalence ratio (HPV 6/11/16/18; 2003‐6 vs 2015‐18)	0.19 (0.09 to 0.40	Race/ethnicity and ever having had sex	Repeated cross‐sectional; *age at outcome
Rosenblum 2021‐USA	Gardasil (Merck quadrivalent)	Female, 25 to 29 years*	Vaccinated: 430 Unvaccinated: 403	Prevalence ratio (HPV 6/11/16/18; 2003‐6 vs 2015‐18)	0.85 (0.50 to 1.46)	Race/ethnicity and ever having had sex	Repeated cross‐sectional; *age at outcome
Rosenblum 2021‐USA	Gardasil (Merck quadrivalent)	Female, 30 to 34 years*	Vaccinated: 413 Unvaccinated: 389	Prevalence ratio (HPV 6/11/16/18; 2003‐6 vs 2015‐18)	0.67 (0.37 to 1.21)	Race/ethnicity and ever having had sex	Repeated cross‐sectional; *age at outcome
Sankaranarayanan 2018‐IND	Gardasil (Merck quadrivalent)	Female, 10 to 18 years	Vaccinated: 818 Unvaccinated: 179	Odds ratio (oral HPV 6/11/16/18)	0.6 (0.3 to 1.1)	Age at oral sample collection	Cross‐sectional
Sarr 2019‐CAN	Gardasil (Merck quadrivalent)	Female, > 18 years*	Vaccinated: 79 Unvaccinated: 956	Vaccine effectiveness (HPV 6/11/16/18)	61.9% (‐23.5 to 92.6)	Age and number of new sexual partners in the last 12 months	Cross‐sectional; *age at outcome
Sayinzoga 2023‐RWA	Gardasil (Merck quadrivalent)	Female, 17 to 29 years	Vaccinated: 655 Unvaccinated: 2349	Vaccine effectiveness (HPV 6/11/16/18)	70% (52 to 82)	Age, level of education, HIV status and lifetime number of sexual partners	Cross‐sectional
Schlecht 2016‐USA	Gardasil (Merck quadrivalent)	Female, 12 to 19 years*	Vaccinated: 957 Unvaccinated: 182	Incidence rate ratio (HPV 6/11/16/18)	0.22 (0.13 to 0.37)	Exposure time, all concurrent types, current age, race/ethnicity, lifetime number of sex partners, history of anal sex, recent number of vaginal sex partners, age at first intercourse and sexual experience at time of vaccination	Repeated cross‐sectional; *age at outcome
Schlecht 2016‐USA	Gardasil (Merck quadrivalent)	Female, 12 to 19 years	Vaccinated: 957 Unvaccinated: 182	Incidence rate ratio (anal HPV 6/11/16/18)	0.33 (0.18 to 0.69)	Exposure time, all concurrent types, current age, race/ethnicity, lifetime number of sex partners, history of anal sex, recent number of vaginal sex partners, age at first intercourse and sexual experience at time of vaccination	Repeated cross‐sectional; *age at outcome
Schlecht 2019‐USA	Gardasil (Merck quadrivalent)	Female, 13 to 21 years*	Vaccinated: 1067 Unvaccinated: 192	Odds ratio (oral HPV 6/11/16/18)	0.20 (0.04 to 0.998)	Age, years since first sexual activity, concurrent cervical detection of quadrivalent HPV vaccine types	Repeated cross‐sectional; *age at outcome
Shilling 2021‐AUS	Gardasil (Merck quadrivalent)	Female, 18 to 35 years*	Vaccinated: 964 Unvaccinated: 348	Odds ratio (HPV 6/11/16/18)	0.13 (0.05 to 0.32)	Age	Cross‐sectional; *age at outcome
Soderlund‐Strand 2014‐SWE	Gardasil (Merck quadrivalent)	Female, all ages*	Vaccinated: 532 Unvaccinated: 10,840	Prevalence ratio (HPV 6/11/16/18; 2008 vs 2013)	0.49 (0.34 to 0.70)	Unadjusted	Repeated cross‐sectional; *age at outcome
Soderlund‐Strand 2014‐SWE	Gardasil (Merck quadrivalent)	Male, all ages*	Vaccinated: 1255 Unvaccinated: 11,009	Prevalence ratio (HPV 6/11/16/18; 2008 vs 2013)	0.47 (0.32 to 0.68)	Unadjusted	Repeated cross‐sectional; *age at outcome
Spinner 2019‐USA	Gardasil (Merck quadrivalent)	Female, 13 to 26 years*	Vaccinated: 865 Unvaccinated: 715	Odds ratio (HPV 6/11/16/18)	0.13 (0.08 to 0.22)	Enrolment site, age, race, history of STI, age at first intercourse, number of sexual partners, main partner being male, ever had anal sex, condom use and smoking history	Repeated cross‐sectional; *age at outcome
Subasinghe 2020‐AUS	Gardasil (Merck quadrivalent)	Female, 16 to 25 years*	Vaccinated: 218 Unvaccinated: 8	Risk ratio (HPV 6/11/16/18)	0.02 (0.00 to 0.18)	Unadjusted	Cross‐sectional; *age at outcome
Tabrizi 2014‐AUS	Gardasil (Merck quadrivalent)	Female, 18 to 24 years*	Vaccinated: 909 Unvaccinated: 351	Vaccine effectiveness (HPV 6/11/16/18)	86% (71% to 93%)	Age, hormonal contraceptive use, education, country of birth and number of sexual partners in the past 12 months	Repeated cross‐sectional; *age at outcome
Tabrizi 2014‐AUS	Gardasil (Merck quadrivalent)	Female, 18 to 24 years*	Vaccinated: 909 Unvaccinated: 351	Prevalence ratio (HPV 6/11/16/18; 2005‐2007 vs 2010‐2012)	0.22 (0.16 to 0.31)	Age, hormonal contraceptive use	Repeated cross‐sectional; *age at outcome
Wendland 2021‐BRA	Gardasil (Merck quadrivalent)	Female, 16 to 25 years*	Vaccinated: 677 Unvaccinated: 5268	Risk ratio (HPV 6/11/16/18)	0.43 (0.33 to 0.58)	Unadjusted	Cross‐sectional; *age at outcome
Widdice 2019‐USA	Gardasil (Merck quadrivalent)	Male, 13 to 26 years*	Vaccinated: 143 Unvaccinated: 471	Risk ratio (HPV 6/11/16/18; 3 doses)	0.85 (0.60 to 1.20)	Unadjusted	Cross‐sectional; *age at outcome
Widdice 2019‐USA	Gardasil (Merck quadrivalent)	Male, 13 to 26 years*	Vaccinated: 37 Unvaccinated: 471	Risk ratio (HPV 6/11/16/18; 2 doses)	1.06 (0.61 to 1.84)	Unadjusted	Cross‐sectional; *age at outcome
Widdice 2019‐USA	Gardasil (Merck quadrivalent)	Male, 13 to 26 years*	Vaccinated: 58 Unvaccinated: 471	Risk ratio (HPV 6/11/16/18; 1 dose)	0.74 (0.43 to 1.30)	Unadjusted	Cross‐sectional; *age at outcome
Winer 2021‐USA	Gardasil (Merck quadrivalent)	Male, 11 to 18 years	Vaccinated: 348 Unvaccinated: 339	Prevalence ratio (penile HPV 6/11/16/18)	0.15 (0.04 to 0.62)	Age, history of ever taking PrEP for HIV prevention, HIV status, lifetime number of sex partners	Cross‐sectional
Winer 2021‐USA	Gardasil (Merck quadrivalent)	Male, 19 to 26 years	Vaccinated: 348 Unvaccinated: 339	Prevalence ratio (penile HPV 6/11/16/18)	0.80 (0.52 to 1.22)	Age, history of ever taking PrEP for HIV prevention, HIV status, lifetime number of sex partners	Cross‐sectional
Winer 2021‐USA	Gardasil (Merck quadrivalent)	Male, 11 to 18 years	Vaccinated: 348 Unvaccinated: 339	Prevalence ratio (anal and/or oral HPV 6/11/16/18)	0.41 (0.24 to 0.57)	Age, history of ever taking PrEP for HIV prevention, HIV status, lifetime number of sex partners	Cross‐sectional
Winer 2021‐USA	Gardasil (Merck quadrivalent)	Male, 19 to 26 years	Vaccinated: 348 Unvaccinated: 339	Prevalence ratio (anal and/or oral HPV 6/11/16/18)	0.82 (0.67 to 0.98)	Age, history of ever taking PrEP for HIV prevention, HIV status, lifetime number of sex partners	Cross‐sectional
Wissing 2019‐CAN	Gardasil (Merck quadrivalent)	Female, 18 to 26 years*	Vaccinated: 63 Unvaccinated: 434	Odds ratio (HPV 6/11/16/18)	0.14 (0.04 to 0.51)	Age, race, smoking status, age at first coitus, number of lifetime sex partners, same‐sex partners and/or concurrent sex partners, condom use, average frequency of coitus and duration of the sexual relationship	Cross‐sectional; *age at outcome
Khoo 2022‐MYS	Cervarix (GSK bivalent); Gardasil (Merck quadrivalent)	Female, 18 to 24 years	Vaccinated: 75 Unvaccinated: 1135	Prevalence change (HPV 6/11/16/18)	‐86.5% (‐97.5% to ‐27.5%)	Unadjusted	Pre‐ vs post‐vaccine introduction
Khoo 2022‐MYS	Cervarix (GSK bivalent); Gardasil (Merck quadrivalent)	Female, 35 to 45 years	Vaccinated: 75 Unvaccinated: 1135	Prevalence change (HPV 6/11/16/18)	‐40.5% (‐74.3% to 37.9%)	Unadjusted	Pre‐ vs post‐vaccine introduction

HPV: human papillomavirus; MSM: men who have sex with men; PrEP: pre‐exposure prophylaxis; STI: sexually transmitted infection

**68:** Secondary clinical outcomes effect estimates: prevalent HPV 31/33/45/52/58 infection

**Study**	**Vaccine**	**Population (sex, age at vaccination)**	**Sample size**	**Effect measure (time period)**	**Effect estimate**	**Adjustment factors**	**Notes**
Abel 2021‐USA	Gardasil (Merck quadrivalent)	Female and male, 18 to 36 years*	Vaccinated: 198 Unvaccinated: 4801	Prevalence ratio (HPV 31/33/45/52/58; 1 dose)	0.90 (0.12 to 6.58)	Unadjusted	Cross‐sectional; *age at outcome
Abel 2021‐USA	Gardasil (Merck quadrivalent)	Female and male, 18 to 36 years*	Vaccinated: 799 Unvaccinated: 4801	Prevalence ratio (HPV 31/33/45/52/58; 2 or 3 doses)	1.34 (0.55 to 3.22)	Unadjusted	Cross‐sectional; *age at outcome
DeSisto 2024‐USA	Gardasil (Merck quadrivalent); Gardasil 9 (Merck nonavalent)	MSM, 18 to 45 years	Vaccinated: 1249 Unvaccinated: 1553	Prevalence ratio (anal HPV 31/33/45/52/58)	0.73 (0.62 to 0.85)	Adjusted for city, race/ethnicity and non‐9vHPV type prevalent infection.	Cross‐sectional
Mesher 2018‐GBR	Cervarix (GSK bivalent)	Female, 12 to 15 years	Vaccinated: 1176 Unvaccinated: 117	Vaccine effectiveness (HPV 31/33/45/52/58)	16.4% (‐30.9 to 46.5)	Age, testing venue type and chlamydia positivity	Repeated cross‐sectional
Mesher 2018‐GBR	Cervarix (GSK bivalent)	Female, 16 to 18 years	Vaccinated: 614 Unvaccinated: 289	Vaccine effectiveness (HPV 31/33/45/52/58)	20.6% (−3.5 to 39.1)	Age, testing venue type and chlamydia positivity	Repeated cross‐sectional
Rosenblum 2021‐USA	Gardasil (Merck quadrivalent)	Female, 14 to 19 years*	Vaccinated: 666 Unvaccinated: 1363	Prevalence ratio (HPV 31/33/45/52/58; 2003‐6 vs 2015‐18)	0.35 (0.18 to 0.65	Race/ethnicity and ever having had sex	Repeated cross‐sectional; *age at outcome
Rosenblum 2021‐USA	Gardasil (Merck quadrivalent)	Female, 20 to 24 years*	Vaccinated: 368 Unvaccinated: 432	Prevalence ratio (HPV 31/33/45/52/58; 2003‐6 vs 2015‐18)	0.62 (0.38 to 1.01	Race/ethnicity and ever having had sex	Repeated cross‐sectional; *age at outcome
Rosenblum 2021‐USA	Gardasil (Merck quadrivalent)	Female, 25 to 29 years*	Vaccinated: 430 Unvaccinated: 403	Prevalence ratio (HPV 31/33/45/52/58; 2003‐6 vs 2015‐18)	0.99 (0.58 to 1.67	Race/ethnicity and ever having had sex	Repeated cross‐sectional; *age at outcome
Rosenblum 2021‐USA	Gardasil (Merck quadrivalent)	Female, 30 to 34 years*	Vaccinated: 413 Unvaccinated: 389	Prevalence ratio (HPV 31/33/45/52/58; 2003‐6 vs 2015‐18)	0.68 (0.37 to 1.27	Race/ethnicity and ever having had sex	Repeated cross‐sectional; *age at outcome
Spinner 2019‐USA	Gardasil (Merck quadrivalent)	Female, 13 to 26 years*	Vaccinated: 865 Unvaccinated: 715	Odds ratio (HPV 31/33/45/52/58)	0.26 (0.16 to 0.42)	Enrolment site, age, race, history of STI, age at first intercourse, number of sexual partners, main partner being male, ever had anal sex, condom use and smoking history	Repeated cross‐sectional; *age at outcome
Tanton 2017‐GBR	Cervarix (GSK bivalent)	Female, 18 to 20 years*	Vaccinated: 84 Unvaccinated: 265	Prevalence ratio (HPV 31/33/45/52/58; 1999‐2001 vs 2010‐2012)	1.19 (0.69 to 2.05)	Age	Repeated cross‐sectional; *age at outcome
Khoo 2022‐MYS	Cervarix (GSK bivalent); Gardasil (Merck quadrivalent)	Female, 18 to 24 years	Vaccinated: 75 Unvaccinated: 1135	Prevalence change (HPV 31/33/45/52/58)	21.8% (‐73.1% to 45.2%)	Unadjusted	Pre‐ vs post‐vaccine introduction
Khoo 2022‐MYS	Cervarix (GSK bivalent); Gardasil (Merck quadrivalent)	Female, 35 to 45 years	Vaccinated: 75 Unvaccinated: 1135	Prevalence change % (HPV 31/33/45/52/58)	‐38.2% (‐69.9% to 26.9%)	Unadjusted	Pre‐ vs post‐vaccine introduction

HPV: human papillomavirus; MSM: men who have sex with men; STI: sexually transmitted infection

**69:** Secondary clinical outcomes effect estimates: prevalent HPV 6/11/16/18/31/33/45/52/58 infection

**Study**	**Vaccine**	**Population (sex, age at vaccination)**	**Sample size**	**Effect measure (time period)**	**Effect estimate**	**Adjustment factors**	**Notes**
Berenson 2021‐USA	Gardasil (Merck quadrivalent)	Female and male, 18 to 59 years*	Vaccinated: 939 Unvaccinated: 8498	Risk ratio (oral HPV 6/11/16/18/31/33/45/52/58)	0.60 (0.34 to 1.08)	Unadjusted	Cross‐sectional; *age at outcome
Berenson 2021‐USA	Gardasil (Merck quadrivalent)	Female, 18 to 59 years*	Vaccinated: 723 Unvaccinated: 4164	Risk ratio (oral HPV 6/11/16/18/31/33/45/52/58)	0.90 (0.35 to 2.30)	Unadjusted	Cross‐sectional; *age at outcome
Berenson 2021‐USA	Gardasil (Merck quadrivalent)	Male, 18 to 59 years*	Vaccinated: 216 Unvaccinated: 4334	Risk ratio (oral HPV 6/11/16/18/31/33/45/52/58)	0.94 (0.45 to 1.98)	Unadjusted	Cross‐sectional; *age at outcome
Chambers 2022‐CAN	Gardasil (Merck quadrivalent); Gardasil 9 (Merck nonavalent)	Male ≤ 23 years at vaccination	Vaccinated: 118 Unvaccinated: 349	Prevalence ratio (HPV 6/11/16/18/31/33/45/52/58)	0.76 (0.56 to 1.02)	Age group, city, education, lifetime smoking history, lifetime history of STIs (excluding HIV and anogenital warts) and number of condomless receptive anal sex encounters in the past 6 months	Cross‐sectional
Chambers 2022‐CAN	Gardasil (Merck quadrivalent); Gardasil 9 (Merck nonavalent)	Male > 23 years at vaccination	Vaccinated: 112 Unvaccinated: 349	Prevalence ratio (HPV 6/11/16/18/31/33/45/52/58)	0.69 (0.49 to 0.96)	Age group, city, education, lifetime smoking history, lifetime history of STIs (excluding HIV and anogenital warts) and number of condomless receptive anal sex encounters in the past 6 months	Cross‐sectional
Chambers 2022‐CAN	Gardasil (Merck quadrivalent); Gardasil 9 (Merck nonavalent)	Male, 16 to 30 years*	Vaccinated: 136 Unvaccinated: 349	Prevalence ratio (HPV 6/11/16/18/31/33/45/52/58; 3 doses)	0.70 (0.52 to 0.94)	Age group, city, education, lifetime smoking history, lifetime history of STIs (excluding HIV and anogenital warts) and number of condomless receptive anal sex encounters in the past 6 months	Cross‐sectional; *age at outcome
Chambers 2022‐CAN	Gardasil (Merck quadrivalent); Gardasil 9 (Merck nonavalent)	Male, 16 to 30 years*	Vaccinated: 184 Unvaccinated: 349	Prevalence ratio (HPV 6/11/16/18/31/33/45/52/58; 2 doses)	0.76 (0.59 0.98)	Age group, city, education, lifetime smoking history, lifetime history of STIs (excluding HIV and anogenital warts) and number of condomless receptive anal sex encounters in the past 6 months	Cross‐sectional; *age at outcome
Chambers 2022‐CAN	Gardasil (Merck quadrivalent); Gardasil 9 (Merck nonavalent)	Male 16 to 30 years*	Vaccinated: 241 Unvaccinated: 349	Prevalence ratio (HPV 6/11/16/18/31/33/45/52/58; at least 1 dose)	0.72 (0.57 to 0.91)	Age group, city, education, lifetime smoking history, lifetime history of STIs (excluding HIV and anogenital warts) and number of condomless receptive anal sex encounters in the past 6 months	Cross‐sectional; *age at outcome
Chow 2019‐AUS	Gardasil (Merck quadrivalent)	Male, 17 to 19 years*	Vaccinated: 146 Unvaccinated: 152	Prevalence ratio (Penile HPV 6/11/16/18/31/33/45/52/58; 2014‐5 vs 2016‐7)	0.58 (0.22 to 1.51)	Age and source of recruitment	Repeated cross‐sectional; *age at outcome
De Souza 2023‐AUS	Gardasil (Merck quadrivalent); Gardasil 9 (Merck nonavalent)	Female and male, 18 to 70 years	Vaccinated: 230 Unvaccinated: 671	Relative risk (oral; HPV 6/11/16/18/31/33/45/52/58)	0.25 (0.06 to 1.07)	Unadjusted	Cross‐sectional
Hirth 2017‐USA	Gardasil (Merck quadrivalent)	Female, 18 to 30 years*	Vaccinated: 668 Unvaccinated: 2372	Prevalence ratio (oral HPV 6/11/16/18/31/33/45/52/58)	0.53 (0.23 to 1.25)	Unadjusted	Cross‐sectional; *age at outcome
Laake 2020‐NOR	Gardasil (Merck quadrivalent)	Female, 17 years*	Vaccinated: 6360 Unvaccinated: 5468	Relative risk (HPV 6/11/16/18/31/33/45/52/58)	0.28 (0.24 to 0.34)	Unadjusted	Cross‐sectional; *age at outcome
Latsuzbaia 2019‐LUX	Gardasil (Merck quadrivalent)	Female, 18 to 29 years*	Vaccinated: 216 Unvaccinated: 232	Odds ratio (HPV 6/11/16/18/31/33/45/52/58)	0.81 (0.47 to 1.40)	Number of lifetime sexual partners, last partnership duration and age	Cross‐sectional; *age at outcome
Latsuzbaia 2019‐LUX	Cervarix (GSK bivalent)	Female, 18 to 29 years*	Vaccinated: 216 Unvaccinated: 131	Odds ratio (HPV 6/11/16/18/31/33/45/52/58)	0.29 (0.13 to 0.67)	Number of lifetime sexual partners, last partnership duration and age	Cross‐sectional; *age at outcome
Napolitano 2024‐ITA	Not reported	Female and male, 18 to 30 years	Vaccinated: 490 Unvaccinated: 512	Prevalence ratio (HPV 6/11/16/18/31/33/45/52/58)	0.47 (0.15 to 1.51)	Unadjusted	Cross‐sectional
Schlecht 2016‐USA	Gardasil (Merck quadrivalent)	Female, 12 to 19 years	Vaccinated: 957 Unvaccinated: 182	Incidence rate ratio (HPV 6/11/16/18/31/33/45/52/58)	0.97 (0.61 to 1.54)	Exposure time, all concurrent types, current age, race/ethnicity, lifetime number of sex partners, history of anal sex, recent number of vaginal sex partners, age at first intercourse and sexual experience at time of vaccination	Repeated cross‐sectional; *age at outcome
Schlecht 2016‐USA	Gardasil (Merck quadrivalent)	Female, 12 to 19 years	Vaccinated: 957 Unvaccinated: 182	Incidence rate ratio (anal HPV 6/11/16/18/31/33/45/52/58)	0.64 (0.35 to 1.19)	Exposure time, all concurrent types, current age, race/ethnicity, lifetime number of sex partners, history of anal sex, recent number of vaginal sex partners, age at first intercourse and sexual experience at time of vaccination	Repeated cross‐sectional; *age at outcome
Spinner 2019‐USA	Gardasil (Merck quadrivalent)	Female, 13 to 26 years*	Vaccinated: 865 Unvaccinated: 715	Odds ratio (HPV 6/11/16/18/31/33/45/52/58)	0.18 (0.12 to 0.26)	Enrolment site, age, race, history of STI, age at first intercourse, number of sexual partners, main partner being male, ever had anal sex, condom use and smoking history	Repeated cross‐sectional; *age at outcome
Woestenberg 2020‐NLD	Cervarix (GSK bivalent)	Female, 16 to 24 years*	Vaccinated: 357 Unvaccinated: 191	Vaccine effectiveness (anal HPV 6/11/16/18/31/33/45/52/58)	33.5 (‐0.3 to 55.9)	Age, education level, history of anal sex, number of sex partners in the past 6 months, sexually transmitted infection‐related symptoms and use of hormonal contraceptives	Cross‐sectional; *age at outcome

HPV: human papillomavirus; STI: sexually transmitted infection

**70:** Risk of bias summary: prevalent HPV infection

**Study**	**Confounding**	**Selection**	**Classification of interventions**	**Deviations from intended interventions**	**Missing data**	**Measurement of outcomes**	**Selection of reported result**	**Overall risk of bias**
**Prevalent HPV 16/18 infection**								
Batmunkh 2020‐MNG	Serious	Low	Low	Low	Low	Low	Low	Serious
Batmunkh 2019‐MNG	Serious	Moderate	Moderate	Low	Low	Moderate	Low	Serious
Bobadilla 2024‐PAR	Critical	Moderate	Moderate	Low	Low	Low	Low	Critical
Bogaards 2019‐NLD	Serious	Moderate	Low	Low	Low	Low	Low	Serious
Carnalla 2021‐MEX	Serious	Moderate	Moderate	Low	Low	Low	Low	Serious
Carozzi 2018‐ITA	Serious	Low	Low	Low	Low	Low	Low	Serious
Combita 2021‐COL	Serious	Low	Moderate	Low	Moderate	Moderate	Low	Serious
Cummings 2012‐USA	Serious	Moderate	Serious	Low	Low	Low	Low	Serious
Delere 2014‐DEU	Critical	Moderate	Moderate	Low	Moderate	Low	Low	Critical
Enerly 2019‐NOR	Serious	Low	Low	Low	Moderate	Low	Low	Serious
Feder 2019‐USA	Critical	Moderate	Moderate	Low	Low	Low	Low	Critical
Gonzalez 2020‐ARG	Critical	Moderate	Moderate	Low	Moderate	Low	Low	Critical
Heard 2017‐FRA	Serious	Moderate	Low	Low	Moderate	Low	Low	Serious
Hiramatsu 2021‐JPN	Critical	Serious	Low	Low	Moderate	Low	Low	Critical
Hirth 2017‐USA	Critical	Low	Moderate	Low	Moderate	Low	Low	Critical
Jeannot 2018‐CHE	Serious	Low	Moderate	Low	Moderate	Moderate	Low	Serious
Kahn 2016‐USA	Moderate	Moderate	Low	Low	Low	Low	Low	Moderate
Kitamura 2023‐JPN	Serious	Low	Serious	Low	Moderate	Low	Low	Serious
Kreimer 2011‐CRI	Serious	Moderate	Moderate	Low	Moderate	Low	Low	Serious
Kudo 2019‐JPN	Serious	Low	Low	Low	Serious	Low	Low	Serious
Kumakech 2016‐UGA	Critical	Low	Low	Low	Moderate	Low	Low	Critical
Laake 2020‐NOR	Critical	Low	Low	Low	Low	Low	Low	Critical
Latsuzbaia 2019‐LUX	Serious	Moderate	Low	Low	Moderate	Low	Low	Serious
Lee 2022‐THA	Serious	Low	Low	Low	Low	Low	Low	Serious
Lehtinen 2017a‐FIN	Critical	Moderate	Low	Low	Low	Moderate	Low	Critical
Loenenbach 2023‐DEU	Serious	Low	Moderate	Low	Moderate	Moderate	Low	Serious
Lynge 2020‐DNK	Critical	Serious	Serious	Low	Moderate	Low	Low	Critical
Markowitz 2019‐USA	Serious	Moderate	Low	Low	Low	Low	Low	Serious
Mehanna 2019‐GBR	Serious	Low	Low	Low	Low	Low	Low	Serious
Mesher 2018‐GBR	Serious	Moderate	Low	Low	Moderate	Low	Low	Serious
Napolitano 2024‐ITA	Critical	Low	Moderate	Low	Moderate	Low	Low	Critical
Nilyanimit 2024‐THA	Serious	Low	Low	Low	Moderate	Low	Low	Serious
Palmer 2019‐GBR	Serious	Moderate	Low	Low	Moderate	Low	Low	Serious
Purrinos‐Hermida 2018‐ESP	Serious	Moderate	Moderate	Low	Low	Low	Low	Serious
Reyburn 2023‐FJI	Serious	Low	Low	Low	Moderate	Low	Low	Serious
Saeki 2024‐JPN	Critical	Low	Serious	Low	Low	Low	Low	Critical
Saldanha 2020‐PRT	Critical	Serious	Serious	Low	Low	Low	Low	Critical
Sankaranarayanan 2018‐IND	Serious	Low	Low	Low	Moderate	Low	Low	Serious
Sarr 2019‐CAN	Serious	Low	Moderate	Low	Low	Low	Low	Serious
Tanton 2017‐GBR	Serious	Low	Moderate	Low	Low	Low	Low	Serious
Van Eer 2021‐NLD	Critical	Moderate	Moderate	Low	Moderate	Low	Low	Critical
Wendland 2021‐BRA	Critical	Low	Moderate	Low	Low	Low	Low	Critical
Woestenberg 2020‐NLD	Serious	Serious	Moderate	Low	Low	Low	Low	Serious
Wright 2019‐USA	Serious	Moderate	Moderate	Low	Low	Low	Low	Serious
Huyghe 2023‐BEL	Critical	Moderate	Moderate	Low	Low	Low	Low	Critical
Khoo 2022‐MYS	Critical	Low	Serious	Low	Moderate	Low	Low	Critical
Rebolj 2022‐GBR	Serious	Moderate	Moderate	Low	Low	Low	Low	Serious
Saeki 2024‐JPN	Critical	Low	Serious	Low	Low	Low	Low	Critical

**Prevalent HPV 6/11/16/18 infection**								
Ahrlund‐Richter 2019‐SWE	Critical	Low	Low	Low	Serious	Low	Low	Critical
Abel 2021‐USA	Serious	Low	Moderate	Low	Moderate	Low	Low	Serious
Balgovind 2024‐AUS	Critical	Low	Moderate	Low	Moderate	Low	Low	Critical
Baussano 2021‐RWA/BTN	Serious	Moderate	Moderate	Low	Moderate	Low	Low	Serious
Baussano 2020‐BTN	Serious	Low	Serious	Low	Low	Low	Low	Serious
Berenson 2021‐USA	Critical	Low	Moderate	Low	Moderate	Low	Low	Critical
Bobadilla 2024‐PAR	Critical	Moderate	Moderate	Low	Low	Low	Low	Critical
Carozzi 2018‐ITA	Serious	Low	Low	Low	Low	Low	Low	Serious
Chambers 2022‐CAN	Serious	Moderate	Moderate	Low	Moderate	Low	Low	Serious
Chow 2017‐AUS	Critical	Serious	Serious	Moderate	Moderate	Low	Low	Critical
Chow 2019‐AUS	Serious	Serious	Serious	Moderate	Low	Low	Low	Serious
Chow 2021a‐AUS	Serious	Moderate	Serious	Low	Moderate	Low	Low	Serious
Closson 2020‐USA	Moderate	Low	Moderate	Low	Moderate	Low	Low	Moderate
Combita 2021‐COL	Serious	Low	Moderate	Low	Moderate	Moderate	Low	Serious
Cummings 2012‐USA	Serious	Moderate	Serious	Low	Low	Low	Low	Serious
DeSisto 2024‐USA	Serious	Moderate	Low	Low	Low	Low	Low	Serious
De Souza 2023‐AUS	Critical	Low	Low	Low	Moderate	Low	Low	Critical
Dillner 2018‐EU	Critical	Moderate	Serious	Low	Low	Low	Low	Critical
Enerly 2019‐NOR	Serious	Low	Low	Low	Moderate	Low	Low	Serious
Garland 2018‐AUS	Critical	Moderate	Low	Low	Moderate	Low	Low	Critical
Gonzalez 2020‐ARG	Critical	Moderate	Moderate	Low	Moderate	Low	Low	Critical
Heard 2017‐FRA	Serious	Moderate	Low	Low	Moderate	Low	Low	Serious
Hirth 2017‐USA	Critical	Low	Moderate	Low	Moderate	Low	Low	Critical
Jacot‐Guillarmod 2017‐CHE	Critical	Moderate	Moderate	Low	Moderate	Low	Low	Critical
Kahn 2016‐USA	Moderate	Moderate	Low	Low	Low	Low	Low	Moderate
Laake 2020‐NOR	Critical	Low	Low	Low	Low	Low	Low	Critical
Loenenbach 2023‐DEU	Serious	Low	Moderate	Low	Moderate	Moderate	Low	Serious
Machalek 2018‐AUS	Serious	Moderate	Low	Low	Low	Low	Low	Serious
Markowitz 2020‐USA	Serious	Moderate	Low	Low	Moderate	Low	Low	Serious
Markowitz 2019‐USA	Serious	Moderate	Low	Low	Low	Low	Low	Serious
McDaniel 2020‐USA	Critical	Low	Moderate	Low	Low	Low	Low	Critical
McGregor 2018‐AUS	Critical	Serious	Serious	Low	Low	Low	Low	Critical
Napolitano 2024‐ITA	Critical	Low	Moderate	Low	Moderate	Low	Low	Critical
Rosenblum 2021‐USA	Moderate	Low	Moderate	Low	Low	Low	Low	Moderate
Sankaranarayanan 2018‐IND	Serious	Low	Low	Low	Moderate	Low	Low	Serious
Sarr 2019‐CAN	Serious	Low	Moderate	Low	Low	Low	Low	Serious
Sayinzoga 2023‐RWA	Serious	Low	Moderate	Low	Low	Low	Low	Serious
Schlecht 2016‐USA	Serious	Moderate	Low	Low	Low	Low	Low	Serious
Schlecht 2019‐USA	Serious	Moderate	Low	Low	Low	Low	Low	Serious
Shilling 2021‐AUS	Serious	Moderate	Low	Low	Low	Low	Low	Serious
Soderlund‐Strand 2014‐SWE	Critical	Low	Serious	Low	Low	Low	Low	Critical
Spinner 2019‐USA	Serious	Moderate	Low	Low	Low	Low	Low	Serious
Subasinghe 2020‐AUS	Critical	Serious	Low	Low	Serious	Low	Low	Critical
Tabrizi 2014‐AUS	Critical	Moderate	Low	Low	Low	Low	Low	Critical
Wendland 2021‐BRA	Critical	Low	Moderate	Low	Low	Low	Low	Critical
Widdice 2019‐USA	Critical	Moderate	Low	Low	Moderate	Low	Low	Critical
Winer 2021‐USA	Serious	Moderate	Moderate	Low	Moderate	Low	Low	Serious
Wissing 2019‐CAN	Serious	Moderate	Moderate	Low	Low	Low	Low	Serious
Khoo 2022‐MYS	Critical	Low	Serious	Low	Moderate	Low	Low	Critical

**Prevalent HPV 31/33/45/52/58 infection**								
Abel 2021‐USA	Serious	Low	Moderate	Low	Moderate	Low	Low	Serious
DeSisto 2024‐USA	Serious	Moderate	Low	Low	Low	Low	Low	Serious
Mesher 2018‐GBR	Serious	Moderate	Low	Low	Moderate	Low	Low	Serious
Rosenblum 2021‐USA	Moderate	Low	Moderate	Low	Low	Low	Low	Moderate
Spinner 2019‐USA	Serious	Moderate	Low	Low	Low	Low	Low	Serious
Tanton 2017‐GBR	Serious	Low	Moderate	Low	Low	Low	Low	Serious
Khoo 2022‐MYS	Critical	Low	Serious	Low	Moderate	Low	Low	Critical
								
**Prevalent HPV 6/11/16/18/31/33/45/52/58 infection**								
Berenson 2021‐USA	Critical	Low	Moderate	Low	Moderate	Low	Low	Critical
Chambers 2022‐CAN	Serious	Moderate	Moderate	Low	Moderate	Low	Low	Serious
Chow 2019‐AUS	Serious	Serious	Moderate	Moderate	Low	Low	Low	Serious
De Souza 2023‐AUS	Critical	Low	Low	Low	Moderate	Low	Low	Critical
Hirth 2017‐USA	Serious	Low	Moderate	Low	Moderate	Low	Low	Serious
Laake 2020‐NOR	Critical	Low	Low	Low	Low	Low	Low	Critical
Latsuzbaia 2019‐LUX	Serious	Moderate	Low	Low	Moderate	Low	Low	Serious
Napolitano 2024‐ITA	Critical	Low	Moderate	Low	Moderate	Low	Low	Critical
Schlecht 2016‐USA	Serious	Moderate	Low	Low	Low	Low	Low	Serious
Spinner 2019‐USA	Serious	Moderate	Low	Low	Low	Low	Low	Serious
Woestenberg 2020‐NLD	Serious	Serious	Moderate	Low	Low	Low	Low	Serious

HPV: human papillomavirus

*HPV 16/18*

Forty‐six studies were included that reported on prevalent HPV 16/18 infection following HPV vaccination (Batmunkh 2020‐MNG; Bobadilla 2024‐PAR; Bogaards 2019‐NLD; Carnalla 2021‐MEX; Carozzi 2018‐ITA; Combita 2021‐COL; Cummings 2012‐USA; Delere 2014‐DEU; Enerly 2019‐NOR; Feder 2019‐USA; Gonzalez 2020‐ARG; Heard 2017‐FRA; Hiramatsu 2021‐JPN; Hirth 2017‐USA; Huyghe 2023‐BEL; Jeannot 2018‐CHE; Kahn 2016‐USA; Khoo 2022‐MYS; Kitamura 2023‐JPN; Kreimer 2011‐CRI; Kudo 2019‐JPN; Kumakech 2016‐UGA; Laake 2020‐NOR; Latsuzbaia 2019‐LUX; Lee 2022‐THA; Lehtinen 2017a‐FIN; Loenenbach 2023‐DEU; Lynge 2020‐DNK; Markowitz 2019‐USA; Mehanna 2019‐GBR; Mesher 2018‐GBR; Napolitano 2024‐ITA; Nilyanimit 2024‐THA; Palmer 2019‐GBR; Purrinos‐Hermida 2018‐ESP; Rebolj 2022‐GBR; Reyburn 2023‐FJI; Saeki 2024‐JPN; Saldanha 2020‐PRT; Sankaranarayanan 2018‐IND; Sarr 2019‐CAN; Tanton 2017‐GBR; Van Eer 2021‐NLD; Wendland 2021‐BRA; Woestenberg 2020‐NLD; Wright 2019‐USA).

The type of effect estimate reported varied across studies, but almost all studies reported a reduction in HPV genital 16/18 infection with HPV vaccine. Three studies reported on oral HPV 16/18 infection (Hirth 2017‐USA; Mehanna 2019‐GBR; Sankaranarayanan 2018‐IND). All three studies reported a reduction in prevalence following HPV vaccination but had confidence intervals that included no effect. One study reported a reduction of anal HPV 16/18 infection with a vaccine effectiveness of 89.9% (63.0% to 97.2%) (Woestenberg 2020‐NLD).

Four studies reported on the effect of two doses or one dose of HPV vaccine on HPV 16/18 infection (Batmunkh 2020‐MNG; Kreimer 2011‐CRI; Palmer 2019‐GBR; Reyburn 2023‐FJI). The studies reported a reduction in HPV 16/18 infection following vaccination with two doses or one dose.

*HPV 6/11/16/18*

Forty‐nine studies were included that reported on prevalent HPV 6/11/16/18 infection following HPV vaccination (Ahrlund‐Richter 2019‐SWE; Abel 2021‐USA; Balgovind 2024‐AUS; Baussano 2021‐RWA/BTN; Baussano 2020‐BTN; Berenson 2021‐USA; Bobadilla 2024‐PAR; Carozzi 2018‐ITA; Chambers 2022‐CAN; Chow 2017‐AUS; Chow 2019‐AUS; Chow 2021a‐AUS; Closson 2020‐USA; Combita 2021‐COL; Cummings 2012‐USA; De Souza 2023‐AUS; DeSisto 2024‐USA; Dillner 2018‐EU; Enerly 2019‐NOR; Garland 2018‐AUS; Goggin 2018‐CAN; Gonzalez 2020‐ARG; Heard 2017‐FRA; Hirth 2017‐USA; Jacot‐Guillarmod 2017‐CHE; Kahn 2016‐USA; Khoo 2022‐MYS; Laake 2020‐NOR; Machalek 2018‐AUS; Markowitz 2020‐USA; Markowitz 2019‐USA; McDaniel 2020‐USA; McGregor 2018‐AUS; Napolitano 2024‐ITA; Rosenblum 2021‐USA; Sankaranarayanan 2018‐IND; Sarr 2019‐CAN; Sayinzoga 2023‐RWA; Schlecht 2016‐USA; Schlecht 2019‐USA; Shilling 2021‐AUS; Soderlund‐Strand 2014‐SWE; Spinner 2019‐USA; Subasinghe 2020‐AUS; Tabrizi 2014‐AUS; Wendland 2021‐BRA; Widdice 2019‐USA; Winer 2021‐USA; Wissing 2019‐CAN).

The type of effect estimate reported varied across studies, but almost all studies reported a reduction in genital HPV 6/11/16/18 infection with HPV vaccine. Nine studies reported on oral HPV 6/11/16/18 (Berenson 2021‐USA; Chow 2021a‐AUS; De Souza 2023‐AUS; Hirth 2017‐USA; McDaniel 2020‐USA; Rosenblum 2021‐USA; Sankaranarayanan 2018‐IND; Schlecht 2019‐USA; Winer 2021‐USA) and all except one study (McDaniel 2020‐USA) reported a reduced prevalence following vaccination. One study reported a decrease in oral HPV prevalence in males but not in females (Berenson 2021‐USA). Three studies reported that anal HPV 6/11/16/18 prevalence in males decreased with HPV vaccination (Chambers 2022‐CAN; Chow 2021a‐AUS; Winer 2021‐USA). One study reported the effect was more pronounced in males receiving the vaccine at a younger age (Chambers 2022‐CAN). One study reported a reduction in anal HPV 6/11/16/18 prevalence in females following HPV vaccination (Schlecht 2016‐USA). Three studies reported a reduction in penile HPV 6/11/16/18 prevalence in males following vaccination (Chow 2019‐AUS; Chow 2021a‐AUS; Winer 2021‐USA).

Five studies reported on the effect of two doses or one dose of HPV vaccine on HPV 6/11/16/18 infection (Abel 2021‐USA; Chambers 2022‐CAN; Markowitz 2020‐USA; Rosenblum 2021‐USA; Widdice 2019‐USA). Three studies reported no effect of two doses or one dose (Abel 2021‐USA; Chambers 2022‐CAN; Widdice 2019‐USA), while two studies reported a reduced prevalence following at least one dose (Markowitz 2020‐USA; Rosenblum 2021‐USA). One study reported that effectiveness varied according to age at first vaccination (Markowitz 2020‐USA).

*HPV 31/33/45/52/58*

Seven studies were included that reported on prevalent HPV 31/33/45/52/58 infection following HPV vaccination (Abel 2021‐USA; DeSisto 2024‐USA; Khoo 2022‐MYS; Mesher 2018‐GBR; Rosenblum 2021‐USA; Spinner 2019‐USA; Tanton 2017‐GBR). Three studies reported a reduction in HPV 31/33/45/52/58 infection following HPV vaccination (DeSisto 2024‐USA; Rosenblum 2021‐USA; Spinner 2019‐USA). Only one of these studies reported on the effectiveness of the 9‐valent HPV vaccine, which includes these HPV subtypes (DeSisto 2024‐USA). The prevalence ratio for anal HPV 31/33/45/52/58 infection in men who have sex with men was 0.73 (95% CI 0.62 to 0.85) following HPV vaccination.

*HPV 6/11/16/18/31/33/45/52/58*

Eleven studies were included that reported on prevalent HPV 6/11/16/18/31/33/45/52/58 infection following HPV vaccination (Berenson 2021‐USA; Chambers 2022‐CAN; Chow 2019‐AUS; De Souza 2023‐AUS; Hirth 2017‐USA; Laake 2020‐NOR; Latsuzbaia 2019‐LUX; Napolitano 2024‐ITA; Schlecht 2016‐USA; Spinner 2019‐USA; Woestenberg 2020‐NLD). Five studies reported a reduction of prevalence following HPV vaccination (Chambers 2022‐CAN; De Souza 2023‐AUS; Laake 2020‐NOR; Latsuzbaia 2019‐LUX; Spinner 2019‐USA).

## Discussion

### Summary of main results

We included 225 studies from 347 records in this review. We included 86 cohort studies, four case‐control studies, 46 cross‐sectional studies, 69 pre‐post vaccine introduction studies, five RCT extensions and two self‐controlled case series. Thirteen additional studies reported on more than one type of analysis. Of the included studies, 177 reported on only females, 11 only males, and 37 a combination of males and females. Risk of bias ranged from overall low risk of bias in the self‐controlled case series to moderate, serious and critical risk of bias in the other study designs.

### Clinical outcomes

There was moderate‐certainty evidence that HPV vaccination reduces the incidence of cervical cancer. Meta‐analysis of cohort studies with effect estimates adjusted for confounding showed a reduced risk of cervical cancer following HPV vaccination (RR 0.37, 95% CI 0.25 to 0.56). Six studies of different designs reported no cases of cervical cancer in the HPV vaccine groups. Eight pre‐post vaccine introduction studies reported a reduction in cervical cancer incidence following HPV vaccine introduction.

There was moderate‐certainty evidence that HPV vaccination reduces the incidence of CIN3+. Eleven of 12 cohort studies reported a reduced risk of CIN3+ following HPV vaccination. Eight studies of different designs reported a decrease in CIN3+ incidence in HPV vaccinated participants. One other study reported no difference in the risk of CIN3+. Three pre‐post vaccine introduction studies reported a decrease in CIN3+ incidence following HPV vaccine introduction.

There was low‐certainty evidence that HPV vaccination reduces the incidence of vaginal cancer, penile cancer, head and neck cancer, VaIN and AIN.

There was only very low‐certainty evidence on the effect of HPV vaccination on the incidence of AIS, vulval cancer, anal cancer in males or females, and VIN.

There was moderate‐certainty evidence that HPV vaccination reduces the incidence of CIN3. One cohort study and a case‐control study reported a reduced risk of CIN3 following HPV vaccination. Two cross‐sectional studies reported no difference in the risk of CIN3 in vaccinated and unvaccinated participants. Four pre‐post vaccine introduction studies reported a reduction in CIN3 incidence following HPV introduction and one study reported an increased risk.

There was moderate‐certainty evidence that HPV vaccination reduces the incidence of CIN2+. Twelve cohort studies, three case‐control studies, three cross‐sectional studies and one RCT extension study reported a reduced risk of CIN2+ following HPV vaccination. Five pre‐post vaccine introduction studies reported a reduction in CIN2+ incidence following HPV introduction and one study reported an increased incidence.

There was moderate‐certainty evidence that HPV vaccination reduces the incidence of CIN2. Three cohort studies and one case‐control study reported a reduced risk of CIN2 following HPV vaccination. Two cross‐sectional studies reported no difference in risk of CIN2 between vaccinated and unvaccinated participants. Three pre‐post vaccine introduction studies reported a reduction in CIN2 incidence following HPV vaccine introduction.

There was moderate‐certainty evidence that HPV vaccination reduces the incidence of anogenital warts. Thirteen from 15 cohort studies reported a reduced risk of anogenital warts in vaccinated compared with unvaccinated participants. Twenty‐five pre‐post vaccine introduction studies reported a decrease in anogenital warts incidence following the introduction of HPV vaccine. Six studies reported no difference in anogenital warts incidence.

### Specific adverse events

Across a range of study designs, there was moderate‐certainty evidence that HPV vaccination likely does not increase the risk of POTS, CFS/ME, paralysis, CRPS, premature ovarian failure, infertility or sexual activity. There was low‐certainty evidence that suggests HPV vaccination does not increase the risk of Guillain‐Barré syndrome.

### Completeness

We have performed an extensive review of the published literature and engaged with clinicians and experts in this area to ensure comprehensive coverage of the literature in this field. The included studies reported data from 46 countries. Most of these are high‐income countries that have national HPV vaccination programmes that are often complemented with cervical screening programmes. There are fewer data on the effectiveness of HPV vaccination in lower‐income countries, where cervical cancer is more common and screening programmes are lacking.

The HPV vaccine was only licensed in 2006, so many of the population‐level studies that were included in this review had less than 10 years of follow‐up data. With a longer follow‐up, additional effectiveness questions, such as those around the number of doses required for protection, the effectiveness at different ages of vaccination or the effectiveness in males, can be answered with more confidence.

### Applicability

The design of this review, with its objective to address population‐level impact, is directly related to the limitations of randomised controlled trial data assessing long‐term outcomes such as cancer (Bergman 2025). RCTs are unable to estimate the effects of vaccination strategies at a population level, where reducing the level of infection within a population can benefit both those vaccinated and those unvaccinated, if coverage is sufficient to induce a degree of herd immunity. However, population‐level studies often have less rigorous data collection procedures than RCTs for both the exposure and the outcome, as well as suffering from selection bias with limited opportunity to control for confounding. We refer readers to the companion review for a comprehensive analysis of RCT data on HPV vaccine efficacy (Bergman 2025).

The specific adverse events evaluated in this review were derived from a social media search (Appendix 2) to directly address the concerns of the public with regard to HPV vaccination. There is little evidence to suggest an association between HPV vaccines and the most mentioned adverse effects from social media.

### Equality and diversity

Importantly, these data come largely from high‐income countries, whereas cervical cancer is predominately a disease of low‐ to middle‐income countries (WHO 2020). Improved vaccination and cervical screening coverage, especially in countries that lack resources for organised population‐level cervical cancer screening programmes, will be vital to achieve the WHO ambition for the elimination of cervical cancer in our lifetime (WHO 2023).

### Quality of the evidence

The certainty of the evidence for different outcomes ranged from very low to moderate. In many cases, we downgraded the certainty due to limitations in study design. Overall risk of bias for the primary and secondary outcomes ranged from moderate risk to critical risk of bias. The observational and retrospective designs of most studies contributed to the high risk of bias. In retrospective studies, controlling for confounding between vaccinated and unvaccinated groups becomes challenging, especially when additional characteristics of the population are unknown or unrecorded. Many studies were carried out using routine healthcare administrative or insurance databases which, while large and rich in clinical data, are retrospective and can suffer from potential risk of measurement and outcome bias. We were unable to assess outcome reporting bias (failing to report on a planned outcome) for the included studies because most observational studies are not pre‐registered and often lack study protocols or statistical analysis plans.

For some outcomes, we downgraded the certainty of evidence by one level for inconsistency. This occurred when the effect estimates in the included studies were in different directions; that is, studies showed a combination of no effect, a possible harm and a possible benefit of HPV vaccination.

We did not downgrade any outcomes for indirectness. The outcomes were prespecified and only studies reporting one of the outcomes were included. The inclusion criteria of the review ensured that only the intervention and population of interest were considered.

Despite the large sample size of many included studies, we downgraded for imprecision if there were no or unclear numbers of outcome events.

### Multiple data sets

We identified 347 published records for inclusion in this review, which were combined into 225 unique 'studies' or, more specifically, 'data sets'. We checked all records for overlapping sources of data (i.e. insurance databases or national registers) and dates to ensure participants and outcomes were only included once. This process limits the number of effectiveness estimates that can be derived from the same databases. However, this did involve selection of the most representative population and effect estimate that closely fit the outcomes of interest for this review.

We focussed on extracting effect estimates for different ages at vaccination, however this was often not reported or reported in inconsistent age groups. Further insight into the effectiveness of HPV vaccination at different ages of vaccination could be gained from re‐analysis of the original data sets in consistent age groups, as seen in other reviews (Drolet 2019).

We did not stratify analyses by type of vaccine because there were many more effectiveness estimates available for Gardasil than Cervarix or other HPV vaccines. Some studies did not specify the HPV vaccine in use or reported effectiveness estimates of the different vaccines combined.

### Synthesis of evidence from different study types

By including a range of different observational study designs, we encountered challenges in combining data across studies and synthesising evidence for an outcome. The different designs provide insight into different aspects of HPV vaccine effectiveness.

### Agreements and disagreements with other studies or reviews

The results of this review align closely with other systematic reviews of population‐level impact of the HPV vaccine (Drolet 2019; Ellingson 2023; Wang 2022).

In a large systematic review of the population‐based impact of HPV vaccination, Drolet 2019 reported that anogenital wart diagnoses decreased by 67% (RR 0.33, 95% CI 0.24 to 0.46) among girls aged 15 to 19 years, and 31% to 54% in older women. Our review had similar results when limiting the analysis to those receiving vaccination at or before 16 years (RR 0.30, 95% CI 0.20 to 0.43). Drolet 2019 also reported that CIN2+ decreased by 51% (RR 0.49, 95% CI 0.42 to 0.58) among screened girls aged 15 to 19 years and by 31% (RR 0.69, 95% CI 0.57 to 0.84) among women aged 20 to 24 years. Our results indicated a reduction of 62% (RR 0.38, 95% CI 0.31 to 0.45) in those receiving vaccination at or before 16 years.

In a review of the real‐world impact of the quadrivalent HPV vaccine, Wang 2022 reported reductions in infection, anogenital warts and cervical lesions across different regions of the world, similar to the results of the current review. Another review by Ellingson 2023 evaluated the effectiveness of HPV vaccine by age at vaccination. Results were similar to the sensitivity analysis in the current review, with the highest vaccine effectiveness in the youngest age group (9 to 14 years).

While the analytic approach between existing systematic reviews (Drolet 2019; Ellingson 2023; Wang 2022) and the current one differ, the overall results of the impact of HPV vaccination on genital HPV infection, anogenital warts and cervical lesions are similar.

The evaluation of specific adverse events that are commonly discussed on social media has been more limited than vaccine effectiveness outcomes. These events are rare and often not evaluated in clinical trials (Jørgensen 2020). We have attempted to prospectively identify all studies reporting on these specific adverse events and almost all studies did not report any association between HPV vaccination and these events.

## Authors' conclusions

**Implications for practice**

There are now long‐term outcome data from different countries and of different study designs that consistently demonstrate a probable reduction in the development of high‐grade cervical intraepithelial neoplasia (CIN) and cervical cancer in females vaccinated against human papillomavirus (HPV) in early adolescence. Data show that there is greater benefit to vaccinating younger adolescents prior to sexual debut, before most are exposed to high‐risk human papillomavirus (hrHPV) through sexual activity, whilst the benefit from vaccinating adults, untested for hrHPV, at a population level is minimal.

Data are now mature enough to see a beneficial effect of HPV vaccination, which probably reduces cervical cancer rates. Other HPV‐related cancers have a longer natural history, and it will take many more years, or even decades, to understand the impact of HPV vaccination on vulval, peri‐anal, and head and neck cancer diagnoses.

Data also show that HPV vaccination probably reduces the incidence of anogenital warts.

This will, most probably, result in a reduction in rates of high‐grade CIN (CIN2 or worse, CIN2+) and fewer HPV infections, and it will mean that cervical screening programmes will need to consider adapting, in order to remain cost‐effective. The introduction of primary HPV testing by some programmes has already enabled a change in screening intervals to five‐yearly (Morgan 2022; Public Health Scotland 2022), and these intervals could be even longer for those vaccinated in early adolescence (Rebolj 2022). This will have implications for service delivery at a laboratory level, especially in systems that employ HPV‐triage testing, since many fewer cytology samples will be screened, and in the delivery of colposcopy and cervical cancer care, including the training of healthcare professionals. Screening databases may also need to collate vaccination data to allow more personalised, adaptive screening.

Importantly, these data come largely from high‐income countries, whereas cervical cancer is predominately a disease of low‐ to middle‐income countries (WHO 2020). Improved vaccination coverage, especially in countries that lack resources for organised population‐level cervical cancer screening programmes, will be vital to achieve the World Health Organization (WHO) ambition for the elimination of cervical cancer in our lifetime (WHO 2023).

**Implications for research**

The results of this review complement those of the parallel systematic review and network meta‐analysis of randomised controlled trial (RCT)‐level data for HPV vaccination (Bergman 2025). Taken together, these results demonstrate that RCTs alone are unable to answer important questions in research. These two reviews highlight the difficulty for RCTs alone to detect very rare harms and long‐term beneficial and adverse outcomes. RCTs, due to restrictions of time and funding, commonly have follow‐up time periods that are too short, and they are not powered to detect rarer outcomes in diseases with long natural histories or for effects on outcomes that happen later in life, for example cancers and pregnancy outcomes; these important outcomes are unlikely to be picked up in RCT studies of childhood vaccination.

Identification of valid short‐term surrogate markers for longer‐term important clinical endpoints is vital to avoid the significant harms of missing prevention opportunities over many years, or exposing people to unnecessary interventions, should they not work in practice. The decision to use HPV antibody levels, infection rates and development of CIN2+ as surrogate endpoints for cancer outcomes in these population‐level studies has proven legitimate (IARC 2014). If we had waited for evidence of effect on cervical cancer outcomes: 1) we would have had many years of lost opportunity to prevent death and disease; and 2) studies would have needed to be extremely large (and expensive) in order to demonstrate effects on rare outcomes in well‐screened study populations. Use of surrogate endpoints, therefore, has the potential to prevent avoidable harm and waste of health and research resources. However, these endpoints need to be based on the natural history of the disease and correlate well with the clinical outcomes we wish to measure, rather than be merely more convenient and cheaper.

Another limitation of the RCTs in HPV vaccination is that these studies were not performed in younger adolescents. This was due to the design of several studies, which required HPV testing from vaginal samples. Those that were performed concentrated on immunological outcomes, e.g. neutralising antibody titres. This therefore means that RCTs may underestimate the true effect of the intervention on the more ideal target: the prepubertal population that is likely to benefit the most.

One challenge for both this review and the parallel network meta‐analysis is the lack of standardisation of outcome measures and time points for measurement. This has made combining outcomes difficult, and often impossible, which limits the certainty of our conclusions. This is a shame and the development, and consistent implementation, of core outcome measures that are reported at agreed time points, is required with urgency in this area and many others.

Quality improvement (QI) methodology, including statistical process charts (SPC), may be better able to demonstrate trends and effects of interventions over time (Benneyan 2003). However, QI methodology on its own may not be able to exclude the possibility that change is due to other effects, unless used in parallel with more conventional cohort or case‐control studies. Combining these different types of studies to give a deeper understanding of the effects on long‐term health outcomes is an important challenge for methodologists.

## History

Protocol first published: Issue 5, 2022

## Appendices

### Appendix 1. Study design definitions

#### Population‐level studies

Pre‐ versus post‐vaccine introduction studies: a type of ecologic study that focuses on the comparison of groups, rather than individuals. Studies compare the frequency of an outcome between pre‐vaccination and post‐vaccination periods among the general population and should use the same population source and recruitment methods before and after vaccination. These types of studies are often considered to evaluate the 'impact' of vaccine introduction.

Interrupted time‐series study (ITS): a study that uses observations at multiple time points before and after an intervention (the ‘interruption’). The design attempts to detect whether the intervention, in this case HPV vaccine introduction, has had an effect significantly greater than any underlying trend over time (Reeves 2022).

Controlled before‐and‐after study (CBA): a study in which observations are made before and after the implementation of an intervention, both in a group that receives the intervention and in a control group that does not.

#### Individual‐level studies

Prospective cohort study/retrospective cohort study: an epidemiological study where groups of individuals are identified who vary in their exposure to an intervention or hazard and are followed to assess outcomes. Association between exposure and outcome are then estimated. Cohort studies are best performed prospectively (prospective cohort study) but can also be undertaken retrospectively (retrospective cohort study) if suitable data records are available. We will consider non‐randomised comparative studies, e.g. comparisons of a vaccinated group with an unvaccinated group, as a type of cohort study.

Cross‐sectional study: an epidemiological study that measures exposure and outcome at the same time. It reports the prevalence of exposure and outcome, and their associations, at a single point in time.

Case‐control study: an epidemiological study usually used to investigate the causes of disease. Study participants who have experienced an adverse outcome or disease are compared with participants who have not. Any differences in the presence or absence of hypothesised risk factors are noted.

Self‐controlled case series study (SCCS): uses individuals as their own controls. The ages at vaccination are regarded as fixed, and the age at the time of an adverse event is the random variable of interest within a predetermined observation period (Farrington 2004; Petersen 2016).

### Appendix 2. Analysis of social media reporting of HPV vaccine adverse events

We sought to identify adverse events that were potentially related to HPV vaccination, which were commonly mentioned on social media.

Firstly, we screened all of the reviews on WebMD of HPV vaccines to identify mentions of adverse events. We coded each mention of a personal experience where possible to MedDRA preferred terms.

There were 276 adverse events mentioned and annotated. The most common adverse events were injection site pain, headaches and missed periods.

**Table:**

**WebMD adverse event mentions** **(rank order of frequency)**	**Adverse event**
1	injection site pain
2	headache
3	missing periods
4	dizziness
5	fatigue
6	nausea
7	myalgia
8	fever
9	malaise
10	pain
11	syncope
12	abdominal pain
13	influenza‐like illness
14	alopecia
15	cramping
16	dyspnoea
17	rash
18	tremor
19	vomiting
20	anxiety
21	arthralgia
22	chest pain
23	cough
24	diarrhoea
25	infertility
26	syncope (recurrent)
27	tingling
28	aluminium toxicity
29	back pain
30	death
31	dehydration
32	hives
33	hypoaesthesia
34	insomnia
35	migraine
36	shoulder pain
37	swollen glands
38	seizure
39	auto‐immune disease

We also investigated an analysis of 'Tweets' on X (formerly Twitter). Recent news events with the release of the results of a clinical trial and activity on Twitter related to the COVID‐19 vaccines meant that recent posts suffered from a lot of noise. Many posts mentioning adverse events were also doing so to promote an anti‐HPV vaccination stance rather than personal experience, with accounts dedicated to promoting HPV side effect information (@HPVSideEffects) and reference to the vaccine as ‘Human Paralysis inducing Vaccine’. Refusal of the vaccine was also stated to be related to parents not wanting to promote sexual activity in their children.

We were able to uncover 46 recent adverse event experience mentions.

**Table:**

**Twitter adverse event mentions** **(rank order of frequency)**	**Adverse event**
1	death
2	auto‐immune disease
3	chronic fatigue syndrome
4	inability to walk
5	infertility
6	myalgic encephalomyelitis
7	paralysed
8	seizures/epilepsy
9	tremors
10	aluminium toxicity
11	anxiety
12	chronic kidney disease
13	encephalitis
14	epilepsy
15	Epstein Barr
16	functional neurologic disorder
17	Hashimoto's disease
18	heart problem
19	missing periods
20	myocarditis
21	nervous breakdown
22	pain
23	postural orthostatic tachycardia syndrome
24	stuttering
25	syncope
26	systemic lupus erythematosus
27	weakness
28	amyotrophic lateral sclerosis

### Appendix 3. MEDLINE search strategy

1. exp Papillomavirus Vaccines/ 2. gardasil*.mp. 3. (cervarix* or cecolin*).mp. 4. ((human papilloma virus* or human papiloma virus*) adj (vaccin* or immuni*)).tw. 5. ((human papillomavirus* or human papilomavirus*) adj (vaccin* or immuni*)).tw. 6. (HPV* adj3 (vaccin* or immuni*)).tw. 7. 1 or 2 or 3 or 4 or 5 or 6 8. ae.fs. 9. safe*.ti,ab. 10. de.fs. 11. adverse.ti,ab. 12. co.fs. 13. side effect*.ti,ab. 14. complication*.ti,ab. 15. ci.fs. 16. tolerated.ti,ab. 17. tolerance.ti,ab. 18. harm*.ti,ab. 19. toxicity.ti,ab. 20. risk.ti. 21. Pregnancy complications/dt 22. Clinical trial phase IV.pt. 23. Drug hypersensitivity/ 24. Tolerability.ti,ab. 25. to.fs. 26. toxicology/ 27. Drug induced.ti,ab. 28. Negative effects.ti,ab. 29. 8 or 9 or 10 or 11 or 12 or 13 or 14 or 15 or 16 or 17 or 18 or 19 or 20 or 21 or 22 or 23 or 24 or 25 or 26 or 27 or 28 30. exp cohort studies/ or exp epidemiologic studies/ or exp clinical trial/ or exp evaluation studies as topic/ or exp statistics as topic/ 31. (control and (group* or study)).mp. 32. (time and factors).mp. 33. Program.mp. 34. survey*.mp. 35. ci.mp. 36. cohort.mp. 37. (comparative stud* or prospective* or retrospective* or longitudinal*).mp. 38. evaluation studies.mp. 39. 30 or 31 or 32 or 33 or 34 or 35 or 36 or 37 or 38 40. (animals/ not humans/) or comment/ or editorial/ or exp review/ or meta analysis/ or consensus/ or exp guideline/ 41. case report.mp. 42. 40 or 41 43. 39 not 42 44. 7 and 29 45. 43 and 44

### Appendix 4. Embase search strategy

1. exp Wart virus vaccine/

2. gardasil*.mp.

3. cervarix*.mp.

4. ((human papilloma virus* or human papiloma virus*) adj (vaccin* or immuni*)).tw.

5. ((human papillomavirus* or human papilomavirus*) adj (vaccin* or immuni*)).tw.

6. (HPV* adj3 (vaccin* or immuni*)).tw.

7. 1 or 2 or 3 or 4 or 5 or 6

8. exp Papillomavirus Infection/

9. exp Papillomaviridae/

10. (HPV* or papilloma*).ti,ab.

11. uterine cervix carcinoma in situ/

12. Uterine Cervical Dysplasia/

13. (CIN* or adenocarcinoma in situ or AIS).ti,ab.

14. (cervi* adj5 (wart* or infection* or condyloma* or neoplas* or dysplas* or lesion* or cancer* or precancer* or "pre‐cancer*" or "pre‐invasive" or preinvasive or "intra‐epithel*" or intraepithelial* or disease* or maligna*)).ti,ab.

15. ($genit* adj5 (wart* or infection* or condyloma* or neoplas* or dysplas* or lesion* or cancer* or precancer* or "pre‐cancer*" or "pre‐invasive" or preinvasive or "intra‐epithel*" or intraepithelial* or disease* or maligna*)).ti,ab.

16. (vagina* adj5 (wart* or infection* or condyloma* or neoplas* or dysplas* or lesion* or cancer* or precancer* or "pre‐cancer*" or "pre‐invasive" or preinvasive or "intra‐epithel*" or intraepithelial* or disease* or maligna*)).ti,ab.

17. (vulv* adj5 (wart* or infection* or condyloma* or neoplas* or dysplas* or lesion* or cancer* or precancer* or "pre‐cancer*" or "pre‐invasive" or preinvasive or "intra‐epithel*" or intraepithelial* or disease* or maligna*)).ti,ab.

18. (anal* adj5 (wart* or infection* or condyloma* or neoplas* or dysplas* or lesion* or cancer* or precancer* or "pre‐cancer*" or "pre‐invasive" or preinvasive or "intra‐epithel*" or intraepithelial* or disease* or maligna*)).ti,ab.

19. ((head or neck) adj5 (neoplas* or dysplas* or lesion* or cancer* or precancer* or "pre‐cancer*" or "pre‐invasive" or preinvasive or disease* or maligna*)).ti,ab.

20. (penile* adj5 (wart* or infection* or neoplas* or dysplas* or lesion* or cancer* or precancer* or "pre‐cancer*" or "pre‐invasive" or preinvasive or "intra‐epithel*" or intraepithelial* or disease* or maligna*)).ti,ab.

21. Uterine Cervix Tumor/

22. exp Condylomata Acuminata/

23. vulva tumor/ or vagina tumor/ or anus tumor/ or anus disease/

24. "Head and Neck Neoplasms"/

25. penis tumor/ or penis disease/

26. Postural Orthostatic Tachycardia Syndrome/

27. (postural tachycardia syndrome* or postural orthostatic tachycardia syndrome* or POTS).mp.

28. Chronic Fatigue Syndrome/

29. chronic fatigue*.mp.

30. (myalgic encephalomyelitis or ME or chronic fatigue* or CFS).mp.

31. Paralysis/

32. paralys*.mp.

33. Complex Regional Pain Syndrome/

34. (complex regional pain syndrome or CRPS).mp.

35. premature ovarian failure/

36. ((premature ovar* or primary ovar*) adj2 (fail* or insufficien*)).mp.

37. Guillain‐Barre Syndrome/

38. (Guillain Barr* syndrome or GBS).mp.

39. Infertility/

40. infertil*.mp.

41. Sexual Behavior/

42. (earl* adj3 (sex* activity or sex* behaviour)).mp.

43. 8 or 9 or 10 or 11 or 12 or 13 or 14 or 15 or 16 or 17 or 18 or 19 or 20 or 21 or 22 or 23 or 24 or 25 or 26 or 27 or 28 or 29 or 30 or 31 or 32 or 33 or 34 or 35 or 36 or 37 or 38 or 39 or 40 or 41 or 42

44. ae.fs.

45. Adverse.ti,ab,kw,ox.

46. Safe*.ti,ab,kw.

47. Po.fs.

48. Co.fs.

49. exp adverse drug reaction/

50. Complication*.ti,ab,kw.

51. Drug safety/

52. To.fs.

53. Side effect*.ti,ab.

54. Risk.ti.

55. Tolerance.ti,ab.

56. Tolerated.ti,ab.

57. Harm.ti,ab.

58. Side reaction*.ti,ab.

59. drug withdrawal/

60. health risks.ti,ab.

61. potential risks.ti,ab.

62. toxic effects.ti,ab.

63. toxicity.ti,ab.

64. toxicities.ti,ab.

65. 44 or 45 or 46 or 47 or 48 or 49 or 50 or 51 or 52 or 53 or 54 or 55 or 56 or 57 or 58 or 59 or 60 or 61 or 62 or 63 or 64

66. 43 or 65

67. 7 and 66

### Appendix 5. CENTRAL search strategy

#1 MeSH descriptor: [Papillomavirus Vaccines] explode all trees

#2 gardasil*

#3 cervarix*

#4 ((human papilloma virus* or human papiloma virus*) near (vaccin* or immuni*))

#5 ((human papillomavirus* or human papilomavirus*) near (vaccin* or immuni*))

#6 (HPV* near/3 (vaccin* or immuni*))

#7 #1 or #2 or #3 or #4 or #5 or #6

#8 MeSH descriptor: [Papillomavirus Infections] explode all trees

#9 MeSH descriptor: [Papillomaviridae] explode all trees

#10 (HPV* or papilloma*)

#11 MeSH descriptor: [Cervical Intraepithelial Neoplasia] this term only

#12 MeSH descriptor: [Uterine Cervical Dysplasia] this term only

#13 (CIN* or adenocarcinoma in situ or AIS)

#14 (cervi* near/5 (wart* or infection* or condyloma* or neoplas* or dysplas* or lesion* or cancer* or precancer* or "pre‐cancer*" or "pre‐invasive" or preinvasive or "intra‐epithel*" or intraepithelial* or disease* or maligna*))

#15 ($genit* near/5 (wart* or infection* or condyloma* or neoplas* or dysplas* or lesion* or cancer* or precancer* or "pre‐cancer*" or "pre‐invasive" or preinvasive or "intra‐epithel*" or intraepithelial* or disease* or maligna*))

#16 (vagina* near/5 (wart* or infection* or condyloma* or neoplas* or dysplas* or lesion* or cancer* or precancer* or "pre‐cancer*" or "pre‐invasive" or preinvasive or "intra‐epithel*" or intraepithelial* or disease* or maligna*))

#17 (vulv* near/5 (wart* or infection* or condyloma* or neoplas* or dysplas* or lesion* or cancer* or precancer* or "pre‐cancer*" or "pre‐invasive" or preinvasive or "intra‐epithel*" or intraepithelial* or disease* or maligna*))

#18 (anal* near/5 (wart* or infection* or condyloma* or neoplas* or dysplas* or lesion* or cancer* or precancer* or "pre‐cancer*" or "pre‐invasive" or preinvasive or "intra‐epithel*" or intraepithelial* or disease* or maligna*))

#19 ((head or neck) near/5 (neoplas* or dysplas* or lesion* or cancer* or precancer* or "pre‐cancer*" or "pre‐invasive" or preinvasive or disease* or maligna*))

#20 (penile* near/5 (wart* or infection* or neoplas* or dysplas* or lesion* or cancer* or precancer* or "pre‐cancer*" or "pre‐invasive" or preinvasive or "intra‐epithel*" or intraepithelial* or disease* or maligna*))

#21 MeSH descriptor: [Uterine Cervical Neoplasms] this term only

#22 MeSH descriptor: [Condylomata Acuminata] explode all trees

#23 MeSH descriptor: [Vulvar Neoplasms] this term only

#24 MeSH descriptor: [Vaginal Neoplasms] this term only

#25 MeSH descriptor: [Anus Neoplasms] explode all trees

#26 MeSH descriptor: [Anus Diseases] this term only

#27 MeSH descriptor: [Head and Neck Neoplasms] this term only

#28 MeSH descriptor: [Penile Neoplasms] this term only

#29 MeSH descriptor: [Penile Diseases] this term only

#30 MeSH descriptor: [Postural Orthostatic Tachycardia Syndrome] this term only

#31 (postural tachycardia syndrome* or postural orthostatic tachycardia syndrome* or POTS)

#32 MeSH descriptor: [Fatigue Syndrome, Chronic] this term only

#33 chronic fatigue*

#34 (myalgic encephalomyelitis or ME or chronic fatigue* or CFS)

#35 MeSH descriptor: [Paralysis] this term only

#36 paralys*

#37 MeSH descriptor: [Complex Regional Pain Syndromes] this term only

#38 complex regional pain syndrome or CRPS

#39 MeSH descriptor: [Primary Ovarian Insufficiency] this term only

#40 ((premature ovar* or primary ovar*) near/2 (fail* or insufficien*))

#41 MeSH descriptor: [Guillain‐Barre Syndrome] this term only

#42 Guillain Barr* syndrome or GBS

#43 MeSH descriptor: [Infertility] this term only

#44 infertil*

#45 MeSH descriptor: [Sexual Behavior] this term only

#46 (earl* near/3 (sex* activity or sex* behaviour))

#47 #8 or #9 or #10 or #11 or #12 or #13 or #14 or #15 or #16 or #17 or #18 or #19 or #20 or #21 or #22 or #23 or #24 or #25 or #26 or #27 or #28 or #29 or #30 or #31 or #32 or #33 or #34 or #35 or #36 or #37 or #38 or #39 or #40 or #41 or #42 or #43 or #44 or #45 or #46

#48 #7 and #47

## Data and analyses

**Comparison 1:** Primary clinical outcomes

Outcome or subgroup title	No. of studies	No. of participants	Statistical method	Effect size
1.1 Invasive cervical cancer (cohort studies; long‐term)	5		Risk Ratio (IV, Random, 95% CI)	0.37 [0.25, 0.56]
1.2 Invasive cervical cancer (cohort studies; long‐term; ≤ 16 years at vaccination)	3		Risk Ratio (IV, Random, 95% CI)	0.20 [0.09, 0.44]
1.3 Invasive cervical cancer (RCT extension studies; medium/long‐term)	3		Risk Ratio (IV, Random, 95% CI)	Subtotals only
1.3.1 Medium‐term	1		Risk Ratio (IV, Random, 95% CI)	0.15 [0.01, 2.36]
1.3.2 Long‐term	2		Risk Ratio (IV, Random, 95% CI)	0.15 [0.02, 1.17]
1.4 CIN3+ (cohort studies; medium/long‐term)	8		Risk Ratio (IV, Random, 95% CI)	Subtotals only
1.4.1 Medium‐term	1		Risk Ratio (IV, Random, 95% CI)	0.43 [0.35, 0.53]
1.4.2 Long‐term	7		Risk Ratio (IV, Random, 95% CI)	0.39 [0.32, 0.48]
1.5 CIN3+ (cohort studies; medium/long‐term; ≤ 16 years at vaccination)	3		Risk Ratio (IV, Random, 95% CI)	Subtotals only
1.5.1 Medium‐term	1		Risk Ratio (IV, Random, 95% CI)	0.43 [0.35, 0.53]
1.5.2 Long‐term	2		Risk Ratio (IV, Random, 95% CI)	0.26 [0.12, 0.56]
1.6 CIN3 (cohort studies; long‐term)	1		Risk Ratio (IV, Random, 95% CI)	0.17 [0.06, 0.45]
1.7 CIN3 (cohort studies; long‐term; ≤ 16 years at vaccination)	1		Risk Ratio (IV, Random, 95% CI)	0.09 [0.01, 0.70]
1.8 CIN2+ (cohort studies; medium/long‐term)	9		Risk Ratio (IV, Random, 95% CI)	Subtotals only
1.8.1 Medium‐term	3		Risk Ratio (IV, Random, 95% CI)	0.62 [0.45, 0.85]
1.8.2 Long‐term	6		Risk Ratio (IV, Random, 95% CI)	0.51 [0.41, 0.64]
1.9 CIN2+ (cohort studies; medium/long‐term; ≤ 16 years at vaccination)	7		Risk Ratio (IV, Random, 95% CI)	Subtotals only
1.9.1 Medium‐term	2		Risk Ratio (IV, Random, 95% CI)	0.59 [0.54, 0.65]
1.9.2 Long‐term	5		Risk Ratio (IV, Random, 95% CI)	0.38 [0.31, 0.45]
1.10 CIN2+ (cross‐sectional studies; medium/long‐term)	4		Risk Ratio (IV, Random, 95% CI)	Subtotals only
1.10.1 Medium‐term	3		Risk Ratio (IV, Random, 95% CI)	0.62 [0.28, 1.34]
1.10.2 Long‐term	1		Risk Ratio (IV, Random, 95% CI)	0.46 [0.21, 1.00]

**Comparison 2:** Specific adverse events

Outcome or subgroup title	No. of studies	No. of participants	Statistical method	Effect size
2.1 Postural orthostatic tachycardia syndrome (cohort studies)	2		Risk Ratio (IV, Random, 95% CI)	Subtotals only
2.1.1 Short‐term	2		Risk Ratio (IV, Random, 95% CI)	0.87 [0.34, 2.22]
2.1.2 Medium‐term	1		Risk Ratio (IV, Random, 95% CI)	0.99 [0.46, 2.12]
2.2 Chronic fatigue syndrome/myalgic encephalomyelitis (cohort studies; short/medium‐term)	4		Risk Ratio (IV, Random, 95% CI)	Subtotals only
2.2.1 Short‐term	3		Risk Ratio (IV, Random, 95% CI)	0.40 [0.22, 0.75]
2.2.2 Medium‐term	3		Risk Ratio (IV, Random, 95% CI)	0.96 [0.67, 1.39]
2.3 Chronic fatigue syndrome/myalgic encephalomyelitis (self‐controlled case series; medium‐term)	3		Risk Ratio (IV, Random, 95% CI)	0.74 [0.40, 1.39]
2.4 Paralysis (cohort studies; short/medium/long‐term)	5		Risk Ratio (IV, Random, 95% CI)	Subtotals only
2.4.1 Short‐term	4		Risk Ratio (IV, Random, 95% CI)	0.54 [0.39, 0.74]
2.4.2 Medium‐term	3		Risk Ratio (IV, Random, 95% CI)	0.61 [0.39, 0.96]
2.4.3 Long‐term	2		Risk Ratio (IV, Random, 95% CI)	0.62 [0.36, 1.07]
2.5 Complex regional pain syndrome (cohort studies; immediate/short/medium/long‐term)	3		Risk Ratio (IV, Random, 95% CI)	Subtotals only
2.5.1 Immediate‐term	1		Risk Ratio (IV, Random, 95% CI)	0.90 [0.46, 1.75]
2.5.2 Short‐term	2		Risk Ratio (IV, Random, 95% CI)	0.95 [0.46, 1.96]
2.5.3 Medium‐term	2		Risk Ratio (IV, Random, 95% CI)	0.43 [0.18, 1.03]
2.5.4 Long‐term	1		Risk Ratio (IV, Random, 95% CI)	0.76 [0.62, 0.94]
2.6 Guillain‐Barré syndrome (cohort studies; short/medium/long‐term)	6		Risk Ratio (IV, Random, 95% CI)	Subtotals only
2.6.1 Short‐term	4		Risk Ratio (IV, Random, 95% CI)	0.78 [0.10, 6.03]
2.6.2 Medium‐term	4		Risk Ratio (IV, Random, 95% CI)	1.56 [0.40, 5.99]
2.6.3 Long‐term	2		Risk Ratio (IV, Random, 95% CI)	0.89 [0.36, 2.20]
2.7 Guillain‐Barré syndrome (self‐controlled case series)	3		Risk Ratio (IV, Random, 95% CI)	Subtotals only
2.7.1 Immediate‐term	2		Risk Ratio (IV, Random, 95% CI)	1.98 [0.55, 7.12]
2.7.2 Short‐term	3		Risk Ratio (IV, Random, 95% CI)	1.53 [0.78, 2.98]
2.8 Premature ovarian failure (cohort studies; short/medium/long‐term)	3		Risk Ratio (IV, Random, 95% CI)	Subtotals only
2.8.1 Short‐term	2		Risk Ratio (IV, Random, 95% CI)	0.21 [0.03, 1.28]
2.8.2 Medium‐term	1		Risk Ratio (IV, Random, 95% CI)	0.91 [0.55, 1.51]
2.8.3 Long‐term	1		Risk Ratio (IV, Random, 95% CI)	0.96 [0.55, 1.68]

**Comparison 3:** Secondary clinical outcomes

Outcome or subgroup title	No. of studies	No. of participants	Statistical method	Effect size
3.1 Cervical screening attendance (cohort studies; long‐term)	2		Risk Ratio (IV, Random, 95% CI)	1.60 [1.57, 1.62]
3.2 Anogenital warts (cohort studies; medium/long‐term)	15		Risk Ratio (IV, Random, 95% CI)	Subtotals only
3.2.1 Medium‐term	4		Risk Ratio (IV, Random, 95% CI)	0.53 [0.37, 0.77]
3.2.2 Long‐term	13		Risk Ratio (IV, Random, 95% CI)	0.47 [0.36, 0.61]
3.3 Anogenital warts (cohort studies; medium/long‐term; ≤ 16 years at vaccination)	8		Risk Ratio (IV, Random, 95% CI)	Subtotals only
3.3.1 Medium‐term	3		Risk Ratio (IV, Random, 95% CI)	0.60 [0.30, 1.21]
3.3.2 Long‐term	6		Risk Ratio (IV, Random, 95% CI)	0.30 [0.20, 0.43]

## Characteristics of studies

### Characteristics of included studies [ordered by study ID]

**Table:** Abel 2021‐USA

***Study characteristics***		
Methods	Cohort study USA; 2009‐2016 V: self‐report O: oral rinse samples	
Participants	N = 5798 females 18 to 36 years	
Interventions	Gardasil (Merck quadrivalent)	
Outcomes	Prevalent HPV infection Follow‐up: cross‐sectional, repeated over 8 years	
Notes	Source of funding: public/non‐profit: Denise Cobb Hale; The Fisher Family Fund Conflicts of interest: some authors received funding from the vaccine developer

**Table:** Ahrlund‐Richter 2019‐SWE

***Study characteristics***		
Methods	Cohort study Sweden; 2008‐2018 V: youth clinic O: youth clinic	
Participants	N = 1274 females 15 to 23 years	
Interventions	Gardasil (Merck quadrivalent)	
Outcomes	Prevalent HPV infection Follow‐up: not reported	
Notes	Source of funding: both public/non‐profit and private/industry sources: Ferring Pharmaceuticals, the Swedish Foundation for Strategic Research (SSF), ÅkeWibergs Foundation, Jeanssons Foundation, The Stockholm Cancer Foundation, The Swedish Cancer Foundation, The Swedish Cancer and Allergy Foundation, Fredrik and Ingrid Thurings Foundation, The Foundations Längmanska Kulturfonden, Lars Hiertas Minne, Clas Groschinskys Minnesfond, Föreningen för Klinisk Mikrobiologi, Svenska Läkaresällskapet, The Stockholm City Council, Karolinska Institutet, and Grigore T Conflicts of interest: no

**Table:** Ali 2013‐AUS

***Study characteristics***		
Methods	Pre‐ vs post‐vaccine introduction Australia; 2000‐2011 V: no individual vaccination status O: Medicare, the universal health insurance scheme of Australia (insurance database)	
Participants	N = 6950 GW cases/national population females and males 15 to 44 years	
Interventions	Gardasil (Merck quadrivalent)	
Outcomes	Anogenital warts Follow‐up: 12 years	
Notes	Source of funding: private/industry: CSL Biotherapies Conflicts of interest: authors include stockholders of the vaccine developer

**Table:** Andrews 2017‐GBR

***Study characteristics***		
Methods	Self‐controlled case series United Kingdom (UK); September 2007‐March 2016 V: GP records/community child health information system (CHIS) (national database) O: Hospital Episode Statistics (HES) database (national database)	
Participants	N = 100 females 11 to 19 years	
Interventions	Cervarix (GSK bivalent), Gardasil (Merck quadrivalent)	
Outcomes	Guillain‐Barré syndrome (GBS) Follow‐up: 10 years	
Notes	Source of funding: public/non‐profit: Public Health England Conflicts of interest: no

**Table:** Arnheim‐Dahlström 2013‐DNK/SWE

***Study characteristics***		
Methods	Cohort study Denmark, Sweden: October 2006 to December 2010 V: Denmark: the childhood vaccination database at Statens Serum Institut + national prescription register; Sweden: Svevac (national HPV vaccination register, established in 2006 and held by the Swedish Institute for Communicable Disease) and the drug prescription register held by the National Board of Health and Welfare (national databases) O: national patient registers in both countries using ICD‐10 codes (national databases)	
Participants	N = 997585 females 12 to 17 years	
Interventions	Gardasil (Merck quadrivalent)	
Outcomes	Paralysis; Guillain‐Barré syndrome (GBS) Follow‐up: 6 months	
Notes	Source of funding:public/non‐profit: the Swedish Foundation for Strategic Research and the Danish Medical Research Council Conflicts of interest: some authors received funding from the vaccine developer

**Table:** Ba 2021‐USA

***Study characteristics***		
Methods	Cohort study USA; January 2006 to December 2016 V: claims of HPV vaccine using current procedural terminology (CPT) codes O: ICD‐9 and ICD‐10 codes for cervical screening	
Participants	N = 954910 females 21 to 26 years	
Interventions	Cervarix (GSK bivalent), Gardasil (Merck quadrivalent), Gardasil 9 (Merck nonavalent)	
Outcomes	Participation rates in screening Follow‐up: at least 30 days after the index date	
Notes	Funding: no specific funding Conflicts of interest: no

**Table:** Baandrup 2021‐DNK

***Study characteristics***		
Methods	Cohort study Denmark; 2006‐2016 V: Danish National Health Service Register; Danish National Prescription Registry (national database) O: Danish National Prescription Registry; Danish National Patient Register (national database)	
Participants	N = 1,076,945 females 12 to 31 years	
Interventions	Gardasil (Merck quadrivalent)	
Outcomes	Anogenital warts Follow‐up: up to 10 years	
Notes	Source of funding: public/non‐profit: the Mermaid project Conflicts of interest: some authors received funding from the vaccine developer

**Table:** Baandrup 2024‐DNK

***Study characteristics***		
Methods	Retrospective cohort study Denmark; October 2006‐December 2021 V: National Health Service register and National Prescription registry (national database) O: Danish pathology registry (national database)	
Participants	N = 926881 females 17 to 32 years	
Interventions	Cervarix (GSK bivalent), Gardasil (Merck quadrivalent), Gardasil 9 (Merck nonavalent)	
Outcomes	Anal intraepithelial neoplasia (AIN) Follow‐up: 15 years	
Notes	Funding: public/non‐profit: the Mermaid project (MERMAID II) Conflict of interest: none

**Table:** Badre‐Esfahani 2019‐DNK

***Study characteristics***		
Methods	Cohort study Denmark; October 2008‐December 2017 V: Danish National Health Service Register O: Danish Pathology Register (data on participation in the Danish National Cervical Cancer Screening Programme)	
Participants	N = 24828 females 22 to 24 years	
Interventions	Not reported	
Outcomes	Participation rates in screening Follow‐up: 18 months (after 22.5 years of age)	
Notes	Source of funding: public/non‐profit: Family Hede Nielsen’s Foundation and Helsefonden Conflicts of interest: an author received a speaker's fee from the vaccine developers

**Table:** Baldur‐Felskov 2014‐DNK

***Study characteristics***		
Methods	Pre‐ vs post‐vaccine introduction Denmark; January 2000‐March 2013 V: individual vaccination status not used ‐ pre‐/post‐introduction populations O: nationwide Pathology Data Bank (national database)	
Participants	N > 2,500,000 females > 12 years	
Interventions	Gardasil (Merck quadrivalent)	
Outcomes	CIN2+; participation rates in screening Follow‐up: 12 years	
Notes	Source of funding:public/non‐profit: the Mermaid project (MERMAID II) Conflicts of interest: some authors received funding from the vaccine developer

**Table:** Baldur‐Felskov 2015‐DNK

***Study characteristics***		
Methods	Pre‐ vs post‐vaccine introduction Denmark; 1997‐2019 V: not reported O: Danish Cancer Registry (national database)	
Participants	N = 5927 females 12 to 99 years	
Interventions	Gardasil (Merck quadrivalent)	
Outcomes	Cervical cancer; adenocarcinoma in situ; CIN3 Follow‐up: not reported	
Notes	Source of funding:public/non‐profit: the Mermaid project (MERMAID II) Conflicts of interest: some authors received funding from the vaccine developer

**Table:** Balgovind 2024‐AUS

***Study characteristics***		
Methods	Cross‐sectional study Australia; January 2015‐November 2018 V: National HPV Vaccination Program Register; self‐report O: HPV DNA detection and genotyping	
Participants	N = 1625 males 18 to 35 years	
Interventions	Gardasil (Merck quadrivalent)	
Outcomes	Prevalent HPV infection Follow‐up: cross‐sectional, up to 8 years since vaccination	
Notes	Source of funding: public/non‐profit: Commonwealth Department of Health HPV Surveillance Fund Conflicts of interest: some authors received funding from the vaccine developer

**Table:** Baril 2015‐GBR

***Study characteristics***		
Methods	Cohort study United Kingdom (UK); September 2008‐June 2011 V: Clinical Practice Research Datalink (national database) O: Clinical Practice Research Datalink (national database)	
Participants	N = 962 females 14 to 23 years	
Interventions	Cervarix (GSK bivalent)	
Outcomes	Birth outcomes Follow‐up 3 years	
Notes	Source of funding:private/industry: GlaxoSmithKline Biologicals SA Conflicts of interest: authors include employees of the vaccine developer

**Table:** Batmunkh 2019‐MNG

***Study characteristics***		
Methods	Cohort study Mongolia; August 2017‐January 2018 V: participants vaccinated in the preceding original trial O: self‐administered vaginal swabs were analysed	
Participants	1587 trial participants, community controls, females 18 to 23 years	
Interventions	Gardasil (Merck quadrivalent)	
Outcomes	Prevalent HPV infection Follow‐up: cross‐sectional	
Notes	Source of funding: public/non‐profit: Australian Department of Foreign Affairs and Trade Direct Aid Program; Murdoch Children's Research Institute and the World Health Organization, Mongolia office; Bill & Melinda Gates Foundation Conflicts of interest: none

**Table:** Batmunkh 2020‐MNG

***Study characteristics***		
Methods	Cohort study Mongolia; September 2018‐February 2019 V: immunisation records at the National Center for Communicable Diseases, Ulaanbaatar O: self‐administered vaginal swab for HPV detection and to complete a short questionnaire	
Participants	N = 475 females 16 to 26 years	
Interventions	Gardasil (Merck quadrivalent)	
Outcomes	Prevalent HPV infection Follow‐up: 6 years	
Notes	Source of funding: public/non‐profit: Bill & Melinda Gates Foundation Conflicts of interest: no

**Table:** Bauer 2012‐USA

***Study characteristics***		
Methods	Pre‐ vs post‐vaccine introduction USA; 2007‐2010 V: no individual vaccination status O: clinical encounter claims data from the California Family Planning Access Care and Treatment (Family PACT) program (insurance database)	
Participants	N = n/a (different total by year) females and males < 21 to ≥ 31 years	
Interventions	Gardasil (Merck quadrivalent)	
Outcomes	Anogenital warts Follow‐up: not reported	
Notes	Source of funding: public/non‐profit: the Centers for Disease Control and Prevention and the California Department of Public Health, Office of Family Planning Conflicts of interest: no information

**Table:** Baussano 2020‐BTN

***Study characteristics***		
Methods	Cohort study Bhutan; 2011‐2018 V: self‐report O: cervical cell collection, DNA extraction, and HPV testing and genotyping	
Participants	N = 3040 females 17 to 29 years	
Interventions	Gardasil (Merck quadrivalent)	
Outcomes	Prevalent HPV infection Follow‐up: cross‐sectional, follow‐up potentially to 7 years after vaccination	
Notes	Source of funding: public/non‐profit: Bill & Melinda Gates Foundation Conflicts of interest: unclear

**Table:** Baussano 2021‐RWA/BTN

***Study characteristics***		
Methods	Cohort study Bhutan and Rwanda; 2013‐2017 V: self‐report O: urine collection and DNA extraction	
Participants	N = 3881 females 17 to 22 years	
Interventions	Gardasil (Merck quadrivalent)	
Outcomes	Prevalent HPV infection Follow‐up: cross‐sectional	
Notes	Source of funding: public/non‐profit: Bill and Melinda Gates Foundation Conflicts of interest: some authors hold shares in companies with related interests

**Table:** Bednarczyk 2012‐USA

***Study characteristics***		
Methods	Cohort study USA; July 2006‐December 2010 V: Kaiser Permanente Georgia clinical (routine administrative database, insurance) O: Kaiser Permanente Georgia clinical (routine administrative database, insurance)	
Participants	N = 1398 females 14 to 16 years	
Interventions	Gardasil (Merck quadrivalent)	
Outcomes	Sexual activity (incidence of sexually transmitted infections) Follow‐up: up to 3 years	
Notes	Funding: no specific funding Conflicts of interest: some authors received funding from the vaccine developer

**Table:** Benard 2017‐USA

***Study characteristics***		
Methods	Pre‐ vs post‐vaccine introduction USA; January 2007‐December 2020 V: no individual vaccination status reported or used ‐ pre‐/post‐vaccine analysis O: the New Mexico HPV Pap Registry (NMHPVPR) (national database)	
Participants	N = 219797 females attending cervical screening 15 to 29 years	
Interventions	Gardasil (Merck quadrivalent)	
Outcomes	CIN3; CIN2 Follow‐up: cross‐sectional, repeated over 14 years	
Notes	Source of funding: public/non‐profit: National Institute of Allergy and Infectious Diseases Conflicts of interest: some authors received funding from the vaccine developer

**Table:** Berenson 2021‐USA

***Study characteristics***		
Methods	Cross‐sectional study USA; 2011‐2016 V: self‐report O: oral sample	
Participants	N = 9437 females and males 18 to 59 years	
Interventions	Gardasil (Merck quadrivalent)	
Outcomes	Prevalent HPV infection Follow‐up: cross‐sectional	
Notes	Source of funding: public/non‐profit: Cancer Prevention and Research Institute of Texas Conflicts of interest: no

**Table:** Bertoli 2020‐DNK

***Study characteristics***		
Methods	Pre‐ vs post‐vaccine introduction Denmark; January 1978‐December 2017 V: no individual vaccination status reported or used ‐ pre‐/post‐vaccine analysis O: The Danish Pathology Register and the Danish Cancer Registry (national database)	
Participants	N = not reported, females All ages	
Interventions	Not reported	
Outcomes	Vaginal cancer Follow‐up: 39 years	
Notes	Source of funding: not reported Conflicts of interest: some authors received funding from the vaccine developer

**Table:** Bobadilla 2024‐PAR

***Study characteristics***		
Methods	Cross‐sectional study Paraguay; May 2020‐September 2023 V: self‐report O: Central Laboratory of Public Health	
Participants	N = 254 females 18 to 25 years	
Interventions	Gardasil (Merck quadrivalent)	
Outcomes	Prevalent HPV infection Follow‐up: cross‐sectional	
Notes	Funding: National Council of Science and Technology Conflicts of interest: none

**Table:** Bogaards 2019‐NLD

***Study characteristics***		
Methods	Cross‐sectional study Netherlands; 2011‐2017 V: self‐report O: vaginal swab	
Participants	N = 2104 females 16 to 24 years	
Interventions	Cervarix (GSK bivalent)	
Outcomes	Prevalent HPV infection Follow‐up: cross‐sectional, up to 8 years after vaccination	
Notes	Source of funding: public/non‐profit: Dutch Ministry of Health, Welfare and Sport; Strategic Programme from the National Institute for Public Health and the Environment Conflicts of interest: no

**Table:** Boone 2016‐USA

***Study characteristics***		
Methods	Cohort study USA; July 2006‐July 2013 V: vaccination status was ascertained from patient logs maintained for vaccine accountability, billing records and EMR searches for quadrivalent HPV vaccine, HPV4 or Gardasil O: electronic medical records	
Participants	N = 2246 females 14 to 26 years	
Interventions	Gardasil (Merck quadrivalent)	
Outcomes	Participation rates in screening Follow‐up: 7 years	
Notes	Funding: no specific funding Conflicts of interest: none

**Table:** Brotherton 2019‐AUS

***Study characteristics***		
Methods	Cohort study; retrospective cohort study/database linkage Australia; 1 January 2000–31 December 2014 V: National HPV Vaccination Program Register (NHVPR) (national database) O: National Cervical Screening Program (national database)	
Participants	N = 250,648 females 15 to 22 years	
Interventions	Gardasil (Merck quadrivalent)	
Outcomes	CIN3+; CIN2+ Follow‐up: median follow‐up time of 1.7 years (IQR 0.8 to 2.5 years)	
Notes	Source of funding: public/non‐profit: Australian Department of Health; National Health and Medical Research Council; Centre for Research Excellence in Cervical Cancer Control Conflicts of interest: none

**Table:** Bukowinski 2020‐USA

***Study characteristics***		
Methods	Cohort study USA; 2007‐2014 V: Defense Manpower Data Center (national database) O: inpatient/outpatient records (study‐level targeted ascertainment)	
Participants	N = 906,000 females 17 to 28 years	
Interventions	Gardasil	
Outcomes	Birth outcomes Follow‐up: not reported	
Notes	Source of funding:public/non‐profit: Defense Health Agency Immunization Healthcare Division and US Navy Bureau of Medicine and Surgery Conflicts of interest: none

**Table:** Cameron 2016‐GBR

***Study characteristics***		
Methods	Pre‐ vs post‐ vaccine introduction United Kingdom (UK); 2004‐2014 V: no individual vaccine status reported O: hospital discharge data, generated by Scottish National Health Service (national database)	
Participants	N = not reported, females and males 12 to 18 years	
Interventions	Cervarix (GSK bivalent), Gardasil (Merck quadrivalent)	
Outcomes	Chronic fatigue syndrome/myalgic encephalomyelitis (CFS/ME); Guillain‐Barré syndrome (GBS) Follow‐up: not reported	
Notes	Funding: no specific funding Conflicts of interest: none

**Table:** Canvin 2017‐GBR

***Study characteristics***		
Methods	Pre‐ vs post‐vaccine introduction United Kingdom (UK); 2009‐2014 V: no individual vaccination status (coverage estimates from published reports) O: GUM Clinic Activity Dataset (GUMCADv2) submitted by GUM and integrated GUM/sexual and reproductive health clinics (national database)	
Participants	N = not reported, females and males (attending sexual health clinic) 15 to 24 years	
Interventions	Cervarix (GSK bivalent), Gardasil (Merck quadrivalent)	
Outcomes	Anogenital warts Follow‐up: repeated cross‐sectional 2009‐2014	
Notes	Source of funding: public/non‐profit: Public Health England Conflicts of interest: no

**Table:** Carnalla 2021‐MEX

***Study characteristics***		
Methods	Cohort study Mexico 2017‐2019 V: participants vaccinated in the preceding original trial O: lab test HPV DNA in urine was determined with the commercial kit BD OnclarityTM HPV Assay	
Participants	N = 232 females 17 to 19 years	
Interventions	Cervarix (GSK bivalent), Gardasil (Merck quadrivalent)	
Outcomes	Prevalent HPV infection Follow‐up: cross‐sectional	
Notes	Funding: no specific funding Conflicts of interest: none

**Table:** Carozzi 2018‐ITA

***Study characteristics***		
Methods	Cohort study Italy; May 2012 to February 2014 V: official computerised HPV vaccine registry of the LHU of Matera O: at the enrolment visit, two cervical samples (one each for Pap and HPV testing) were obtained, and participants completed self‐administered sociodemographic and behavioural questionnaires	
Participants	N = 2804 females 24 to 50 years	
Interventions	Gardasil (Merck quadrivalent)	
Outcomes	Prevalent HPV infection Follow‐up: not reported	
Notes	Source of funding: private/industry: Sanofi‐Pasteur Conflicts of interest: authors include employees of the vaccine developer

**Table:** Castle 2019‐USA

***Study characteristics***		
Methods	Cohort study USA; December 2006‐May 2017 V: Kaiser Permanente Northern California (insurance database) O: Kaiser Permanente Northern California (insurance database)	
Participants	N = 75,649 females attending cervical screening 21 to 24 years	
Interventions	Gardasil (Merck quadrivalent)	
Outcomes	CIN3+; CIN2+ Follow‐up: 3 years	
Notes	Source of funding: not reported Conflicts of interest: some authors received funding from the vaccine developer

**Table:** Chambers 2022‐CAN

***Study characteristics***		
Methods	Cross‐sectional study Canada; February 2017‐August 2019 V: self‐report O: anal specimens were self‐collected at study sites using moistened Dacron swabs	
Participants	N = 645 males 16 to 30 years	
Interventions	Gardasil (Merck quadrivalent), Gardasil 9 (Merck nonavalent)	
Outcomes	Prevalent HPV infection Follow‐up: cross‐sectional	
Notes	Source of funding: public/non‐profit: Canadian Institutes of Health Research, the CIHR Canadian HIV/AIDS Trials Network, the Canadian Association for HIV/AIDS Research, the Ontario HIV Treatment Network, the Public Health Agency of Canada, Ryerson University, the Canadian Immunization Research Network. The HIV/AIDS network of Fonds de Recherche du Québec – Santé supported quality assurance and control of human papillomavirus testing. Conflicts of interest: some authors received funding from the vaccine developer

**Table:** Cho 2024‐KOR

***Study characteristics***		
Methods	Retrospective cohort study/database linkage Korea; July 2011‐December 2021 V: Immunization Registry Integration System O: National Health Information Database	
Participants	N = 332,062 females 12 to 13 years	
Interventions	Gardasil (Merck quadrivalent); Gardasil 9 (Merck nonavalent)	
Outcomes	Anogenital warts	
Notes	Funding source: none Conflicts of interest: none

**Table:** Chow 2017‐AUS

***Study characteristics***		
Methods	Pre‐ vs post‐vaccine introduction Australia; July 2004‐June 2015 V: no individual vaccination status O: stored chlamydia‐positive, urine and urethral swab specimens	
Participants	N = 1466 males ≤ 25 years	
Interventions	Gardasil (Merck quadrivalent)	
Outcomes	Prevalent HPV infection Follow‐up: 11 years	
Notes	Source of funding: public/non‐profit: The Australian National Health and Medical Research Council Program Conflicts of interest: authors include stockholders of the vaccine developer

**Table:** Chow 2019‐AUS

***Study characteristics***		
Methods	Pre‐ vs post‐ vaccine introduction Australia; 2014‐2017 V: self‐reported vaccine doses were confirmed with doses reported to the National HPV Vaccination Program Register O: males provided a self‐collected penile swab for 37 HPV genotypes using Roche Linear Array and completed a questionnaire (study‐level targeted ascertainment)	
Participants	N = 298 males (attending sexual health clinic) 17 to 19 years	
Interventions	Gardasil (Merck quadrivalent)	
Outcomes	Anogenital warts; prevalent HPV infection Follow‐up: cross‐sectional, 2 years after possible vaccination	
Notes	Source of funding: both public/non‐profit and private/industry sources: Merck & Co.; Australian Government Department of Health Conflicts of interest: authors include stockholders of the vaccine developer

**Table:** Chow 2021a‐AUS

***Study characteristics***		
Methods	Pre‐ vs post‐vaccine introduction Australia; October 2010‐December 2018 V: National HPV Vaccination Program Register (before October 2018) or the Australian Immunisation Register (after October 2018) O: participants provided three specimens for HPV genotyping: an anal swab, a penile swab and an oral rinse	
Participants	N = 400 males 16 to 20 years	
Interventions	Gardasil (Merck quadrivalent)	
Outcomes	Prevalent HPV infection Follow‐up: cross‐sectional (repeated)	
Notes	Source of funding: both public/non‐profit and private/industry sources: Merck; Australian Government Department of Health Conflicts of interest: authors include stockholders of the vaccine developer

**Table:** Chow 2021b‐AUS

***Study characteristics***		
Methods	Pre‐ vs post‐vaccine introduction Australia; January 2004‐December 2018 V: no individual vaccination status O: clinical diagnosis of genital warts for all new patients who attended the GWSN sexual health clinics (study‐level targeted ascertainment)	
Participants	N = 237,379 females and males (attending sexual health clinic) ≥ 15 years	
Interventions	Gardasil (Merck quadrivalent)	
Outcomes	Anogenital warts Follow‐up: cross‐sectional, repeated over 15 years	
Notes	Source of funding: both public/non‐profit and private/industry sources: Seqirus Australia; Australian Government Department of Health Conflicts of interest: some authors received funding from the vaccine developer

**Table:** Clark 2021‐CAN

***Study characteristics***		
Methods	Pre‐ vs post‐vaccine introduction Canada; January 2003‐December 2018 V: not reported O: Cytobase, database of patient medical records of Pap tests performed in Ontario	
Participants	N = 221,039 females 18 to 23 years	
Interventions	Gardasil (Merck quadrivalent)	
Outcomes	Treatment rates for CIN and other HPV‐related disease Follow‐up: 5 years	
Notes	Funding: no specific funding Conflicts of interest: none

**Table:** Closson 2020‐USA

***Study characteristics***		
Methods	Cross‐sectional study USA; 2013‐2016 V: self‐report (National Health and Nutrition Examination Survey) O: self‐collected urine and cervicovaginal samples	
Participants	N = 1050 females 18 to 35 years	
Interventions	Gardasil (Merck quadrivalent)	
Outcomes	Prevalent HPV infection Follow‐up: cross‐sectional	
Notes	Funding: no specific funding Conflicts of interest: some authors received funding from the vaccine developer

**Table:** Cocchio 2017‐ITA

***Study characteristics***		
Methods	Pre‐ vs post‐vaccine introduction Italy; 2004‐2015 V: no individual vaccination status O: hospital discharge records (hospital database)	
Participants	N = 6076 females and males 12 to 48+ years	
Interventions	Gardasil (Merck quadrivalent)	
Outcomes	Anogenital warts Follow‐up: cross‐sectional (repeated)	
Notes	Source of funding: public/non‐profit: university grant Conflicts of interest: no

**Table:** Combita 2021‐COL

***Study characteristics***		
Methods	Cross‐sectional study Colombia; May 2014‐February 2015 and January 2016‐December 2018 V: self‐administered questionnaire O: each woman underwent a gynaecologic examination, and two cervical samples (one each for Pap and HPV testing) were obtained	
Participants	N = 3273 females 18 to 25 years	
Interventions	Cross‐sectional (repeated)	
Outcomes	Prevalent HPV infection Follow‐up: cross‐sectional	
Notes	Source of funding: public/non‐profit: Colombian Health and Social Protection Ministry Conflicts of interest: none

**Table:** Crowe 2014‐AUS

***Study characteristics***		
Methods	Case‐control study Australia; April 2007 and March 2011 V: Queensland Health Vaccination Information Vaccination Administration System O: Queensland Health Pap Smear Register	
Participants	N = 108,353 females Age not reported	
Interventions	Gardasil (Merck quadrivalent)	
Outcomes	CIN2+ Follow‐up: median follow‐up time from study start date to index date 808 days (interquartile range 456 to 1131 days)	
Notes	Funding: no specific funding Conflicts of interest: not reported

**Table:** Cruickshank 2017‐GBR

***Study characteristics***		
Methods	Pre‐ vs post‐vaccine introduction United Kingdom (UK); 2008‐2014 V: individual vaccination status not used. Pre‐/post‐vaccine analysis. O: colposcopy results from National Colposcopy Clinical Information and Audit System (NCCIAS) (national database)	
Participants	N = 7013 females attending colposcopy 20 to 21 years	
Interventions	Gardasil (Merck quadrivalent)	
Outcomes	CIN2+; treatment rates for CIN and other HPV‐related disease Follow‐up: 7 repeated cross‐sectional surveys	
Notes	Source of funding:public/non‐profit: Chief Scientist Office Conflicts of interest: none

**Table:** Cummings 2012‐USA

***Study characteristics***		
Methods	Cohort study USA; 1999‐2010 V: HPV vaccination status was verified through clinical records after enrolment (post‐vaccine group) O: self‐report (questionnaire) (study‐level targeted ascertainment)	
Participants	N = 225 females 14 to 17 years	
Interventions	Gardasil (Merck quadrivalent)	
Outcomes	Sexual activity (incidence of sexually transmitted infections); prevalent HPV infection Follow‐up: cross‐sectional	
Notes	Source of funding:public/non‐profit: National Institutes of Health (NIH) Conflicts of interest: some authors received funding from the vaccine developer

**Table:** Cuschieri 2023‐GBR

***Study characteristics***		
Methods	Pre‐ vs post‐vaccine introduction United Kingdom (Scotland); 2011‐2017 V: individual vaccination status not used. Pre‐/post‐vaccine analysis. O: 10 pathology laboratories in Scotland that serve 14 NHS territorial board areas	
Participants	N = 1706 females 20 to 25 years	
Interventions	Cervarix (GSK bivalent)	
Outcomes	CIN3; CIN2+; CIN2 Follow‐up: not reported	
Notes	Funding: the Scottish Government Conflicts of interest: none

**Table:** Deceuninck 2018‐CAN

***Study characteristics***		
Methods	Cohort study Canada; October 1999‐March 2014 V: targeted for vaccination – no individual vaccination status data O: Quebec provincial hospital discharge database (national database)	
Participants	N = 13,736,169 person‐years females and males 7 to 17 years	
Interventions	Gardasil (Merck quadrivalent)	
Outcomes	Guillain‐Barré syndrome (GBS) Follow‐up: 15 years	
Notes	Source of funding:public/non‐profit: Quebec Ministry of Health and Social Services Conflicts of interest: some authors received funding from the vaccine developer

**Table:** Dehlendorff 2018‐DNK/SWE

***Study characteristics***		
Methods	Cohort study Denmark and Sweden; 2006‐2013 V: Swedish and Danish national health registry (national database) O: Swedish and Danish national health registry (national database)	
Participants	N = not reported, females 13 to 30 years	
Interventions	Gardasil (Merck quadrivalent)	
Outcomes	CIN2+ Follow‐up: 7 years	
Notes	Source of funding: public/non‐profit: Mermaid Project (Mermaid 2), the Swedish Foundation for Strategic Research, the Swedish Research Council and the Swedish Cancer Society Conflicts of interest: some authors received funding from the vaccine developer

**Table:** Delere 2014‐DEU

***Study characteristics***		
Methods	Cross‐sectional study Germany; October 2010‐September 2012 V: self‐report (questionnaire) O: self‐report; self‐sampling was performed by cervicovaginal lavage	
Participants	N = 787 females 20 to 25 years	
Interventions	Cervarix (GSK bivalent), Gardasil (Merck quadrivalent)	
Outcomes	Prevalent HPV infection Follow‐up: cross‐sectional	
Notes	Source of funding: public/non‐profit: Robert Koch Institute Conflicts of interest: some authors received expenses from the vaccine developer

**Table:** Del Mistro 2021‐ITA

***Study characteristics***		
Methods	Cohort study Italy; January 2008‐December 2019 V: LHUs’ vaccination databases (regional database) O: screening programmes (national database)	
Participants	N = 96,230 females 25 to 64 years	
Interventions	Gardasil (Merck quadrivalent)	
Outcomes	Cervical cancer; CIN3+; CIN2+; participation rates in screening Follow‐up: 12 years overall	
Notes	Source of funding: public/non‐profit: Italian Ministry of Health Conflicts of interest: no

**Table:** DeSisto 2024‐USA

***Study characteristics***		
Methods	Cross‐sectional study USA; August 2018‐July 2023 V: medical records or registry data O: self‐ or clinician‐collected anal swab samples	
Participants	N = 6350 MSM 18 to 45 years	
Interventions	Gardasil (Merck quadrivalent); Gardasil 9 (Merck nonavalent)	
Outcomes	Prevalent HPV infection Follow‐up: cross‐sectional	
Notes	Funding: Centers for Diseases Control and Prevention Conflict of interest: none

**Table:** De Souza 2023‐AUS

***Study characteristics***		
Methods	Cross‐sectional study Australia; October 2020‐November 2021 V: Australian Immunisation Register O: oral saliva samples	
Participants	N = 911 females and males 18 to 70 years	
Interventions	Gardasil (Merck quadrivalent); Gardasil 9 (Merck nonavalent)	
Outcomes	Prevalent HPV infection Follow‐up: cross‐sectional	
Notes	Source of funding: private/industry: Merck & Co Conflicts of interest: none

**Table:** Dillner 2018‐EU

***Study characteristics***		
Methods	Pre‐ vs post‐vaccine introduction Denmark, Sweden and Norway; 2006‐2013 V: no individual vaccination status O: national cervical screening registries	
Participants	N = 12870 females 18 to 50 years	
Interventions	Gardasil (Merck quadrivalent)	
Outcomes	Prevalent HPV infection Follow‐up: cross‐sectional	
Notes	Source of funding: private/industry: Merck & Co Conflicts of interest: authors include employees of the vaccine developer

**Table:** Dominiak‐Felden 2015‐BEL

***Study characteristics***		
Methods	Cohort study; pre‐ vs post‐vaccine introduction Belgium; January 2006 to December 2013 V: MLOZ database of reimbursements (insurance database) O: MLOZ database of reimbursements (insurance database)	
Participants	N = 106,579 females and males Age (median) 19.3	
Interventions	Gardasil (Merck quadrivalent)	
Outcomes	Anogenital warts Follow‐up: 6 years	
Notes	Source of funding: private/industry: Sanofi Pasteur MSD Conflicts of interest: authors include stockholders of the vaccine developer

**Table:** Donegan 2013‐GBR

***Study characteristics***		
Methods	Pre‐ vs post‐vaccine introduction; self‐controlled case series United Kingdom; October 2008‐December 2011 V: Clinical Practice Research Datalink (national database) O: Clinical Practice Research Datalink (national database)	
Participants	161 cases of CFS/ME, females 12 to 20 years	
Interventions	Cervarix (GSK bivalent)	
Outcomes	Chronic fatigue syndrome/myalgic encephalomyelitis (CFS/ME) Follow‐up: not reported	
Notes	Source of funding:not reported Conflicts of interest: not reported

**Table:** Donken 2018‐NLD

***Study characteristics***		
Methods	Cohort study Netherlands; 2009‐2016 V: national vaccination registration system, Praeventis O: self‐collected vaginal swab	
Participants	N = 1635 females 20 to 23 years	
Interventions	Cervarix (GSK bivalent)	
Outcomes	Incident HPV infection; persistent HPV infection Follow‐up: 6 years	
Notes	Source of funding: public/non‐profit: Ministry of Health, Welfare, and Sport, the Netherlands Conflicts of interest: some authors received funding from the vaccine developer

**Table:** Donken 2021‐CAN

***Study characteristics***		
Methods	Cohort; pre‐ vs post‐vaccine introduction Canada; 2004‐2017 V: individual vaccination status not used – pre‐/post‐introduction populations O: BC Cancer Cervix Screening Program Database (national database)	
Participants	N = not reported, females attending cervical screening 16 to 28 years	
Interventions	Cervarix (GSK bivalent), Gardasil (Merck quadrivalent), Gardasil 9 (Merck nonavalent)	
Outcomes	CIN3; CIN2+; CIN2 Follow‐up: not reported	
Notes	Source of funding: public/non‐profit: Canadian Immunization Research Network (CIRN), the Michael Smith Foundation for Health Research (MSFHR), Canadian Institutes of Health Research (CIHR), BC Children's Hospital Foundation, and the Canadian Child Health Clinician Scientist Program Conflicts of interest: some authors received funding from the vaccine developer

**Table:** Dorton 2015‐USA

***Study characteristics***		
Methods	Cross‐sectional study USA; February 2007 to March 2014 V: self‐report O: electronic patient registry (hospital database)	
Participants	N = 1392 females attending colposcopy ≤ 26 years	
Interventions	Gardasil (Merck quadrivalent)	
Outcomes	Cervical cancer; adenocarcinoma in situ; CIN2+ Follow‐up: cross‐sectional	
Notes	Source of funding: not reported Conflicts of interest: not reported

**Table:** Elies 2022‐FRA

***Study characteristics***		
Methods	Retrospective cohort study France; January 2006‐December 2016 V: French National Health Insurance database O: French National Health Insurance database	
Participants	N = 42,452 females 19 to 30 years	
Interventions	Cervarix (GSK bivalent); Gardasil (Merck quadrivalent)	
Outcomes	Treatment rates for CIN and other HPV‐related disease (conisation) Follow‐up: 10 years	
Notes	Funding: none reported Conflicts of interest: none reported

**Table:** Enerly 2019‐NOR

***Study characteristics***		
Methods	Cohort study Norway; September 2016‐February 2017 V: Norwegian Immunization Registry SYSVAK O: cervico‐vaginal and oral samples	
Participants	N = 312 females 18 to 20 years	
Interventions	Gardasil (Merck quadrivalent)	
Outcomes	Prevalent HPV infection Follow‐up: 6 to 8 years after vaccination	
Notes	Source of funding: public/non‐profit: Cancer Registry of Norway Conflicts of interest: some authors received funding from the vaccine developer

**Table:** Faber 2019‐DNK

***Study characteristics***		
Methods	Cohort study Denmark; October 2006‐December 2014 V: Health Service Registry and the Danish Prescription Registry (national database) O: Medical Birth Registry and the National Patient Registry (national database)	
Participants	N = 522,705 females Mean age (SD): 28 (4) years	
Interventions	Gardasil (Merck quadrivalent)	
Outcomes	Birth outcomes Follow‐up: 95 weeks	
Notes	Source of funding:public/non‐profit: Mermaid project Conflicts of interest: some authors received funding from the vaccine developer

**Table:** Falcaro 2021‐GBR

***Study characteristics***		
Methods	Cohort study United Kingdom; January 2006‐June 2019 V: no individual vaccination status. Age cohorts offered vaccination at specific ages or not offered vaccination. O: National Cancer Registration and Analysis Service, Public Health England (PHE) (national database)	
Participants	N = not reported, females 20 to 64 years	
Interventions	Gardasil (Merck quadrivalent)	
Outcomes	Cervical cancer; CIN3 Follow‐up: not reported	
Notes	Source of funding: public/non‐profit: Cancer Research UK Conflicts of interest: none

**Table:** Feder 2019‐USA

***Study characteristics***		
Methods	Cross‐sectional study USA; March 2012‐December 2014 V: self‐report O: self‐collected samples of vaginal cells	
Participants	N = 375 females 21 to 29 years	
Interventions	Not reported	
Outcomes	Prevalent HPV infection Follow‐up: cross‐sectional	
Notes	Source of funding: public/non‐profit: National Institutes of Health NIH; U.S. Department of Health and Human Services, Health Resources and Services Administration’s Maternal and Child Health Bureau Conflicts of interest: none

**Table:** Feiring 2017‐NOR

***Study characteristics***		
Methods	Cohort study Norway; 2009‐2014 V: Norwegian Immunisation Registry (national database) O: Norwegian Patient Registry (national database)	
Participants	N = 176,453 females 11 to 17 years	
Interventions	Gardasil (Merck quadrivalent)	
Outcomes	Chronic fatigue syndrome/myalgic encephalomyelitis (CFS/ME) Follow‐up: not reported	
Notes	Funding: no specific funding Conflicts of interest: none

**Table:** Fernandes 2021‐PRT

***Study characteristics***		
Methods	Pre‐ vs post‐vaccine introduction Portugal; May 2006‐December 2017 V: no individual vaccination status O: medical records of all male or female patients attending a first STD consultation (hospital database)	
Participants	N = 28,354 females (attending sexual health clinic) Age not reported	
Interventions	Gardasil (Merck quadrivalent)	
Outcomes	Anogenital warts Follow‐up: 12 years	
Notes	Source of funding: private/industry: Merck Sharp & Dohme Corp Conflicts of interest: unclear

**Table:** Flagg 2018‐USA

***Study characteristics***		
Methods	Pre‐ vs post‐vaccine introduction USA; January 2006‐December 2014 V: no individual vaccination status O: MarketScan Commercial Claims and Encounters Database (Truven Health Analytics, Ann Arbor, MI) (national database)	
Participants	N = 35,000,000 (88,911,951 person‐years) females and males 15 to 39 years	
Interventions	Gardasil (Merck quadrivalent)	
Outcomes	Anogenital warts Follow‐up: up to 9 years	
Notes	Source of funding: unclear: both authors are with the Division of STD Prevention, National Center for HIV/AIDS, Viral Hepatitis, STD, and TB Prevention, Centers for Disease Control and Prevention Conflicts of interest: not reported

**Table:** Frisch 2018‐DNK

***Study characteristics***		
Methods	Cohort study Denmark; October 2006‐November 2016 V: Danish Vaccination Register (national database) O: Danish National Patient Register (national database)	
Participants	N = 568,410 males 10 to 28 years	
Interventions	Gardasil (Merck quadrivalent)	
Outcomes	Paralysis Follow‐up: 10 years	
Notes	Source of funding:public/non‐profit: Danish Medicines Agency, Danish Cancer Society, Novo Nordisk Foundation Conflicts of interest: no

**Table:** Gargano 2021‐USA

***Study characteristics***		
Methods	Cohort study USA; 2009‐2016 V: Michigan Care Improvement Registry (regional database) O: Michigan Cancer Surveillance Program (national database)	
Participants	N = 773,193 females Age not reported	
Interventions	Gardasil (Merck quadrivalent)	
Outcomes	CIN3+ Follow‐up: 8 years	
Notes	Source of funding: public/non‐profit: Public Health Service Act and National Program of Cancer Registries Conflicts of interest: none

**Table:** Gargano 2023‐USA

***Study characteristics***		
Methods	Pre‐ vs post‐vaccine introduction USA, 2008‐2016 V: individual vaccination status not used – pre‐/post‐introduction populations O: HPV‐IMPACT study	
Participants	N = 18,344 CIN2+ cases Female 20 to 39 years	
Interventions	Gardasil (Merck quadrivalent)	
Outcomes	CIN2+; CIN3+ Follow‐up: up to 8 years	
Notes	Funding: public/non‐profit: Centers for Disease Control and Prevention Emerging Infections Program Conflict of interest: one author received funding from the vaccine developer

**Table:** Garland 2018‐AUS

***Study characteristics***		
Methods	Cohort study Australia; October 2011‐June 2015 V: self‐reported HPV vaccination details were verified with the National HPV Vaccination Program Register (NHVPR) O: self‐collected vaginal swab for HPV DNA detection and genotyping	
Participants	N = 737 females 18 to 25 years	
Interventions	Gardasil (Merck quadrivalent)	
Outcomes	Prevalent HPV infection Follow‐up: potentially to 8 years after vaccination	
Notes	Source of funding: public/non‐profit: Victorian Cancer Agency Conflicts of interest: some authors received funding from the vaccine developer

**Table:** Goggin 2018‐CAN

***Study characteristics***		
Methods	Cohort study Canada; March 2013‐July 2014 V: computer‐assisted questionnaire O: biological specimens were obtained by self‐sampling	
Participants	N = 1550 females 17 to 29 years	
Interventions	Gardasil (Merck quadrivalent)	
Outcomes	Prevalent HPV infection Follow‐up: 5–6 years after HPV vaccination	
Notes	Source of funding: public/non‐profit: Ministere de la Sante et des Services Sociaux du Quebec Conflicts of interest: some authors received funding from the vaccine developer

**Table:** Gonzalez 2020‐ARG

***Study characteristics***		
Methods	Pre‐ vs post‐vaccine introduction Argentina; 2014‐2015; 2017‐2018 V: vaccination card, electronic clinical history or self‐report. Self‐reporting was the prevalent source of information. O: cervical cell samples	
Participants	N = 2181 females 15 to 17 years	
Interventions	Cervarix (GSK bivalent)	
Outcomes	Prevalent HPV infection Follow‐up: cross‐sectional	
Notes	Source of funding: public/non‐profit: Salud Investiga (“Carrillo‐O˜nativia” and “Abraam Sonis” fellowships), Direction de Control de Enfermedades Inmunoprevenibles and Instituto Nacional de Enfermedades Infecciosas‐ ANLIS Malbran, Ministerio de Salud de la Nacion Conflicts of interest: none

**Table:** Goodman 2024‐DEU

***Study characteristics***		
Methods	Pre‐ vs post‐vaccine introduction Germany; January 2013‐December 2021 V: individual vaccination status not used – pre‐/post‐introduction populations O: Institut fur angewandte Gesundheitsforschung Berlin GmbH (InGef) research database	
Participants	N = 22,533 (pre‐vaccine cohort); 38,987 (post‐vaccine cohort) 28 to 33 years	
Interventions	Cervarix (GSK bivalent); Gardasil (Merck quadrivalent); Gardasil 9 (Merck nonavalent)	
Outcomes	CIN2+, CIN2, CIN3, anogenital warts, cervical cancer Follow‐up: 15 years	
Notes	Funding: for profit: Merck Sharp & Dohme LLC Conflicts of interest: authors are employees of vaccine manufacturer

**Table:** Grieger 2024‐DEU

***Study characteristics***		
Methods	Pre‐ vs post‐vaccine introduction Germany; 2004‐2018 V: individual vaccination status not used – pre‐/post‐introduction populations O: German Center for Cancer Registry Data	
Participants	N = 265,365 cases 18 to 35 years	
Interventions	Cervarix (GSK bivalent); Gardasil (Merck quadrivalent); Gardasil 9 (Merck nonavalent)	
Outcomes	Cervical cancer	
Notes	Funding: not reported Conflicts of interest: none

**Table:** Grimaldi‐Bensouda 2017‐FRA

***Study characteristics***		
Methods	Case‐control study France; April 2008‐October 2014 V: a tangible proof for HPV vaccination: vaccine batch number, vaccination booklet, prescription noted in health medical record, pharmacist's report, or any other type of certificate of HPV vaccination (study‐level targeted ascertainment) O: definite diagnosis of Guillain‐Barré syndrome at specialised centres across France (study‐level targeted ascertainment)	
Participants	N = 143 cases of Guillain‐Barré syndrome, females 11 to 25 years	
Interventions	Cervarix (GSK bivalent), Gardasil (Merck quadrivalent)	
Outcomes	Guillain‐Barré syndrome (GBS) Follow‐up: 6 years	
Notes	Source of funding: private/industry: GlaxoSmithKline Biologicals SA Conflicts of interest: some authors received funding from the vaccine developer

**Table:** Gronlund 2016‐SWE

***Study characteristics***		
Methods	Cohort study Sweden; October 2006‐December 2012 V: Swedish Voluntary Vaccination Register; Prescribed Drug Register (national database) O: National Patient Register (NPR) (national database)	
Participants	N = 70,265 females 10 to 30 years	
Interventions	Gardasil (Merck quadrivalent)	
Outcomes	Guillain‐Barré syndrome (GBS) Follow‐up: 6 years	
Notes	Source of funding:public/non‐profit: Swedish Foundation for Strategic Research and the Strategic Research Area in Epidemiology (SfoEpi) Conflicts of interest: some authors received funding from the vaccine developer

**Table:** Guerra 2016‐CAN

***Study characteristics***		
Methods	Pre‐ vs post‐vaccine introduction Canada; April 2003‐March 2013 V: no individual vaccination status O: Ontario Health Insurance Program (OHIP) (insurance database)	
Participants	N = not reported, females 12 to 13 years	
Interventions	Gardasil (Merck quadrivalent)	
Outcomes	Anogenital warts Follow‐up: 11 years	
Notes	Source of funding: public/non‐profit Public Health Ontario; the Institute for Clinical Evaluative Sciences, Ontario Ministry of Health and Long‐Term Care Conflicts of interest: none

**Table:** Guo 2023‐USA

***Study characteristics***		
Methods	Pre‐ vs post‐vaccine introduction USA; 2001‐2019 V: no individual vaccination status reported or used – pre‐/post‐vaccine analysis O: US Cancer Statistic Database (national database)	
Participants	N = 8062 males and females 15 to 44 years	
Interventions	Gardasil (Merck quadrivalent)	
Outcomes	Cervical cancer; anal cancer; vulvar cancer; head and neck cancer; anal cancer; vaginal cancer Follow‐up: 18 years	
Notes	Funding: The Cancer Prevention and Research Institute of Texas Conflict of interest: none

**Table:** Hariri 2018‐USA

***Study characteristics***		
Methods	Cohort study USA; August 2006‐September 2012 V: Kaiser Permanente electronic medical records (insurance database) O: Kaiser Permanente electronic medical records (insurance database)	
Participants	N = 64,517 females (128,010 person‐years) 11 to 22 years	
Interventions	Gardasil (Merck quadrivalent)	
Outcomes	Anogenital warts Follow‐up: up to 6 years	
Notes	Source of funding: public/non‐profit: Centers for Disease Control and Prevention Conflicts of interest: some authors received funding from the vaccine developer

**Table:** Harrison 2014‐AUS

***Study characteristics***		
Methods	Pre‐ vs post‐vaccine introduction Australia; July 2000‐June 2012 V: no individual vaccination status O: randomly selected general practitioners (GPs), records	
Participants	N = 1,175,879 encounters with patients, females Age not reported	
Interventions	Gardasil (Merck quadrivalent)	
Outcomes	Treatment rates for CIN and other HPV‐related disease Follow‐up: 12 years	
Notes	Source of funding: both public/non‐profit and private/industry sources: Australian Government Department of Health and Ageing, the Australian Government Department of Veterans’ Affairs, Australian Institute of Health and Welfare, National Prescribing Service, AstraZeneca Pty Ltd (Australia), Janssen‐Cilag Pty Ltd, Merck, Sharpe and Dohme (Australia) Pty Ltd, Pfizer Australia Pty Ltd, Abbott Australasia Pty Ltd, Sanofi‐Aventis Australia Pty Ltd, Wyeth Australia Pty Ltd, Novartis Pharmaceuticals Australia Pty Ltd, GlaxoSmithKline Australia Pty Ltd, Roche Products Pty Ltd, BioCSL Pty Ltd, Bayer Australia Ltd. Conflicts of interest: authors include employees of the vaccine developer

**Table:** Heard 2017‐FRA

***Study characteristics***		
Methods	Cross‐sectional study France; 6 June 2014 to 25 March 2015 V: immunisation record O: the HPV analysis was performed on the residual material that remained after DNA extraction and analysis for *C trachomatis*. All samples were anonymised.	
Participants	N = 2715 females 18 to 25 years	
Interventions	Gardasil (Merck quadrivalent)	
Outcomes	Prevalent HPV infection Follow‐up: cross‐sectional	
Notes	Source of funding: public/non‐profit: Institut National du Cancer, Ville de Paris, SPILF (Société de pathologie infectieuse de langue Francaise) Conflicts of interest: no

**Table:** Herweijer 2016‐SWE

***Study characteristics***		
Methods	Cohort study Sweden; January 2006‐December 2013 V: Swedish HPV Vaccination register; National Vaccination register; Prescribed Drug register (national databases) O: The National Swedish Cervical Screening Registry (NKCx); Swedish Cancer Register (national database)	
Participants	N = 1,333,691 females 13 to 30 years	
Interventions	Gardasil (Merck quadrivalent)	
Outcomes	CIN3+; CIN2+ Follow‐up: 8 years	
Notes	Source of funding: mixed: Merck Sharp & Dohme; GlaxoSmithKline; Swedish Foundation for Strategic Research; Strategic Research Area in Epidemiology Conflicts of interest: some authors received funding from the vaccine developer

**Table:** Herweijer 2018‐SWE

***Study characteristics***		
Methods	Cohort study; pre‐ vs post‐vaccine introduction Sweden; 2006‐2012 V: Statistics Sweden, Swedish Patient Register (national database) O: Swedish Patient Register, Prescribed Drug Register (national database)	
Participants	N = 100,000 person‐years females and males 15 to 44 years	
Interventions	Gardasil (Merck quadrivalent)	
Outcomes	Anogenital warts Follow‐up: not reported	
Notes	Source of funding: public/non‐profit: Swedish Foundation for Strategic Research Conflicts of interest: no

**Table:** Hikari 2022‐JPN

***Study characteristics***		
Methods	Cross‐sectional study Japan; April 2014‐March 2020 V: self‐report survey O: cervical cancer screening database, Saga Health Promotion Foundation	
Participants	N = 7253 females 20 to 24 years	
Interventions	Cervarix (GSK bivalent); Gardasil (Merck quadrivalent)	
Outcomes	CIN2+, CIN3+ Follow‐up: not reported	
Notes	Funding: not reported Conflicts of interest: none

**Table:** Hiramatsu 2021‐JPN

***Study characteristics***		
Methods	Cross‐sectional study Japan; April 2011 – NR V: local government database or clinical record in the clinic or hospitals (regional databases) O: screening attendance (study‐level targeted ascertainment)	
Participants	N = 1047 females attending cervical screening 20 to 21 years	
Interventions	Cervarix (GSK bivalent), Gardasil (Merck quadrivalent)	
Outcomes	CIN3; CIN2+; prevalent HPV infection Follow‐up: not reported, maximum would be 9 years (12 yo to 21 yo)	
Notes	Source of funding: private/industry: Merck Sharp and Dohme Conflicts of interest: some authors received funding from the vaccine developer

**Table:** Hirth 2017‐USA

***Study characteristics***		
Methods	Cross‐sectional study USA; 2009‐2014 V: self‐report O: oral samples	
Participants	N = 3040 females and males 18 to 30 years	
Interventions	Gardasil (Merck quadrivalent)	
Outcomes	Prevalent HPV infection Follow‐up: cross‐sectional	
Notes	Source of funding: public/non‐profit: Office of Research on Women’s Health (ORWH); Eunice Kennedy Shriver National Institute of Child Health and Human Development (NICHD) at the National Institutes of Health Conflicts of interest: not reported

**Table:** Hoes 2021‐NLD

***Study characteristics***		
Methods	Cohort study Netherlands; 2014‐2018 V: the national vaccination registry, Praeventis O: vaginal self‐sample	
Participants	N = 2027 females 16 to 17 years	
Interventions	Cervarix (GSK bivalent)	
Outcomes	Incident HPV infection Follow‐up: 4 years	
Notes	Source of funding: public/non‐profit: Dutch Ministry of Health, Welfare and Sport Conflicts of interest: no

**Table:** Howell‐Jones 2013‐GBR

***Study characteristics***		
Methods	Pre‐ vs post‐vaccine introduction United Kingdom (UK); 2002‐2011 V: no individual vaccination status. Data on 3‐dose coverage achieved by the National HPV Immunisation Programme for each academic year (September to August) and Primary Care Trust (PCT) were obtained from published reports. O: diagnoses of STIs made at GUM clinics in England are reported to Public Health England (national database)	
Participants	N = not reported, females (attending sexual health clinic) 15 to 24 years	
Interventions	Cervarix (GSK bivalent)	
Outcomes	Anogenital warts Follow‐up: repeated cross‐sectional 2002‐2011	
Notes	Source of funding: public/non‐profit: Medicines and Healthcare products Regulatory Agency (MHRA); NHS National Institute for Health Research (NIHR) Conflicts of interest: authors include stockholders of the vaccine developer

**Table:** Huyghe 2023‐BEL

***Study characteristics***		
Methods	Pre‐ vs post‐vaccine introduction Belgium; 2010‐2019 V: no individual vaccination status used O: Algemeen Medisch Labo (AML) in Antwerp	
Participants	N = 3008 females 20 to 23 years	
Interventions	Cervarix (GSK bivalent); Gardasil (Merck quadrivalent)	
Outcomes	Prevalent HPV infection Follow‐up: 9 years	
Notes	Source of funding: none Conflict of interest: none

**Table:** Hviid 2017‐DNK/SWE

***Study characteristics***		
Methods	Cohort study Denmark, Sweden; October 2006‐June 2013 V: National vaccination registers : Childhood Vaccination Database (Denmark), Swedish HPV vaccination register; national prescription registers (national databases) O: hospital patient registers (national databases)	
Participants	N = 3,126,790 females 18 to 44 years	
Interventions	Gardasil (Merck quadrivalent)	
Outcomes	Paralysis; Guillain‐Barré syndrome (GBS) Follow‐up: 7 years	
Notes	Source of funding: public/non‐profit: Novo Nordisk Foundation; SFO, Karolinska Institutet; Danish Medical Research Council Conflicts of interest: some authors received funding from the vaccine developer

**Table:** Hviid 2020‐DNK

***Study characteristics***		
Methods	Self‐controlled case series Denmark; January 2007‐December 2016 V: Danish Vaccination Register (national database) O: Danish National Patient Register (national database)	
Participants	N = 1,375,737 females; 198 cases of POTS 10 to 44 years	
Interventions	Gardasil (Merck quadrivalent)	
Outcomes	Postural orthostatic tachycardia syndrome (POTS); chronic fatigue syndrome/myalgic encephalomyelitis (CFS/ME); complex regional pain syndrome (CRPS) Follow‐up: 10 years	
Notes	Source of funding: public/non‐profit: Danish Medicines Agency; Danish Cancer Society; Novo Nordisk Foundation Conflicts of interest: no

**Table:** Hviid 2021‐DNK

***Study characteristics***		
Methods	Cohort study Denmark; October 2020‐January 2021 V: Danish vaccination register (national database) O: Danish National Patient Registry (national database)	
Participants	N = 996,300 females 11 to 34 years	
Interventions	Gardasil (Merck quadrivalent)	
Outcomes	Primary ovarian insufficiency Follow‐up: not reported	
Notes	Source of funding: private/industry: Novo Nordisk Foundation Conflicts of interest: no

**Table:** Ikeda 2021‐JPN

***Study characteristics***		
Methods	Case‐control study Japan; April 2013‐March 2017 V: municipality immunisation records (regional database) O: screening (study‐level targeted ascertainment)	
Participants	N = 12,513 females attending cervical screening 20 to 24 years	
Interventions	Cervarix (GSK bivalent), Gardasil (Merck quadrivalent)	
Outcomes	Cervical cancer; CIN3+; CIN3; CIN2+; CIN2 Follow‐up: 4 years	
Notes	Source of funding: public/non‐profit: The Ministry of Health, Labor, and Welfare, Japan; the Japan Agency for Medical Research and Development Conflicts of interest: some authors received funding from the vaccine developer

**Table:** Innes 2020‐NZL

***Study characteristics***		
Methods	Cohort study New Zealand; 2010‐2015 V: New Zealand National Immunisation Register (NIR) (national database) O: National Cervical Screening Programme (NCSP) (national database)	
Participants	N = 104,313 females 20 to 24 years	
Interventions	Gardasil (Merck quadrivalent)	
Outcomes	CIN2+ Follow‐up: up to 5 years	
Notes	Source of funding: not reported Conflicts of interest: no

**Table:** Jacot‐Guillarmod 2017‐CHE

***Study characteristics***		
Methods	Pre‐ vs post‐vaccine introduction Switzerland; 2013 V: self‐report O: self‐collected cervicovaginal sample	
Participants	N = 690 females 18 years	
Interventions	Gardasil (Merck quadrivalent)	
Outcomes	Prevalent HPV infection Follow‐up: cross‐sectional, up to 5 years after vaccination	
Notes	Source of funding: public/non‐profit: University of Lausanne ; Public Health Office of the canton of Vaud Conflicts of interest: no

**Table:** Jeannot 2018‐CHE

***Study characteristics***		
Methods	Cross‐sectional study Switzerland; January 2016 and October 2017 V: self report O: self sampling procedure	
Participants	N = 409 females 24 years	
Interventions	Gardasil (Merck quadrivalent)	
Outcomes	Prevalent HPV infection Follow‐up: cross‐sectional	
Notes	Funding source: no specific funding Conflicts of interest: no

**Table:** Jemal 2013‐USA

***Study characteristics***		
Methods	Pre‐ vs post‐vaccine introduction USA; 1975‐2009 V: National Immunization Survey‐Teen (NIS‐Teen) (survey) O: CDC’s National Program of Cancer Registries (NPCR) and/or the NCI’s Surveillance, Epidemiology, and End Results (SEER) program, CDC National Center for Health Statistics’ National Vital Statistics System (national database)	
Participants	N = not reported (population estimates as of July 1 of each year), females Age not reported	
Interventions	Gardasil (Merck quadrivalent)	
Outcomes	Cervical cancer; vaginal cancer; vulval cancer; anal cancer; penile cancer; head and neck cancer Follow‐up: not reported	
Notes	Source of funding: public/non‐profit: the American Cancer Society, the Centers for Disease Control and Prevention, the National Cancer Institute, the National Institutes of Health, and the North American Association of Central Cancer Registries Conflicts of interest: not reported

**Table:** Jena 2015‐USA

***Study characteristics***		
Methods	Cohort study USA, January 2005‐December 2010 V: data on all pharmacy and medical claims from 41 large employers across the United States (routine administrative database, insurance) O: data on all pharmacy and medical claims from 41 large employers across the United States (routine administrative database, insurance)	
Participants	N = 21610 females 12 to 18 years	
Interventions	Gardasil (Merck quadrivalent)	
Outcomes	Sexual activity (incidence of sexually transmitted infections) Follow‐up: 6 years	
Notes	Source of funding: public/non‐profit: National Institutes of Health (Early Independence Award); National Institute of Aging Conflicts of interest: no

**Table:** Judlin 2016‐FRA

***Study characteristics***		
Methods	Pre‐ vs post‐vaccine introduction France; December 2008‐March 2012 V: no individual vaccination status O: AGW cases prospectively recorded by gynaecologists (study‐level targeted ascertainment)	
Participants	N = 84818 females (attending sexual health clinic) 15 to 26 years	
Interventions	Gardasil (Merck quadrivalent)	
Outcomes	Anogenital warts Follow‐up: cross‐sectional	
Notes	Source of funding: private/industry: Sanofi Pasteur MSD Conflicts of interest: authors include employees of the vaccine developer

**Table:** Kahn 2016‐USA

***Study characteristics***		
Methods	Pre‐ vs post‐ vaccine introduction USA; 2006‐2014 V: review of electronic medical records and Ohio statewide immunisation registry data O: cervicovaginal testing for HPV	
Participants	N = 1180 females 13 to 26 years	
Interventions	Gardasil (Merck quadrivalent)	
Outcomes	Prevalent HPV infection Follow‐up: cross‐sectional, repeated at 0, 3 and 7 years	
Notes	Source of funding: public/non‐profit: National Institute of Allergy and Infectious Diseases, National Institutes of Health Conflicts of interest: some authors received funding from the vaccine developer

**Table:** Kalliala 2021‐FIN

***Study characteristics***		
Methods	RCT extension Finland; 2007‐2014 V: HPV‐040 trial records ; Finnish Medical Drug Agency O:Finnish Medical Birth Registry	
Participants	N = 27845 females 15 to 22 years	
Interventions	Cervarix (GSK bivalent)	
Outcomes	Birth outcomes Follow‐up: up to 7 years	
Notes	Source of funding: both public/non‐profit and private/industry sources: Academy of Finland; Finnish Cancer Organizations; EU FP7; IMI networks PREHDICT and CoheaHR; ADVANCE; GlaxoSmithKline Biologicals SA; Helsinki Uusimaa Hospital District, Academy of Finland; Jalmari and Rauha Ahokas Foundation; Paulo Foundation Conflicts of interest: some authors received funding from the vaccine developer

**Table:** Katz 2021‐USA

***Study characteristics***		
Methods	Cohort study USA; June 2011‐April 2020 V: medical records (hospital database) O: medical records (hospital database)	
Participants	N = 1,310,334 females and males 0 to 84 years	
Interventions	Gardasil (Merck quadrivalent), Gardasil 9 (Merck nonavalent)	
Outcomes	Head and neck cancer Follow‐up: not reported	
Notes	Source of funding: public/non‐profit: National Center for Advancing Translational Sciences of the National Institutes of Health Conflicts of interest: no

**Table:** Khoo 2022‐MYS

***Study characteristics***		
Methods	Pre‐ vs post‐vaccine introduction Malaysia; 2013‐2020 V: self‐report O: self sampling procedure	
Participants	N = 1577 females 18 to 24 and 35 to 45 years	
Interventions	Cervarix (GSK bivalent); Gardasil (Merck quadrivalent)	
Outcomes	Prevalent HPV infection Follow‐up: cross‐sectional	
Notes	Funding: private/industry: vaccine manufacturer Conflict of interest: some authors received funding from the vaccine developer

**Table:** Kitamura 2023‐JPN

***Study characteristics***		
Methods	Cross‐sectional study Japan; April 2017‐March 2020 V: self‐report O: self‐sampling procedure	
Participants	N = 2044 females 16 to 75 years	
Interventions	Cervarix (GSK bivalent); Gardasil (Merck quadrivalent); Gardasil 9 (Merck nonavalent)	
Outcomes	Prevalent HPV infection Follow‐up: cross‐sectional	
Notes	Funding: Japanese Foundation for Sexual Health Medicine Conflicts of interest: none

**Table:** Kjaer 2020‐EU

***Study characteristics***		
Methods	RCT extension Denmark, Iceland, Norway, Sweden; June 2002‐March 2017 V: vaccinated: FUTURE II trial vaccine recipient (per‐protocol population); unvaccinated population constructed from Nordic national registries and a cohort study O: national registries	
Participants	N = not reported, females 23 to 29 years	
Interventions	Gardasil (Merck quadrivalent)	
Outcomes	CIN2+ Follow‐up: 14 years	
Notes	Source of funding: private/industry: Merck Sharp & Dohme Corp Conflicts of interest: authors include employees of the vaccine developer

**Table:** Kjaer 2021‐DNK

***Study characteristics***		
Methods	Cohort study Denmark; October 2006‐December 2019 V: National Health Service register and National Prescription registry (national database) O: Danish pathology registry (national database)	
Participants	N = 867,689 females < 17, 17 to 19, 20 to 30 years	
Interventions	Cervarix (GSK bivalent), Gardasil (Merck quadrivalent), Gardasil 9 (Merck nonavalent)	
Outcomes	Cervical cancer Follow‐up: up to 13 years	
Notes	Source of funding: public/non‐profit: Mermaid project (Mermaid 2) Conflicts of interest: some authors received funding from the vaccine developer

**Table:** Kjaer 2021‐EU

***Study characteristics***		
Methods	Cohort study Denmark, Norway, Sweden; 2004‐2017 V: vaccinated participants randomised to 9‐valent vaccine in a previous RCT; unvaccinated participants are historic pre‐HPV‐vaccine controls O: national screening registries	
Participants	N = not reported, females 23 to 29 years	
Interventions	Gardasil 9 (Merck nonavalent)	
Outcomes	CIN2+ Follow‐up: 8 years	
Notes	Source of funding: private/industry: Merck Sharp & Dohme Conflicts of interest: authors include employees of the vaccine developer

**Table:** Krasnopolsky 2020‐RUS

***Study characteristics***		
Methods	Cross‐sectional study Russia; study dates: not reported V: not reported O: hospital observation (study‐level targeted ascertainment)	
Participants	N = 440 females 18 to 36 years	
Interventions	Not reported	
Outcomes	Birth outcomes; anogenital warts Follow‐up: not reported	
Notes	Source of funding: not reported Conflicts of interest: not reported

**Table:** Kreimer 2011‐CRI

***Study characteristics***		
Methods	RCT extension Costa Rica; June 2004‐August 2017 V: Costa Rica Vaccine Trial records O: Costa Rica Vaccine Trial extension (study‐level targeted ascertainment)	
Participants	N = 6563 female trial participants, community controls 26 to 38 years	
Interventions	Cervarix (GSK bivalent)	
Outcomes	Birth outcomes; CIN3+; CIN2+; incident HPV infection; prevalent HPV infection Follow‐up: 11 years	
Notes	Source of funding: mixed: public/non‐profit and private/industry: US National Cancer Institute; National Institutes of Health; GlaxoSmithKline Biologicals (GSK) Conflicts of interest: authors include stock holders of the vaccine developer

**Table:** Kudo 2019‐JPN

***Study characteristics***		
Methods	Cross‐sectional study Japan; 2014‐2016 V: municipal records archived at public health centres in addition to self‐report O: residual samples from liquid‐based cytologic analysis (SurePath BD Diagnostics, Sparks, MD) during cervical screening were collected and underwent HPV genotyping.	
Participants	N = 4553 females 20 to 26 years	
Interventions	Cervarix (GSK bivalent); Gardasil (Merck quadrivalent)	
Outcomes	Prevalent HPV infection Follow‐up: not reported	
Notes	Source of funding: public/non‐profit: Health and Labor Sciences (Ministry of Health, Labor and Welfare); Japanese Agency for Medical Research and Development (AMED) Conflicts of interest: some authors received funding from the vaccine developer

**Table:** Kumakech 2016‐UGA

***Study characteristics***		
Methods	Cross‐sectional study Uganda; July 2014‐August 2014 V: 2008 HPV vaccination register O: cervical swabs; interviewer‐administered questionnaire	
Participants	N = 488 females 15 to 24 years	
Interventions	Cervarix (GSK bivalent)	
Outcomes	Prevalent HPV infection Follow‐up: 5.5 years post vaccine	
Notes	Source of funding: public/non‐profit: Swedish International Development Cooperation Agency (SIDA) Conflicts of interest: no

**Table:** Kury 2013‐BRA

***Study characteristics***		
Methods	Pre‐ vs post‐vaccine introduction Brazil; 2007‐2012 V: no individual vaccination status O: National System of Notification (SINAN) of Brazilian Ministry of Health (national database)	
Participants	N = not reported, females < 21 years	
Interventions	Gardasil (Merck quadrivalent)	
Outcomes	Anogenital warts Follow‐up: maximum 2 years	
Notes	Source of funding: public/non‐profit: Secretariat of Health of the Municipality of Campos dos Goytacazes, Rio de Janeiro, Brazil Conflicts of interest: not reported

**Table:** Laake 2020‐NOR

***Study characteristics***		
Methods	Pre‐ vs post‐vaccine introduction Norway; 2011‐2014 V: Norwegian Immunization Registry O: urine samples	
Participants	N = 11,828 females 17 years	
Interventions	Gardasil (Merck quadrivalent)	
Outcomes	Prevalent HPV infection Follow‐up: cross‐sectional, 5 years after vaccination	
Notes	Source of funding: public/non‐profit: Norwegian Institute of Public Health and the Norwegian Ministry of Health and Care Services Conflicts of interest: no

**Table:** Latsuzbaia 2019‐LUX

***Study characteristics***		
Methods	Cross‐sectional study Luxembourg; November 2015‐December 2017 V: social security records, self‐report O: cervical samples	
Participants	N = 716 females 18 to 29 years	
Interventions	Cervarix (GSK bivalent), Gardasil (Merck quadrivalent)	
Outcomes	Prevalent HPV infection Follow‐up: cross‐sectional, longest follow‐up since vaccination 17 years	
Notes	Source of funding: public/non‐profit: Fonds National de la Recherche Luxembourg Conflicts of interest: no

**Table:** Lee 2022‐THA

***Study characteristics***		
Methods	Retrospective cohort study Thailand; November 2018‐July 2019 V: registry departments of 5 institutes/hospitals O: electronic medical records	
Participants	N = 993 20 to 45 years	
Interventions	Cervarix (GSK bivalent); Gardasil (Merck quadrivalent)	
Outcomes	Prevalent HPV infection Follow‐up: 5 years	
Notes	Source of funding: National Vaccine Institute, Ministry of Public Health, Thailand Conflicts of interest: none

**Table:** Lehtinen 2017a‐FIN

***Study characteristics***		
Methods	Cross‐sectional study Finland; 2010‐2014 V: The extracted, pseudonymised DNA samples were identified as being from an HPV‐16/18–vaccinated, a hepatitis B virus–vaccinated, or an unvaccinated participant. O: The extracted DNA from the FVU samples was analysed using a polymerase chain reaction.	
Participants	N = not reported, males 18 years	
Interventions	Not reported	
Outcomes	Prevalent HPV infection Follow‐up: cross‐sectional	
Notes	Source of funding: both public/non‐profit and private/industry sources: Academy of Finland, EU FP7, and IMI networks PREHDICT and CoheaHR, and ADVANCE Conflicts of interest: some authors received funding from the vaccine developer

**Table:** Lehtinen 2017b‐FIN

***Study characteristics***		
Methods	Cohort study Finland; May 2004‐December 2014 V: Finnish Population Register Centre (national database) O: Finnish Cancer Registry; questionnaire on life habits with special emphasis on sexual health (national database, routine administrative database, national)	
Participants	N = 18,137 female trial participants, community controls 22 to 28 years	
Interventions	Cervarix (GSK bivalent)	
Outcomes	CIN3+ Follow‐up: up to 10 years	
Notes	Source of funding: mixed: public/non‐profit and private/industry: GlaxoSmithKline Biologicals SA (Belgium), Academy of Finland, Finnish Cancer Organizations and the Swedish Cancer Society Conflicts of interest: authors include employees of the vaccine developer

**Table:** Lei 2020a‐SWE

***Study characteristics***		
Methods	Cohort study Sweden; January 2006‐December 2017 V: Swedish HPV Vaccination Register, the Prescribed Drug Register, and the National Vaccination Register (national database) O: Swedish Cancer Register (national database)	
Participants	N = 1,672,983 females 10 to 30 years	
Interventions	Cervarix (GSK bivalent), Gardasil (Merck quadrivalent)	
Outcomes	CIN3+; CIN2+ Follow‐up: up to 12 years	
Notes	Source of funding: public/non‐profit: Swedish Foundation for Strategic Research; Swedish Cancer Society; Swedish Research Council; China Scholarship Council Conflicts of interest: some authors received funding from the vaccine developer

**Table:** Lei 2020b‐SWE

***Study characteristics***		
Methods	Cohort study Sweden; 2008‐December 2017 V: Swedish HPV Vaccination Register; Prescribed Drug Register (national database) O: Swedish National Cervical Screening Registry (national database)	
Participants	N = 153,250 females attending cervical screening 10 to 30 years	
Interventions	Gardasil (Merck quadrivalent)	
Outcomes	Cervical cancer Follow‐up: cross‐sectional	
Notes	Source of funding: public/non‐profit: CoheaHr; Swedish Foundation for Strategic Research; Swedish Cancer Society; Swedish Research Council; China Scholarship Council Conflicts of interest: some authors received funding from the vaccine developer

**Table:** Liu 2014‐AUS

***Study characteristics***		
Methods	Cross‐sectional study; pre‐ vs. post‐vaccine introduction Australia; 2001‐2011 V: no individual vaccination status; eligibility for vaccine determined by date of survey and age O: self‐report by telephone survey	
Participants	N = 7225 females 18 to 39 years	
Interventions	Gardasil (Merck quadrivalent)	
Outcomes	Anogenital warts Follow‐up: 10 years	
Notes	Source of funding: public/non‐profit: Australian National Health and Medical Research Council (NHMRC); Victorian Cytology Service Conflicts of interest: authors include stockholders of the vaccine developer

**Table:** Loenenbach 2023‐DEU

***Study characteristics***		
Methods	Cross‐sectional study Germany; June 2017‐January 2018 V: self‐report O: self‐sampling kit	
Participants	N = 1226 females 20 to 25 years	
Interventions	Cervarix (GSK bivalent); Gardasil (Merck quadrivalent); Gardasil 9 (Merck nonavalent)	
Outcomes	Prevalent HPV infection Follow‐up: cross‐sectional	
Notes	Source of funding: public/non‐profit: Federal Ministry of Health of Germany (Bundesministerium für Gesundheit) Conflicts of interest: none

**Table:** Lopez 2018‐ESP

***Study characteristics***		
Methods	Pre‐ vs post‐vaccine introduction Spain; 2003‐2014 V: individual vaccination status not used – pre‐/post‐introduction O: national surveillance system for hospital data (CMBD) (national database)	
Participants	N = not reported, females All ages	
Interventions	Not reported	
Outcomes	Cervical cancer; adenocarcinoma in situ Follow‐up: 12 years	
Notes	Funding: no specific funding Conflicts of interest: some authors received funding from the vaccine developer

**Table:** Lukac 2020‐CAN

***Study characteristics***		
Methods	Pre‐ vs post‐ vaccine introduction Canada; January 2000‐December 2017 V: no individual vaccination status O: system (STI‐IS) – electronic medical record system used at STI clinics (regional database)	
Participants	N = 78,588 females and males (WSM, MSW and MSM) 1) 20 years or less; 2) 21 to 23 years; 3) 28 years or less	
Interventions	Gardasil (Merck quadrivalent)	
Outcomes	Anogenital warts Follow‐up: mean person‐year per individual (SD): 1.90 y (2.26)	
Notes	Funding: no specific funding Conflicts of interest: no

**Table:** Luostarinen 2018‐FIN

***Study characteristics***		
Methods	RCT extension Finland; June 2007‐December 2015 V: RCT records O: Finnish Cancer Registry (national database)	
Participants	N = 27,367 female trial participants, community controls Age not reported	
Interventions	Cervarix (GSK bivalent), Gardasil (Merck quadrivalent)	
Outcomes	Cervical cancer; vulvar cancer; head and neck cancer Follow‐up: 7 years	
Notes	Source of funding: mixed: public/non‐profit and private/industry: Academy of Finland, Cancer Society of Finland, GSK Biologicals SA, Nordic Cancer Union Conflicts of interest: some authors received funding from the vaccine developer

**Table:** Lurie 2017‐ISR

***Study characteristics***		
Methods	Pre‐ vs post‐vaccine introduction Israel; 2006‐2015 V: Maccabi Healthcare Services database (insurance database) O: Maccabi Healthcare Services database (insurance database)	
Participants	N = not reported, females Age not reported	
Interventions	Gardasil (Merck quadrivalent)	
Outcomes	Anogenital warts Follow‐up: not reported, study periods assumed at least 1 year lag to observe vaccine effect	
Notes	Source of funding: not reported Conflicts of interest: some authors received expenses from the vaccine developer

**Table:** Lynge 2020‐DNK

***Study characteristics***		
Methods	Pre‐ vs. post vaccine introduction Denmark; 2017‐2019 V: unclear O: HPV testing for the study embedded in routine cytology examination for cervical screening	
Participants	N = not reported, females 23 years	
Interventions	Gardasil (Merck quadrivalent)	
Outcomes	Prevalent HPV infection Follow‐up: cross‐sectional	
Notes	Source of funding: both public/non‐profit and private/industry sources: Danish Health Foundation, Det Frie Forskningsråd; HPV‐DNA test‐kits for the study were provided free of charge by Roche Conflicts of interest: no

**Table:** Ma 2017‐USA

***Study characteristics***		
Methods	Cohort study USA; October 2010‐May 2012 V: self‐report O: self‐collected vaginal samples for HPV DNA testing	
Participants	N = 164 females 18 to 24 years	
Interventions	Gardasil (Merck quadrivalent)	
Outcomes	Incident HPV infection Follow‐up: 1 year (mean)	
Notes	Source of funding: public/non‐profit: National Institutes of Health Conflicts of interest: no

**Table:** Machalek 2018‐AUS

***Study characteristics***		
Methods	Pre‐ vs post‐vaccine introduction Australia; 2005‐2015 V: National HPV Vaccination Program Register O: 1 mL of the PreservCyt specimen was tested for the presence of 14 high‐risk HPV types using the cobas HPV test	
Participants	N = 656 females 18 to 35 years	
Interventions	Gardasil (Merck quadrivalent)	
Outcomes	Prevalent HPV infection Follow‐up: cross‐sectional, 8 years apart	
Notes	Source of funding: public/non‐profit: Australian Government Department of Health HPV Surveillance Fund Conflicts of interest: authors include stockholders of the vaccine developer

**Table:** Mann 2019‐USA

***Study characteristics***		
Methods	Pre‐ vs post‐vaccine introduction USA; January 2010‐December 2016 V: no individual vaccination status O: Centers for Disease Control and Prevention’s STD Surveillance Network (SSuN) (study‐level targeted ascertainment)	
Participants	N = 653,847 females and males (attending sexual health clinic) All ages	
Interventions	Gardasil (Merck quadrivalent)	
Outcomes	Anogenital warts Follow‐up: cross‐sectional, repeated over 7 years	
Notes	Source of funding: unclear Conflicts of interest: no

**Table:** Markowitz 2019‐USA

***Study characteristics***		
Methods	Cohort study USA; 2007, 2012‐2013, 2015‐2016 V: Kaiser Permanente database O: cytology samples	
Participants	N = 12,788 females 20 to 29 years	
Interventions	Gardasil (Merck quadrivalent)	
Outcomes	Prevalent HPV infection Follow‐up: cross‐sectional	
Notes	Source of funding: public/non‐profit: Centers for Disease Control and Prevention Conflicts of interest: some authors received funding from the vaccine developer

**Table:** Markowitz 2020‐USA

***Study characteristics***		
Methods	Cohort study USA; June 2006‐February 2017 V: Kaiser Permanente medical insurance records O: Kaiser Permanente medical insurance records	
Participants	N = 4269 females 20 to 29 years	
Interventions	Gardasil (Merck quadrivalent)	
Outcomes	Prevalent HPV infection Follow‐up: 11 years	
Notes	Source of funding: public/non‐profit: Centers for Disease Control and Prevention Conflicts of interest: some authors received funding from the vaccine developer

**Table:** Martellucci 2022‐ITA

***Study characteristics***		
Methods	Retrospective cohort study Italy; January 2015‐June 2020 V: local health agency registry O: local health agency registry	
Participants	N = 4665 females 25 to 30 years	
Interventions	Cervarix (GSK bivalent); Gardasil (Merck quadrivalent)	
Outcomes	CIN2+ Follow‐up: up to 5 years	
Notes	Funding: public/non‐profit: Italian Ministry of Health center for disease control and prevention Conflicts of interest: no

**Table:** Martin‐Merino 2021‐ESP

***Study characteristics***		
Methods	Cohort study Spain; January 2007‐December 2016 V: Spanish Primary Care Database for Pharmacoepidemiological Research (national database) O: Spanish Primary Care Database for Pharmacoepidemiological Research (national database)	
Participants	N = 388,849 females 9 to 28 years	
Interventions	Not reported	
Outcomes	Guillain‐Barré syndrome (GBS) Follow‐up: 10 years	
Notes	Source of funding:public/non‐profit: Instituto de Salud Carlos III (Co‐funded by European Regional Development Fund) Conflicts of interest: no

**Table:** McDaniel 2020‐USA

***Study characteristics***		
Methods	Cross‐sectional study USA; 2011‐2014 V: self‐report O: oral samples	
Participants	N = 822 females and males 30 to 33 years	
Interventions	Gardasil (Merck quadrivalent)	
Outcomes	Prevalent HPV infection Follow‐up: cross‐sectional	
Notes	Source of funding: not reported Conflicts of interest: no

**Table:** McGregor 2018‐AUS

***Study characteristics***		
Methods	Pre‐ vs post‐vaccine introduction Australia; 2005‐2015 V: Australian National HPV Vaccination Program Register (NHVPR) O: laboratory methods for HPV genotype detection	
Participants	N = 297 females 18 to 26 years	
Interventions	Gardasil (Merck quadrivalent)	
Outcomes	Prevalent HPV infection Follow‐up: cross‐sectional	
Notes	Source of funding: public/non‐profit: Australian Government Department of Health Conflicts of interest: some authors received funding from the vaccine developer

**Table:** McInerney 2017‐USA

***Study characteristics***		
Methods	Cohort study United States of America (USA); 2013‐2017 V: self‐report O: self‐report (study‐level targeted ascertainment)	
Participants	N = 4505 females and males Mean age: 25 to 32 years	
Interventions	Gardasil (Merck quadrivalent)	
Outcomes	Infertility Follow‐up: not reported	
Notes	Source of funding: public/non‐profit: Eunice Kennedy Shriver National Institute of Child Health and Human Development, National Institute of Health, Danish Cancer Society Conflicts of interest: not reported

**Table:** Mehanna 2019‐GBR

***Study characteristics***		
Methods	Cross‐sectional study United Kingdom (UK); 2013‐2015 V: regional health authorities O: oral samples (oral rinse, either of the oral brushes, or the tonsillar tissue samples)	
Participants	N = 212 females and males 12 to 24 years	
Interventions	Cervarix (GSK bivalent)	
Outcomes	Prevalent HPV infection Follow‐up: cross‐sectional	
Notes	Source of funding: private/industry: GlaxoSmithKline Biologicals SA (GSK) Conflicts of interest: authors include employees of the vaccine developer

**Table:** Mesher 2018‐GBR

***Study characteristics***		
Methods	Pre‐ vs post‐vaccine introduction United Kingdom (UK); 2010‐2016 V: data obtained from laboratories from the chlamydia test request form; data obtained by linkage with local Child Health Information Service (CHIS) Systems O: vulva‐vaginal swab specimens	
Participants	N = 2318 females 16 to 24 years	
Interventions	Cervarix (GSK bivalent)	
Outcomes	Prevalent HPV infection Follow‐up: cross‐sectional	
Notes	Source of funding: public/non‐profit: Public Health England Conflicts of interest: some authors received funding from the vaccine developer

**Table:** Miranda 2017‐FRA

***Study characteristics***		
Methods	Cohort study France; January 2008‐December 2013 V: French national health insurance anonymised claim database (SNIIRAM)/national hospital discharge database (PMSI) (national database) O: French national health insurance anonymised claim database (SNIIRAM)/national hospital discharge database (PMSI) (national database)	
Participants	N = 2,252,716 females 13 to 17 years	
Interventions	Cervarix (GSK bivalent), Gardasil (Merck quadrivalent)	
Outcomes	Guillain‐Barré syndrome (GBS) Follow‐up: mean 33 months	
Notes	Source of funding: not reported Conflicts of interest: no

**Table:** Mix 2022‐USA

***Study characteristics***		
Methods	Pre‐ vs post‐vaccine introduction USA; 2000‐2017 V: no individual vaccine status O: Surveillance, Epidemiology, and End Results (SEER) Program ‐ 18 central cancer registries covering 27.8% of the U.S. population	
Participants	N = not reported, females 15 to 39 years	
Interventions	Not reported	
Outcomes	VaIN; VIN; AIN Follow‐up: 17 years	
Notes	Source of funding: public/non‐profit: Oak Ridge Institute for Science and Education, an asset of the U.S. Department of Energy. Conflicts of interest: not reported

**Table:** Munoz‐Quiles 2021‐ESP

***Study characteristics***		
Methods	Cohort study Spain; January 2009‐December 2017 V: Vaccine Information System (VIS); The Valencia healthcare Integrated Databases (VID) (national, regional database) O: The Valencia healthcare Integrated Databases (VID) (national, regional database)	
Participants	N = 563,240 females 14 to 23 years	
Interventions	Gardasil (Merck quadrivalent)	
Outcomes	Anogenital warts Follow‐up: 9 years	
Notes	Source of funding: private/industry: MSD Conflicts of interest: some authors received funding from the vaccine developer

**Table:** Munro 2017‐GBR

***Study characteristics***		
Methods	Cross‐sectional study United Kingdom (UK); December 2012‐November 2014 V: self‐report (verified by the Scottish Cervical Call Recall System (SCCRS)) O: colposcopy results following an abnormal cytology result at routine cervical screening (national database)	
Participants	N = 163 females attending colposcopy 20 to 25 years	
Interventions	Gardasil (Merck quadrivalent)	
Outcomes	CIN3; CIN2+; CIN2 Follow‐up: mean age last dose: 17.3; mean age colposcopy: 22	
Notes	Source of funding: public/non‐profit: Chief Scientist Office, Scotland; NHS; The Jean Shanks Foundation Conflicts of interest: no

**Table:** Muresu 2022‐ITA

***Study characteristics***		
Methods	Cross‐sectional study Italy; March 2016‐December 2020 V: self‐report O: regional Pap screening programme, Sassari	
Participants	N = 1186 females 25 to 64 years	
Interventions	Gardasil (Merck quadrivalent); Gardasil 9 (Merck nonavalent)	
Outcomes	CIN2+ Follow‐up: cross‐sectional	
Notes	Funding: none Conflict of interest: none

**Table:** Naleway 2020‐USA

***Study characteristics***		
Methods	Pre‐ vs post‐vaccine introduction USA; January 2000‐December 2016 V: no individual vaccination status O: Kaiser Permanente Northwest electronic medical record system (insurance database)	
Participants	N = 565,356 females and males 11 to 39 years	
Interventions	Gardasil (Merck quadrivalent)	
Outcomes	Anogenital warts Follow‐up: cross‐sectional, repeated over 16 years	
Notes	Source of funding: public/non‐profit: US Centers for Disease Control and Prevention Conflicts of interest: no

**Table:** Napolitano 2024‐ITA

***Study characteristics***		
Methods	Cross‐sectional study Italy, November 2022 – September 2023 V: self‐report O: self‐sampling saliva and urine samples	
Participants	N = 1002 males and females 18 to 30 years	
Interventions	Cervarix (GSK bivalent), Gardasil (Merck quadrivalent), Gardasil 9 (Merck nonavalent)	
Outcomes	Prevalent HPV infection Follow‐up: cross‐sectional	
Notes	Source of funding: public/non‐profit: Italian Ministry of University and Research Conflicts of interest: none

**Table:** Nilyanimit 2024‐THA

***Study characteristics***		
Methods	Cross‐sectional study Thailand; 2023 V: defined by location, school vaccination programme O: self‐sampled urine	
Participants	N = 587 females 16 to 18 years	
Interventions	Cervarix (GSK bivalent)	
Outcomes	Prevalent HPV infection Follow‐up: 7 years	
Notes	Source of funding: National Research Council of Thailand, Health Systems Research Institute, the Center of Excellence in Clinical Virology at Chulalongkorn University, Kind Chulalongkorn Memorial Hospital, the MK Restaurant Group and Aunt Thongkham Foundation, the Department of Disease Control and the Education and Public Welfare Foundation Conflicts of interest: none

**Table:** Nsouli‐Maktabi 2013‐USA

***Study characteristics***		
Methods	Pre‐ vs post‐vaccine introduction USA; January 2000‐December 2012 V: no individual vaccination status O: the Defense Medical Surveillance System (DMSS) (insurance database)	
Participants	N = 1,544,029 (2000), 1,440,362 (2012) females and males (armed forces) Age not reported	
Interventions	Gardasil (Merck quadrivalent)	
Outcomes	Anogenital warts Follow‐up: cross‐sectional, repeated over 13 years	
Notes	Source of funding: not reported Conflicts of interest: not reported

**Table:** Nygard 2023‐NOR

***Study characteristics***		
Methods	Retrospective cohort study/database linkage Norway; January 2006‐December 2016 V: Norwegian Immunisation Registry O: Norwegian Prescription Database and the Norwegian Patient Registry	
Participants	N = 2,187,724 males and females 13 to 31 years	
Interventions	Gardasil (Merck quadrivalent)	
Outcomes	Anogenital warts Follow‐up: 3 years	
Notes	Funding: not reported Conflicts of interest: some authors received funding from the vaccine developer

**Table:** Oliphant 2011‐NZL

***Study characteristics***		
Methods	Pre‐ vs post‐vaccine introduction New Zealand; January 2007‐June 2010 V: no individual vaccination status O: Auckland Sexual Health Service (hospital database)	
Participants	N = 40,793 females and males (attending sexual health clinic) Age not reported	
Interventions	Gardasil (Merck quadrivalent)	
Outcomes	Anogenital warts Follow‐up: cross‐sectional repeated	
Notes	Source of funding: not reported Conflicts of interest: no

**Table:** Onuki 2023‐JPN

***Study characteristics***		
Methods	Pre‐ vs post‐vaccine introduction Japan; 1975‐2020 V: individual vaccination status not used – pre‐/post‐vaccine introduction O: nationwide hospital‐based cancer registry	
Participants	N = 418,918 cases of cervical cancer in females 20+ years of age	
Interventions	Cervarix (GSK bivalent), Gardasil (Merck quadrivalent)	
Outcomes	Cervical cancer	
Notes	Funding: Japan Agency for Medical Research and Development Conflicts of interest: none

**Table:** Orumaa 2020‐NOR/DNK

***Study characteristics***		
Methods	Pre‐ vs post‐vaccine introduction Norway and Denmark; 2006–2015 V: The Norwegian Immunization Registry (SYSVAK); The Danish National Prescription Registry (national databases) O: The Norwegian Patient Registry; The Norwegian Prescription Database; The Danish National Patient Register; The Danish National Prescription Registry; The Danish National Health Service Register (national databases)	
Participants	N = 30,866,417 person‐years females and males 12 to 35 years	
Interventions	Gardasil (Merck quadrivalent)	
Outcomes	Anogenital warts Follow‐up: cross‐sectional (repeated yearly)	
Notes	Funding: no specific funding Conflicts of interest: some authors received funding from the vaccine developer

**Table:** Orumaa 2024‐NOR

***Study characteristics***		
Methods	Retrospective cohort study/database linkage Norway; January 2007‐December 2020 V: Norwegian Immunization Registry O: Norwegian Cervical Cancer Screening Program	
Participants	N = 868,403 females 16 to 30 years	
Interventions	Gardasil (Merck quadrivalent)	
Outcomes	CIN2+; CIN3+ Follow‐up: 11 years	
Notes	Funding: MSD (Norge) AS (grant to the Cancer Registry of Norway) Conflict of interest: some authors are employees and others received grants from the vaccine developer

**Table:** Osmani 2022‐DEU

***Study characteristics***		
Methods	Retrospective cohort study Germany; 2008‐2018 V: Bavarian Association of Statutory Health Insurance Physicians O: Bavarian Association of Statutory Health Insurance Physicians	
Participants	N = 433,346 females 19 to 28 years	
Interventions	Gardasil (Merck quadrivalent); Cervarix (GSK bivalent); Gardasil 9 (Merck nonavalent)	
Outcomes	Anogenital warts Follow‐up: 10 years	
Notes	Funding: Open Access funding enabled and organised by Projekt DEAL Conflicts of interest: none

**Table:** Ounchanum 2024‐THA/VNM

***Study characteristics***		
Methods	Prospective cohort study Thailand/Vietnam; 2013‐2018 V: self‐report O: anogenital sampling	
Participants	N = 192 females 12 to 24 years	
Interventions	Cervarix (GSK bivalent)	
Outcomes	Persistent HPV infection Follow‐up: 3 years	
Notes	Source of funding: US National Institute of Health Conflicts of interest: none

**Table:** Ozawa 2017‐JPN

***Study characteristics***		
Methods	Cross‐sectional study Japan; April 2014‐March 2016 V: self‐report O: Miyagi Cancer Society (regional database)	
Participants	N = 5924 females attending cervical screening 20 to 24 years	
Interventions	Cervarix (GSK bivalent), Gardasil (Merck quadrivalent)	
Outcomes	CIN3+; CIN2+ Follow‐up: median about 7 years post‐vaccination (0 to 4 weeks between the 2 visits)	
Notes	Source of funding: not reported Conflicts of interest: no

**Table:** Palmer 2019‐GBR

***Study characteristics***		
Methods	Cohort study United Kingdom (UK); 2008‐2016 V: Scottish Immunisation Call‐Recall System (national database) O: Information Services Division (ISD) of the Scottish National Health Service (national database)	
Participants	N = not reported, females attending cervical screening 20 to 21 years	
Interventions	Cervarix (GSK bivalent)	
Outcomes	CIN3+; CIN2; prevalent HPV infection Follow‐up: up to 6 years	
Notes	Source of funding:public/non‐profit: Health Protection Scotland, a part of the Scottish National Health Service Conflicts of interest: some authors received expenses from the vaccine developer

**Table:** Palmer 2024‐GBR

***Study characteristics***		
Methods	Retrospective cohort study/database linkage United Kingdom (UK); 2020 V: Scottish Cervical Cancer Call Recall System (national database) O: Scottish Cancer Registry (national database)	
Participants	N = 447,845 females 24 to 32 years	
Interventions	Cervarix (GSK bivalent)	
Outcomes	Cervical cancer Follow‐up: up to 12 years	
Notes	Funding: Scottish government through core funding of Public Health Scotland Conflicts of interest: none

**Table:** Paraskevaidis 2020‐GRC

***Study characteristics***		
Methods	Cohort study Greece; 2009‐2019 V: not reported O: all colposcopic evaluations were performed in each department	
Participants	N = 1698 females Age not reported	
Interventions	Not reported	
Outcomes	CIN3; CIN2; treatment rates for CIN and other HPV‐related disease Follow‐up: not reported	
Notes	Source of funding: not reported Conflicts of interest: no

**Table:** Perkins 2015‐USA

***Study characteristics***		
Methods	Pre‐ vs post‐vaccine introduction USA; 2004‐2013 V: electronic medical record (hospital database) O: administrative data (hospital database)	
Participants	N = 45,787 females and males (primary health care) 16 to 26 years	
Interventions	Gardasil (Merck quadrivalent)	
Outcomes	Anogenital warts Follow‐up: cross‐sectional, repeated over 9 years	
Notes	Source of funding: public/non‐profit: American Cancer Society Conflicts of interest: no

**Table:** Perkins 2017‐USA

***Study characteristics***		
Methods	Cohort study USA; January 2007‐December 2013 V: Truven Health Analytics Marketscan Commercial Claims Database (insurance database) O: Truven Health Analytics Marketscan Commercial Claims Database (insurance database)	
Participants	N = 387,906 females 9 to 25 years	
Interventions	Gardasil (Merck quadrivalent)	
Outcomes	Anogenital warts Follow‐up: average 5.64 years	
Notes	Source of funding: public/non‐profit: American Cancer Society Conflicts of interest: no

**Table:** Petras 2015‐CZE

***Study characteristics***		
Methods	Cross‐sectional study Czech Republic; January 2013‐March 2014 V: self‐administered questionnaire (study‐level targeted ascertainment) O: self‐administered questionnaire (study‐level targeted ascertainment)	
Participants	N = 19199 females (primary health care) 16 to 40 years	
Interventions	Gardasil (Merck quadrivalent)	
Outcomes	Anogenital warts Follow‐up: not reported	
Notes	Source of funding: not reported Conflicts of interest: some authors received lecture fees from a vaccine developer

**Table:** Purrinos‐Hermida 2018‐ESP

***Study characteristics***		
Methods	Pre‐ vs post‐vaccine introduction Spain; 2008‐2017 V: electronic clinical history/questionnaire O: self‐filled questionnaire/cervical scrapings	
Participants	N = 1268 females 18 to 26 years	
Interventions	Cervarix (GSK bivalent)	
Outcomes	Prevalent HPV infection Follow‐up: cross‐sectional	
Notes	Source of funding: public/non‐profit: Direccion xeral de Saude Publica Edificio Administrativo S. Lazaro s/n Santiago de Compostela (Galicia ‐ Spain) Conflicts of interest: no

**Table:** Rana 2013‐FIN

***Study characteristics***		
Methods	RCT extension Finland; 2006‐2012 V: RCT records O: Finnish Cancer Registry (national database)	
Participants	N = 16,584 females (trial participants, community controls) 20 to 26 years	
Interventions	Gardasil (Merck quadrivalent)	
Outcomes	Cervical cancer; CIN3 Follow‐up: up to 9 years after vaccination	
Notes	Source of funding: mixed: public/non‐profit and private/industry: Finnish Cancer Organizations and Nordic Cancer Union, Merck & Co. Inc., GSK Biologicals Conflicts of interest: some authors received funding from the vaccine developer

**Table:** Rasmussen 2020‐DNK

***Study characteristics***		
Methods	Pre‐ vs post‐vaccine introduction Denmark; 1997‐2018 V: individual vaccination status not used – pre‐/post‐introduction O: Danish Cancer Registry (national database)	
Participants	N = not reported, females All ages	
Interventions	Not reported	
Outcomes	Vulvar cancer; VIN Follow‐up: 4 years	
Notes	Source of funding: not reported Conflicts of interest: some authors received funding from the vaccine developer

**Table:** Rebolj 2022‐GBR

***Study characteristics***		
Methods	Pre‐ vs post‐vaccine introduction United Kingdom; 2013‐2018 V: individual vaccination status not used – pre‐/post‐introduction O: National Health Service Cervical Screening Programme (national database)	
Participants	N = 64,274 females 24 to 25 years	
Interventions	Cervarix (GSK bivalent)	
Outcomes	Cervical cancer; CIN3+; CIN2+ Follow‐up: 7 years	
Notes	Funding: Public Health England Conflicts of interest: none

**Table:** Restivo 2023‐ITA

***Study characteristics***		
Methods	Pre‐ vs post‐vaccine introduction Italy; 2008‐2018 V: individual vaccination status not used – pre‐/post‐introduction O: Italian hospital discharge records database	
Participants	N = 483,373 females and males Age not reported	
Interventions	Gardasil (Merck quadrivalent), Gardasil 9 (Merck nonavalent)	
Outcomes	Cervical cancer, anal cancer, head and neck cancer, penile cancer, vulvar cancer, anogenital warts Follow‐up: cross‐sectional (repeated)	
Notes	Funding: none Conflict of interest: none

**Table:** Reyburn 2023‐FJI

***Study characteristics***		
Methods	Retrospective cohort study Fiji; October 2015 to March 2019 V: HPV immunisation register O: vaginal swab as part of antenatal testing	
Participants	N = 835 pregnant women 15 to 23 years	
Interventions	Gardasil (Merck quadrivalent)	
Outcomes	Prevalent HPV infection; anogenital warts Follow‐up: 6 to 11 years	
Notes	Source of funding: Bill & Melinda Gates Foundation and the Department of Foreign Affairs and Trade of the Australian Government and Fiji Health Sector Support Program (FHSSP) Conflicts of interest: some authors received funding from the vaccine developer

**Table:** Rodriguez 2020‐USA

***Study characteristics***		
Methods	Cohort study USA; January 2006‐December 2016 V: Optum’s Clinformatics DataMart Database (insurance database) O: Optum’s Clinformatics DataMart Database (insurance database)	
Participants	N = 133,082 females Age not reported	
Interventions	Gardasil (Merck quadrivalent)	
Outcomes	CIN2+ Follow‐up: 22 years	
Notes	Source of funding: public/non‐profit: National Institutes of Health; Cancer Prevention Research Institute of Texas Conflicts of interest: some authors received funding from the vaccine developer

**Table:** Rosenblum 2021‐USA

***Study characteristics***		
Methods	Cohort study; pre‐ vs post‐vaccine introduction USA; 2003‐2018 V: self‐report O: self‐collected cervicovaginal specimens (NHANES)	
Participants	N = not reported, females 14 to 69 years	
Interventions	Gardasil (Merck quadrivalent)	
Outcomes	Prevalent HPV infection Follow‐up: cross‐sectional, repeated over 16 years	
Notes	Source of funding: public/non‐profit: NIH, Cancer Prevention and Research Institute of Texas Conflicts of interest: no

**Table:** Ruiz‐Sternberg 2014‐COL

***Study characteristics***		
Methods	Cohort study Colombia; May 2011‐March 2012 V: self‐administered survey O: self‐administered survey	
Participants	N = 1436 females < 26 years	
Interventions	Not reported	
Outcomes	Participation rates in screening Follow‐up: cross‐sectional	
Notes	Source of funding: private/industry: Merck Conflicts of interest: no

**Table:** Sadler 2015‐GBR

***Study characteristics***		
Methods	Cohort study; cross‐sectional United Kingdom (UK); September 2010‐October 2011 V: standardised clinical history form administered on successive consenting attendees by clinicians at genitourinary medicine clinics (study‐level targeted ascertainment) O: standardised clinical history form administered on successive consenting attendees by clinicians at genitourinary medicine clinics (study‐level targeted ascertainment)	
Participants	N = 363 females 14 to 20 years	
Interventions	Cervarix (GSK bivalent), Gardasil (Merck quadrivalent)	
Outcomes	Sexual activity (incidence of sexually transmitted infections); anogenital warts Follow‐up: cross‐sectional	
Notes	Source of funding: public/non‐profit: Medical Research Council Studentship; Max Elstein Trust ; Central Manchester University Hospitals NHS Foundation Trust Conflicts of interest: no

**Table:** Saeki 2024‐JPN

***Study characteristics***		
Methods	Cross‐sectional study; pre‐ vs post‐vaccine introduction Japan; April 2021‐November 2022 V: self‐report O: outpatient clinics with HPV screening	
Participants	N = 1529 females 16 to 39 years	
Interventions	Cervarix (GSK bivalent); Gardasil (Merck quadrivalent); Gardasil 9 (Merck nonavalent)	
Outcomes	Prevalent HPV infection Follow‐up: cross‐sectional	
Notes	Funding: research grant from vaccine manufacturer Conflicts of interest: none

**Table:** Saldanha 2020‐PRT

***Study characteristics***		
Methods	Pre‐ vs post‐vaccine introduction Portugal; January 2010‐December 2019 V: not reported. Defined by birth cohorts. O: HPV test outcomes at a single laboratory	
Participants	N = 1852 females < 25 years	
Interventions	Gardasil (Merck quadrivalent)	
Outcomes	Prevalent HPV infection Follow‐up: not reported	
Notes	Funding: no specific funding Conflicts of interest: authors have received speaker fees from a vaccine developer

**Table:** Sando 2014‐DNK

***Study characteristics***		
Methods	Pre‐ vs post‐vaccine introduction Denmark; 2001‐2011 V: no individual vaccination status O: Register of Medical Products Statistics combined with data from the National Patient Register (national database)	
Participants	N = not reported, females and males 15 to 34 years	
Interventions	Gardasil (Merck quadrivalent)	
Outcomes	Anogenital warts Follow‐up: 2 years	
Notes	Source of funding: not reported Conflicts of interest: some authors received funding from the vaccine developer

**Table:** Sankaranarayanan 2018‐IND

***Study characteristics***		
Methods	RCT extension India; September 2009‐June 2019 V: RCT records O: cervical samples (study‐level targeted ascertainment)	
Participants	N = 21,258 females (trial participants, community controls) 20 to 28 years	
Interventions	Gardasil (Merck quadrivalent)	
Outcomes	Cervical cancer; CIN2+; incident HPV infection; persistent HPV infection Follow‐up: 5 years	
Notes	Source of funding: mixed: public/non‐profit and private/industry: Bill & Melinda Gates Foundation; Merck Conflicts of interest: some authors received funding from the vaccine developer

**Table:** Sarr 2019‐CAN

***Study characteristics***		
Methods	Cross‐sectional study Canada; 2010‐2016 V: self‐report O: self‐collected cervicovaginal specimen, using a dry polyester swab	
Participants	N = 1035 females ≥ 18 years	
Interventions	Gardasil (Merck quadrivalent)	
Outcomes	Prevalent HPV infection Follow‐up: cross‐sectional	
Notes	Source of funding: public/non‐profit: Canadian Institutes of Health Research (CHIR) Conflicts of interest: some authors received funding from the vaccine developer

**Table:** Sauvageau 2021‐CAN

***Study characteristics***		
Methods	Cross‐sectional study Canada; 2013‐2014 V: computer‐assisted questionnaire (self‐report) (study‐level targeted ascertainment) O: computer‐assisted questionnaire (self‐report) (study‐level targeted ascertainment)	
Participants	N = 1475 females 17 to 29 years	
Interventions	Gardasil (Merck quadrivalent)	
Outcomes	Sexual activity (incidence of sexually transmitted infections); participation rates in screening Follow‐up: cross‐sectional	
Notes	Source of funding: public/non‐profit: Ministére de la Sante et des Services Sociaux du Quebec Conflicts of interest: some authors received funding from the vaccine developer

**Table:** Sayinzoga 2023‐RWA

***Study characteristics***		
Methods	Cross‐sectional study Rwanda; July 2013‐December 2020 V: self‐report survey O: cervical cell samples for the detection of HPV DNA	
Participants	N = 3140 females 17 to 29 years	
Interventions	Gardasil (Merck quadrivalent)	
Outcomes	Prevalent HPV infection Follow‐up: cross‐sectional, repeated	
Notes	Source of Funding: Bill & Melinda Gates Foundation Conflicts of interest: none

**Table:** Scheller 2017‐DNK

***Study characteristics***		
Methods	Cohort study Denmark; October 2006‐November 2013 V: Childhood Vaccination database at Statens Serum Institut; The National Prescription Register (national database) O: The Medical Birth Register; The National Patient Register (national database)	
Participants	N = not reported, females 12 to 27 years	
Interventions	Gardasil (Merck quadrivalent)	
Outcomes	Birth outcomes Follow‐up: 7 years	
Notes	Source of funding: public/non‐profit: Novo Nordisk Foundation; Danish Medical Research Council Conflicts of interest: no

**Table:** Schlecht 2016‐USA

***Study characteristics***		
Methods	Cohort study USA; study dates not reported V: medical records O: specimen collection was performed at each 6‐month visit by clinicians	
Participants	N = 1139 females Age not reported	
Interventions	Gardasil (Merck quadrivalent)	
Outcomes	Prevalent HPV infection Follow‐up: median = 28.5 months ; mean = 30.9 (± 23.1)	
Notes	Source of funding: public/non‐profit: National Institute of Allergy and Infectious Diseases; Icahn School of Medicine at Mount Sinai; National Cancer Institute Conflicts of interest: some authors received funding from the vaccine developer

**Table:** Schlecht 2019‐USA

***Study characteristics***		
Methods	Cohort study USA; October 2007‐April 2017 V: medical records O: oral rinse sample	
Participants	N = 1259 females 13 to 21 years	
Interventions	Gardasil (Merck quadrivalent)	
Outcomes	Prevalent HPV infection Follow‐up: cross‐sectional	
Notes	Source of funding: public/non‐profit: National Institute of Allergy and Infectious Diseases; National Cancer Institute; Icahn School of Medicine at Mount Sinai Conflicts of interest: some authors received funding from the vaccine developer

**Table:** Schmuhl 2020‐USA

***Study characteristics***		
Methods	Cross‐sectional study United States of America (USA); 2006‐2016 V: self report: National Health and Nutrition Examination Survey (NHANES) (study‐level targeted ascertainment) O: self report: National Health and Nutrition Examination Survey (NHANES) (study‐level targeted ascertainment)	
Participants	N = 1114 females 18 to 33 years	
Interventions	Not reported	
Outcomes	Infertility Follow‐up: 10 years	
Notes	Source of funding: public/non‐profit: University of Wisconsin Carbone Cancer Center Conflicts of interest: not reported

**Table:** Schurink‐Van't Klooster 2018‐NLD

***Study characteristics***		
Methods	Pre‐ vs post‐vaccine introduction Netherlands; January 2007‐December 2014 V: electronic national immunisation register 'Præventis’ (national database) O: electronic records (study‐level targeted ascertainment)	
Participants	N = 69,429 females 12 to 16 years	
Interventions	Cervarix (GSK bivalent)	
Outcomes	Chronic fatigue syndrome/myalgic encephalomyelitis (CFS/ME) Follow‐up: 5 years	
Notes	Source of funding: public/non‐profit: Dutch Ministry of Health, ZONMW Conflicts of interest: no

**Table:** Schurink‐Van't Klooster 2023‐NLD

***Study characteristics***		
Methods	Retrospective cohort study/database linkage Netherlands; January 2009‐March 2018 V: national vaccination registry (Praeventis) O: Dutch National Pathology Databank (PALGA)	
Participants	N = 42,171 females 13 to 22 years	
Interventions	Cervarix (GSK bivalent); Gardasil (Merck quadrivalent); Gardasil 9 (Merck nonavalent)	
Outcomes	CIN3+ Follow‐up: up to 10 years	
Notes	Funding: Ministry of Health, Welfare, and Sport, The Netherlands Conflicts of interest: none

**Table:** Shiko 2020‐JPN

***Study characteristics***		
Methods	Cross‐sectional study Japan; April 2015‐March 2017 V: questionnaire (self‐report) O: Japan Cancer Society cervical screening database (national database)	
Participants	N = 34,281 females (attending cervical screening) 20 to 29 years	
Interventions	Cervarix (GSK bivalent)	
Outcomes	CIN3+; CIN2+ Follow‐up: 10 years	
Notes	Source of funding: public/non‐profit: Research Programme on Emerging and Re‐emerging Infectious Diseases from Japan Agency for Medical Research and Development Conflicts of interest: some authors received funding from the vaccine developer

**Table:** Shilling 2021‐AUS

***Study characteristics***		
Methods	Cohort study Australia; January 2015‐November 2018 V: National HPV Vaccination Program Register O: HPV DNA detection and genotyping	
Participants	N = 1635 females 18 to 35 years	
Interventions	Gardasil (Merck quadrivalent)	
Outcomes	Prevalent HPV infection Follow‐up: cross‐sectional, up to 8 years since vaccination	
Notes	Source of funding: public/non‐profit: Commonwealth Department of Health HPV Surveillance Fund Conflicts of interest: some authors received funding from the vaccine developer

**Table:** Shing 2019‐USA

***Study characteristics***		
Methods	Pre‐ vs post‐vaccine introduction USA; January 2006‐December 2014 V: no individual vaccination status O: TennCare, Tennessee's Medicaid program (insurance database)	
Participants	N = 799,122 females and males 15 to 39 years	
Interventions	Gardasil (Merck quadrivalent)	
Outcomes	Anogenital warts Follow‐up: cross‐sectional, repeated analyses	
Notes	Source of funding: public/non‐profit: Centers for Disease Control and Prevention; National Center for Advancing Translational Sciences, National Institutes of Health Conflicts of interest: no

**Table:** Silverberg 2018‐USA

***Study characteristics***		
Methods	Case‐control study USA; 2006‐2014 V: Kaiser Permanente Northern California electronic health record O: Kaiser Permanente Northern California electronic health record	
Participants	N = 26,130 females Age not reported	
Interventions	Gardasil (Merck quadrivalent)	
Outcomes	CIN3+; CIN2+ Follow‐up not reported	
Notes	Source of funding: public/non‐profit: National Cancer Institute at the National Institutes of Health Conflicts of interest: no

**Table:** Skufca 2018‐FIN

***Study characteristics***		
Methods	Cohort study Finland; November 2013‐December 2016 V: Finnish National Vaccination Register (national database) O: national hospital discharge register (national database)	
Participants	N = 240,605 females 11 to 15 years	
Interventions	Cervarix (GSK bivalent)	
Outcomes	Postural orthostatic tachycardia syndrome (POTS); chronic fatigue syndrome/myalgic encephalomyelitis (CFS/ME); paralysis; complex regional pain syndrome (CRPS); Guillain‐Barré syndrome (GBS) Follow‐up: 3 years	
Notes	Source of funding: not reported Conflicts of interest: authors are employed by the National Institute for Health and Welfare, which has received research funding from GlaxoSmithKline and Pfizer, Inc.

**Table:** Smith 2015‐CAN

***Study characteristics***		
Methods	Pre‐ vs post‐vaccine introduction Canada; 2005‐2009 V: Immunization Records Information System (IRIS) (regional database) O: "Registered Persons’ Database; Ontario Health; Insurance Plan; Discharge Abstract Database; Same‐Day Surgeries; National Ambulatory Care Reporting System" (routine administrative database, national)	
Participants	N = 260,493 females 13 to 17 years	
Interventions	Gardasil (Merck quadrivalent)	
Outcomes	Sexual activity (incidence of sexually transmitted infections) Follow‐up: 4 years	
Notes	Source of funding: public/non‐profit: Canadian Institutes of Health Research; Institute for Clinical Evaluative Sciences Conflicts of interest: no

**Table:** Smith 2016‐AUS

***Study characteristics***		
Methods	Pre‐ vs post‐vaccine introduction Australia; 1 July 1999 and 30 June 2011 V: no individual vaccination status O: National Hospital Morbidity Database (NHMD) (national database)	
Participants	N = 39,350 females and males 12 to 69 years	
Interventions	Gardasil (Merck quadrivalent)	
Outcomes	Anogenital warts Follow‐up: up to 5 years	
Notes	Source of funding: public/non‐profit: National Health and Medical Research Council Australia; The National Centre for Immunisation Research; Australian Government Department of Health, the NSW Ministry of Health, the Children’s Hospital at Westmead Conflicts of interest: no

**Table:** Soderlund‐Strand 2014‐SWE

***Study characteristics***		
Methods	Pre‐ vs post‐vaccine introduction Sweden; March 2008‐March 2013 V: individual vaccination status not used O: chlamydia screening	
Participants	N = 55,185 females and males All ages	
Interventions	Gardasil (Merck quadrivalent)	
Outcomes	Prevalent HPV infection Follow‐up: 3 repeated cross‐sectional surveys, over 6 years	
Notes	Source of funding: public/non‐profit: Public Health Agency of Sweden Conflicts of interest: some authors received funding from the vaccine developer

**Table:** Sonnenberg 2019‐GBR

***Study characteristics***		
Methods	Pre‐ vs post‐vaccine introduction United Kingdom (UK); 1999‐2012 V: no individual vaccination status O: self‐reported GW diagnosis	
Participants	N = 18,963 females and males 16 to 44 years	
Interventions	Cervarix (GSK bivalent)	
Outcomes	Anogenital warts Follow‐up: cross‐sectional	
Notes	Source of funding: public/non‐profit: Medical Research Council; Wellcome trust; Economic and Social Research Council; Department of Health Conflicts of interest: no

**Table:** Spinner 2019‐USA

***Study characteristics***		
Methods	Pre‐ vs post‐vaccine introduction USA; 2006‐2017 V: Ohio statewide immunisation registry; electronic health record; self‐report O: cervicovaginal swabs (self‐swab or clinician swab)	
Participants	N = 1580 females 13 to 26 years	
Interventions	Gardasil (Merck quadrivalent)	
Outcomes	Prevalent HPV infection Follow‐up: cross‐sectional, repeated over 12 years	
Notes	Source of funding: both public/non‐profit and private/industry sources: National Institutes of Health; Merck provided vaccine and serology testing Conflicts of interest: authors include stockholders of the vaccine developer

**Table:** Steben 2018‐CAN

***Study characteristics***		
Methods	Pre‐ vs post‐vaccine introduction Canada; 2004‐2012 V: school public HPV vaccination programme with the quadrivalent vaccine Gardasil O: the provincial administrative databases of the Régie de l'Assurance Maladie du Québec (RAMQ): the physician service claims (PSCs) and the public drug plan insurance databases (insurance databases)	
Participants	N = 21,411 females and males 15 to ≥ 30 years	
Interventions	Gardasil (Merck quadrivalent)	
Outcomes	Anogenital warts Follow‐up: not reported	
Notes	Source of funding: not reported Conflicts of interest: authors include employees of the vaccine developer

**Table:** Subasinghe 2020‐AUS

***Study characteristics***		
Methods	Cross‐sectional study Australia; 2021‐2017 V: National HPV Vaccination Program Register (NHVPR) O: self‐collected vaginal swab for the detection of HPV DNA	
Participants	N = 344 females 16 to 25 years	
Interventions	Gardasil (Merck quadrivalent)	
Outcomes	Prevalent HPV infection Follow‐up: cross‐sectional, follow‐up potentially to 10 years	
Notes	Source of funding: both public/non‐profit and private/industry sources: National Health and Medical Research Council; Merck Sharp & Dohme Conflicts of interest: some authors received funding from the vaccine developer

**Table:** Swedish 2013‐USA

***Study characteristics***		
Methods	Cohort study USA; April 2007‐January 2013 V: medical records (hospital database) O: medical records (hospital database)	
Participants	N = 313 males (MSM) 26 to 76 years	
Interventions	Gardasil (Merck quadrivalent)	
Outcomes	Anogenital warts Follow‐up: up to 4 years	
Notes	Funding: no specific funding Conflicts of interest: some authors received funding from the vaccine developer

**Table:** Tabrizi 2014‐AUS

***Study characteristics***		
Methods	Pre‐ vs post‐vaccine introduction Australia; October 2005‐November 2012 V: National HPV Vaccination Program Register O: exfoliated cervical cells collected for cervical cytology; self‐completed questionnaire	
Participants	N = 1260 females 18 to 24 years	
Interventions	Gardasil (Merck quadrivalent)	
Outcomes	Prevalent HPV infection Follow‐up: cross‐sectional, potential maximum 7 years after vaccination	
Notes	Source of funding: public/non‐profit: Australian National Health and Medical Research Council and Cancer Council Victoria Conflicts of interest: authors include stockholders of the vaccine developer

**Table:** Tanaka 2017‐JPN

***Study characteristics***		
Methods	Cross‐sectional study Japan; January 2014‐October 2016 V: interviews (self‐report) O: cervical cytology/histology (study‐level targeted ascertainment)	
Participants	N = 2425 females (attending cervical screening) 20 to 24 years	
Interventions	Cervarix (GSK bivalent)	
Outcomes	CIN2+ Follow‐up: cross‐sectional	
Notes	Source of funding: not reported Conflicts of interest: no

**Table:** Taniguchi 2019‐JPN

***Study characteristics***		
Methods	Cross‐sectional study Japan; 2015 V: HPV vaccination status was confirmed from public records in the present study: HPV vaccination status was confirmed from public records in the present study O: survey – no other details	
Participants	N = 2727 females 20 to 21 years	
Interventions	Not reported	
Outcomes	Participation rates in screening Follow‐up: cross‐sectional	
Notes	Source of funding: public/non‐profit: Health and Labor Sciences Research Grant Conflicts of interest: some authors received funding from the vaccine developer

**Table:** Tanton 2017‐GBR

***Study characteristics***		
Methods	Pre‐ vs post‐vaccine introduction United Kingdom (UK); 1999‐2012 V: self‐report O: urine samples	
Participants	N = 471 females 18 to 20 years	
Interventions	Cervarix (GSK bivalent)	
Outcomes	Prevalent HPV infection Follow‐up: cross‐sectional	
Notes	Source of funding: public/non‐profit: Medical Research Council; Wellcome Trust; Economic, Social Research Council and Department of Health Conflicts of interest: no

**Table:** Ter‐Minasyan 2024‐ARM

***Study characteristics***		
Methods	Retrospective cohort study Armenia; dates not reported V: Armenian‐American Wellness Center O: Armenian‐American Wellness Center	
Participants	N = 98 females 15 to 40 years	
Interventions	Gardasil (Merck quadrivalent)	
Outcomes	Premature ovarian failure Follow‐up: not reported	
Notes	Funding: not reported Conflict of interest: not reported

**Table:** Thamsborg 2020‐DNK

***Study characteristics***		
Methods	Pre‐ vs post‐vaccine introduction Denmark; January 1999‐December 2018 V: pre‐/post‐vaccination introduction. No individual vaccination status used O: The National Register of Pathology (national database)	
Participants	N = 45,844 females 15 to 25 years	
Interventions	Gardasil (Merck quadrivalent)	
Outcomes	Participation rates in screening; CIN3+; CIN2+; CIN2 Follow‐up: cross‐sectional	
Notes	Source of funding: public/non‐profit: Independent Research Fund Denmark; Danish Health Foundation Conflicts of interest: no

**Table:** Thompson 2016‐CAN

***Study characteristics***		
Methods	Pre‐ vs post‐vaccine introduction Canada; 1990‐2011 V: individual vaccination status not reported O: Manitoba’s administrative databases of Physician Claims and Hospital Discharge Abstracts	
Participants	N = not reported, females and males All ages	
Interventions	Gardasil (Merck quadrivalent)	
Outcomes	Anogenital warts Follow‐up: cross‐sectional, repeated over 22 years	
Notes	Funding: no specific funding Conflicts of interest: no

**Table:** Thomsen 2020‐DNK

***Study characteristics***		
Methods	Cohort study, pre‐ vs post‐vaccine introduction, self‐controlled case series Denmark; January 2008‐December 2014 V: Danish National Health Service Register; Danish National Prescription Registry (national database) O: Danish National Patient Registry and Psychiatric Central Research Register (national database)	
Participants	N = 628,034 females 11 to 17 years	
Interventions	Gardasil (Merck quadrivalent)	
Outcomes	Postural orthostatic tachycardia syndrome (POTS); chronic fatigue syndrome/myalgic encephalomyelitis (CFS/ME); all‐cause mortality Follow‐up: 1 year	
Notes	Source of funding: public/non‐profit: Danish Medicines Agency Conflicts of interest: no

**Table:** Thöne 2017‐DEU

***Study characteristics***		
Methods	Pre‐ vs post‐vaccine introduction Germany; 2005‐2010 V: no individual vaccine status data O: German Pharmacoepidemiological Research Database (insurance database)	
Participants	N > 9,000,000 females and males (29,740,000 person‐years) 10 to 79 years	
Interventions	Cervarix (GSK bivalent), Gardasil (Merck quadrivalent)	
Outcomes	Anogenital warts Follow‐up: 1 year	
Notes	Source of funding: private/industry: Sanofi Pasteur MSD Conflicts of interest: some authors received funding from the vaccine developer

**Table:** Tozawa‐Ono 2021‐JPN

***Study characteristics***		
Methods	Cross‐sectional study Japan; January 2015‐December 2016 V: questionnaire (self‐report) O: cervical cytology and histology (hospital database)	
Participants	N = 11,903 females (attending cervical screening) 20 to 25 years	
Interventions	Cervarix (GSK bivalent), Gardasil (Merck quadrivalent)	
Outcomes	CIN3+; CIN3; CIN2+; CIN2 Follow‐up: 10 years	
Notes	Source of funding: not reported Conflicts of interest: no

**Table:** Tsai 2023‐TWN

***Study characteristics***		
Methods	Retrospective cohort study/database linkage Taiwan; 2013‐2018 V: Taiwan’s National Immunization Information System O: Taiwan’s National Health Insurance Database	
Participants	N = 227,393 vaccinated females 12 to 15 years	
Interventions	Cervarix (GSK bivalent); Gardasil (Merck quadrivalent); Gardasil 9 (Merck nonavalent)	
Outcomes	Primary ovarian failure; chronic fatigue syndrome; Guillain‐Barré syndrome; complex regional pain syndrome Follow‐up: not reported	
Notes	Funding: Health Promotion Administration (HPA), Ministry of Health and Welfare Conflicts of interest: none

**Table:** Van Eer 2021‐NLD

***Study characteristics***		
Methods	Cross‐sectional study Netherlands; 2009‐2017 V: self‐report O: vaginal and anal swabs (self‐collected)	
Participants	N = 542 females Age not reported	
Interventions	Cervarix (GSK bivalent)	
Outcomes	Prevalent HPV infection Follow‐up: cross‐sectional	
Notes	Source of funding: public/non‐profit: Ministry of Health, Welfare and Sports, the Netherlands Conflicts of interest: no

**Table:** Verdoodt 2020‐DNK

***Study characteristics***		
Methods	Cohort study Denmark; 2006‐2016 V: National Health Service Registry (national database) O: Danish national screening programme for cervical cancer (national database)	
Participants	N = 590,083 females 17 to 25 years	
Interventions	Gardasil (Merck quadrivalent)	
Outcomes	CIN3+; CIN2+ Follow‐up: cross‐sectional, longest potential follow‐up, 6 years	
Notes	Source of funding: public/non‐profit: Mermaid Project and the Danish Council for Independent Research (Danmarks Frie Forskningsfond Sapere Aude‐program Conflicts of interest: some authors received funding from the vaccine developer

**Table:** Vielot 2020‐USA

***Study characteristics***		
Methods	Cohort study United States of America (USA); June 2006‐December 2014 V: IBM MarketScan Commercial Database (national database) O: IBM MarketScan Commercial Database (national database)	
Participants	N = 123,981 females 11 to 19 years	
Interventions	Cervarix (GSK bivalent), Gardasil (Merck quadrivalent)	
Outcomes	Complex regional pain syndrome (CRPS) Follow‐up: 8 years	
Notes	Source of funding: public/non‐profit: National Institute of Allergy and Infectious Diseases Conflicts of interest: no

**Table:** Ward 2024‐GBR

***Study characteristics***		
Methods	Retrospective cohort study/regression discontinuity design United Kingdom; 2009‐2022 V: no individual vaccination status used; birth cohorts O: Hospital Episode Statistics	
Participants	N = 1,445,512 females 17 to 22 years	
Interventions	Cervarix (GSK bivalent); Gardasil (Merck quadrivalent)	
Outcomes	Cervical cancer	
Notes	Funding: not reported Conflicts of interest: not reported

**Table:** Wendland 2021‐BRA

***Study characteristics***		
Methods	Cross‐sectional study Brazil; September 2016 to November 2017 V: vaccination status was self‐reported and was independent of the number of doses and intervals O: cervical samples were obtained using a Qiagen HC2 DNA collection device according to the manufacturer’s instructions.	
Participants	N = 5945 females 16 to 25 years	
Interventions	Gardasil (Merck quadrivalent)	
Outcomes	Prevalent HPV infection Follow‐up: cross‐sectional	
Notes	Source of funding: public/non‐profit: Hospital Moinhos de Vento through the Program for Supporting the Institutional Development of the Public Health System (PROADI‐SUS), supported by the Ministry of Health of Brazil Conflicts of interest: authors include employees of the vaccine developer

**Table:** Widdice 2019‐USA

***Study characteristics***		
Methods	Cohort study USA; 2013‐2017 V: self‐report, verified by medical records or vaccine registry in 85% of participants O: swab samples of the glans penis, including coronal sulcus; penile shaft; scrotum; and the perianal/anal area	
Participants	N = 747 males 13 to 26 years	
Interventions	Gardasil (Merck quadrivalent)	
Outcomes	Prevalent HPV infection Follow‐up: cross‐sectional, repeated 2013‐2014 and 2016‐2017	
Notes	Source of funding: public/non‐profit: NIAID; National Institutes of Health Conflicts of interest: some authors received funding from the vaccine developer

**Table:** Willame 2016‐GBR

***Study characteristics***		
Methods	Cohort study United Kingdom (UK); September 2005‐August 2010 V: Clinical Practice Research Datalink (national database) O: Clinical Practice Research Datalink (national database) and/or Hospital Episode Statistics (HES) (national database and routine administrative database, hospital)	
Participants	N = 259,876 females and males 9 to 25 years	
Interventions	Cervarix (GSK bivalent)	
Outcomes	Guillain‐Barré syndrome (GBS) Follow‐up: 1 year	
Notes	Source of funding: private/industry: GlaxoSmithKline Biologicals Conflicts of interest: authors include employees of the vaccine developer

**Table:** Willows 2018‐CAN

***Study characteristics***		
Methods	Cohort study Canada; August 2001‐December 2017 V: The Manitoba Immunization Monitoring System (MIMS) (regional database) O: hospital, physician and prescription claim databases	
Participants	N = 125,791 females ≥ 9 years	
Interventions	Gardasil (Merck quadrivalent)	
Outcomes	Anogenital warts Follow‐up: 16 years	
Notes	Source of funding: private/industry: Merck Canada Inc. Conflicts of interest: some authors received funding from the vaccine developer

**Table:** Winer 2021‐USA

***Study characteristics***		
Methods	Cross‐sectional study USA; 2016‐2018 V: self‐report O: self‐collected penile swab specimen	
Participants	N = 687 (penile), 1391 (oral/anal) males 18 to 26 years	
Interventions	Gardasil (Merck quadrivalent)	
Outcomes	Prevalent HPV infection Follow‐up: cross‐sectional	
Notes	Source of funding: public/non‐profit: Centers for Disease Control and Prevention Conflicts of interest: no

**Table:** Wissing 2019‐CAN

***Study characteristics***		
Methods	Cohort study Canada; May 2005‐February 2011 V: web‐based questionnaires O: during clinical visits, genital specimens were collected, either by self‐sampling (vaginal samples) or by a nurse (penile samples of male partners)	
Participants	N = 502 females ≥ 18 years	
Interventions	Gardasil (Merck quadrivalent)	
Outcomes	Incident HPV infection; persistent HPV infection; prevalent HPV infection Follow‐up: 2 years	
Notes	Source of funding: both public/non‐profit and private/industry sources: Canadian Institutes of Health Research; U.S. National Institutes of Health; Merck‐Frosst Canada Ltd., and Merck & Co. Ltd; Reseau sida et maladies infectieuses (SIDA/MI) du Fonds de recherche du Quebec ‐ Sante (FRQS) Conflicts of interest: some authors received funding from the vaccine developer

**Table:** Woestenberg 2020‐NLD

***Study characteristics***		
Methods	Cross‐sectional study Netherlands; 2011‐2017 V: self‐report O: anal swabs	
Participants	N = 548 females 16 to 24 years	
Interventions	Cervarix (GSK bivalent)	
Outcomes	Prevalent HPV infection Follow‐up: cross‐sectional. Up to 8 years.	
Notes	Source of funding: public/non‐profit: Ministry of Health, Welfare and Sport, the Netherlands Conflicts of interest: no

**Table:** Woestenberg 2021‐NLD

***Study characteristics***		
Methods	Cohort study Netherlands; January 2007‐December 2015 V: national immunisation registry (Præventis) (national database) O: Nivel Primary Care Database (Nivel‐PCD) (national database)	
Participants	N = 96,468 females (primary health care) Age not reported	
Interventions	Cervarix (GSK bivalent)	
Outcomes	Anogenital warts Follow‐up: median 3 years	
Notes	Source of funding: public/non‐profit: Netherlands Ministry of Health, Welfare and Sport Conflicts of interest: unclear

**Table:** Wright 2019‐USA

***Study characteristics***		
Methods	Cross‐sectional study USA; August 2013‐June 2015 V: self‐report O: HPV testing, standardised colposcopy and biopsy protocols (study‐level targeted ascertainment)	
Participants	N = 14,153 females (attending cervical screening) 21 to 34 years	
Interventions	Gardasil (Merck quadrivalent)	
Outcomes	CIN3+; CIN2+; prevalent HPV infection Follow‐up: 10 years	
Notes	Source of funding: private/industry: Becton, Dickinson and Company, BD Life Sciences Conflicts of interest: some authors received funding from the vaccine developer

**Table:** Xu 2021‐GBR

***Study characteristics***		
Methods	Pre‐ vs post‐vaccine introduction United Kingdom (UK); 2006‐2016 V: no individual vaccination status O: Aberdeen Maternity and Neonatal Databank (routine administrative database, hospital)	
Participants	N = not reported, females 20 to 30 years	
Interventions	Cervarix (GSK bivalent)	
Outcomes	Birth outcomes Follow‐up: not reported	
Notes	Source of funding: public/non‐profit: Newton visiting PhD fellowship Conflicts of interest: some authors received funding from the vaccine developer

**Table:** Yagi 2019‐JPN

***Study characteristics***		
Methods	Cohort study Japan; 2011‐2016 V: no individual vaccine status data: pre‐/post‐eligibility birth cohorts O: cervical screening data (hospital database)	
Participants	N = 15,261 females (attending cervical screening) 20 to 21 years	
Interventions	Cervarix (GSK bivalent), Gardasil (Merck quadrivalent)	
Outcomes	CIN3+; CIN3; CIN2+; CIN2; participation rates in screening Follow‐up: cross‐sectional	
Notes	Source of funding:public/non‐profit: Health and Labor Sciences Research Grant Conflicts of interest: some authors received funding from the vaccine developer

**Table:** Yoon 2021‐KOR

***Study characteristics***		
Methods	Cohort study; self‐controlled case series South Korea; January 2017‐December 2019 V: Korea Immunization Registry Information System (national database) O: National Health Information Database (national database)	
Participants	N = 441,399 females 11 to 14 years	
Interventions	Cervarix (GSK bivalent), Gardasil (Merck quadrivalent)	
Outcomes	Paralysis; Guillain‐Barré syndrome (GBS) Follow‐up: 3 years	
Notes	Source of funding: public/non‐profit: Government‐wide R&D Fund project for infectious disease research (GFID), Republic of Korea Conflicts of interest: no

**Table:** Zeybek 2018‐USA

***Study characteristics***		
Methods	Cohort study USA; 2006‐2015 V: Clinformatics Data Mart (CDM) Database (insurance database) O: Clinformatics Data Mart (CDM) Database (insurance database)	
Participants	N = 573,926 females and males Age not reported	
Interventions	Gardasil (Merck quadrivalent)	
Outcomes	Anogenital warts Follow‐up: up to 5 years	
Notes	Source of funding: public/non‐profit: William & Mary McGanity Research Fund Award from the Department of Obstetrics & Gynecology at The University of Texas Medical Branch at Galveston Conflicts of interest: no	

O: source of outcome data; V: source of vaccination data **Other abbreviations:** AGW: anogenital warts; AIN: anal intraepithelial neoplasia; CFS/ME: chronic fatigue syndrome/myalgic encephalomyelitis; CIN: cervical intraepithelial neoplasia; CIN2: cervical intraepithelial neoplasia grade 2; CIN2+: cervical intraepithelial neoplasia grade 2 or higher; CIN3: cervical intraepithelial neoplasia grade 3; CIN3+: cervical intraepithelial neoplasia grade 3 or higher; CRPS: complex regional pain syndrome; EMR: electronic medical record; GP: general practitioner; GUM: genitourinary medicine; GW: genital warts; HPV: human papillomavirus; ICD‐9/ICD‐10: International Statistical Classification of Diseases and Related Health Problems (9th/10th Revision); IQR: interquartile range; MSM: men who have sex with men; MSW: men who have sex with women; NR: not reported; POTS: postural orthostatic tachycardia syndrome; RCT: randomised controlled trial; SD: standard deviation; STD: sexually transmitted disease; STI: sexually transmitted infection; VaIN: vaginal intraepithelial neoplasia; VIN: vulval intraepithelial neoplasia; WSM: women who have sex with men; yo: year‐old

### Characteristics of excluded studies [ordered by study ID]

**Table:**

Study	Reason for exclusion
Abbas 2023a	No relevant comparison
Abbas 2023b	No relevant comparison
Ai Sahlgren 2015	Irrelevant population – already attending screening with positive result
Amend 2022	No relevant comparison
An 2024	No relevant comparison
Ansstasiou‐Fotaki 2007	Study design not relevant
Aujo 2014	No relevant outcome measures
Bailoni 2024	No relevant population
Barry 2024	Study design not relevant
Bayes 2011	No relevant comparison
Bhardwaj 2022	No relevant population
Bhatla 2023	No relevant outcome measures
Block 2009	Study design not relevant
Boudova 2023	No relevant comparison
Brogly 2014	No relevant outcome measures
Brouwer 2019	No relevant outcome measures
Brouwer 2022a	No relevant outcome measures
Brouwer 2022b	No relevant outcome measures
Caskey 2022	Study design not relevant
Castillo 2019	No relevant outcome measures
Castillo‐Cano 2022	No relevant outcome measures
Chambers 2023	No relevant outcome measures
Chao 2012	No relevant comparison
Chaopotong 2024	No relevant outcome measures
Chen 2022	No relevant comparison
Chidambaram 2023	No relevant outcome measures
Chou 2022	No relevant outcome measures
Cocores 2023	No relevant comparison
Craig 2023	No relevant comparison
Crawley 2022	No relevant outcome measures
Dalla 2024	No relevant comparison
Davis 2024	No relevant outcome measures
Dehlendorff 2021	No relevant outcome measures
Dey 2022	No relevant comparison
Di Lorenzo 2022	No relevant comparison
Donahue 2019	No relevant comparison
Donken 2018	No relevant outcome measures
Ehret 2023	No relevant population
Eun 2023	No relevant comparison
Fan 2023	Study design not relevant
Fappani 2021	No relevant population
Fatima 2022	No relevant outcome measures
Fernandez‐Feito 2018	No relevant outcome measures
Fisher 2023	No relevant outcome measures
Forster 2012	No relevant outcome measures
Freire‐Salinas 2021	No relevant data available for extraction – unable to determine denominators
Frio 2021	No relevant outcome measures
Garces 2022	Study design not relevant
Gardella 2023	Irrelevant population (all with CIN)
Garland 2022	No relevant outcome measures
Gee 2011	Study design not relevant – denominators are total adverse events
Geier 2015	Study design not relevant – denominators are total adverse events
Geier 2017	Study design not relevant – denominators are total adverse events
Gholamzad 2024	No relevant outcome measures
Gibson 2022	No relevant outcome measures
Grimaldi‐Bensouda 2023	No relevant outcome measures
Groom 2023	No relevant outcome measures
Grun 2015	No relevant outcome measures
Grun 2016	No relevant outcome measures
Guido 2020	No relevant outcome measures
Guiqian 2020	No relevant outcome measures
Guo 2022	No relevant outcome measures
Hallam 2020	Irrelevant population – already attending screening with positive result
Han 2017	No relevant outcome measures
Hansen 2014	No relevant outcome measures
Hansen 2023	No relevant comparison
Hariri 2015a	Irrelevant population
Hariri 2015b	Irrelevant population – all with cervical disease
Hategeka 2020	No relevant outcome measures
Hernandez‐Aguado 2022	No relevant outcome measures
Hoes 2021	No relevant comparison
Hofstetter 2016	No relevant outcome measures
Holy 2024	No relevant comparison
Iftner 2010	No relevant outcome measures
Issanov 2022	No relevant outcome measures
Jacobs 2024	No relevant outcome measures
Johnson 2020	Irrelevant population – all with high‐grade cervical lesions
Joshi 2023	No relevant outcome measures
Karachentsova 2024	Study design not relevant
Kenigsberg 2023	No relevant comparison
Kerry‐Barnard 2021	No relevant comparison
Klein 2024	Study design not relevant
Krog 2024	Irrelevant population (all CIN2)
Kwak 2024	Study design not relevant
Lang 2023	Study design not relevant
Lee K 2024	No relevant comparison
Lee P 2024	Study design not relevant
Lee S 2024	No relevant outcome measures
Leidner 2020	No relevant outcome measures
Liang 2022	No relevant outcome measures
Liao 2022	No relevant outcome measures
Lindquist 2024	No relevant comparison
Loerinc 2023	No relevant population
Lonky 2021	Irrelevant population – already attending screening with positive result
Lopez‐Codony 2024	No relevant population
Lynge 2024	No relevant outcome measures
Magdaleno‐Tapial 2022	No relevant comparison
Mahmud 2014	No relevant outcome measures
Maldonado 2022	No relevant comparison
Maldonado 2024	No relevant outcome measures
Man 2023	Study design not relevant (modelling)
Marchand 2013	No relevant outcome measures
Matsumoto 2014	No relevant outcome measures
Matsumoto 2017	No relevant outcome measures
Matsumoto 2019	No relevant outcome measures
Mattis 2023	No relevant comparison
McClung 2019	No relevant population
Megumi 2023	No relevant comparison
Mehlsen 2022	No relevant comparison
Meng 2023	No relevant comparison
Mesher 2021	No relevant outcome measures
Miranda 2024	No relevant comparison
Mix 2021	No relevant population
Mo 2024	No relevant comparison
Morais 2024	No relevant outcome measures
Munk 2024	No relevant comparison
Murall 2020	No relevant outcome measures
Murenzi 2023	Study design not relevant
Na 2024	No relevant population
Nakalembe 2014	No relevant outcome measures
Naleway 2023	Study design not relevant
Nasreen 2023	No relevant outcome measures
Niccolai 2017	No relevant comparison
Ntanika 2023	No relevant outcome measures
Ogilvie 2018	No relevant outcome measures
Oh 2024	No relevant outcome measures
Onuki 2022a	Irrelevant population (all with CIN)
Onuki 2022b	Irrelevant population (all with CIN)
Paavonen 2009	Study design not relevant
Panwar 2022	No relevant outcome measures
Passos 2022	No relevant outcome measures
Pesut 2024	No relevant comparison
Petry 2013	No relevant outcome measures
Pimenoff 2023	Study design not relevant
Powell 2012	No relevant outcome measures
Qiu 2024	No relevant outcome measures
Ramogola‐Masire 2022	No relevant population
Ratanasiripong 2014	No relevant outcome measures
Restrepo 2023	No relevant comparison
Righolt 2019	No relevant outcome measures
Rossotti 2024	Irrelevant population (all vaccinated)
Rotert 2022	Irrelevant population (unclear vaccination proportion)
Rourke 2024	No relevant outcome measures
Sastre‐Canton 2019‐ESP	No relevant outcomes
Satanova 2024	No relevant outcome measures
Seeger 2023	No relevant outcome measures
Sehnal 2022	Study design not relevant
Seoud 2022	No relevant comparison
Serafini 2024	No relevant outcome measures
Sheth 2024	Study design not relevant
Shin 2022	No relevant outcome measures
Shing 2024	No relevant outcome measures
Sivars 2023	Irrelevant population (all with SCC)
Sonnenberg 2013	No relevant outcome measures
Stefanizzi 2023	No relevant comparison
Sundaram 2022	No relevant comparison
Svarrer 2019	No relevant outcome measures
Tan 2023	No relevant comparison
Tarrash 2023	Study design not relevant
Tatang 2021	No relevant outcome measures
Teoh 2022	Irrelevant population (all with abnormal cytology)
Trenque 2022	No relevant outcome measures
Tsang 2022	No relevant outcome measures
Tsukamoto 2022	Study design not relevant
Valle 2022	No relevant comparison
Van Eer 2023	No relevant outcome measures
Van Trang 2022	No relevant outcome measures
Velentzis 2023	Study design not relevant
Wang 2024	No relevant outcome measures
Wei 2022	No relevant outcome measures
Welby 2023	Study design not relevant
Wu 2023	No relevant comparison
Yagi 2024	Study design not relevant (modelling)
Yasuda 2024	Study design not relevant
Zhang 2023	No relevant comparison
Zhang 2024	No relevant comparison
Zhao 2023	Irrelevant study design (RCT)
Zheng 2022	No relevant comparison

CIN: cervical intraepithelial neoplasia; CIN2: cervical intraepithelial neoplasia grade 2; RCT: randomised controlled trial; SCC: squamous cell carcinoma

### Characteristics of studies awaiting classification [ordered by study ID]

**Table:** De Kloe 2024

Methods	Retrospective cohort study
Participants	Patients 9 to 39 years old attending medical encounters
Interventions	HPV vaccination
Outcomes	Malignancies at the following sites: head and neck (HNC), cervix, anus and anal canal, penis, vulva and vagina
Notes	Conference abstract with insufficient details about population for inclusion. Awaiting full publication.

**Table:** Dominicci‐Maura 2024

Methods	Prospective cohort study
Participants	Women aged 21 to 50 attending gynaecology and colonoscopy clinics
Interventions	HPV vaccination
Outcomes	Any HPV, high‐risk HPV, low‐risk HPV and specific HPV types
Notes	Conference abstract with insufficient details about population for inclusion. Awaiting full publication.

**Table:** Elshourbagy 2022

Methods	Self‐controlled case series
Participants	Patients with Guillain‐Barré syndrome in Vaccine Adverse Event Reporting System
Interventions	HPV vaccination
Outcomes	Guillain‐Barré syndrome
Notes	Conference abstract with insufficient details about population for inclusion. Awaiting full publication.

**Table:** Lau 2023

Methods	Prospective cohort study
Participants	Patients evaluated for anal dysplasia by two colorectal surgeons
Interventions	HPV vaccination
Outcomes	Anal dysplasia rates
Notes	Conference abstract with insufficient detail in outcomes reported for inclusion. Awaiting full publication.

**Table:** Neerukonda 2023

Methods	Ecologic study
Participants	Females in Florida and New York
Interventions	HPV vaccination
Outcomes	Cervical cancer incidence and death
Notes	Conference abstract with insufficient details about population for inclusion. Awaiting full publication.

HNC: head and neck cancer; HPV: human papillomavirus

## Differences between protocol and review

We have included an additional secondary outcome, prevalent HPV infection, as this was omitted from the protocol. The review now includes incident HPV infection, prevalent HPV infection and persistent HPV infection as secondary outcomes.

We have removed outcomes about lesions associated with HPV types included in the vaccines to reduce the overall number of outcomes and focus the review on overall rates of lesions and cancers associated with HPV.

We planned to use all studies we identified as relevant as seeds in the Science Citation Index ISI Web of Knowledge ResearchGate and Google Scholar to determine whether articles citing these studies were also relevant. Based on the large number of studies already identified by the search, we considered this step to be an unnecessary amount of work for very little yield.

Analysis was also planned for unadjusted data, however these were considered at critical risk of bias and therefore we limited analysis to adjusted effect estimates.

We performed an additional subgroup analysis of adjusted estimates of effect in those receiving the HPV vaccine at age 16 or younger. RCTs of HPV vaccine have demonstrated better efficacy in younger age groups before the onset of sexual activity, and most community HPV vaccine programmes are designed for younger age groups. Catch‐up programmes in older girls are therefore likely to lead to an underestimate of effect, hence this additional analysis was included.
